# Supplementary figures and images for: Manganese is a potent inducer of lysosomal activity that inhibits de novo HBV infection (part 1 of 2)
Source: PLoS Pathog. 2025 Jan 2;21(1):e1012800. doi: 10.1371/journal.ppat.1012800 (PMC11694974; doi:10.1371/journal.ppat.1012800)

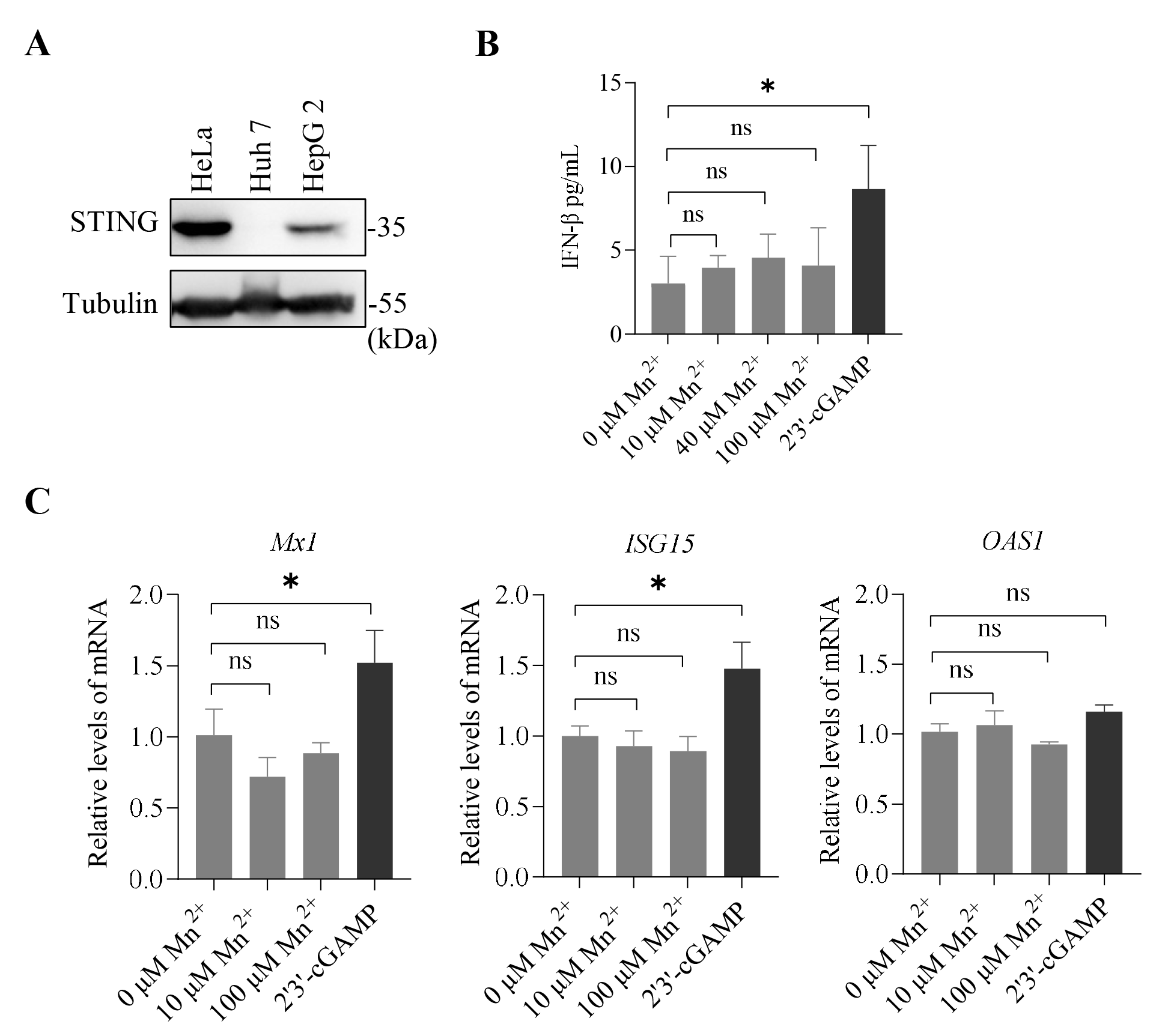

Supplement: S1 Fig — (A) The STING protein was determined in HeLa, Huh7 or HepG2 cells by immunoblotting. (B) HepG2 cells were treated with the indicated concentrations of MnCl2 for 24 h, followed by incubation in fresh medium for an additional 24 h. The supernatant IFN-β was analyzed by ELISA (n = 3). Control, cells treated with cGAMP (10 μg/mL). (C) HepG2 cells were treated with indicated concentrations of MnCl2 or cGAMP for 8 h, followed by quantitative reverse transcription PCR using Mx1-, ISG15-, OAS1-specific primers (n = 3). Values show the mean ± SD. *, P < 0.05; ns, not significant. (TIF) [file ppat.1012800.s001.tif]

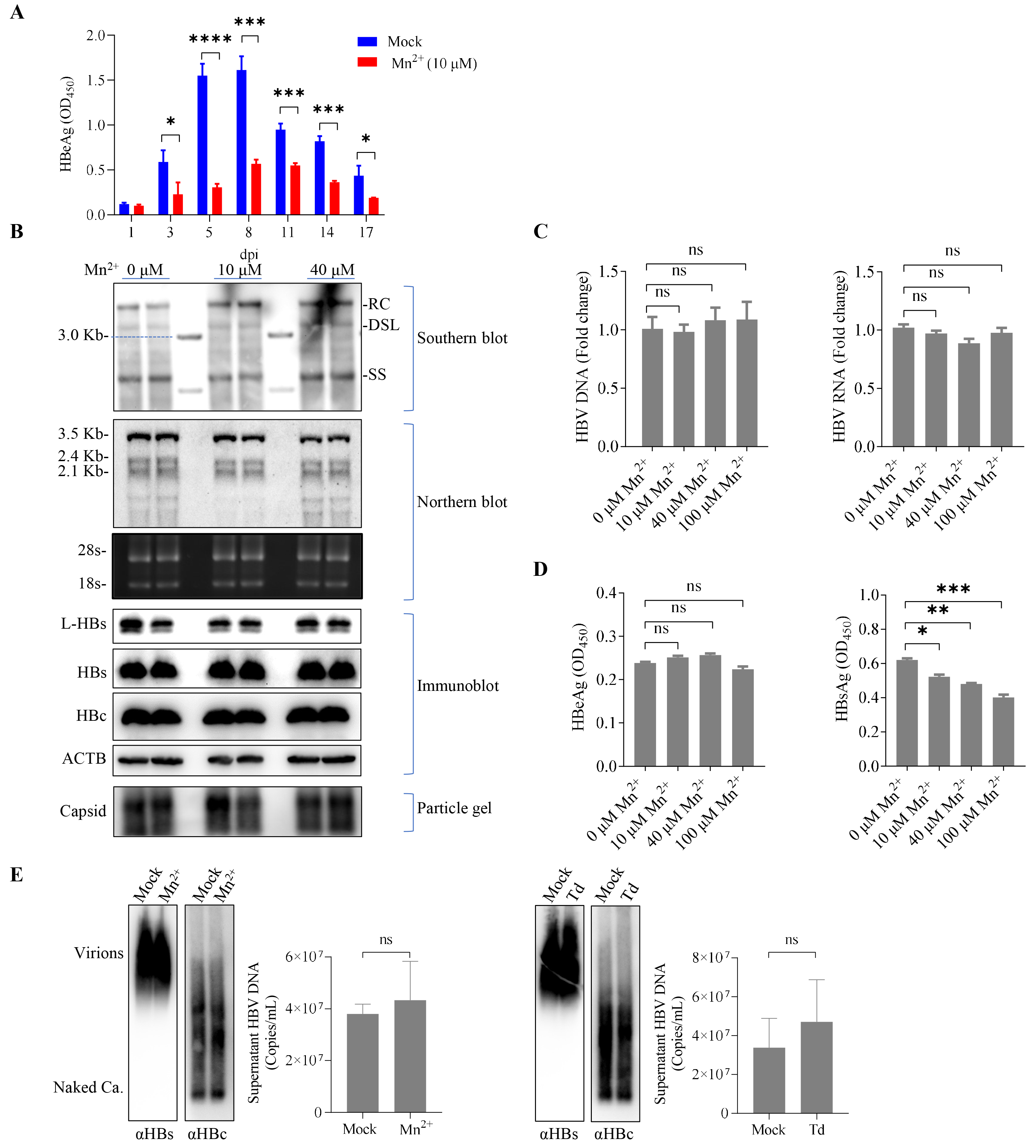

Supplement: S2 Fig — (A) HepG2-NTCP cells were treated with or without 10 μM MnCl2 for 10 h, followed by HBV inoculation as depicted in Fig 1A. Secreted HBeAg was determined by ELISA at the indicated time points (n = 3). (B) HBV replication was induced in HepAD38 cells by removing doxycycline (tet-off) for 72 h. Cells were then treated with MnCl2 at indicated concentrations for an additional 72 h, followed by Southern blotting for intracellular viral DNA species, northern blotting for viral RNA transcripts, immunoblotting for viral proteins, and the particle gel assay for viral capsids, respectively. (C) Quantitative PCR analysis of intracellular HBV DNA (left) or RNA (right) in HepAD38 cells treated with MnCl2 as depicted in (B) (n = 3). (D) ELSIA of HBeAg or HBsAg in the supernatant of HepAD38 cells as depicted in (B) (n = 3). (E) Huh7 cells transfected with pHBV1.3 were treated with 40 μM MnCl2 (left panels) or 10 μM tomatidine (Td, right panels) in fresh medium for 72 h. Viral particles in the supernatant were concentrated by PEG8000 precipitation and resolved by agarose gel electrophoresis. Enveloped virions and naked capsids were detected by immunoblotting with the indicated antibodies. Virion/capsid-associated DNA was determined by quantitative real-time PCR (n = 3). Error bars indicate the mean ± SD. *, P < 0.05; **, P < 0.01; ***, P < 0.001; ****, P < 0.0001; ns, not significant. (TIF) [file ppat.1012800.s002.tif]

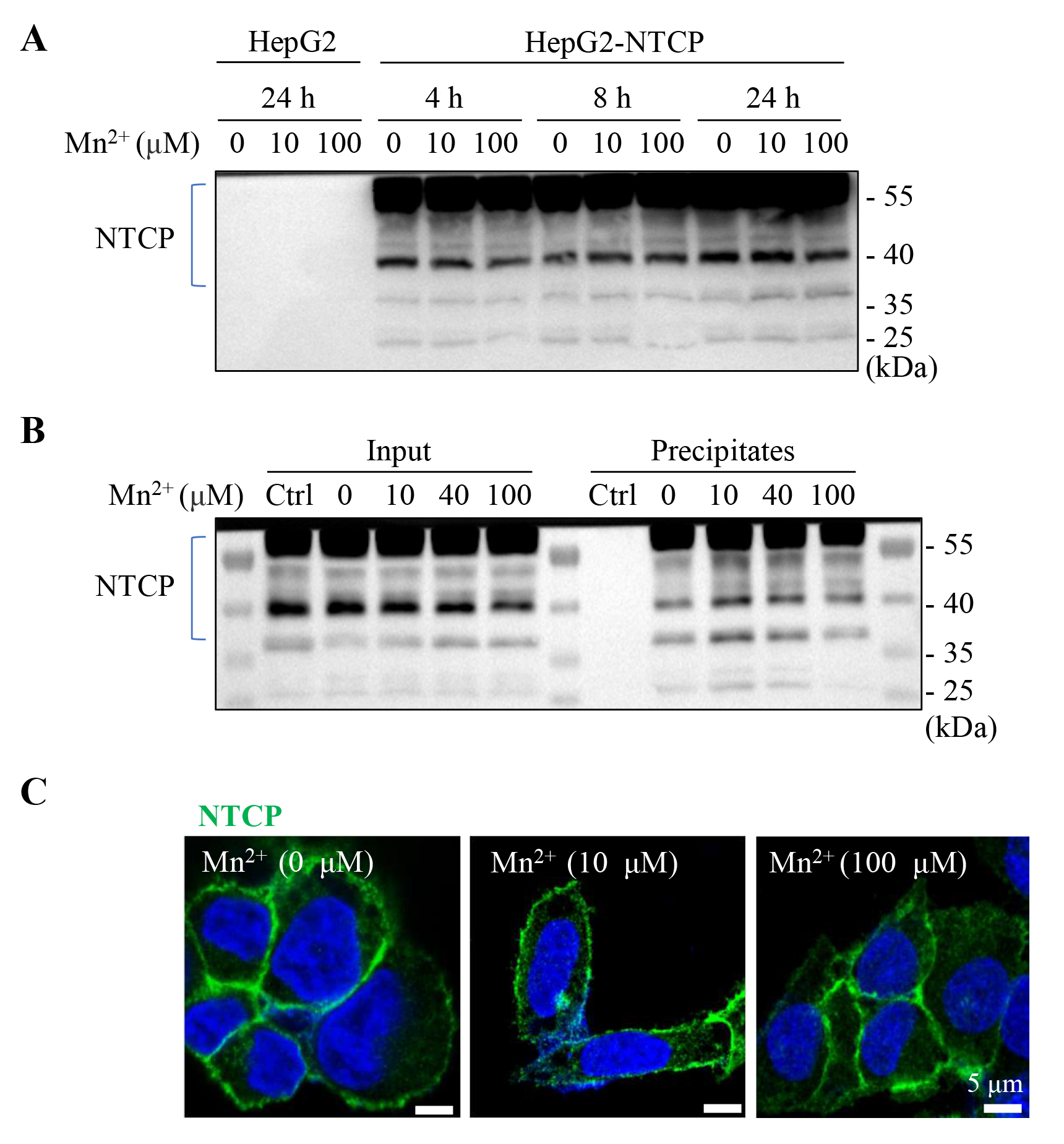

Supplement: S3 Fig — (A) HepG2-NTCP cells were treated with the indicated concentrations of MnCl2 for 4, 8, or 24 h, followed by immunoblotting with anti-NTCP. HepG2 cells served as negative control. (B) HepG2-NTCP cells were treated with indicated concentrations of MnCl2 for 8 h. Biotin-labeled cell surface proteins were precipitated on streptavidin-agarose beads and subjected to immunoblotting. (C) Immunofluorescence confocal microscopy of the subcellular distribution of NTCP in HepG2-NTCP cells treated with MnCl2 at the indicated concentrations for 12 h. Scale bar = 5 μm. Blue, DAPI; Green, NTCP. (TIF) [file ppat.1012800.s003.tif]

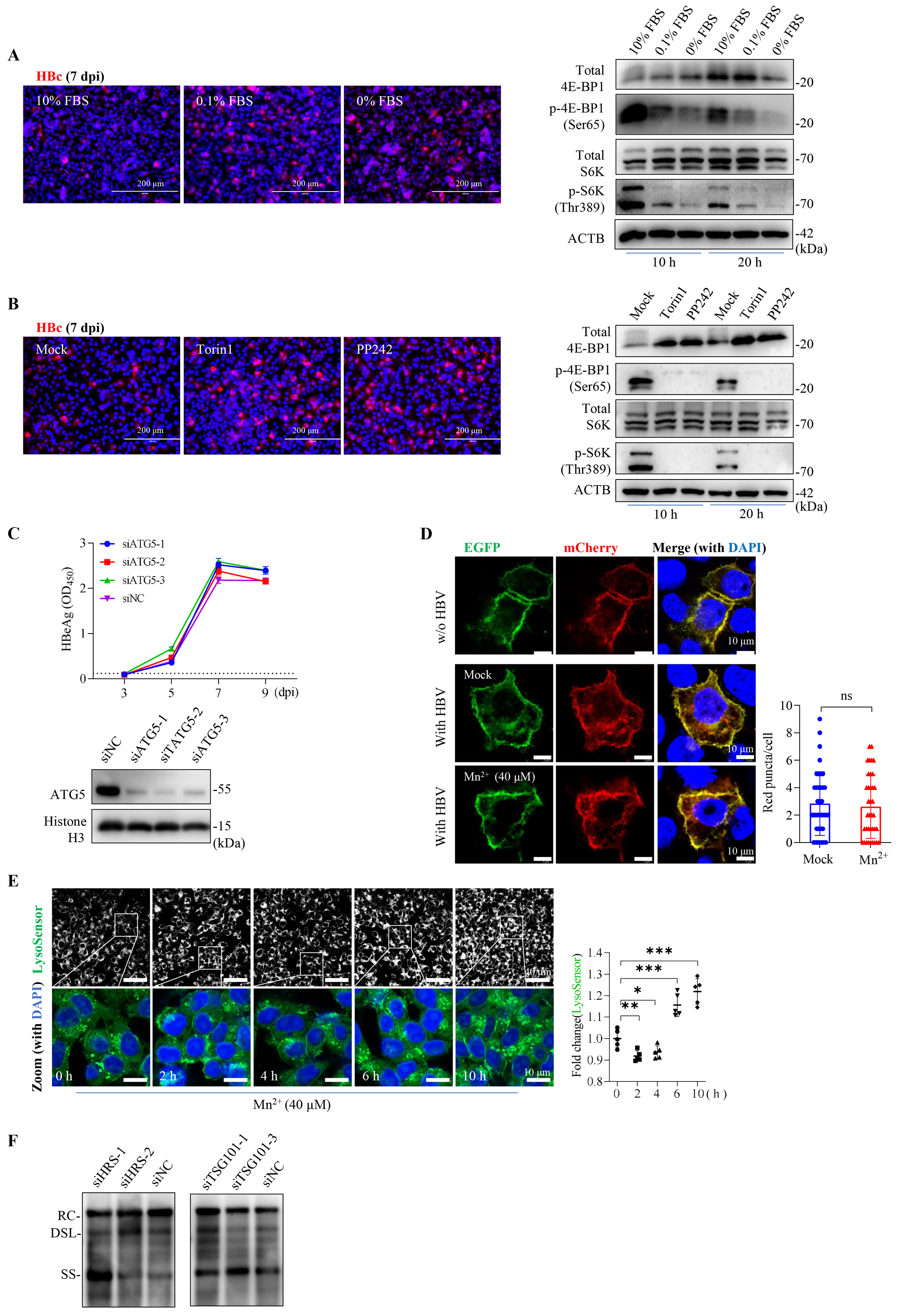

Supplement: S4 Fig — (A) HepG2-NTCP cells were cultured in DMEM medium containing FBS at the indicated concentrations for 10 h, followed by a further 12-h incubation with HBV inoculum. Left, representative immunofluorescence images of intracellular HBc (red) at 7 dpi. Right, mTORC1 activity was assessed by immunoblotting of phosphorylated S6K and 4E-BP1. (B) HepG2-NTCP cells cultured in DMEM medium with 10% FBS were treated with Torin1 (1 μM) or PP242 (1 μM) for 10 h, followed by another 12-h incubation with HBV inoculum (in the presence of the inhibitors). Cells were examined as described in (A). Control, cells mock-treated with DMSO. (C) Knockdown of ATG5 does not suppress de novo HBV infection. HepG2-NTCP cells were transfected with ATG5-specific siRNAs for 48 h, followed by incubation with HBV inoculum for 12 h. Top, secreted HBeAg was determined by ELISA at the indicated time points (n = 3). The dotted line represents the cutoff value. Bottom, the knockdown efficiency of each siRNA was assessed by immunoblotting. siNC, negative control siRNA. (D) Huh7 cells were transiently transfected with a plasmid expressing NTCP-EGFP-mCherry and treated with or without 40 μM MnCl2 for 2 h (corresponding to peak mTORC1 activation (Fig 2C), although lysosomal acidification had not yet increased (see below)). The cells were then inoculated with HBV for 1 h and analyzed by confocal microscopy (left). Red puncta in each cell were counted across 30 fields of view in two independent experiments (right). (E) Representative images of LysoSensor-stained Huh7 cells treated with 40 μM MnCl₂ for the indicated durations are shown (left). Integrated density from five low-magnification (40×) fields of view was quantified using ImageJ (right). (F) HepAD38 tet-off cells were transfected with siRNA against HRS or TSG101 for 3 days. Intracellular viral DNA was detected by Southern blotting. RC, relaxed circular DNA; DSL, double-stranded linear DNA; SS, single-stranded linear DNA. (TIF) [file ppat.1012800.s004.tif]

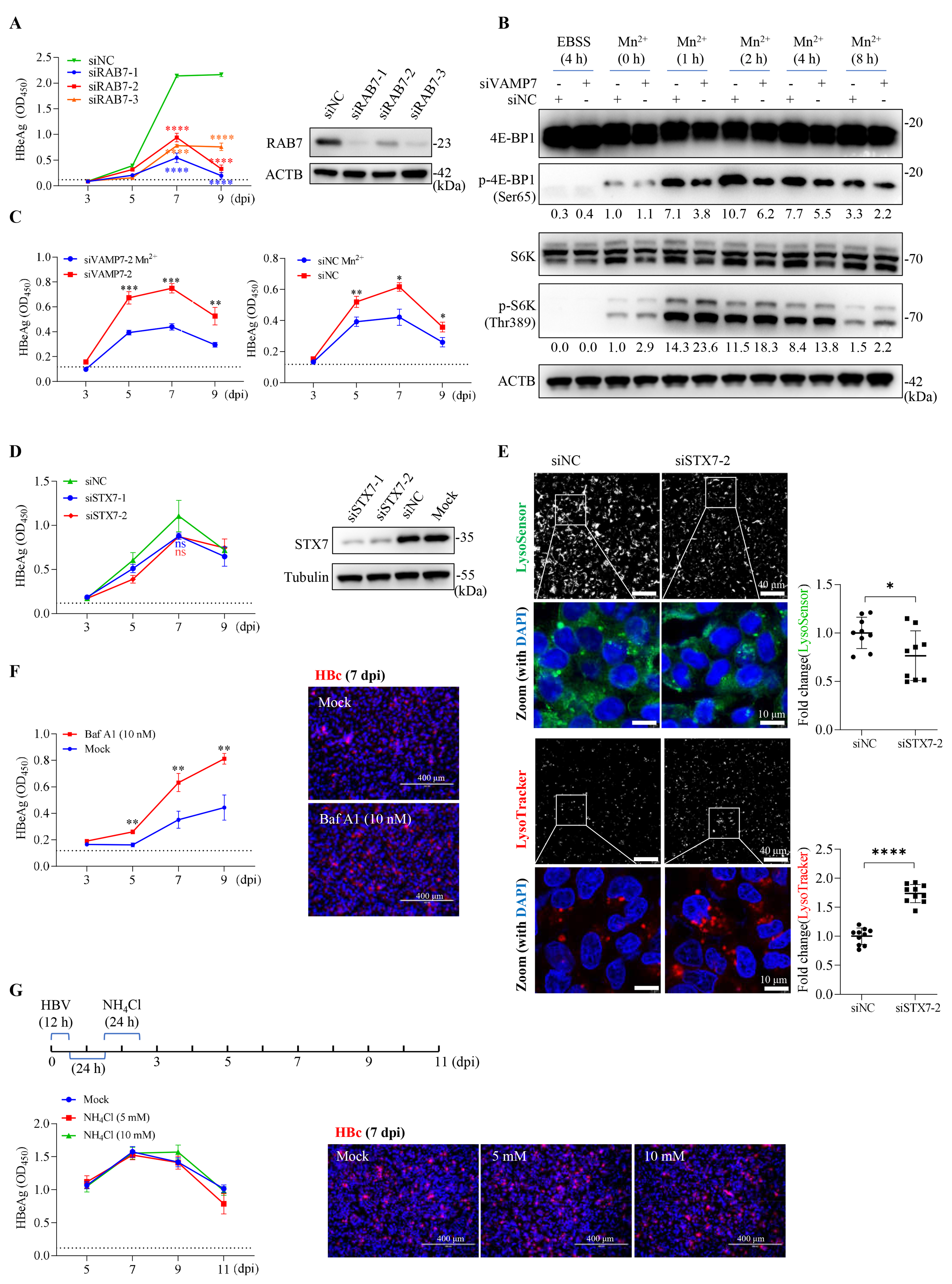

Supplement: S5 Fig — (A) HepG2-NTCP cells were transfected with RAB7-specific siRNAs for 48 h, followed by incubation with HBV inoculum for 12 h. Left, secreted HBeAg was determined by ELISA at the indicated time points (n = 3). Right, the knockdown efficiency of each siRNA was assessed by immunoblotting. siNC, negative control siRNA. (B) 48 h after siVAMP7-2 transfection, HepG2-NTCP cells were treated with MnCl2 (10 μM) for the indicated durations, followed by immunoblotting with the specified antibodies. The blots were analyzed by densitometry, and the intensity of the phospho-protein signals was normalized to the corresponding total protein bands. Cells starved in EBSS medium for 4 h were used as a control for mTORC1 inactivation. (C) HepG2-NTCP cells transfected with siVAMP7-2 (left) or siNC (right) were treated with 40 μM MnCl2 for 10 h, followed by an additional 12 h incubation with HBV inoculum. Secreted HBeAg was determined at the indicated time points (n = 3). (D) HepG2-NTCP cells were transfected with STX7-specific siRNAs for 48 h and analyzed as described in (A). (E) HepG2-NTCP cells were transfected with siSTX7-2 for 48 h. Representative images of cells stained with LysoSensor (top) and LysoTracker (bottom) probes are shown. The integrated density of 9–10 low-magnification (40×) fields of view was calculated using ImageJ. (F) HepG2-NTCP cells were treated 10 nM BafA1 for 10 h, followed by HBV infection for an additional 12 h. Left, the supernatant HBeAg was measured by ELISA at the indicated time points (n = 3). Right, representative immunofluorescence images of intracellular HBc (red) at 7 dpi. (G) HepG2-NTCP cells were inoculated with HBV for 12 h and then cultured in fresh medium for an additional 24 h. Cells were subsequently treated with NH4Cl at the indicated concentrations for another 24 h. Top, experimental scheme. Bottom left, secreted HBeAg was determined at the indicated time points (n = 3). Bottom right, representative images of immunofluorescence labeling of int [file ppat.1012800.s005.tif]

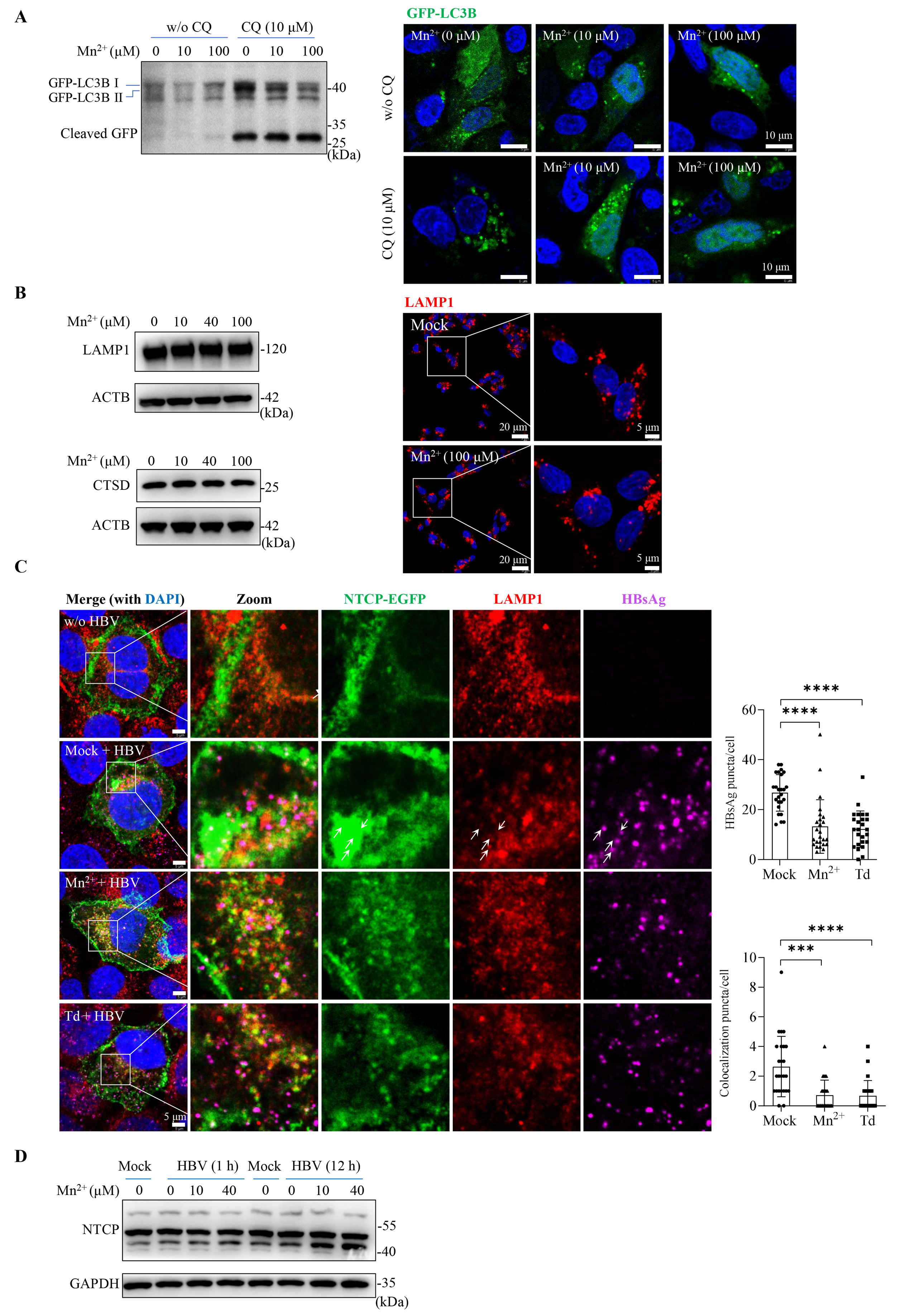

Supplement: S6 Fig — (A) HepG2 cells that stably express GFP-LC3B were treated with indicated concentrations of MnCl2 for 24 h with or without 10 μM of CQ. Left, GFP-LC3B determined by immunoblotting with anti-GFP. Right, representative images of GFP-LC3B by immunofluorescence confocal microscopy. (B) HepG2-NTCP cells were treated with indicated concentrations of MnCl2 for 12 h. Left, endogenous LAMP1 and cleaved CTSD determined by immunoblotting. Right, subcellular distribution of LAMP1 assessed by immunofluorescence confocal microscopy. (C) NTCP-EGFP–expressing Huh7 cells were treated with MnCl2 (40 μM) or tomatidine (Td, 10 μM) for 10 h. After inoculation with HBV for 1 h, cells were stained with anti-HBsAg and anti-LAMP1, and analyzed by immunofluorescence confocal microscopy. Left, representative images from two independent experiments. White puncta (arrows) indicate the colocalization of HBsAg (pink), LAMP1 (red), and NTCP-EGFP (green). Right, the intracellular pink puncta, as well as the white puncta resulting from the merging of the three colors, were quantified per cell from a total of 25 cells in each group. (D) HepG2-NTCP cells were pre-treated with MnCl2 at the indicated concentrations for 10 h, followed by the stimulation with HBV inoculum for either 1 h or 12 h (in the continued presence of MnCl2). NTCP expression was then assessed by immunoblotting. Error bars indicate the mean ± SD. ***, P < 0.001; ****, P < 0.0001. (TIF) [file ppat.1012800.s006.tif]

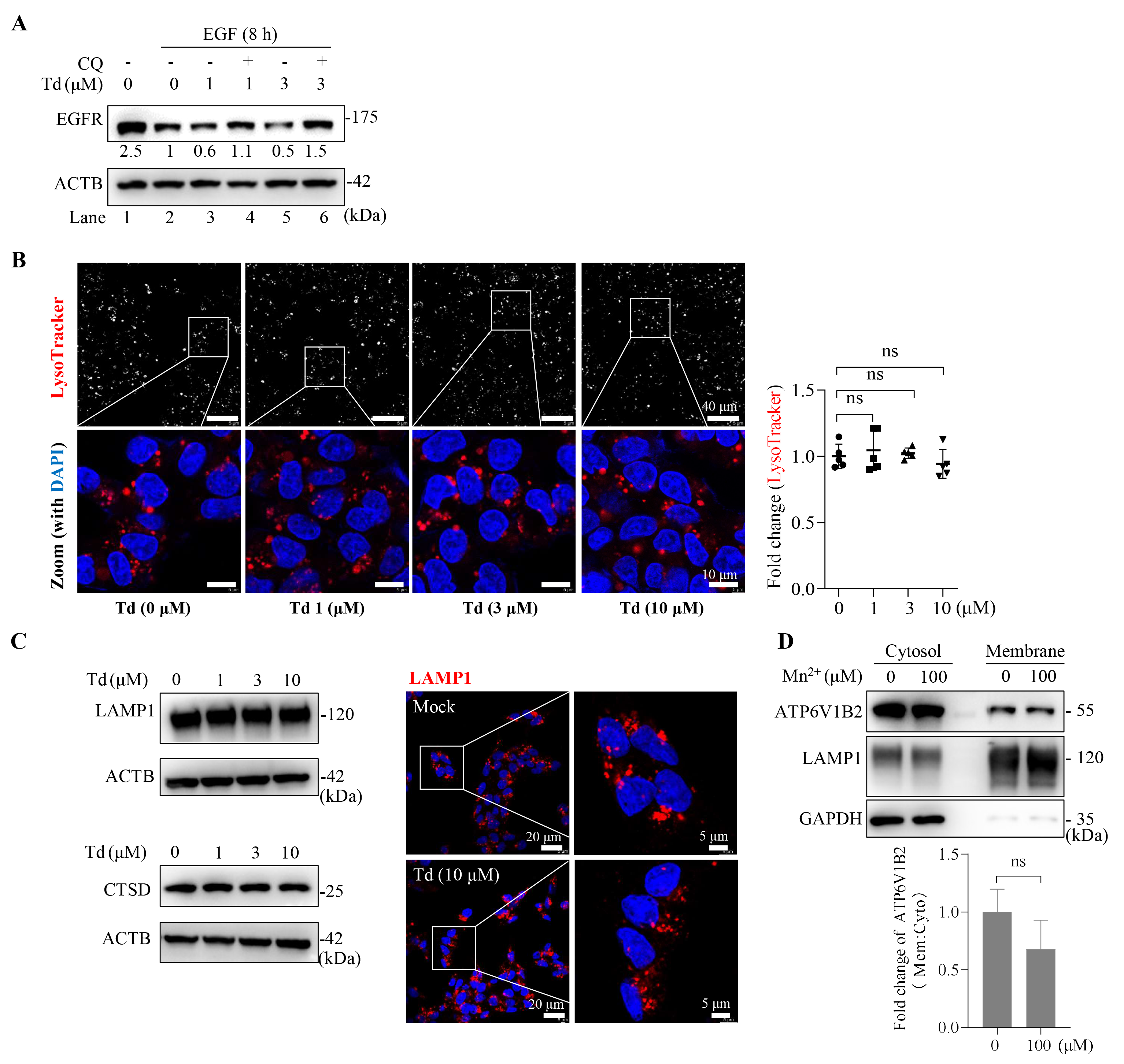

Supplement: S7 Fig — (A) Huh7 cells were stimulated with EGF (10 ng/mL) for 8 h in the presence of tomatidine (Td) at the indicated concentrations, with or without CQ (10 μM). The level of endogenous EGFR was determined by immunoblotting. The blots were analyzed by densitometry, with the intensity of the EGFR band normalized to the corresponding ACTB band. Representative data are shown from three independent experiments. (B) Representative LysoTracker staining of HepG2-NTCP cells treated with or without tomatidine (10 μM) for 12 h(left). The integrated density of five fields of view (40×) was calculated using ImageJ (right). (C) HepG2-NTCP cells were treated with or without tomatidine (10 μM) for 12 h. Left, endogenous LAMP1 and cleaved CTSD determined by immunoblotting. Right, subcellular distribution of LAMP1 assessed by immunofluorescence confocal microscopy. (D) HepG2-NTCP cells were fractionated into crude cytosolic and membrane fractions after 12 h of treatment with or without MnCl2 (100 μM), and then subjected to immunoblotting with the indicated antibodies. Lysosomal LAMP1 was used as a membrane fraction loading control and GAPDH as a cytosolic loading control. Error bars indicate the mean ± SD. ns, not significant. (TIF) [file ppat.1012800.s007.tif]

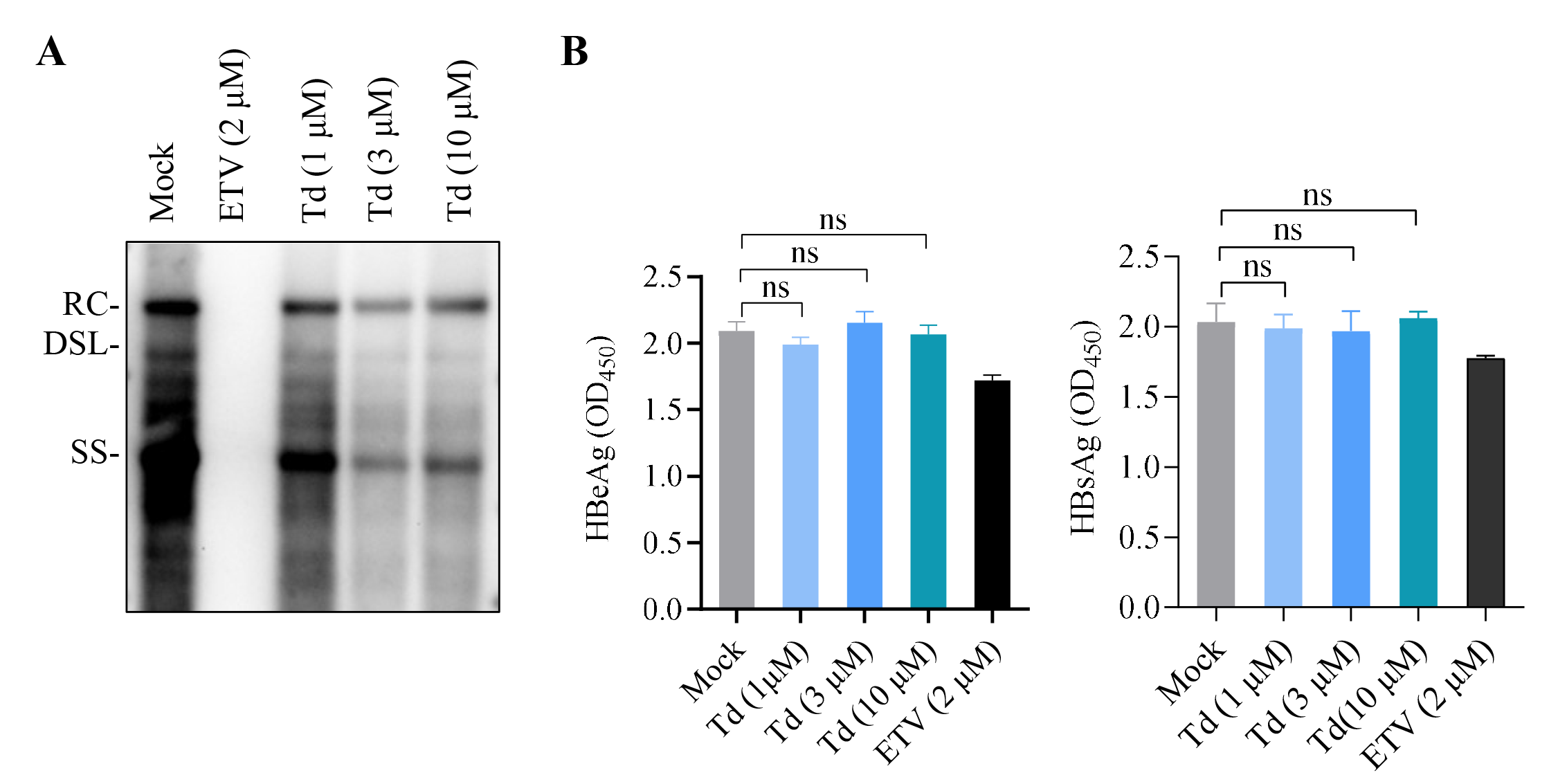

Supplement: S8 Fig — (A) HBV replication was induced in HepAD38 cells by the removal of doxycycline (tet-off) for 72 h. Cells were then treated with tomatidine (Td) at indicated concentrations for an additional 72 h, followed by Southern blotting for intracellular viral DNA species. RC, relaxed circular DNA; DSL, double-stranded linear DNA; SS, single-stranded linear DNA. Cells treated with 2 μM entecavir (ETV) were as a control. (B) Huh7 cells transiently transfected with pHBV1.3 were treated with different concentrations of tomatidine for 3 days. HBeAg and HBsAg in the supernatants were determined by ELISA (n = 3). Error bars indicate mean ± SD. ns, not significant. (TIF) [file ppat.1012800.s008.tif]

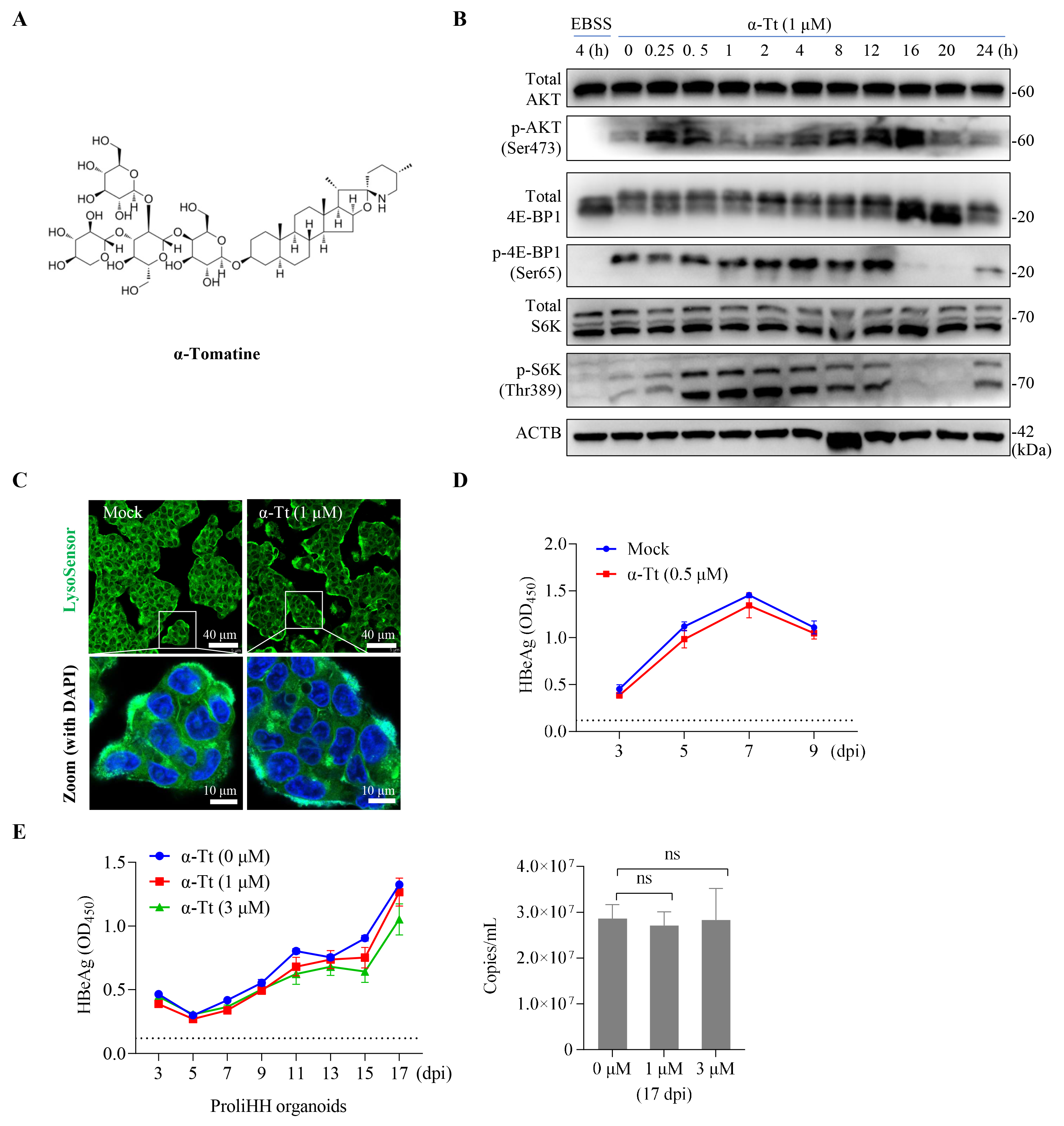

Supplement: S9 Fig — (A) Chemical structure of α-Tomatine (α-Tt). (B) HepG2-NTCP cells maintained in DMEM medium containing 10% FBS were stimulated with α-tomatine (1 μM) for various times, followed by immunoblotting with the indicated antibodies. Cells starved in EBSS medium (without amino acids) for 4 h were used as a control for mTORC1 inactivation. (C) Representative LysoSensor staining of HepG2-NTCP cells treated with or without α-tomatine (1 μM) for 12 h. (D) HepG2-NTCP cells were treated with or without α-tomatine (0.5 μM) for 10 h, followed by HBV infection for an additional 12 h. The supernatant HBeAg was determined by ELISA at the indicated time points (n = 3). Note that treatment with 1 μM α-tomatine slightly reduced the viability of HepG2-NTCP cells. (E) ProliHH organoids were mock-treated or treated with α-tomatine for 10 h, followed by HBV infection for an additional 12 h. ProliHHs were more resistant to α-tomatine-induced cytotoxicity than tumor cells at 1 μM and 3 μM. The secreted HBeAg and HBV DNA in the supernatants were determined by ELISA (left) and real-time PCR (right), respectively, at the indicated time points (n = 3). Error bars indicate mean ± SD. ns, not significant. (TIF) [file ppat.1012800.s009.tif]

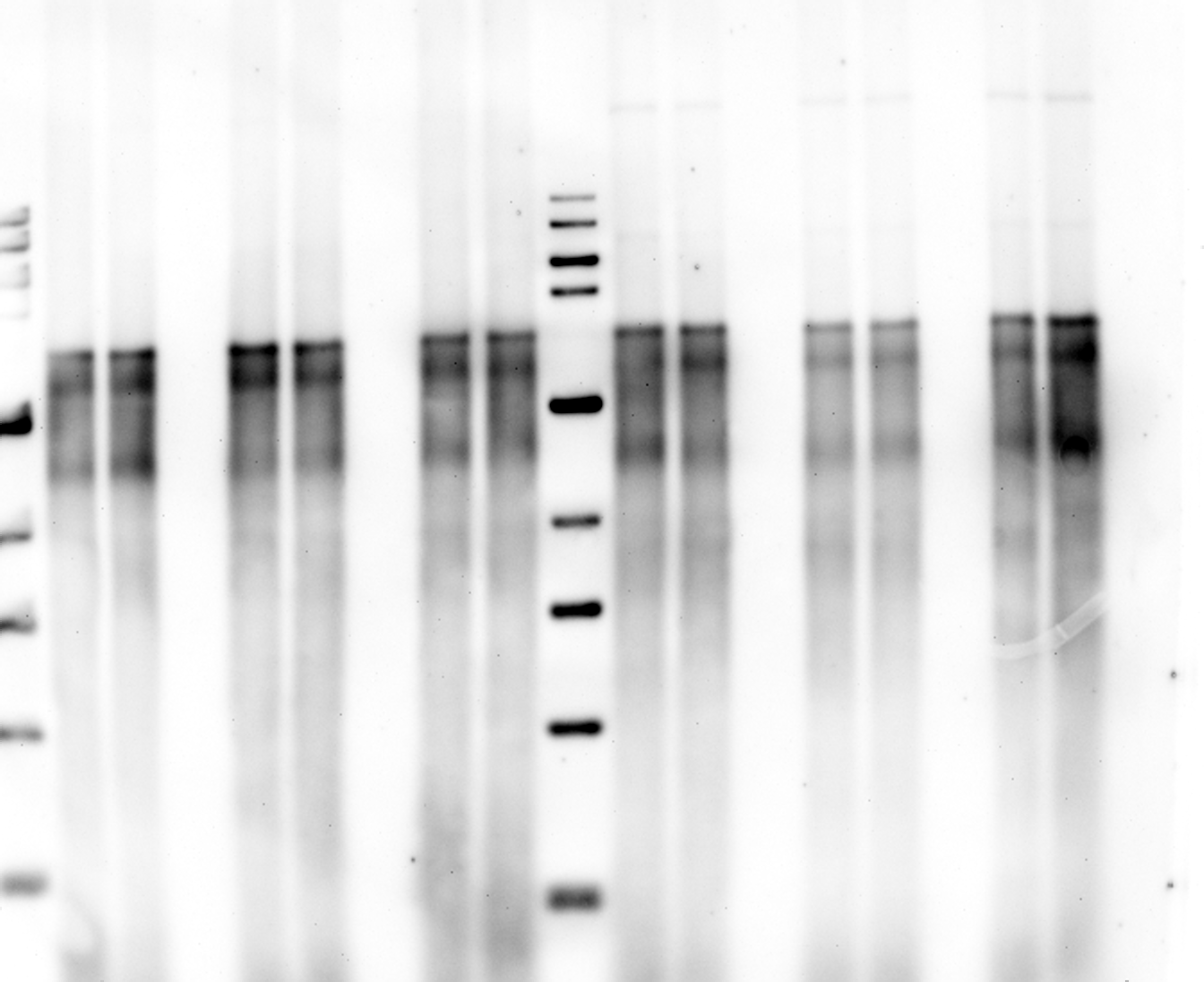

Supplement: S1 Dataset — (ZIP) [file ppat.1012800.s012.zip › Figs 1-9 minimal data set/fig 1/fig 1D.Tif]

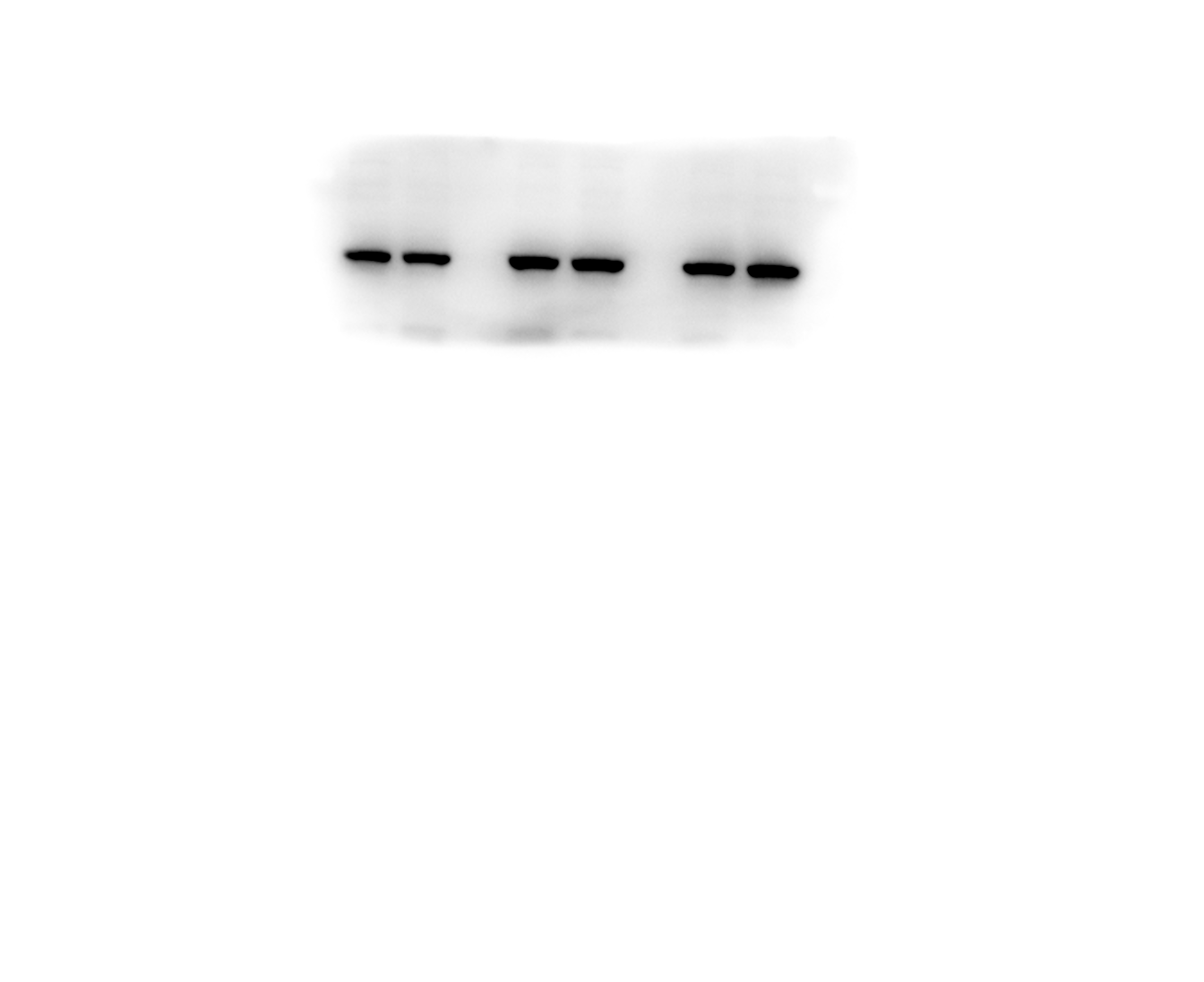

Supplement: S1 Dataset — (ZIP) [file ppat.1012800.s012.zip › Figs 1-9 minimal data set/fig 1/fig 1E/ACTB.Tif]

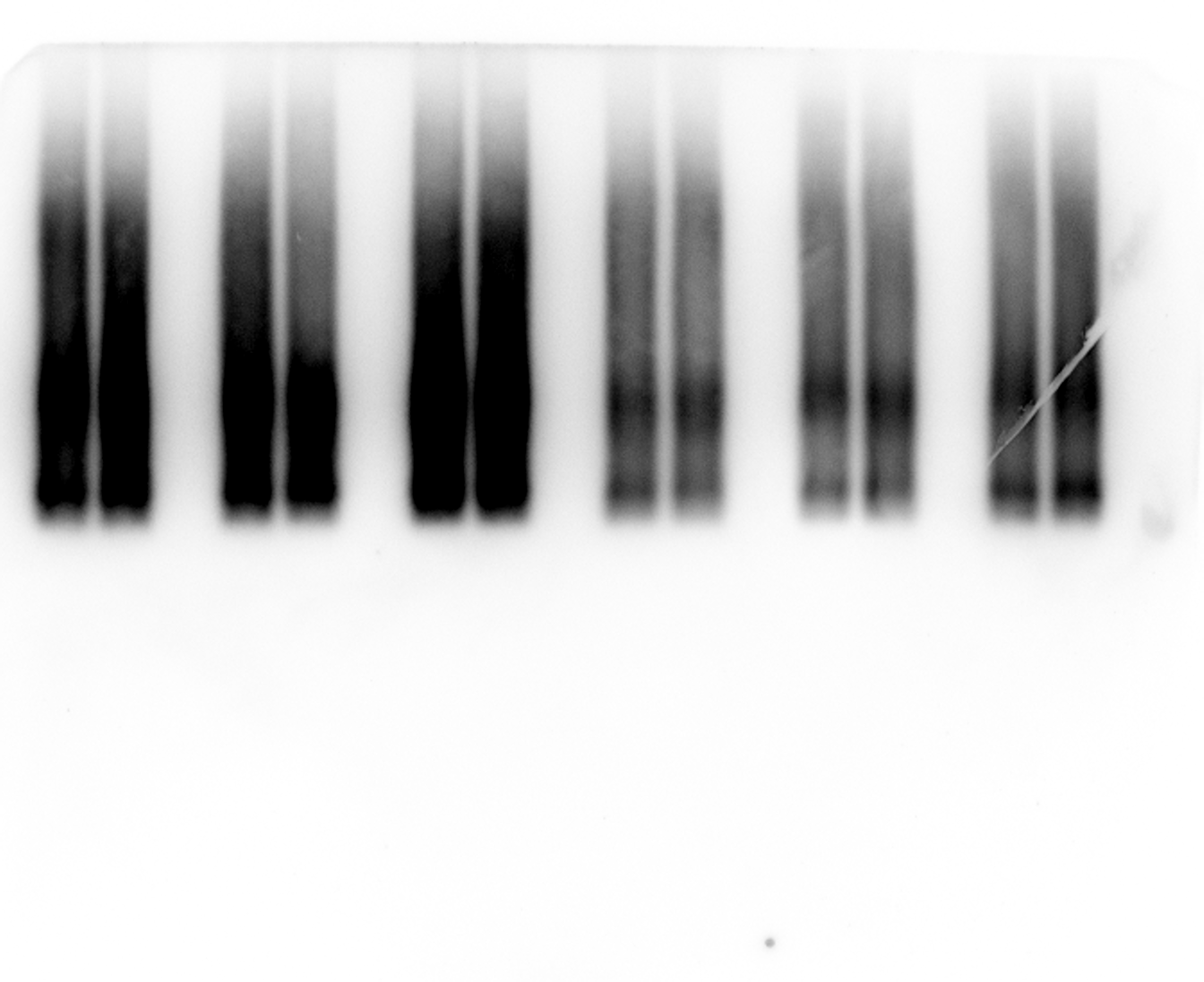

Supplement: S1 Dataset — (ZIP) [file ppat.1012800.s012.zip › Figs 1-9 minimal data set/fig 1/fig 1E/Capsid.Tif]

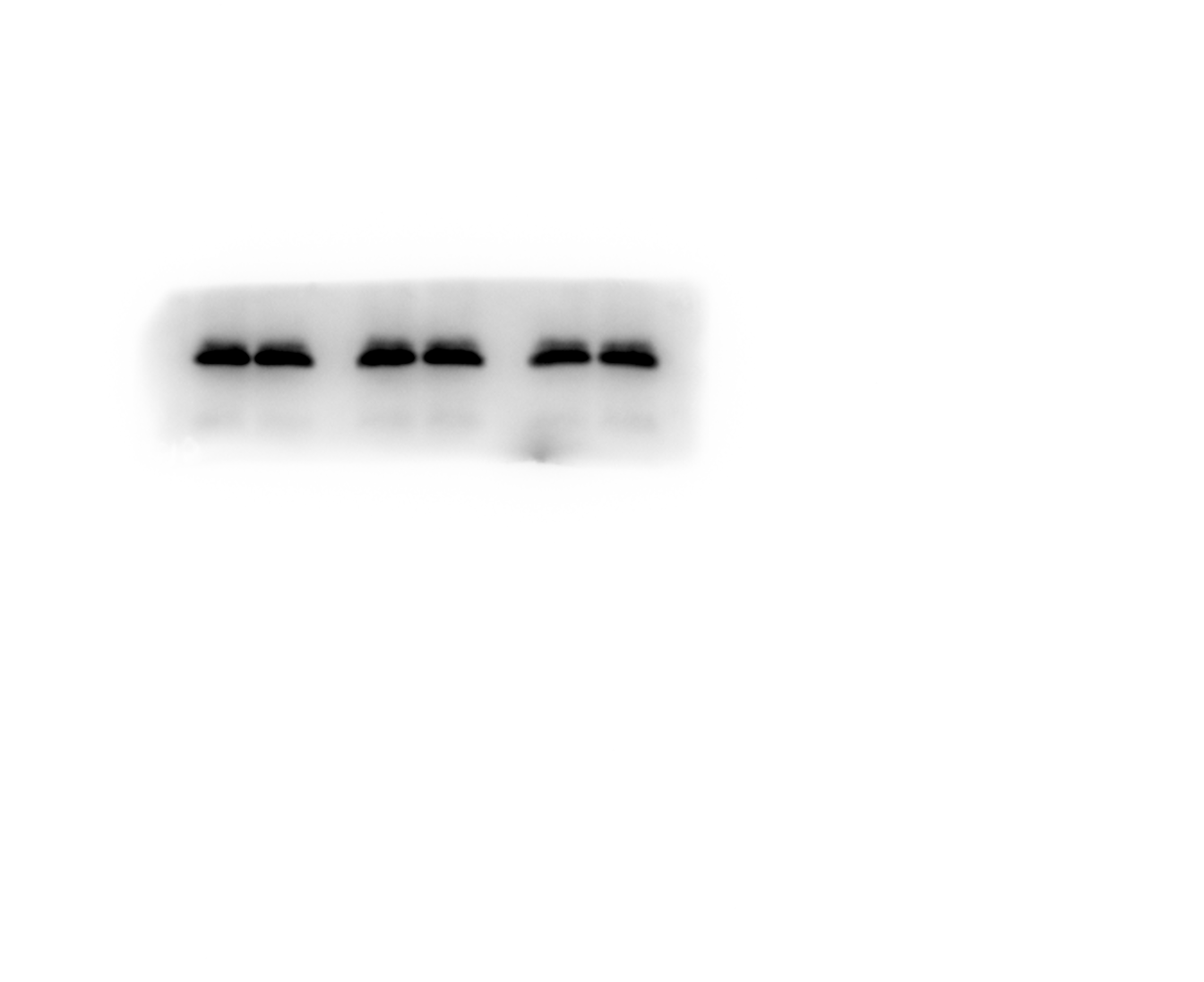

Supplement: S1 Dataset — (ZIP) [file ppat.1012800.s012.zip › Figs 1-9 minimal data set/fig 1/fig 1E/HBc.Tif]

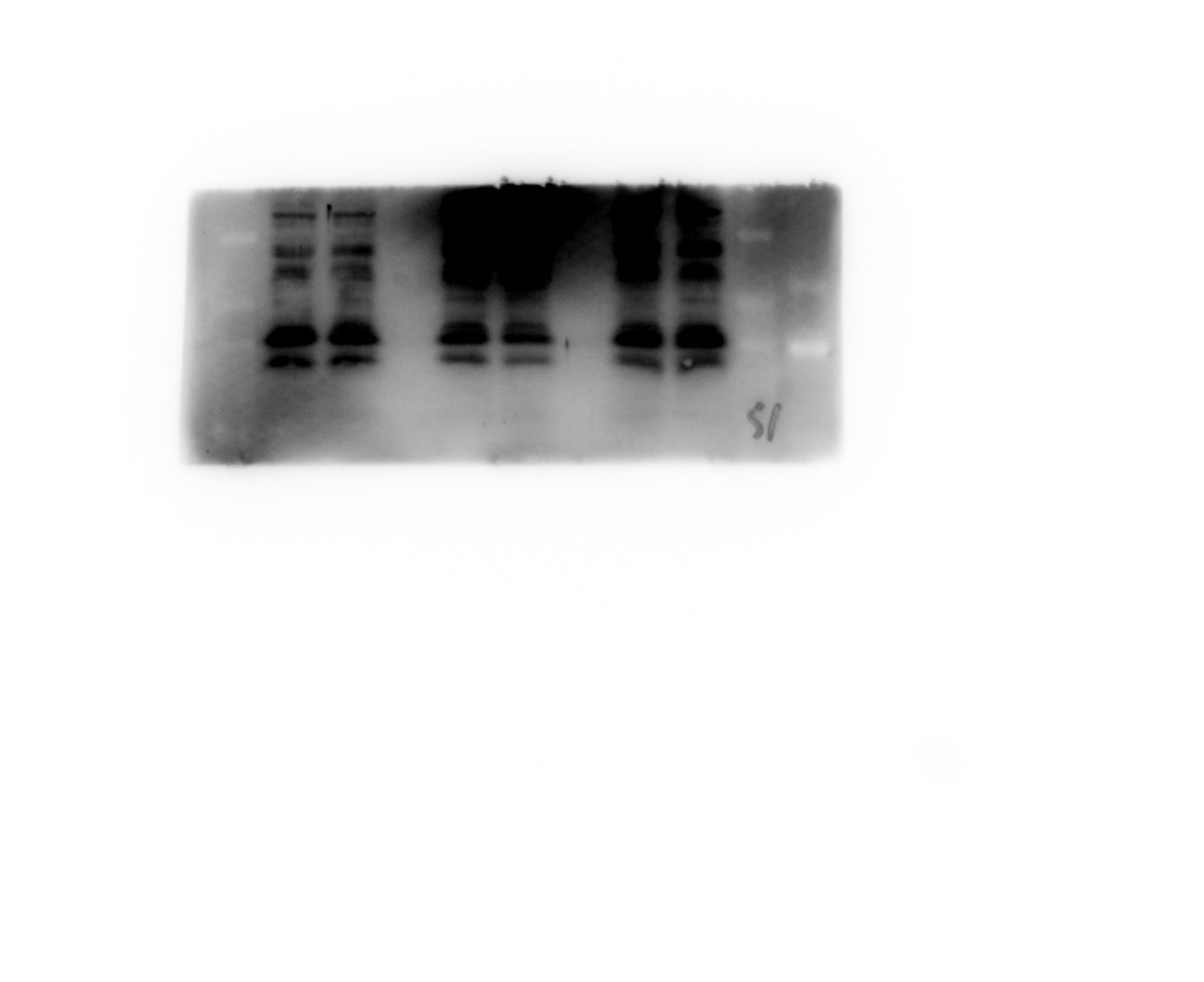

Supplement: S1 Dataset — (ZIP) [file ppat.1012800.s012.zip › Figs 1-9 minimal data set/fig 1/fig 1E/HBs.Tif]

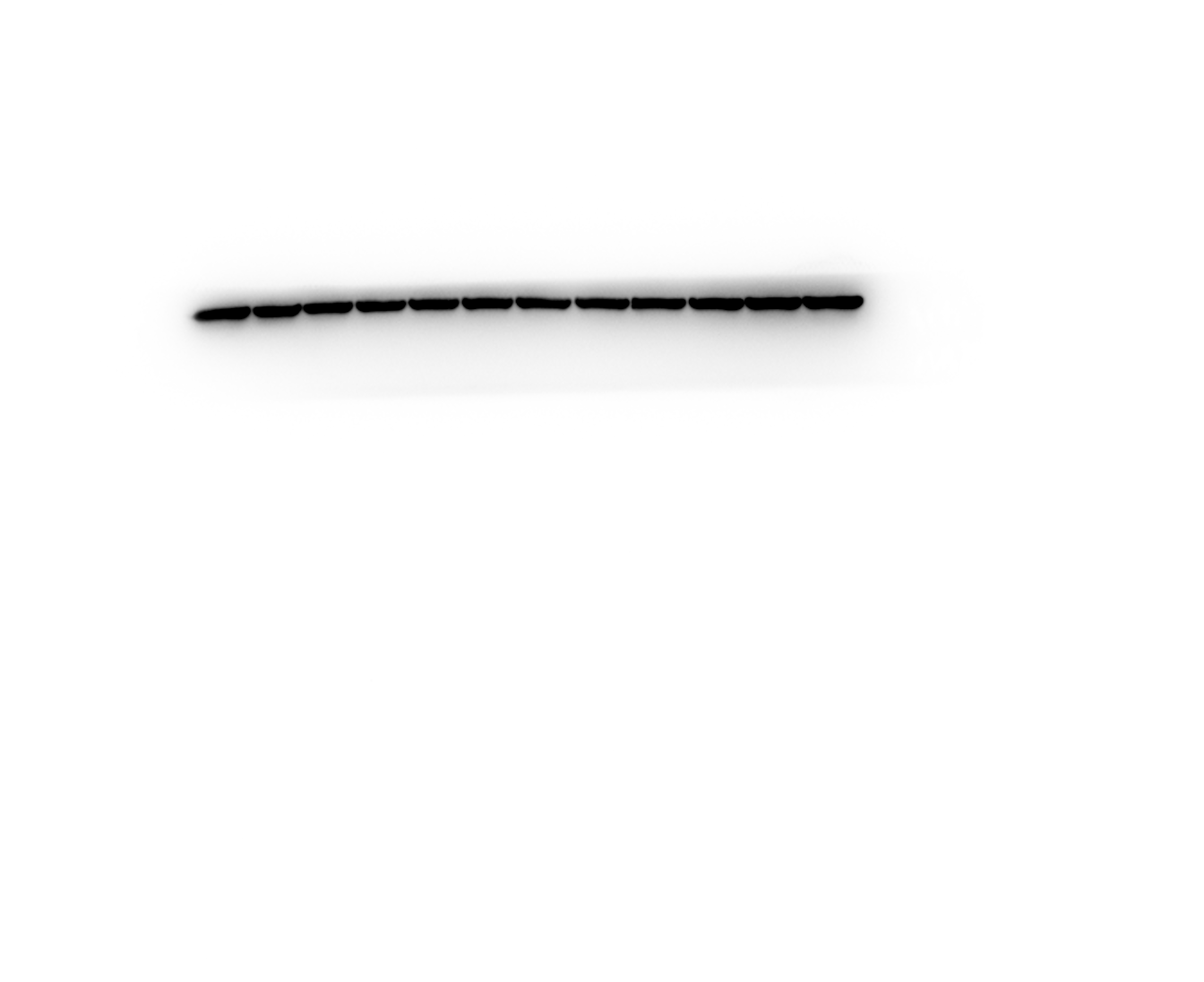

Supplement: S1 Dataset — (ZIP) [file ppat.1012800.s012.zip › Figs 1-9 minimal data set/fig 2/fig 2C/ACTB for AKT.Tif]

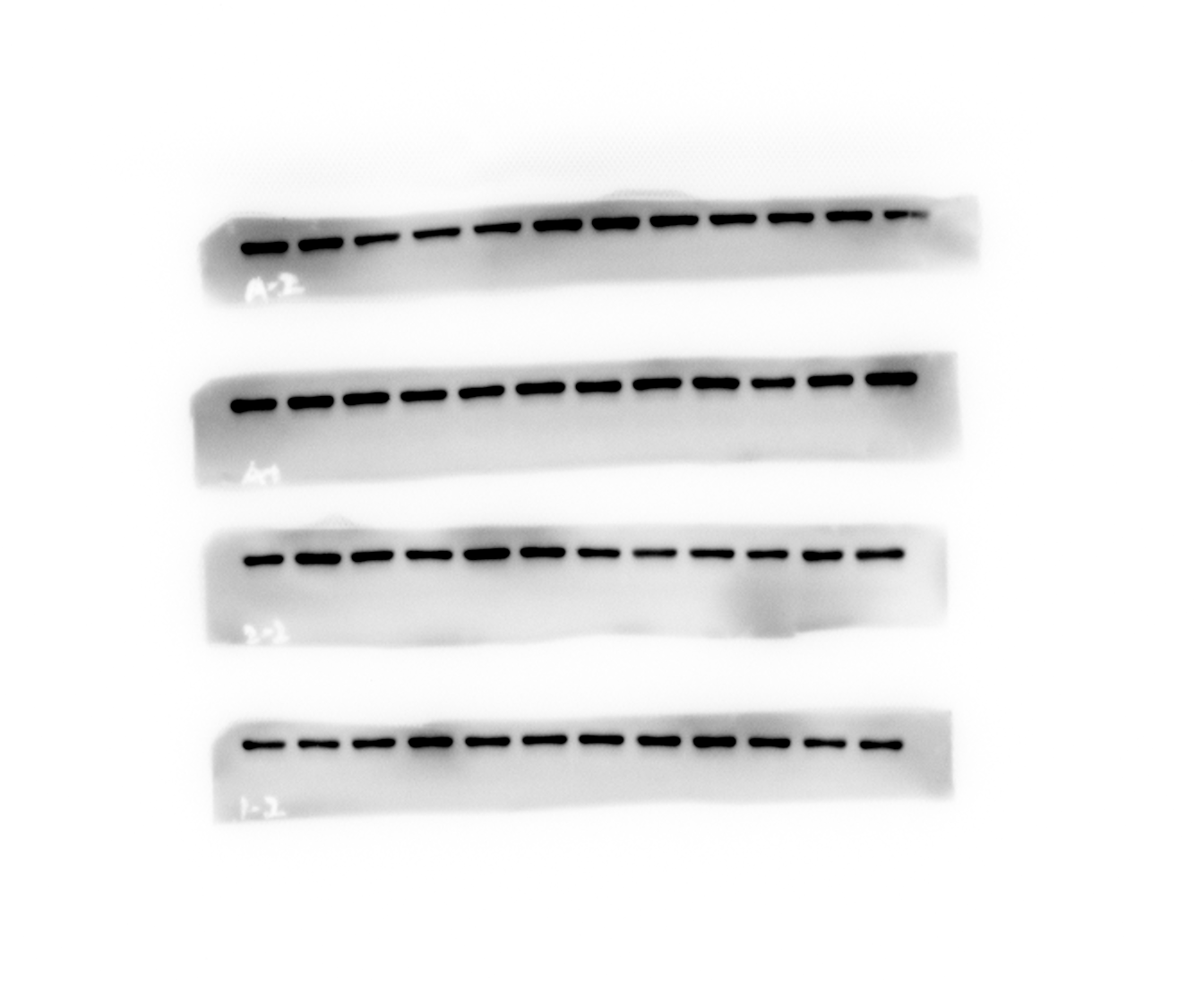

Supplement: S1 Dataset — (ZIP) [file ppat.1012800.s012.zip › Figs 1-9 minimal data set/fig 2/fig 2C/ACTB for upper panel, the second one.Tif]

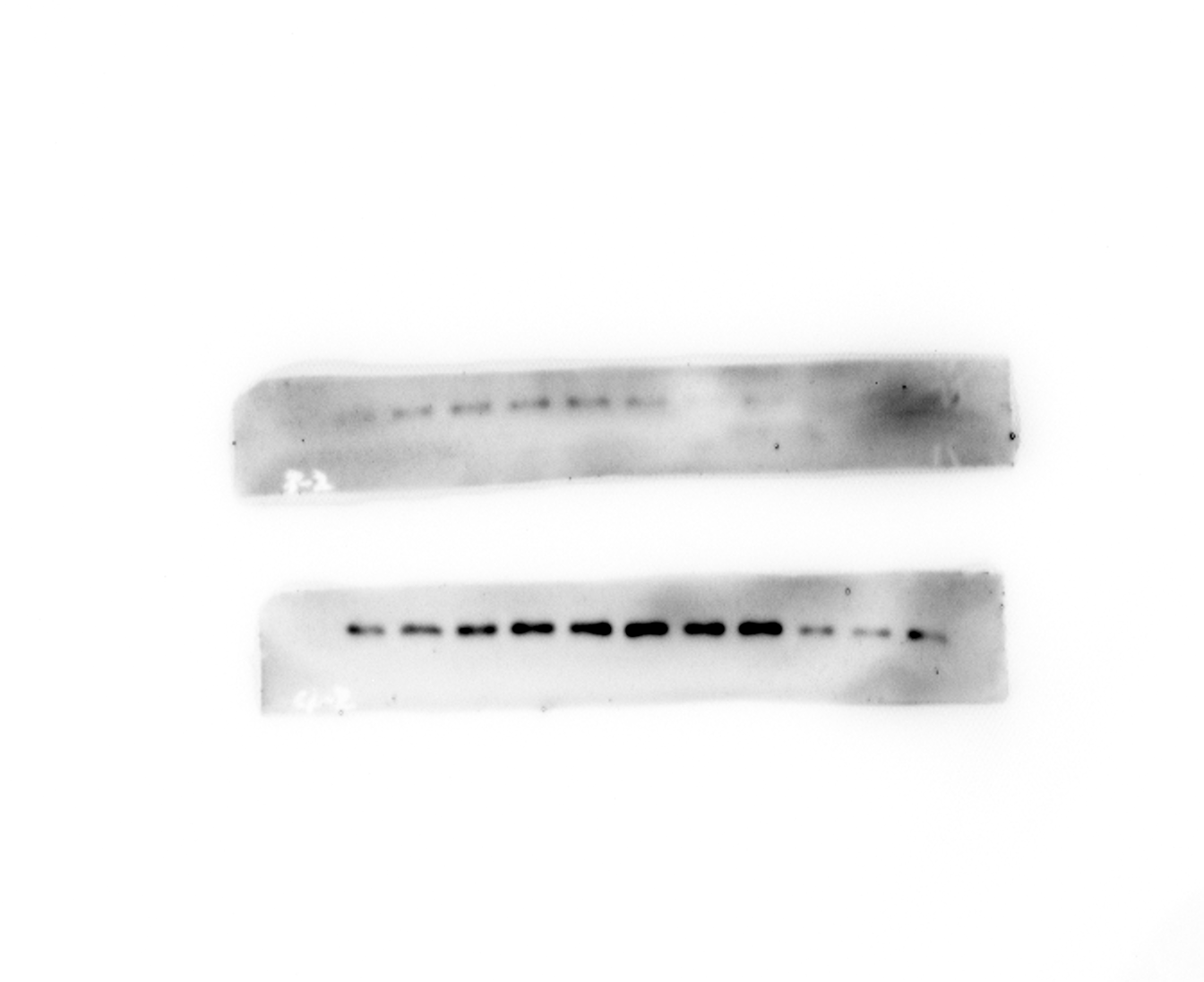

Supplement: S1 Dataset — (ZIP) [file ppat.1012800.s012.zip › Figs 1-9 minimal data set/fig 2/fig 2C/P-4E-BP1,lower.Tif]

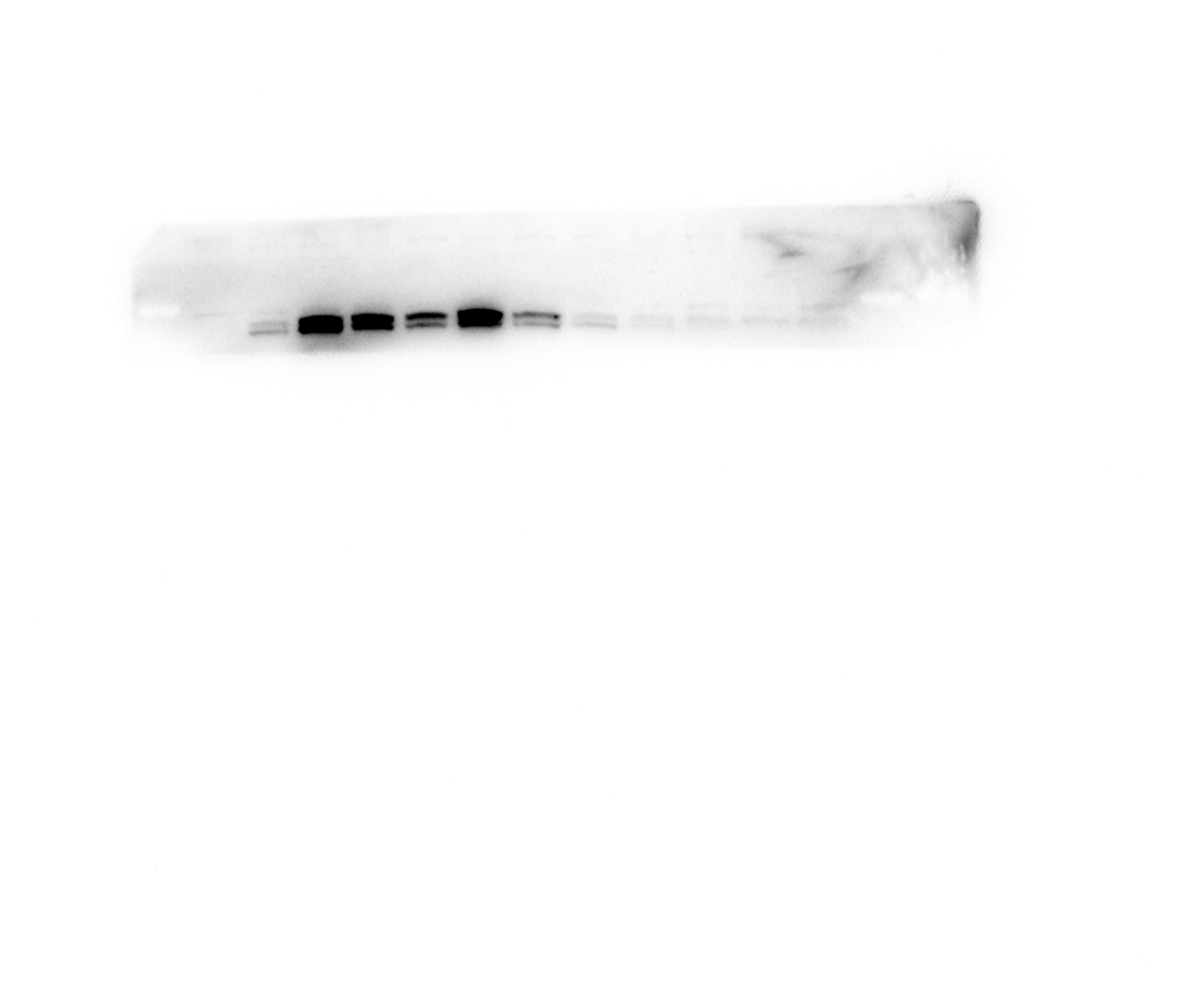

Supplement: S1 Dataset — (ZIP) [file ppat.1012800.s012.zip › Figs 1-9 minimal data set/fig 2/fig 2C/P-AKT.Tif]

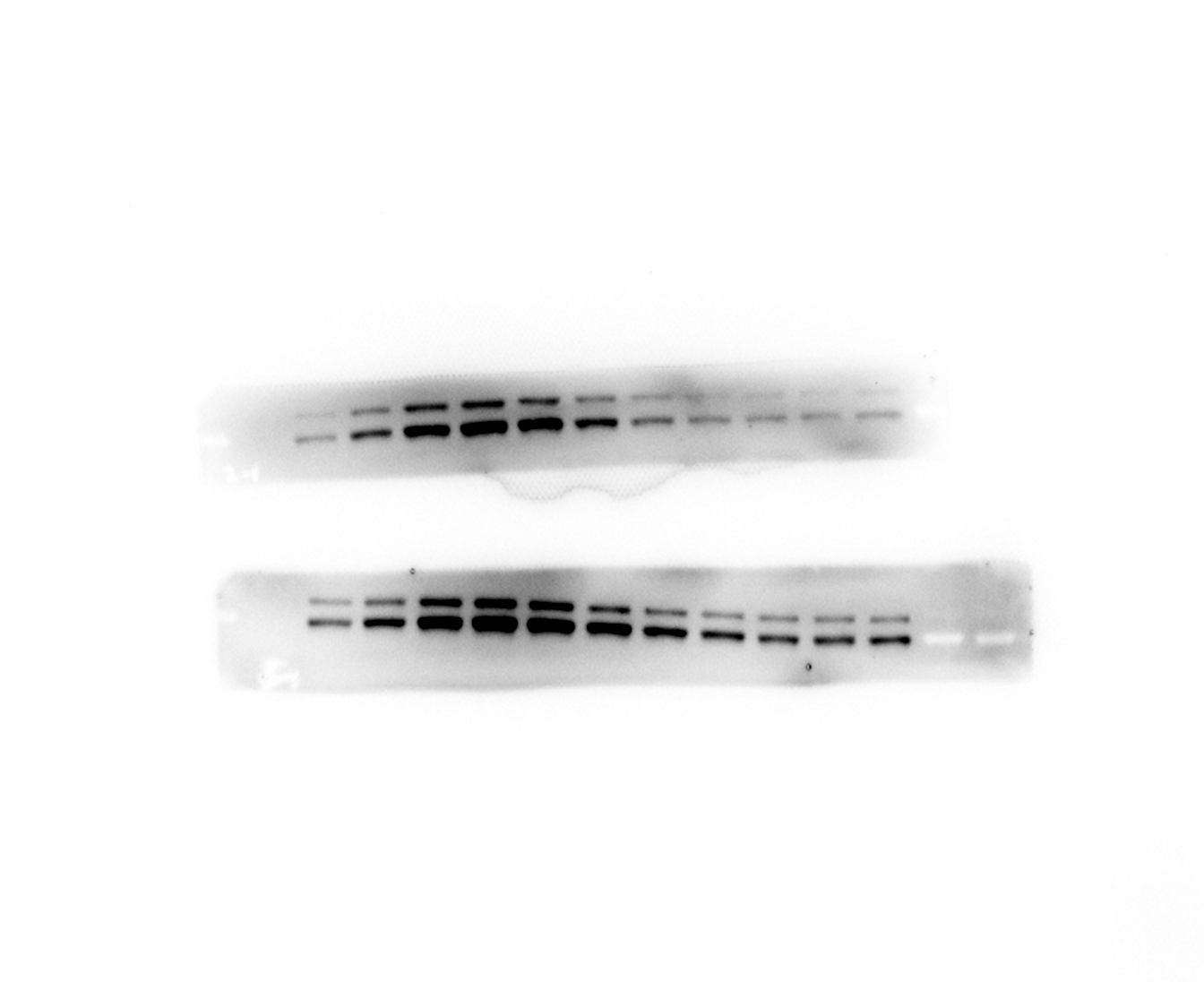

Supplement: S1 Dataset — (ZIP) [file ppat.1012800.s012.zip › Figs 1-9 minimal data set/fig 2/fig 2C/P-S6K, lower.Tif]

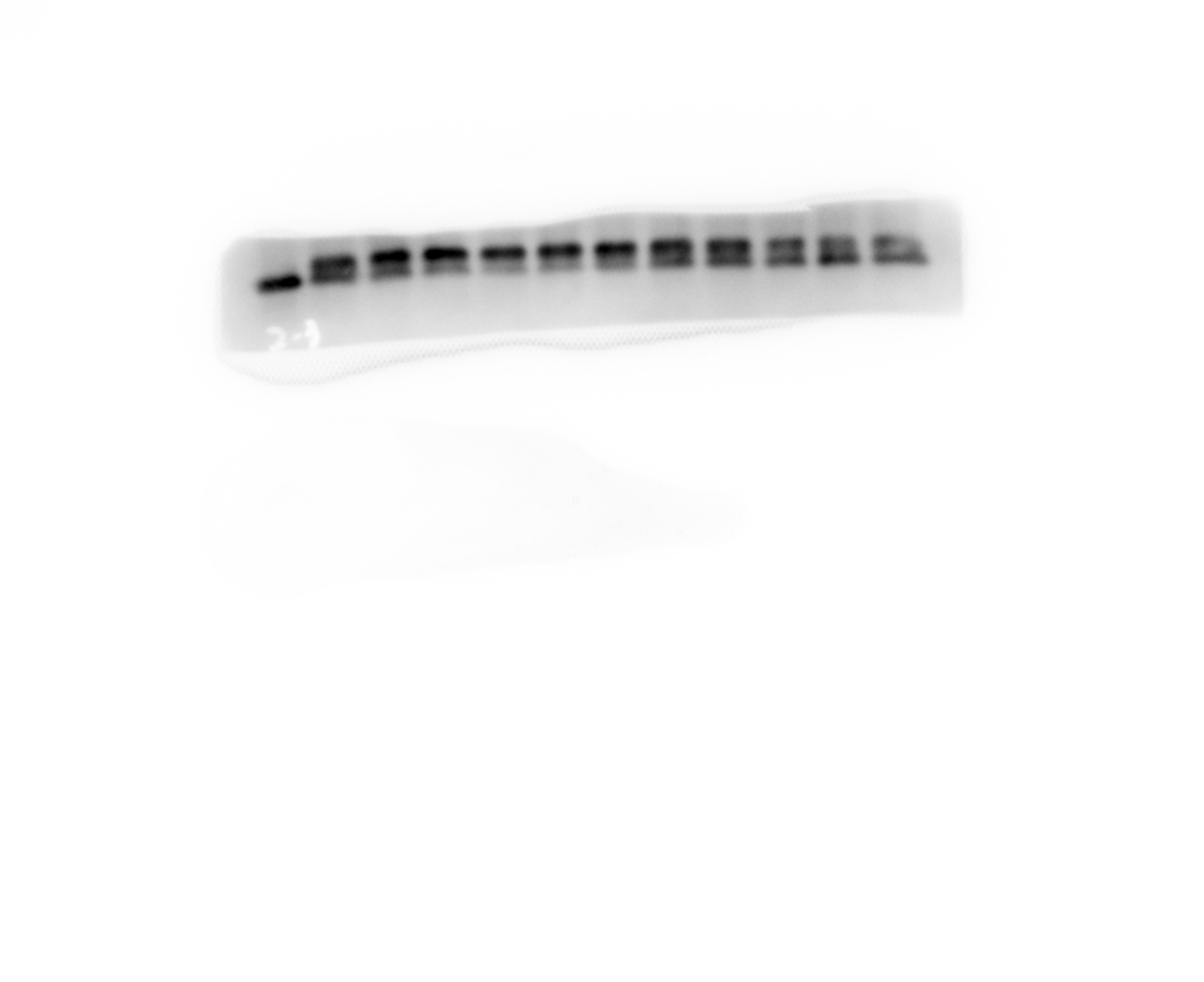

Supplement: S1 Dataset — (ZIP) [file ppat.1012800.s012.zip › Figs 1-9 minimal data set/fig 2/fig 2C/total 4E-BP1.Tif]

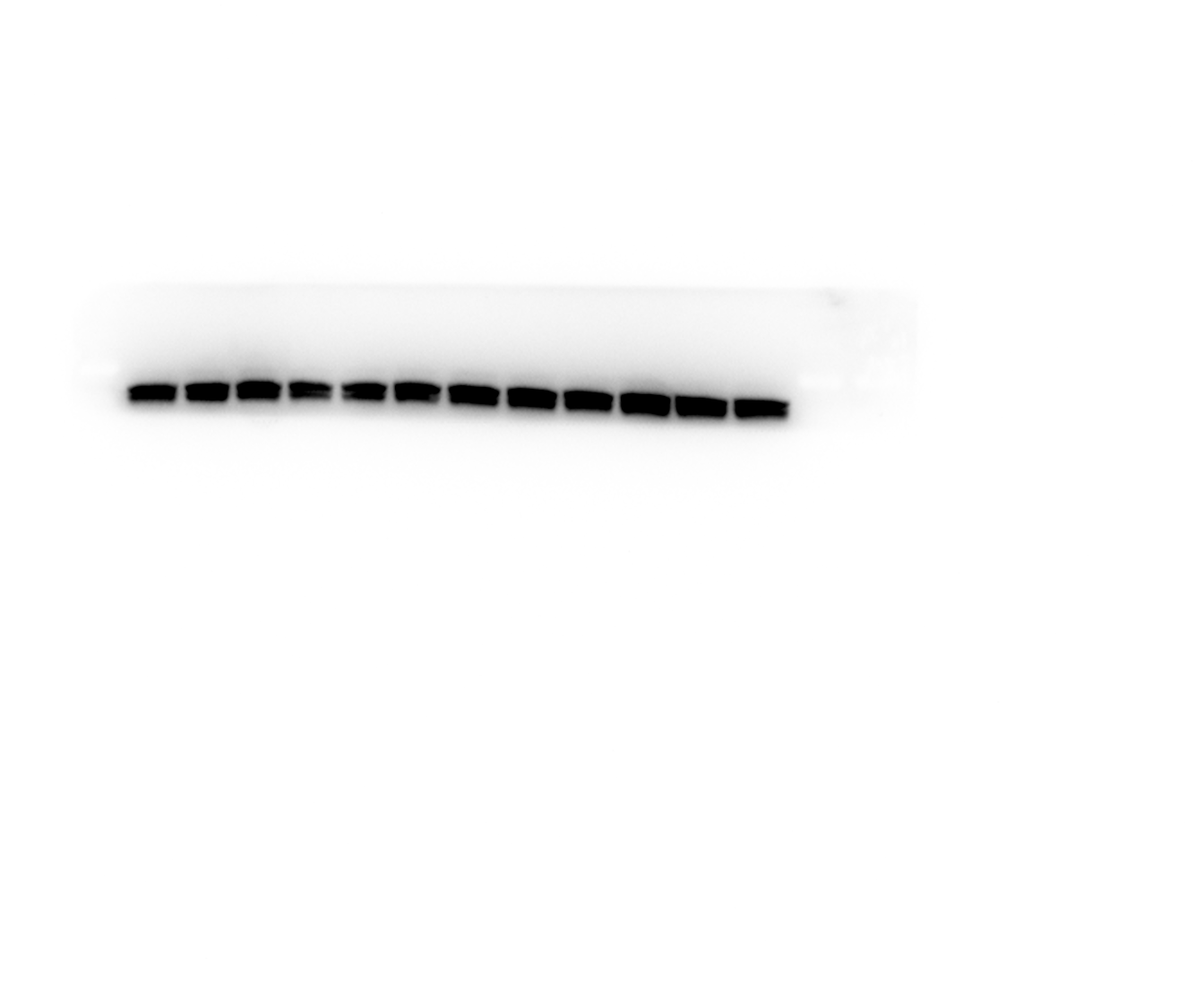

Supplement: S1 Dataset — (ZIP) [file ppat.1012800.s012.zip › Figs 1-9 minimal data set/fig 2/fig 2C/total AKT.Tif]

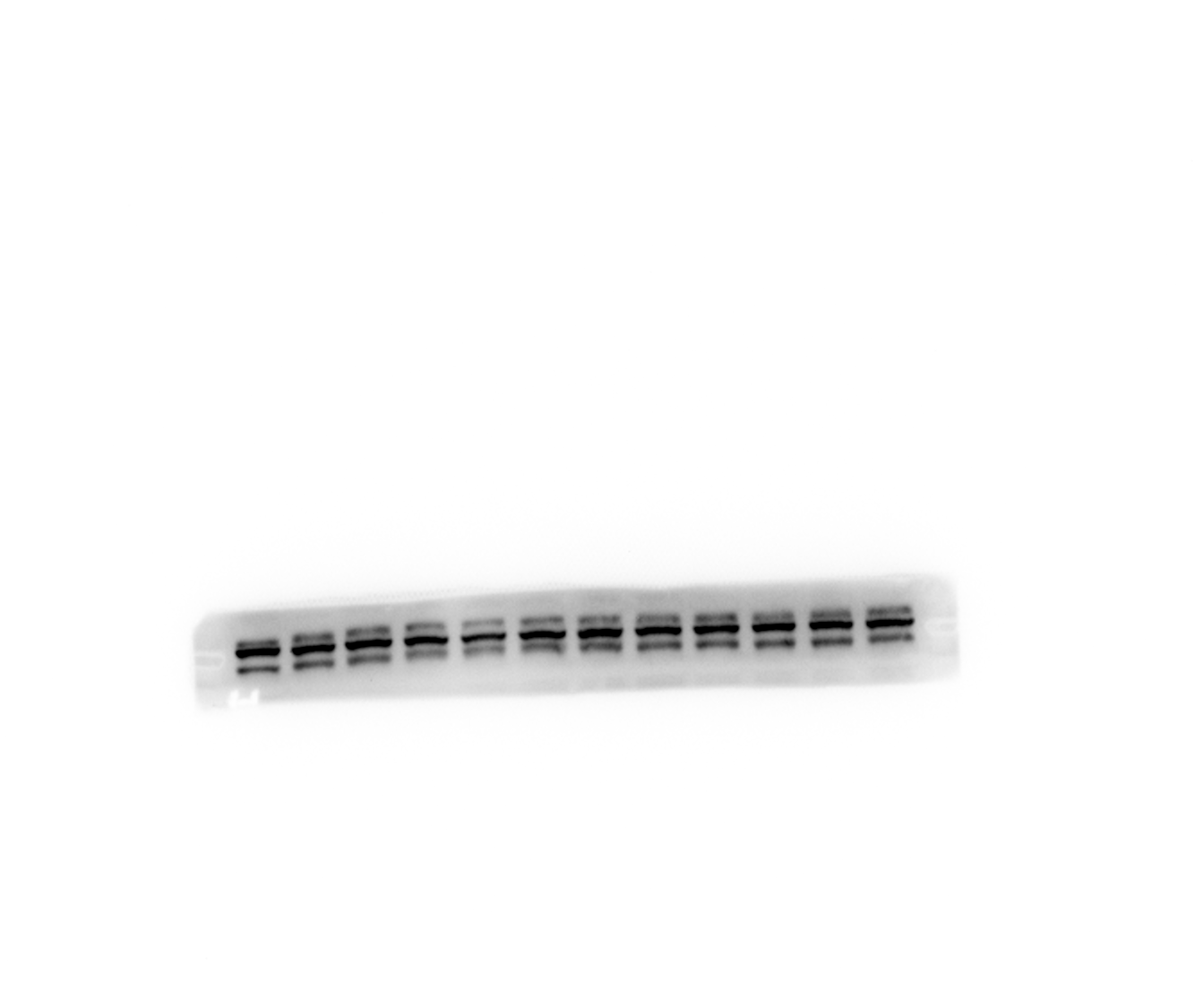

Supplement: S1 Dataset — (ZIP) [file ppat.1012800.s012.zip › Figs 1-9 minimal data set/fig 2/fig 2C/total S6K.Tif]

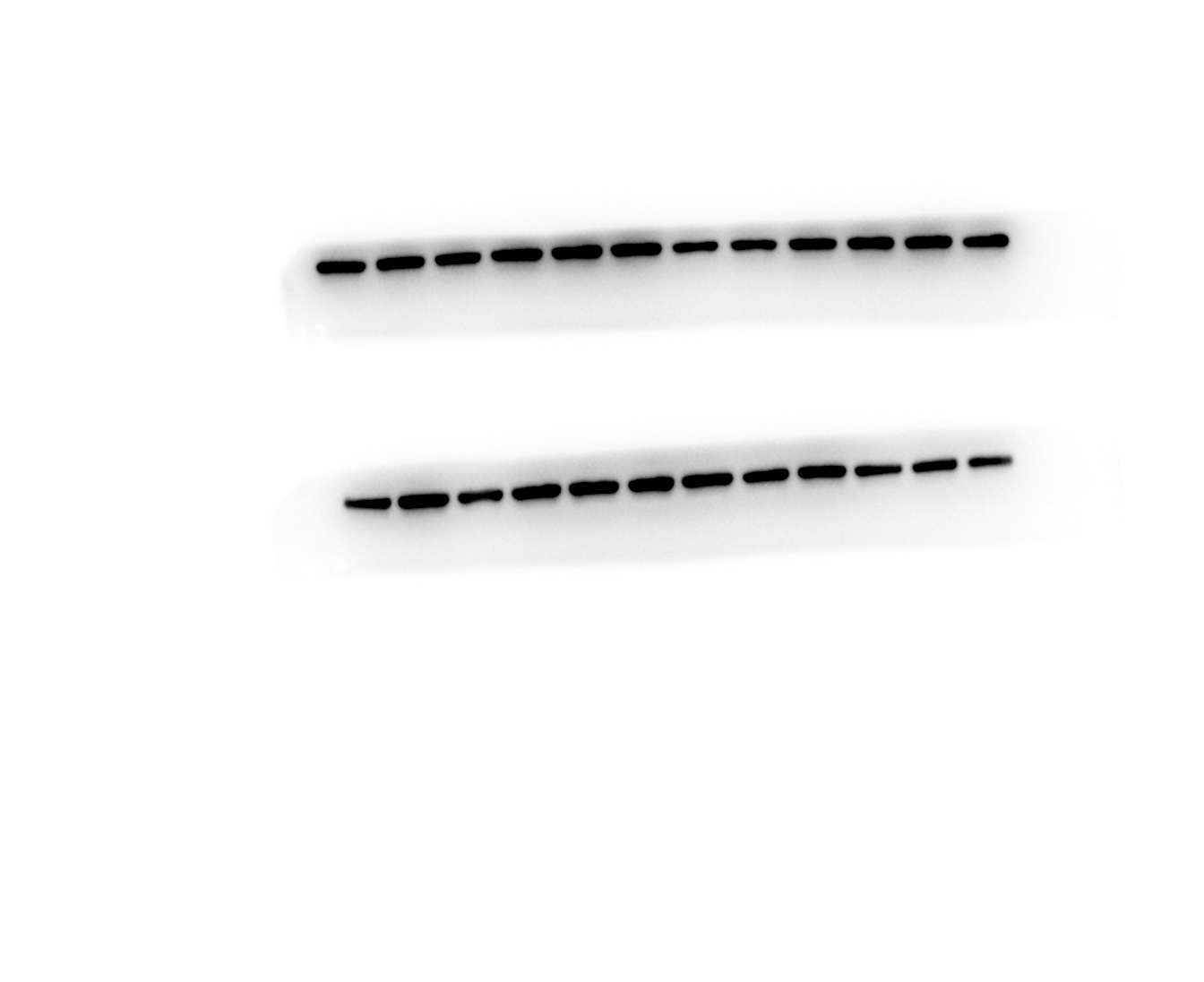

Supplement: S1 Dataset — (ZIP) [file ppat.1012800.s012.zip › Figs 1-9 minimal data set/fig 2/fig 2D/ACTB, upper.Tif]

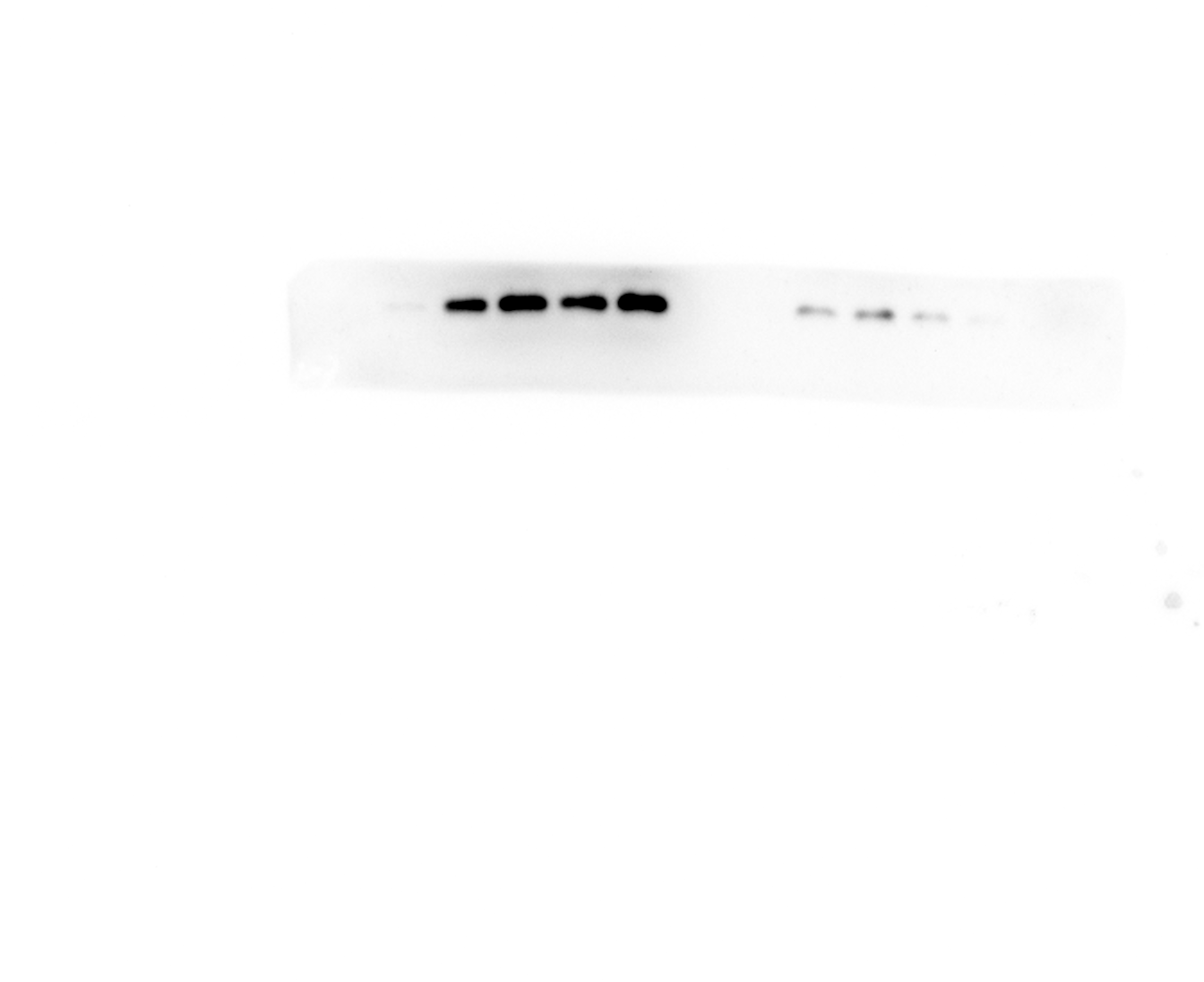

Supplement: S1 Dataset — (ZIP) [file ppat.1012800.s012.zip › Figs 1-9 minimal data set/fig 2/fig 2D/P-4EBP1.Tif]

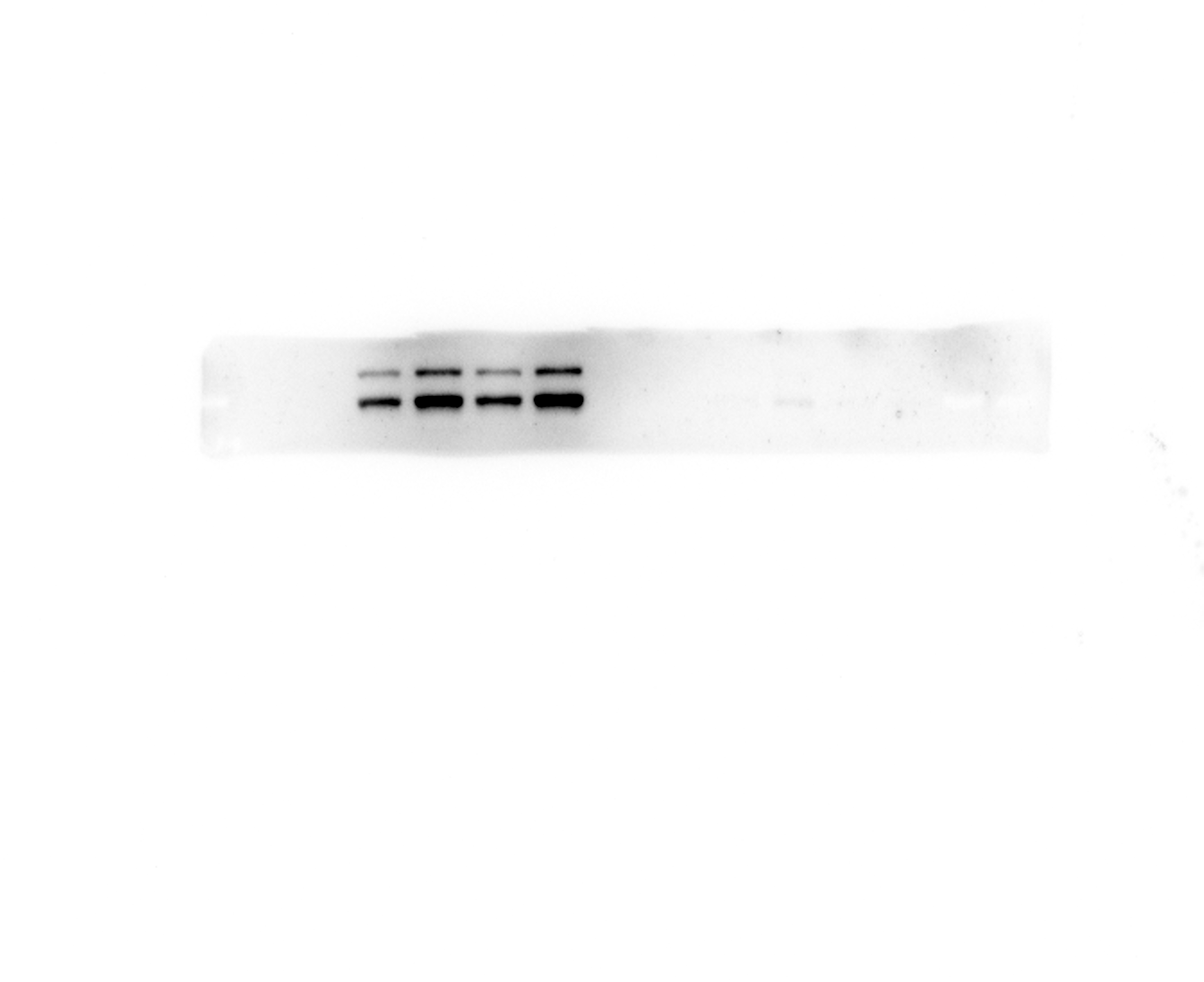

Supplement: S1 Dataset — (ZIP) [file ppat.1012800.s012.zip › Figs 1-9 minimal data set/fig 2/fig 2D/P-S6K.Tif]

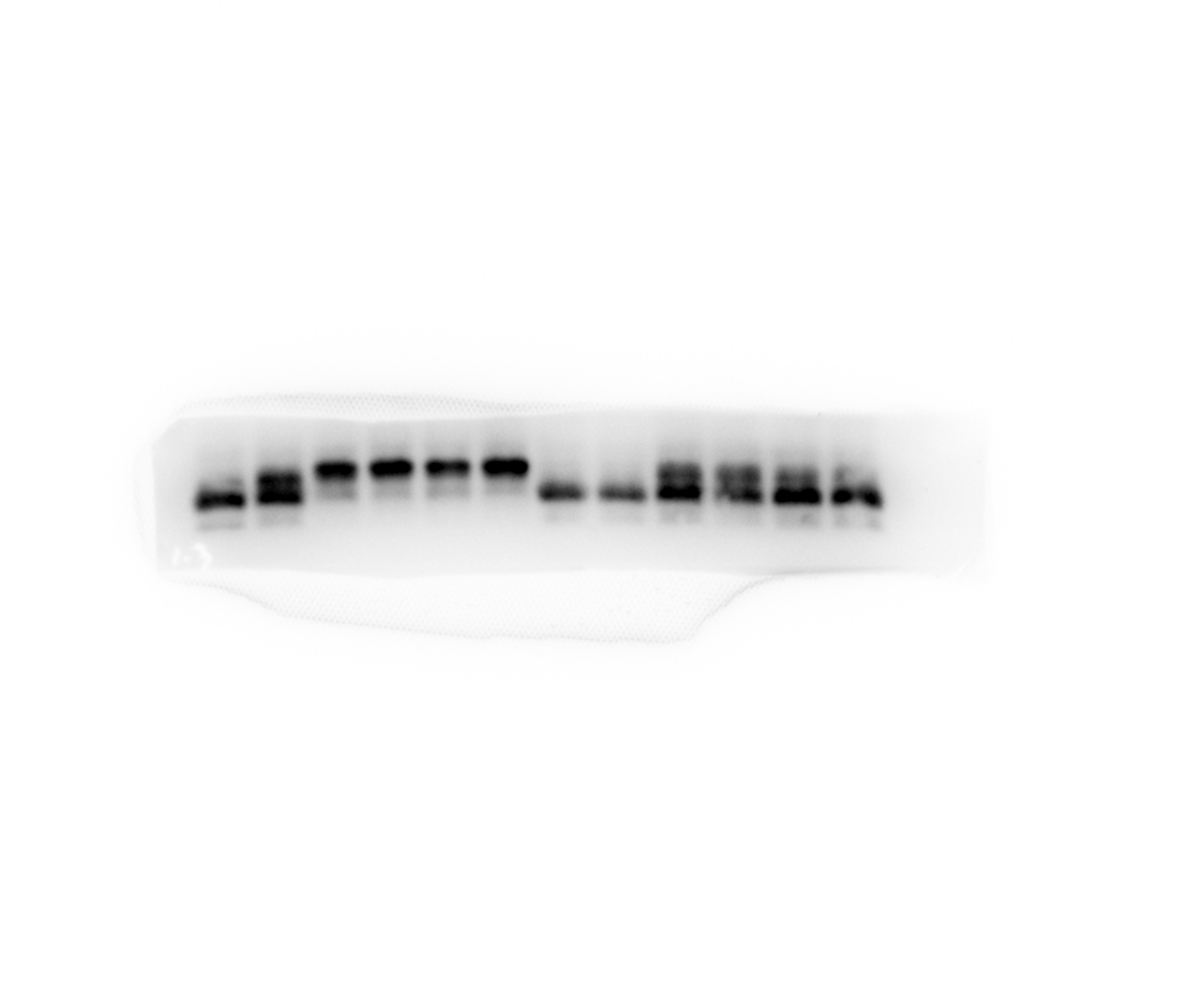

Supplement: S1 Dataset — (ZIP) [file ppat.1012800.s012.zip › Figs 1-9 minimal data set/fig 2/fig 2D/total 4E-BP1.Tif]

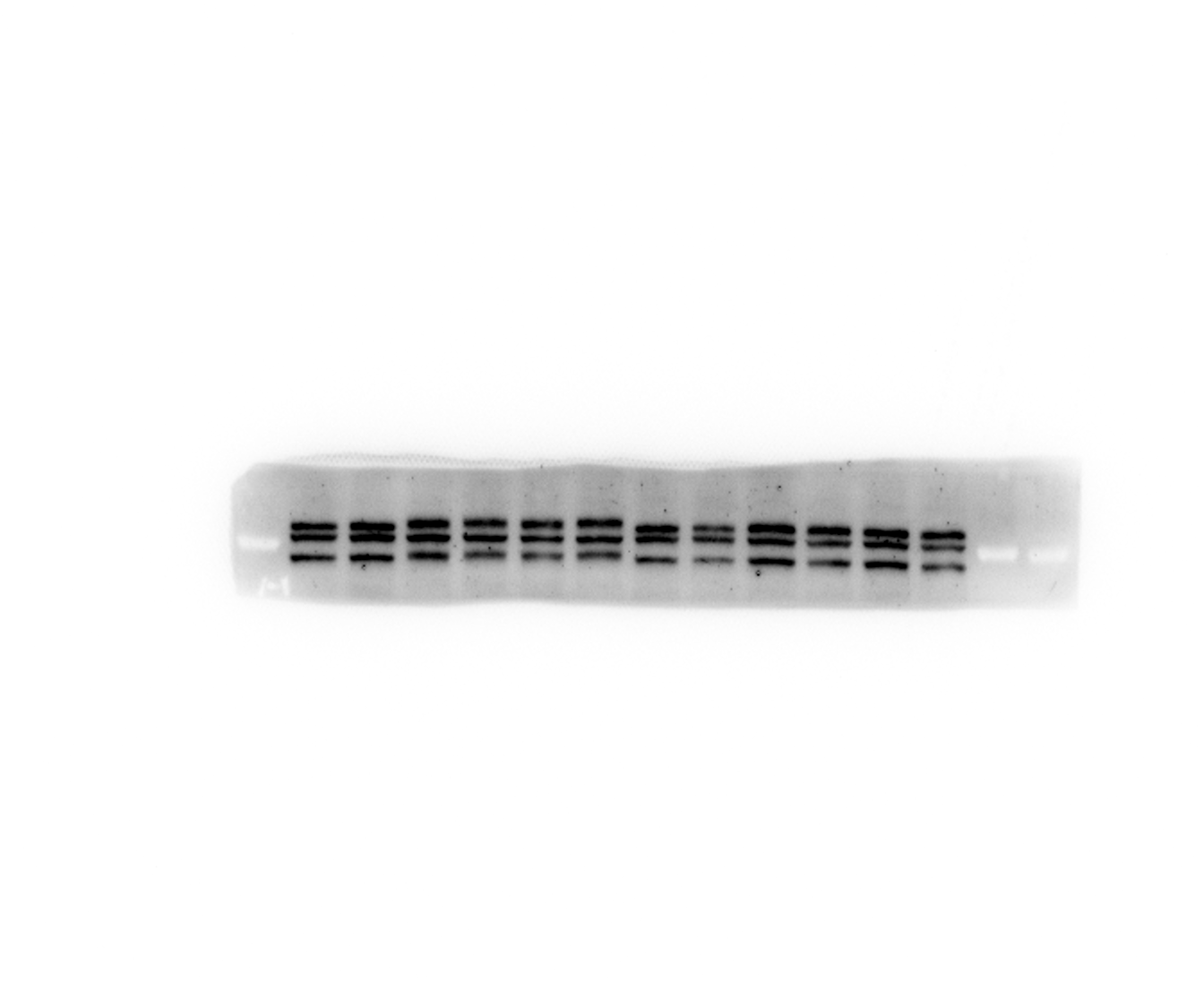

Supplement: S1 Dataset — (ZIP) [file ppat.1012800.s012.zip › Figs 1-9 minimal data set/fig 2/fig 2D/total S6K.Tif]

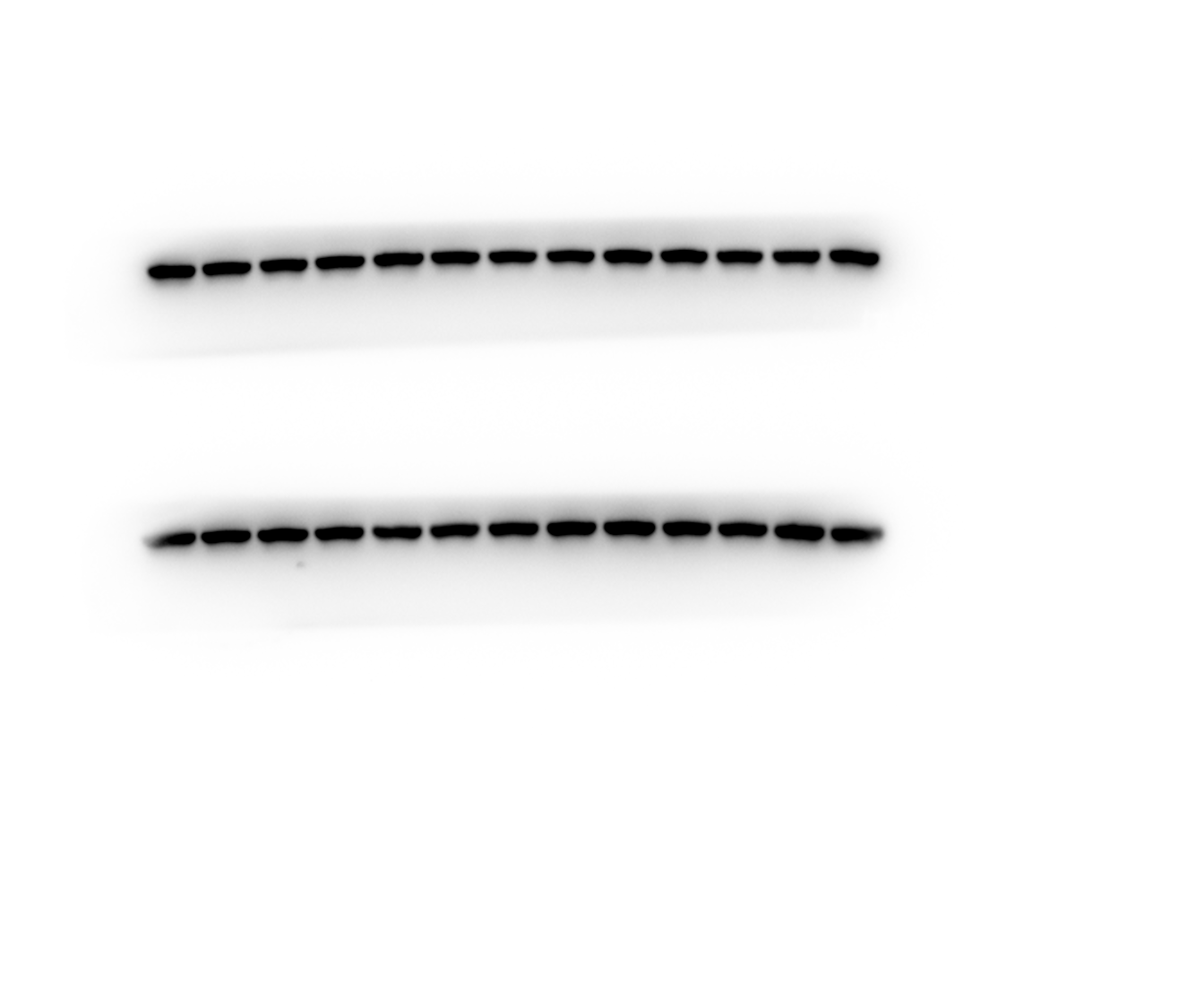

Supplement: S1 Dataset — (ZIP) [file ppat.1012800.s012.zip › Figs 1-9 minimal data set/fig 2/fig 2E/ACTB, upper.Tif]

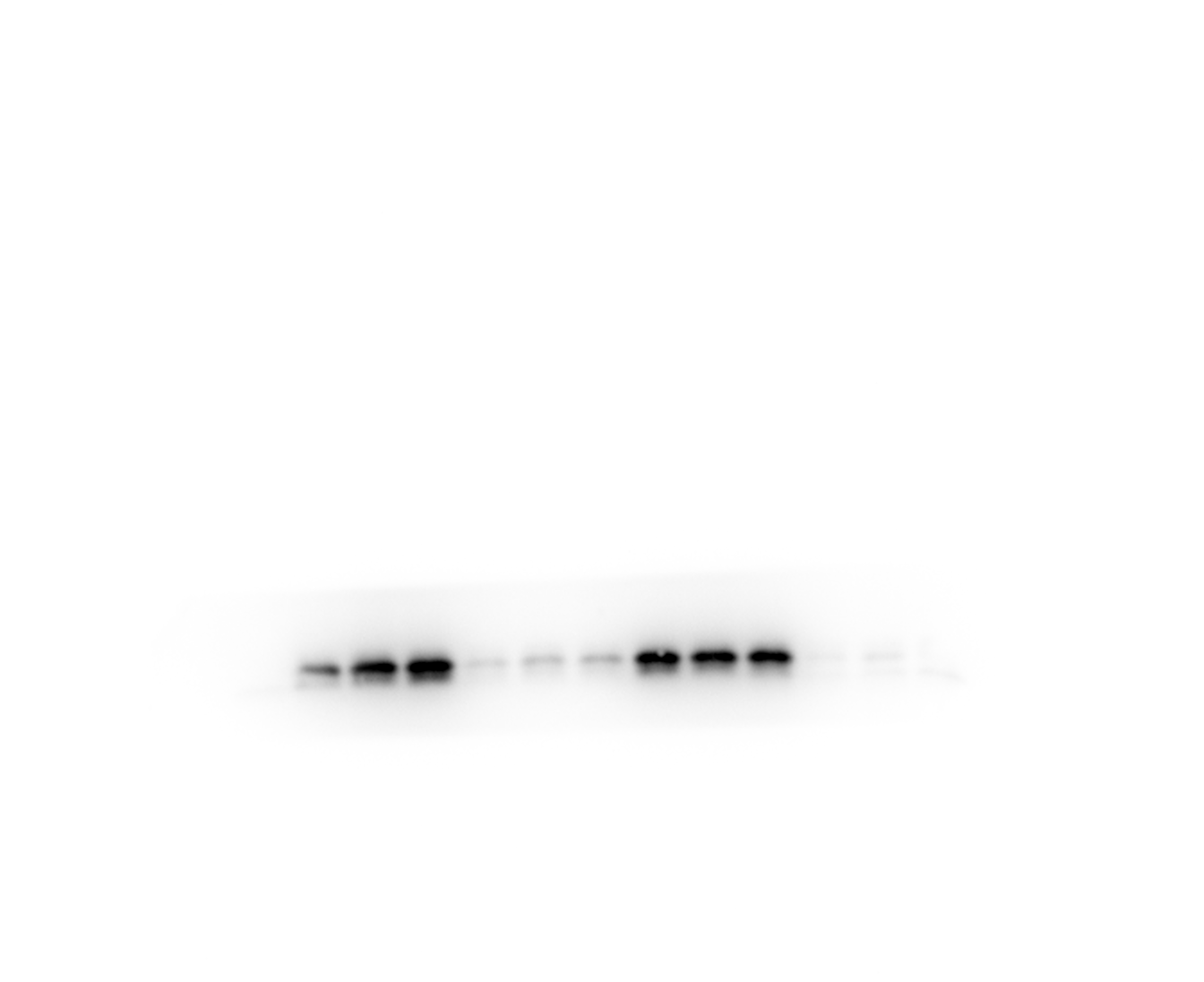

Supplement: S1 Dataset — (ZIP) [file ppat.1012800.s012.zip › Figs 1-9 minimal data set/fig 2/fig 2E/P-4EBP1.Tif]

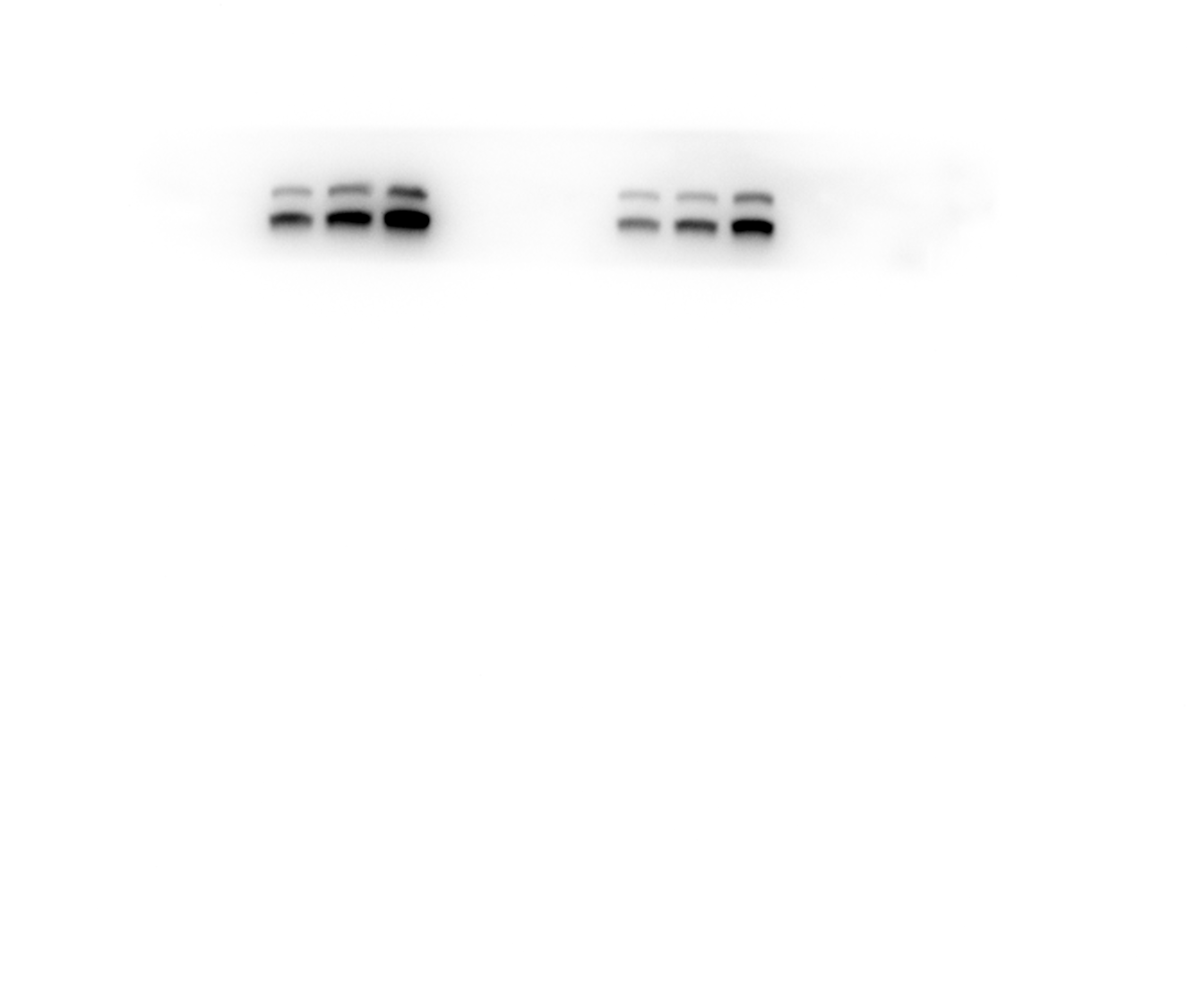

Supplement: S1 Dataset — (ZIP) [file ppat.1012800.s012.zip › Figs 1-9 minimal data set/fig 2/fig 2E/P-S6K.Tif]

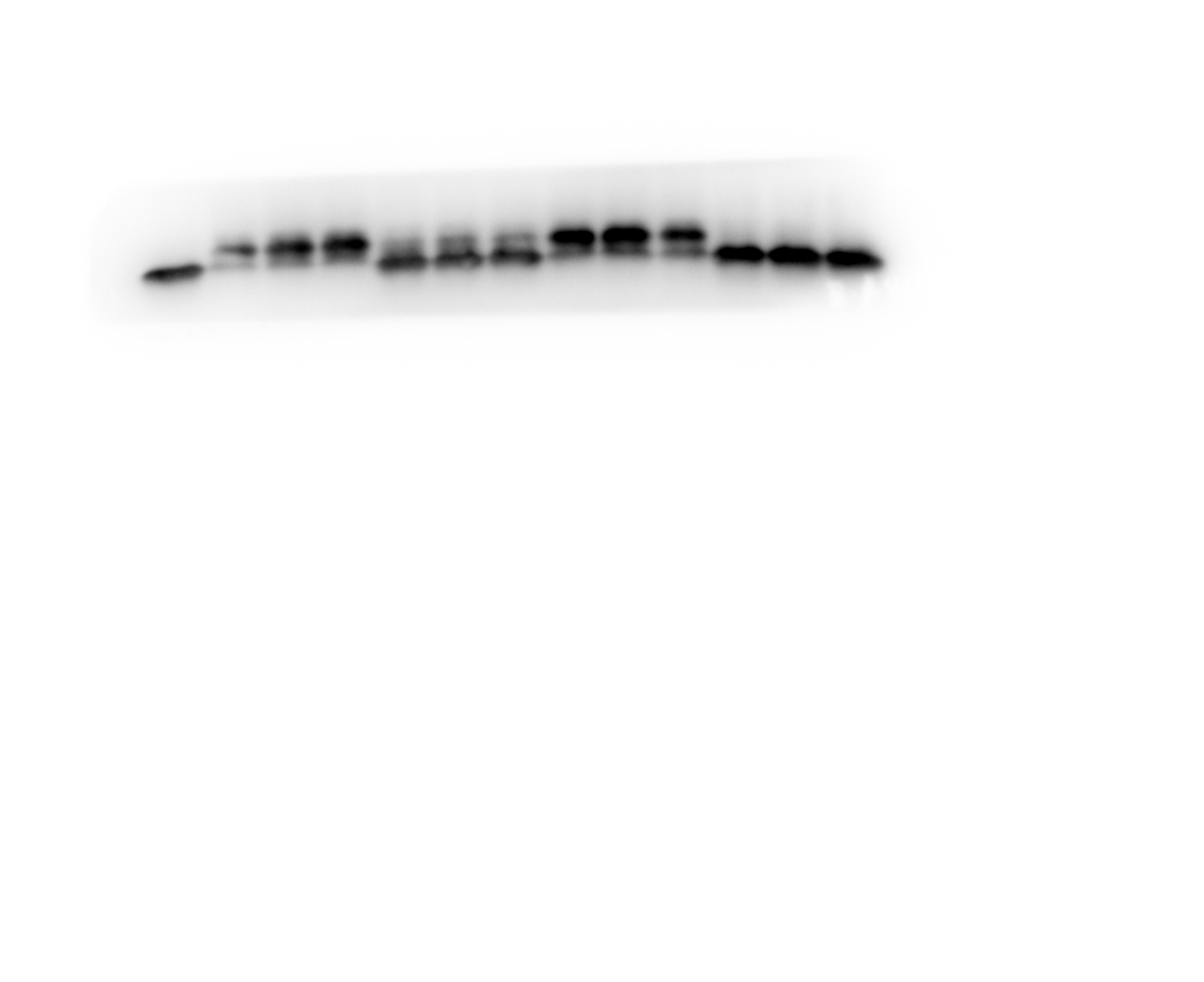

Supplement: S1 Dataset — (ZIP) [file ppat.1012800.s012.zip › Figs 1-9 minimal data set/fig 2/fig 2E/total 4EBP1.Tif]

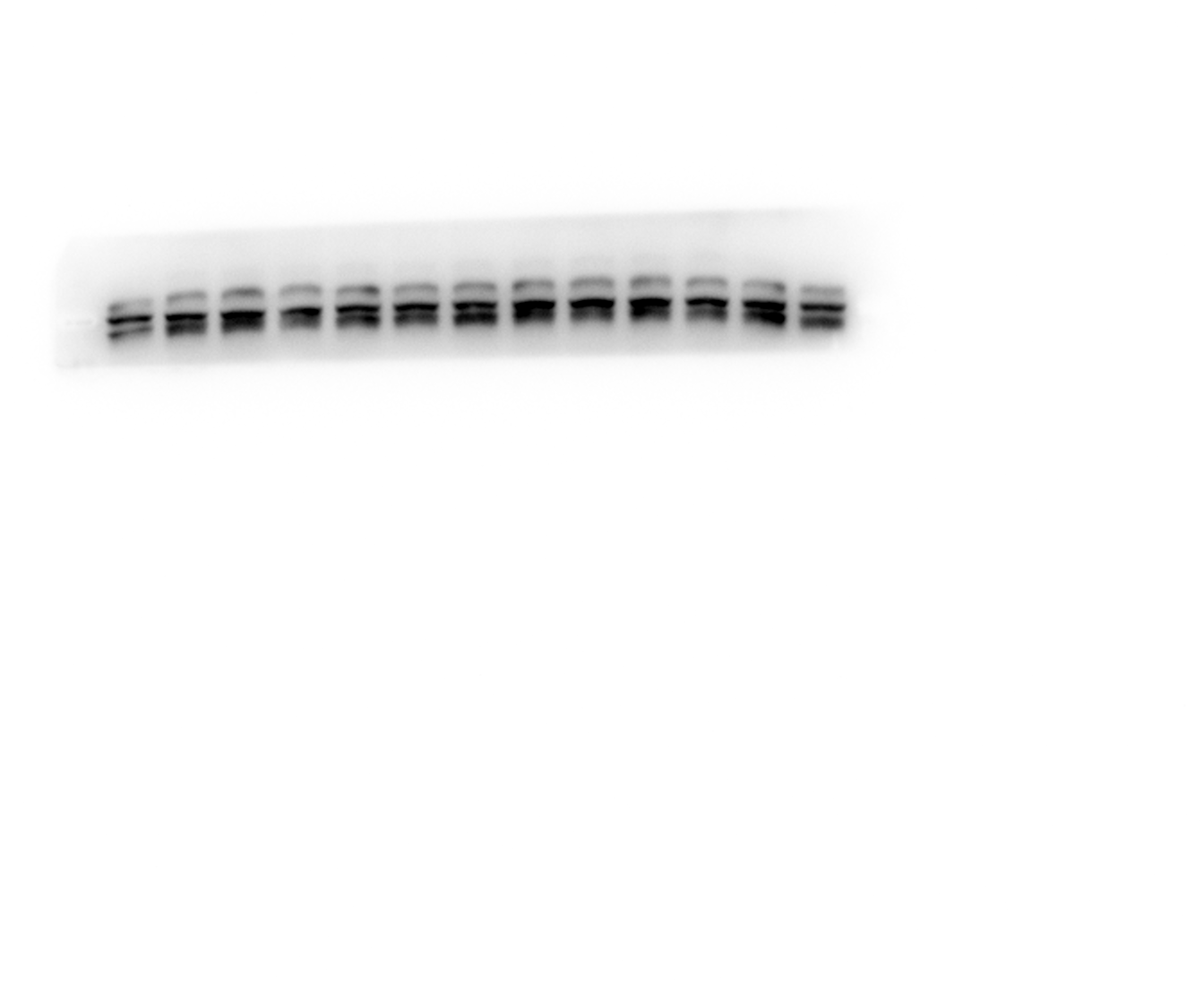

Supplement: S1 Dataset — (ZIP) [file ppat.1012800.s012.zip › Figs 1-9 minimal data set/fig 2/fig 2E/total S6K.Tif]

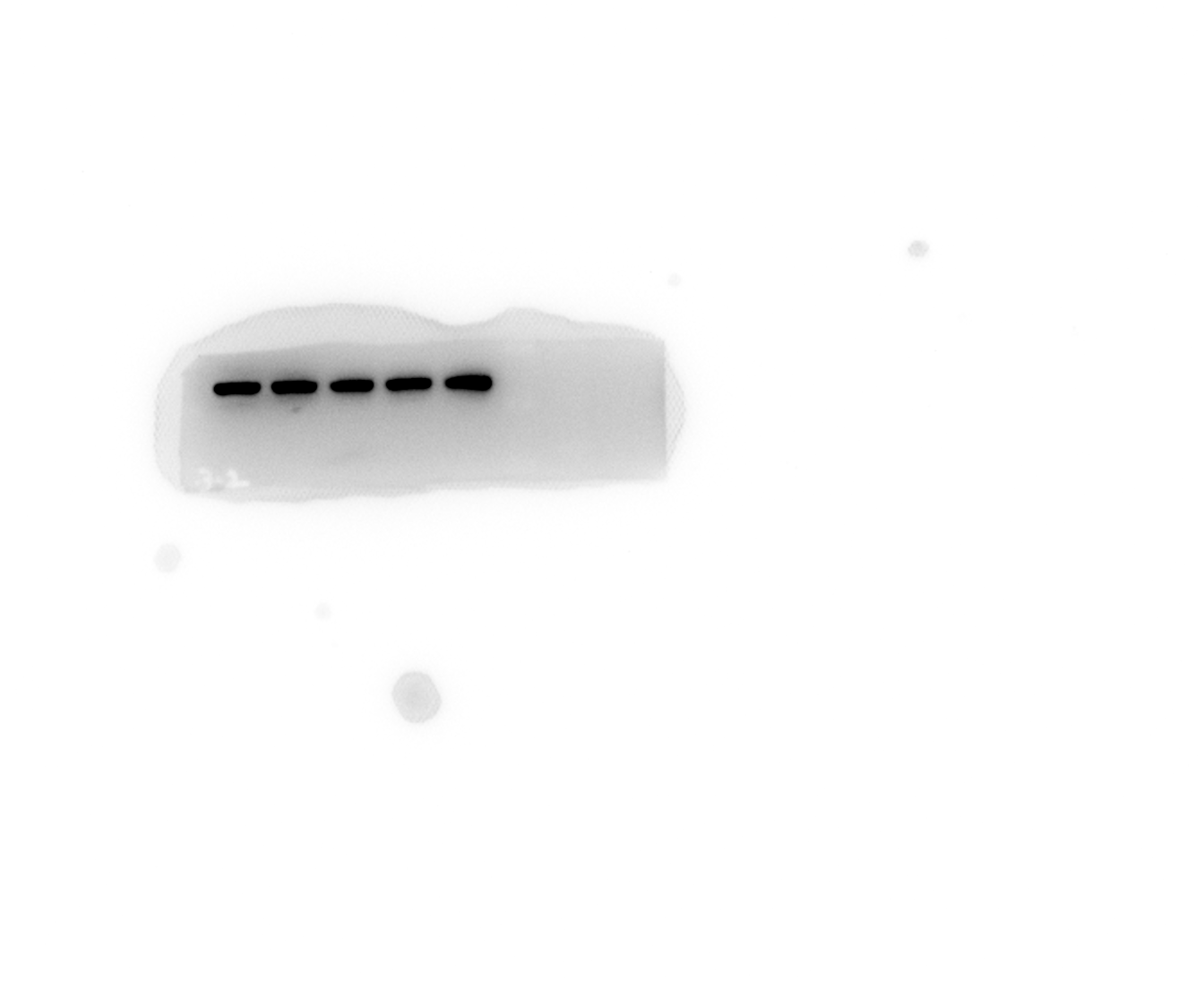

Supplement: S1 Dataset — (ZIP) [file ppat.1012800.s012.zip › Figs 1-9 minimal data set/fig 3/fig 3E/ACTB.Tif]

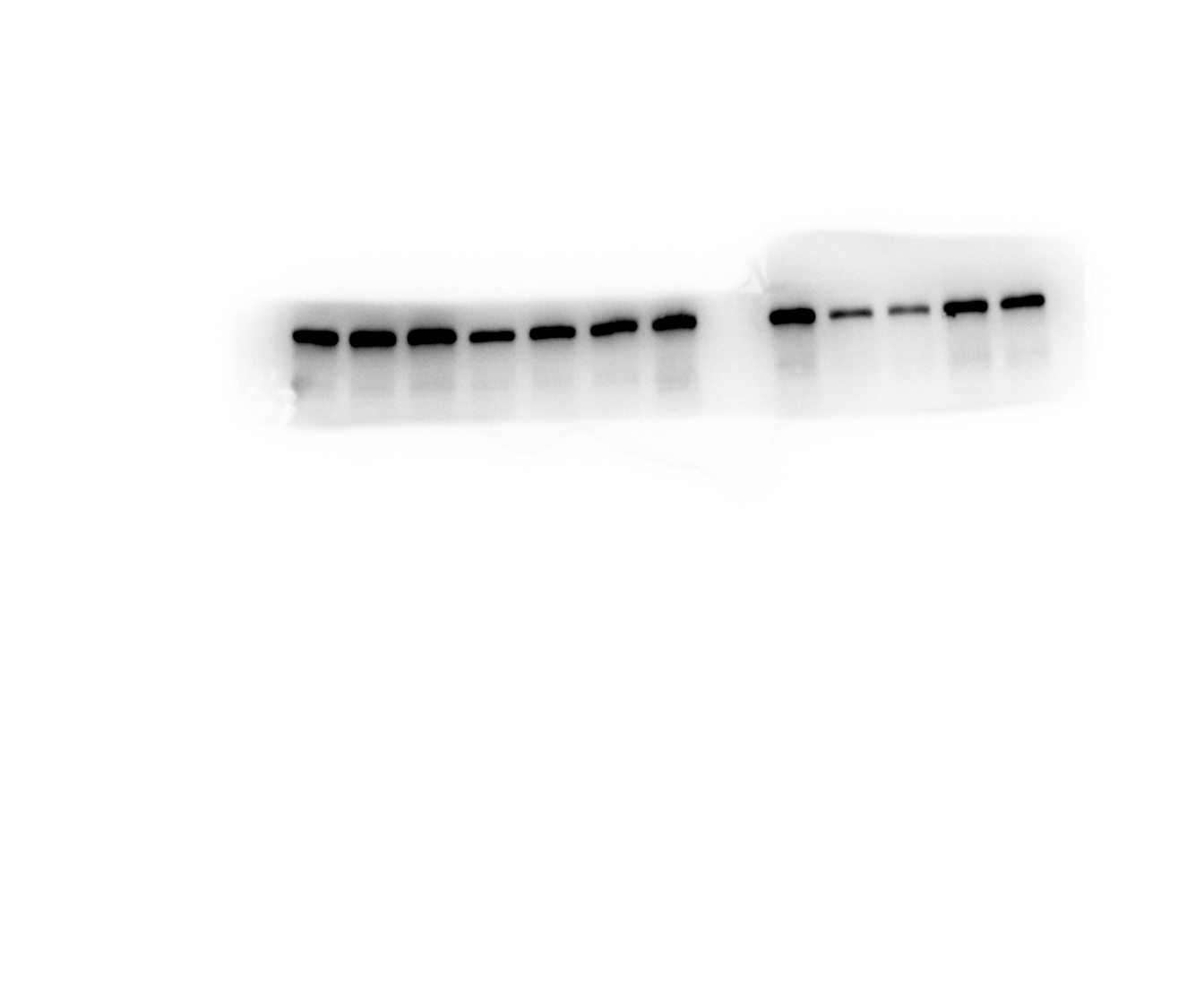

Supplement: S1 Dataset — (ZIP) [file ppat.1012800.s012.zip › Figs 1-9 minimal data set/fig 3/fig 3E/HRS.Tif]

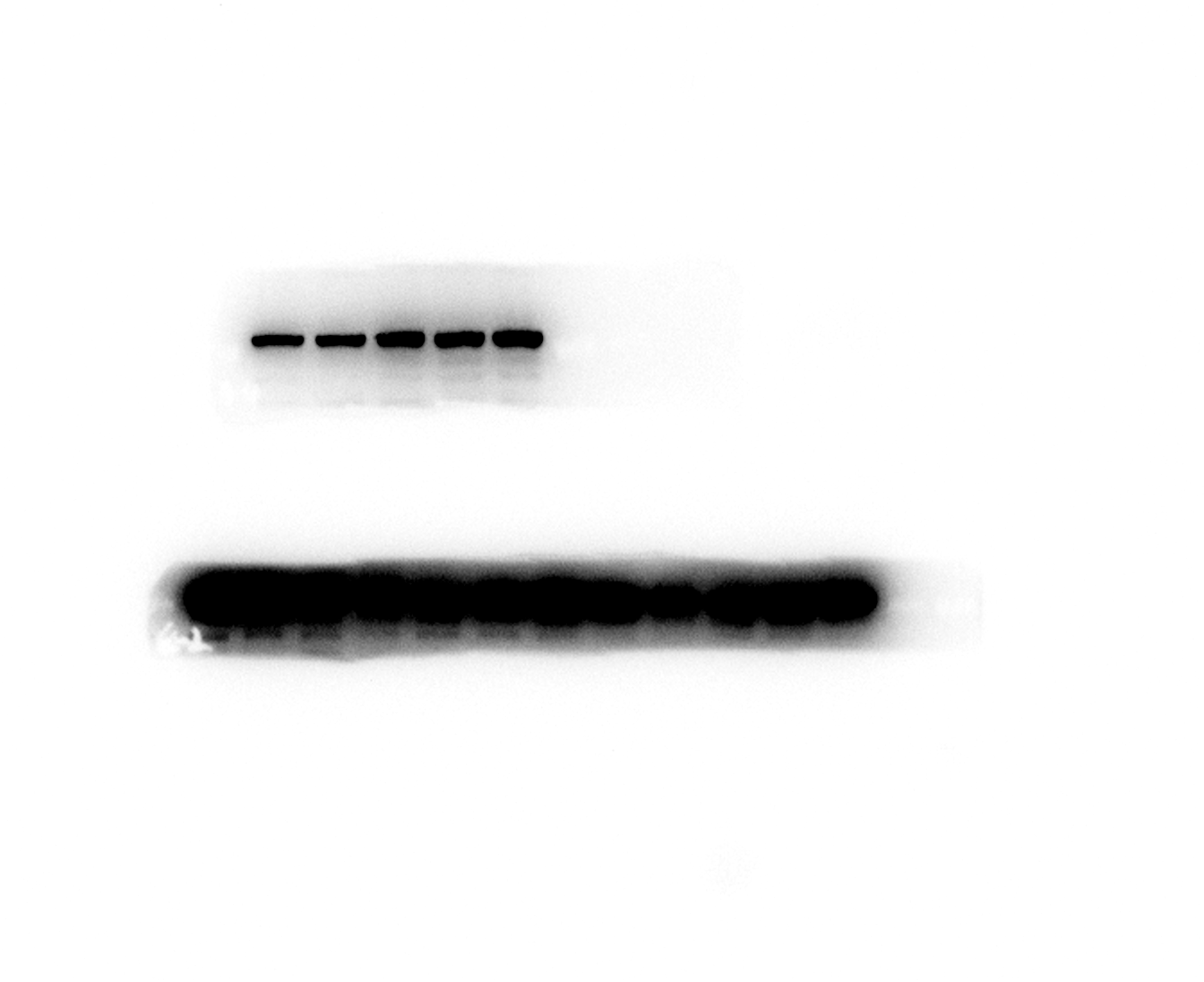

Supplement: S1 Dataset — (ZIP) [file ppat.1012800.s012.zip › Figs 1-9 minimal data set/fig 3/fig 3F/LaminB1, upper.Tif]

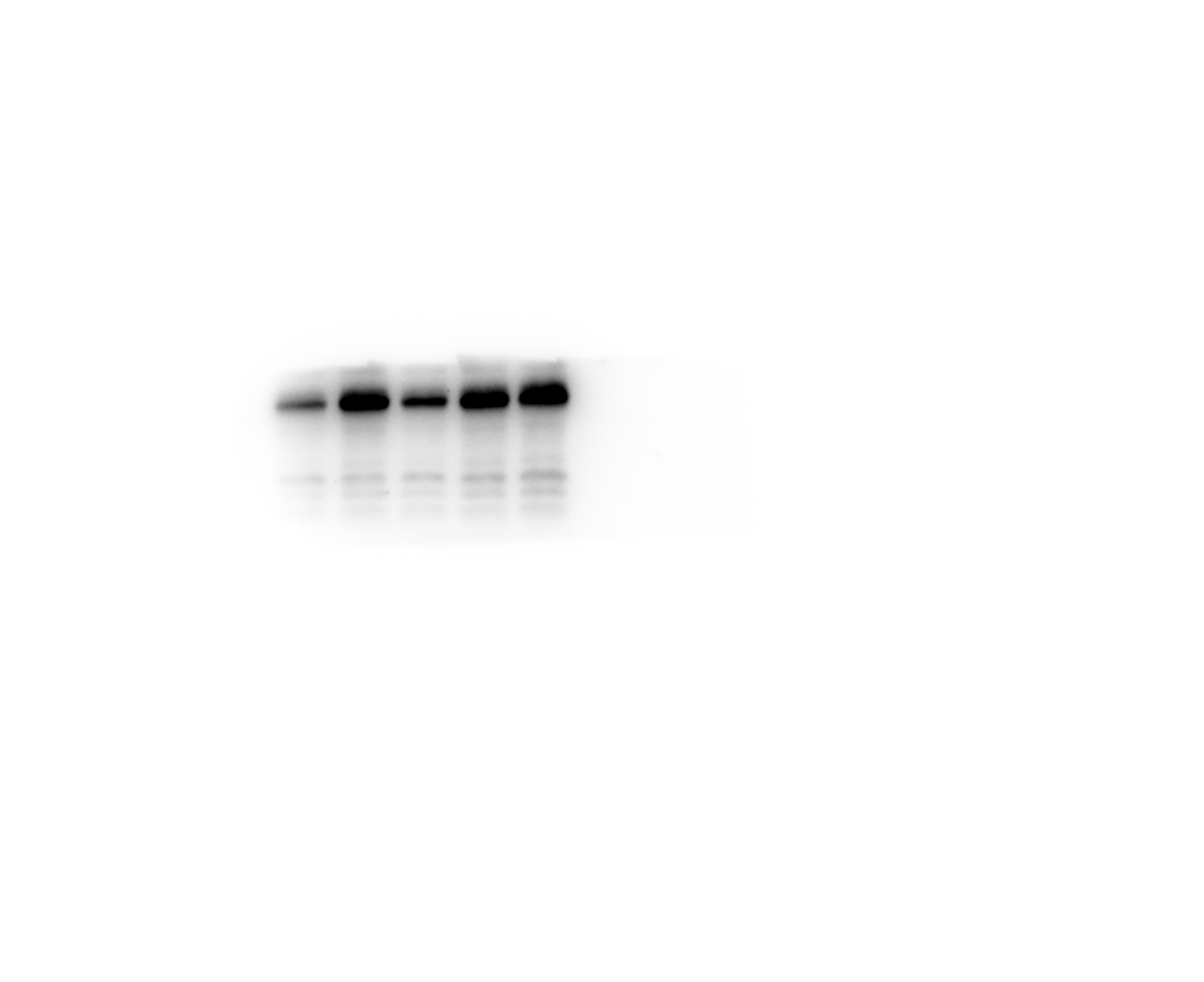

Supplement: S1 Dataset — (ZIP) [file ppat.1012800.s012.zip › Figs 1-9 minimal data set/fig 3/fig 3F/TSG101.Tif]

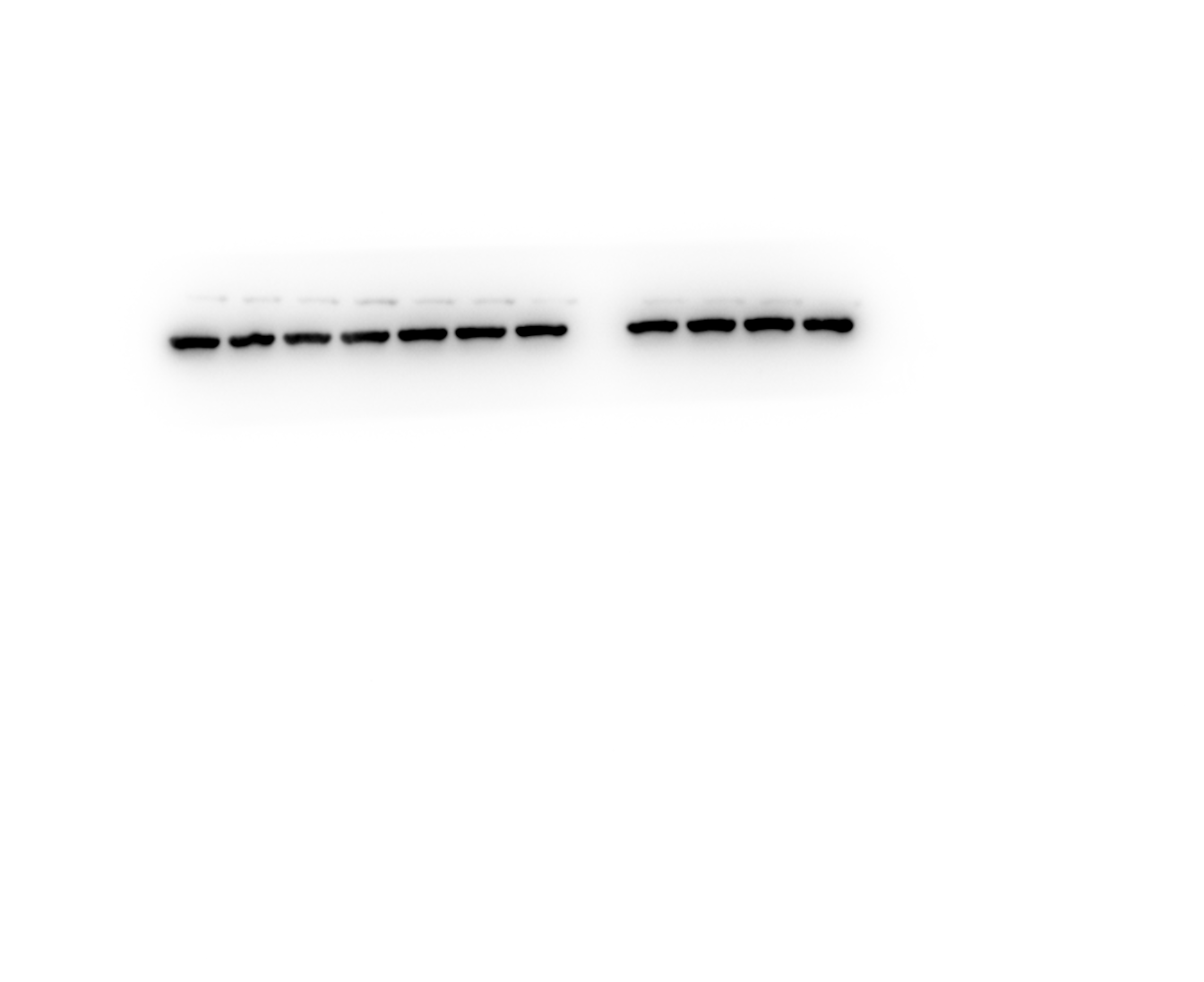

Supplement: S1 Dataset — (ZIP) [file ppat.1012800.s012.zip › Figs 1-9 minimal data set/fig 3/fig 3G/ACTB.Tif]

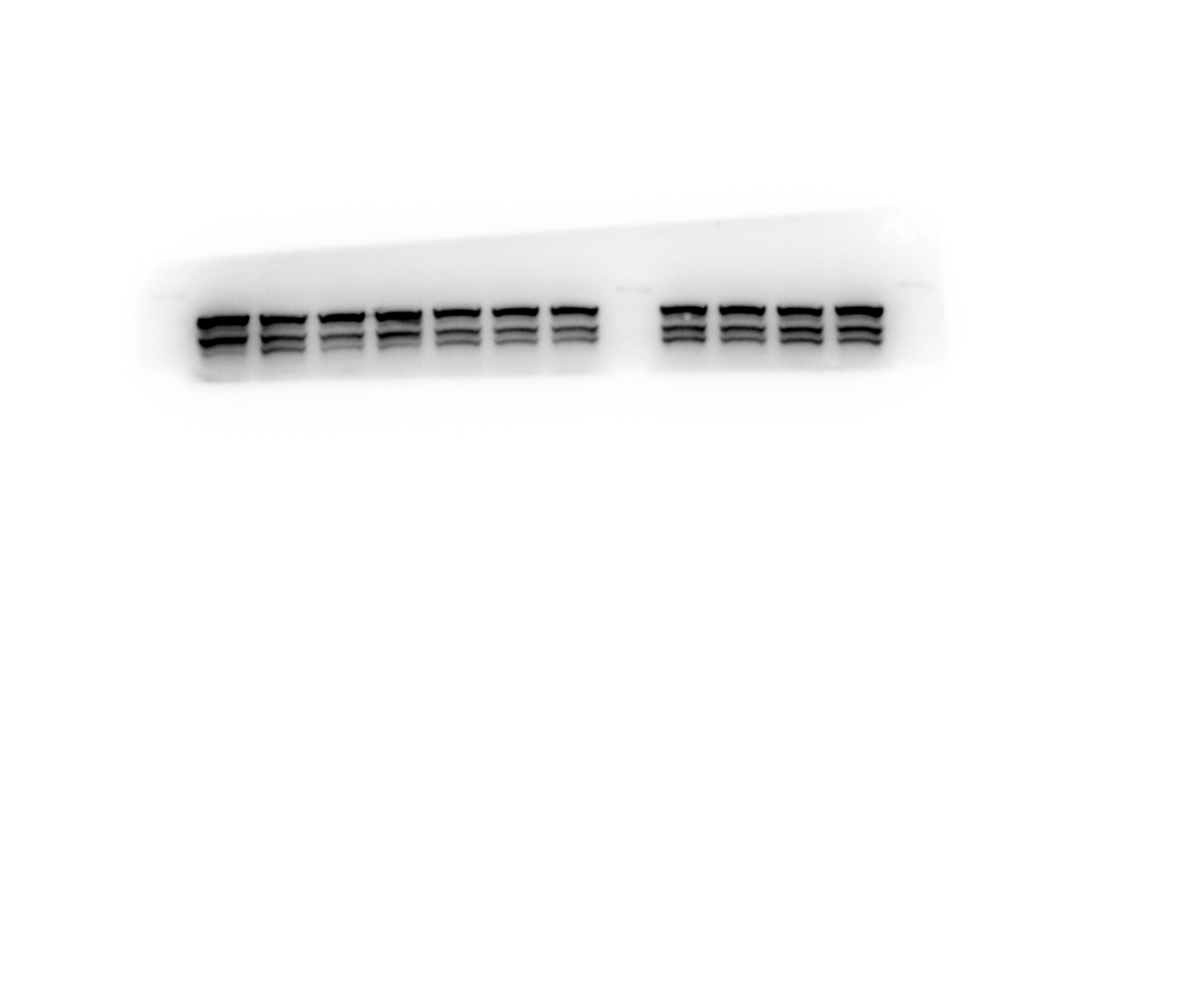

Supplement: S1 Dataset — (ZIP) [file ppat.1012800.s012.zip › Figs 1-9 minimal data set/fig 3/fig 3G/ALIX.Tif]

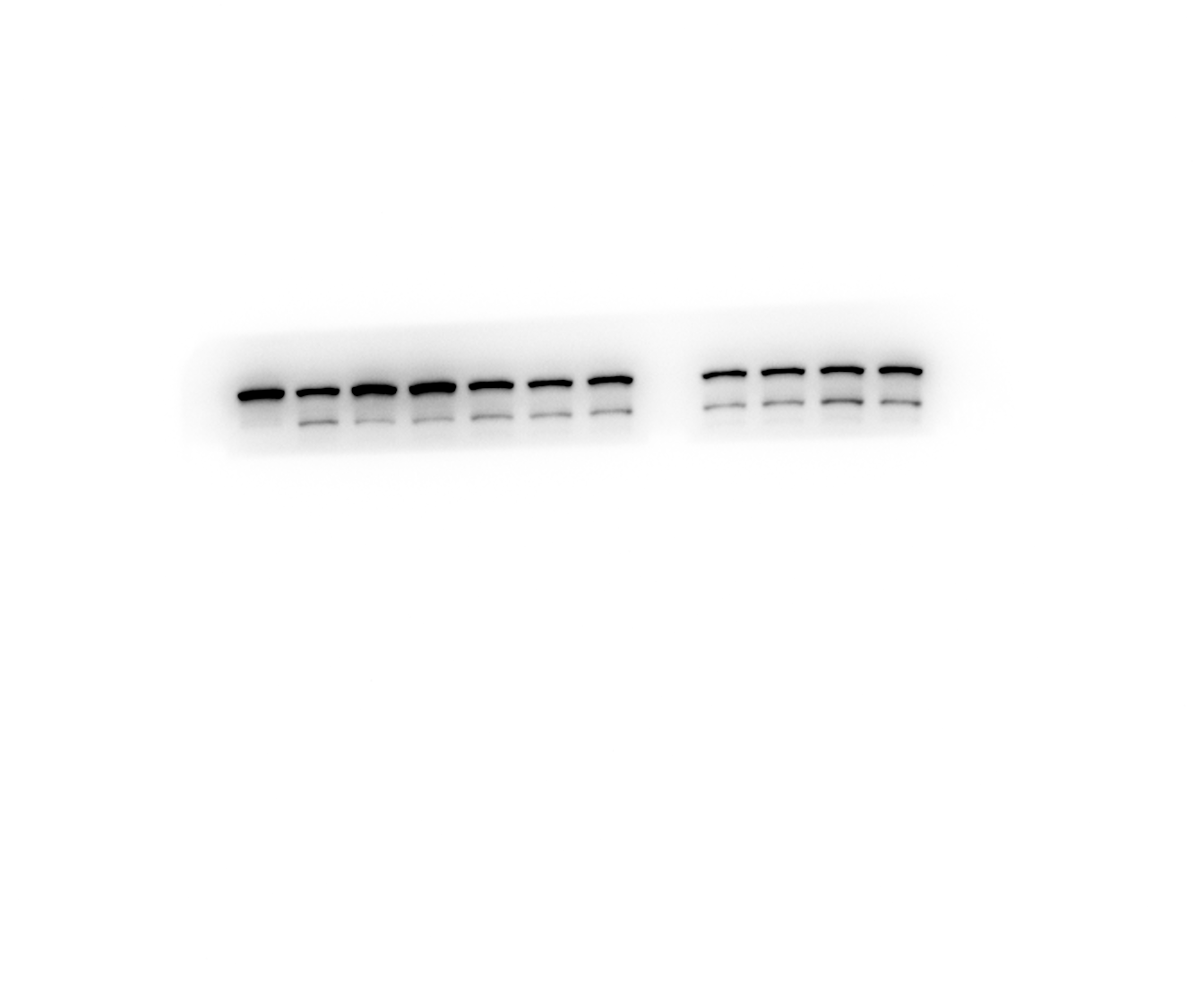

Supplement: S1 Dataset — (ZIP) [file ppat.1012800.s012.zip › Figs 1-9 minimal data set/fig 3/fig 3G/HRS.Tif]

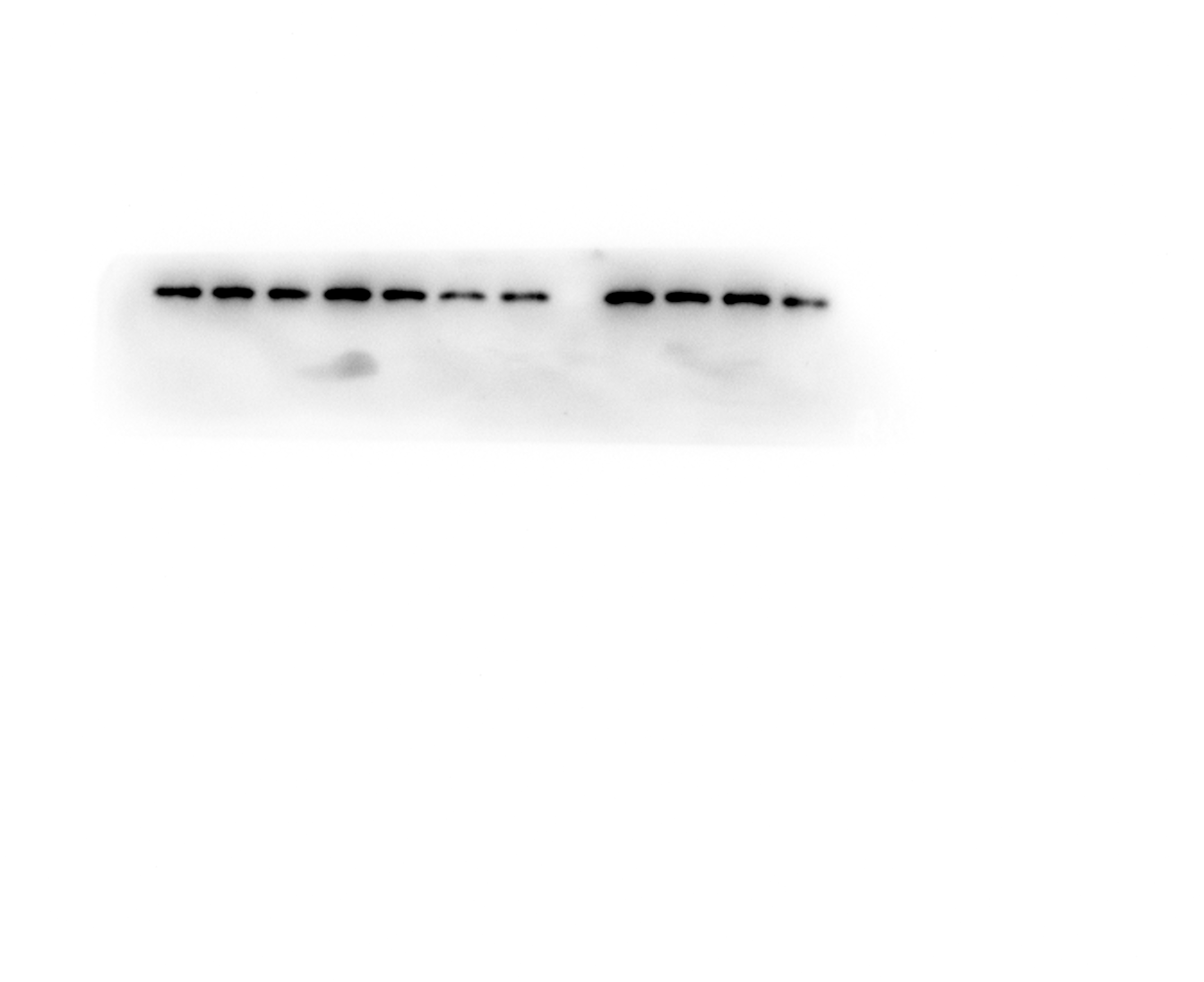

Supplement: S1 Dataset — (ZIP) [file ppat.1012800.s012.zip › Figs 1-9 minimal data set/fig 3/fig 3G/Rab5.Tif]

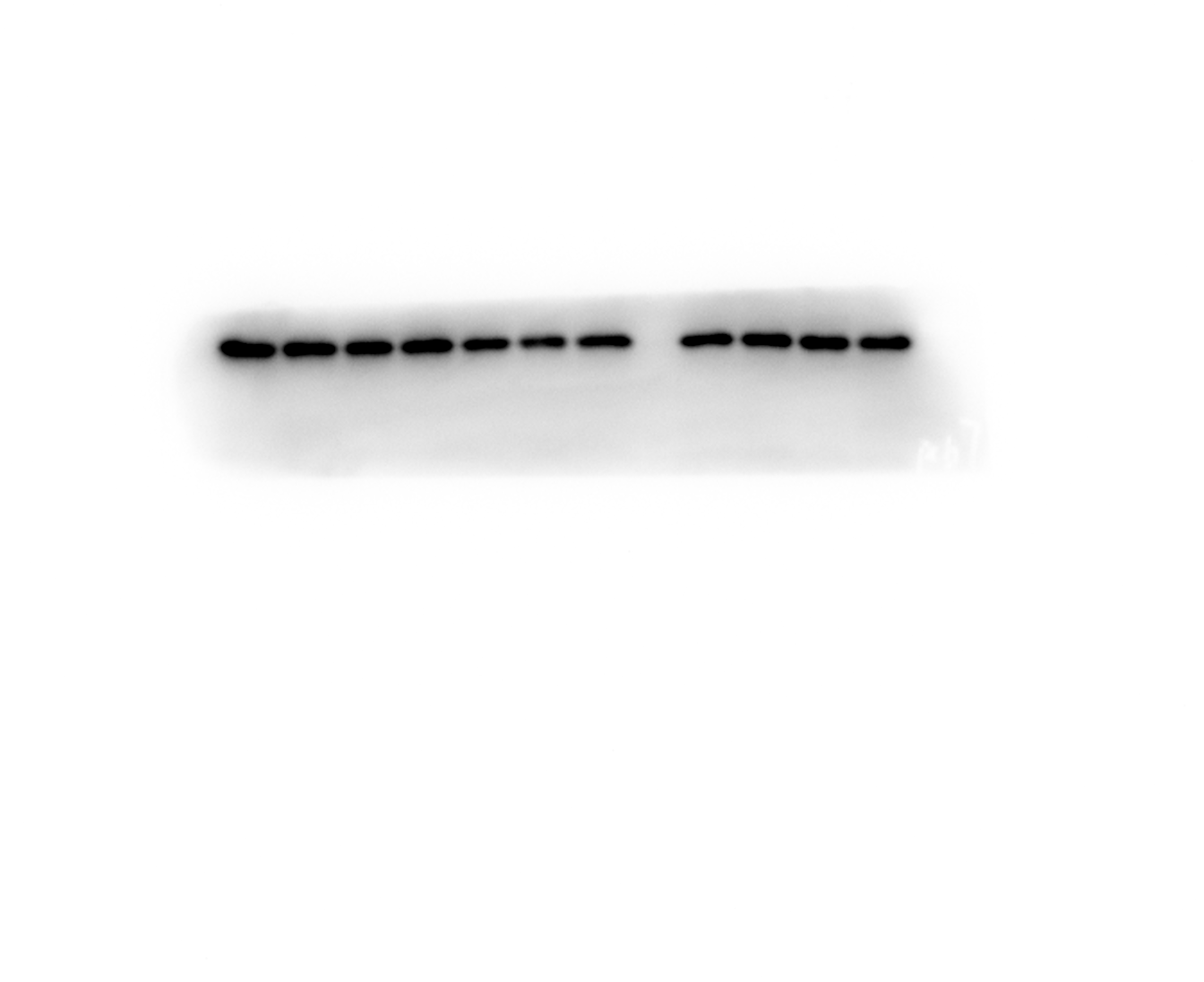

Supplement: S1 Dataset — (ZIP) [file ppat.1012800.s012.zip › Figs 1-9 minimal data set/fig 3/fig 3G/Rab7.Tif]

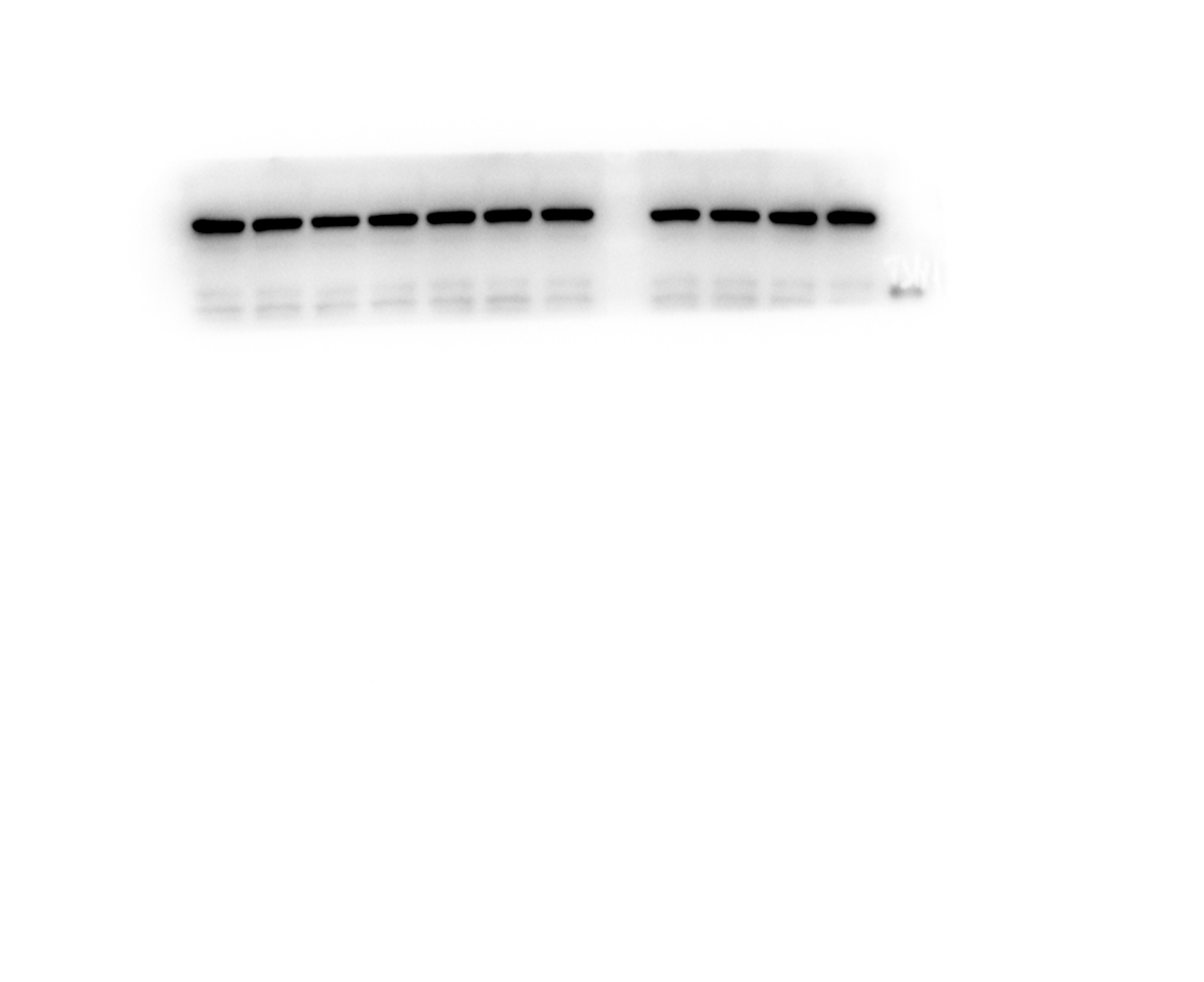

Supplement: S1 Dataset — (ZIP) [file ppat.1012800.s012.zip › Figs 1-9 minimal data set/fig 3/fig 3G/TSG101.Tif]

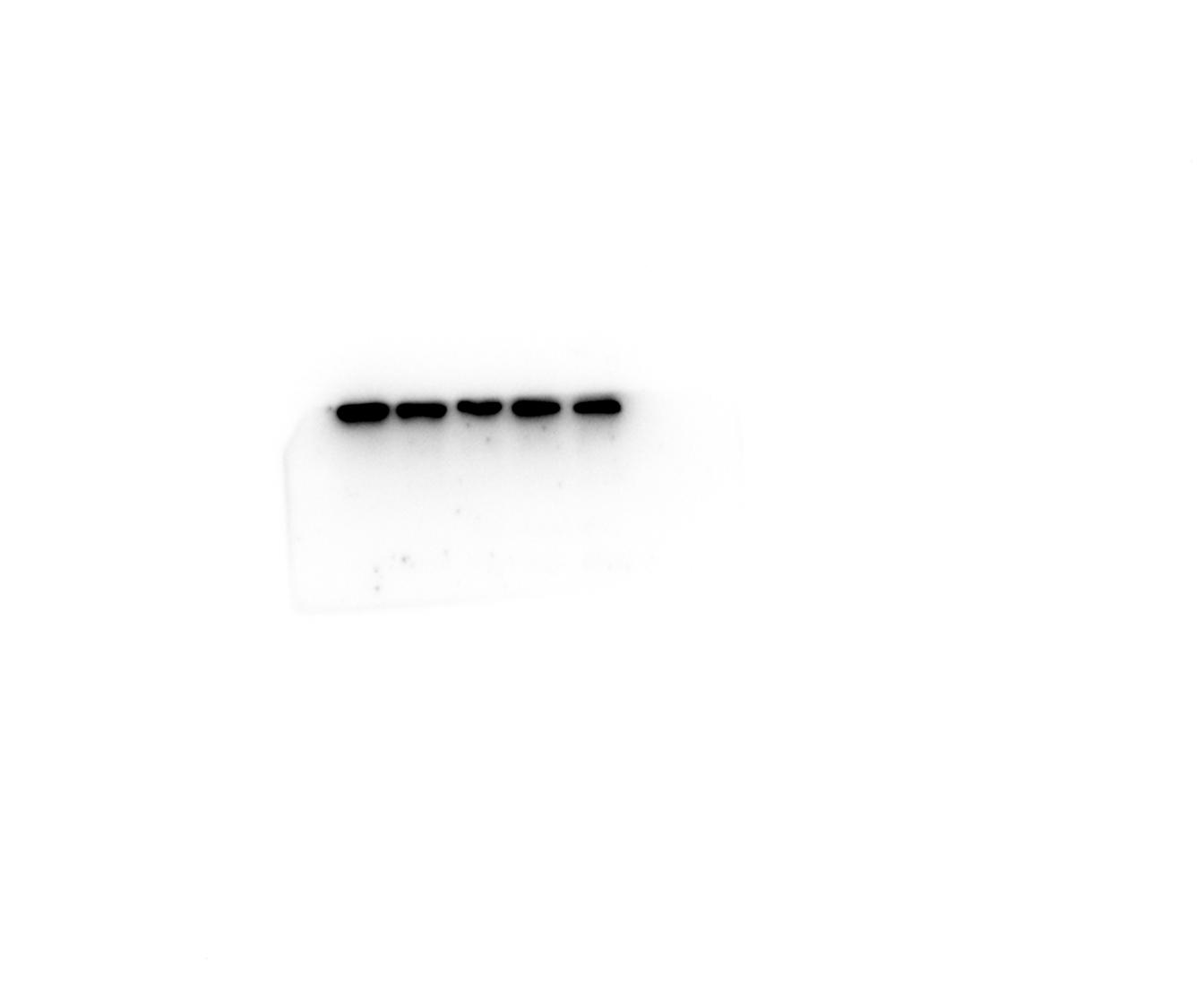

Supplement: S1 Dataset — (ZIP) [file ppat.1012800.s012.zip › Figs 1-9 minimal data set/fig 4/fig 4A/2.Tif]

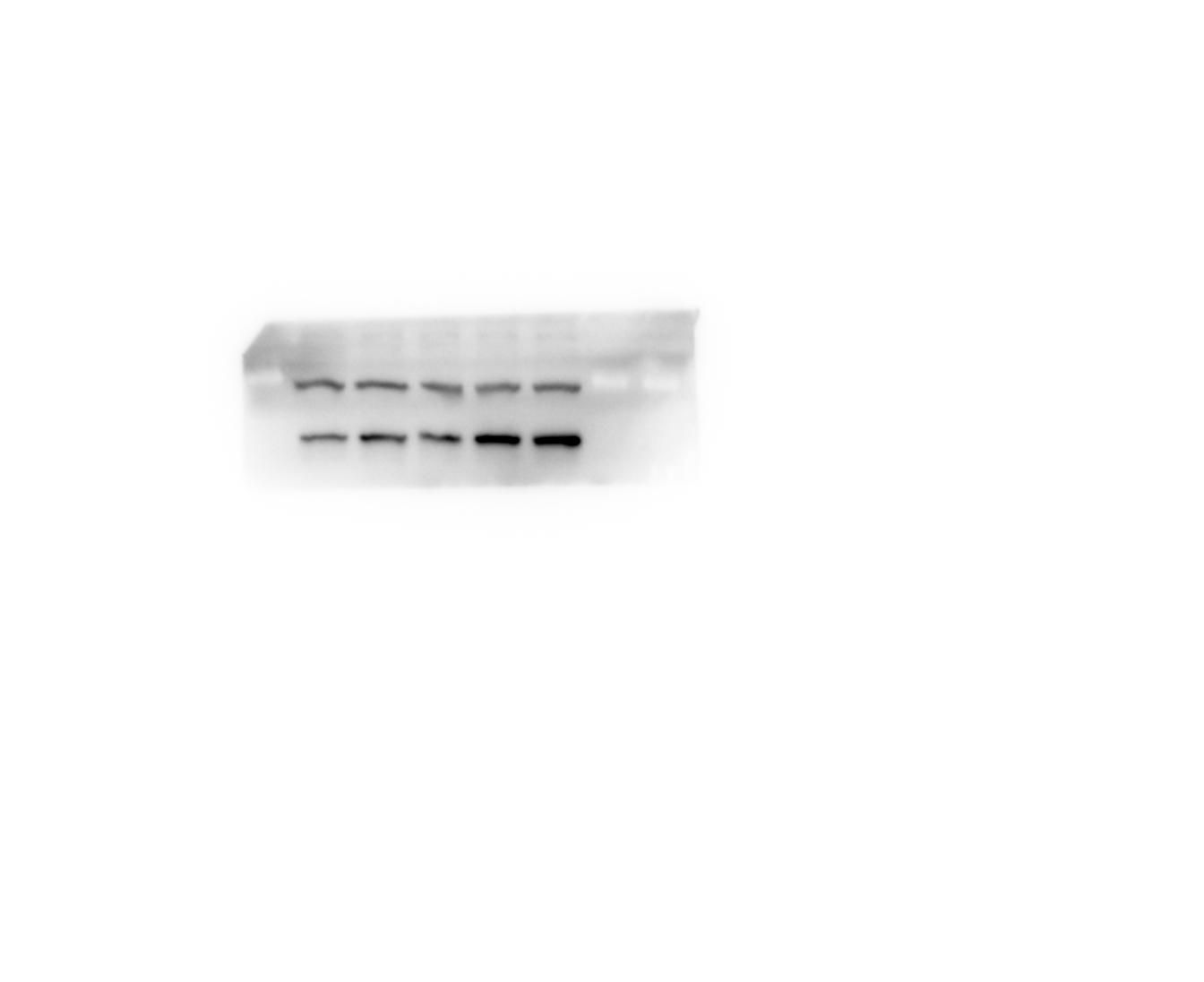

Supplement: S1 Dataset — (ZIP) [file ppat.1012800.s012.zip › Figs 1-9 minimal data set/fig 4/fig 4A/VAMP7.Tif]

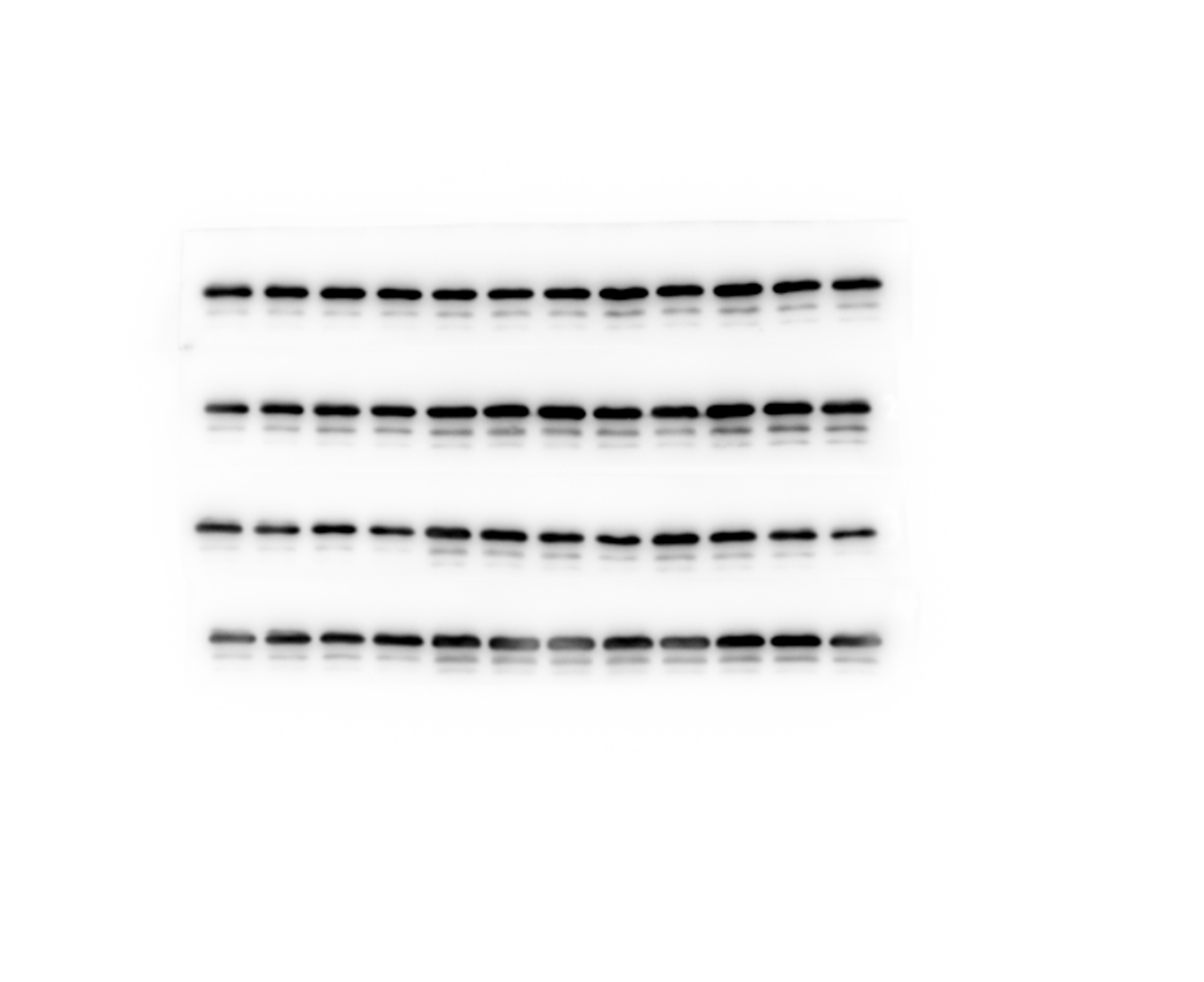

Supplement: S1 Dataset — (ZIP) [file ppat.1012800.s012.zip › Figs 1-9 minimal data set/fig 5/fig 5A/GAPDH, the third one.Tif]

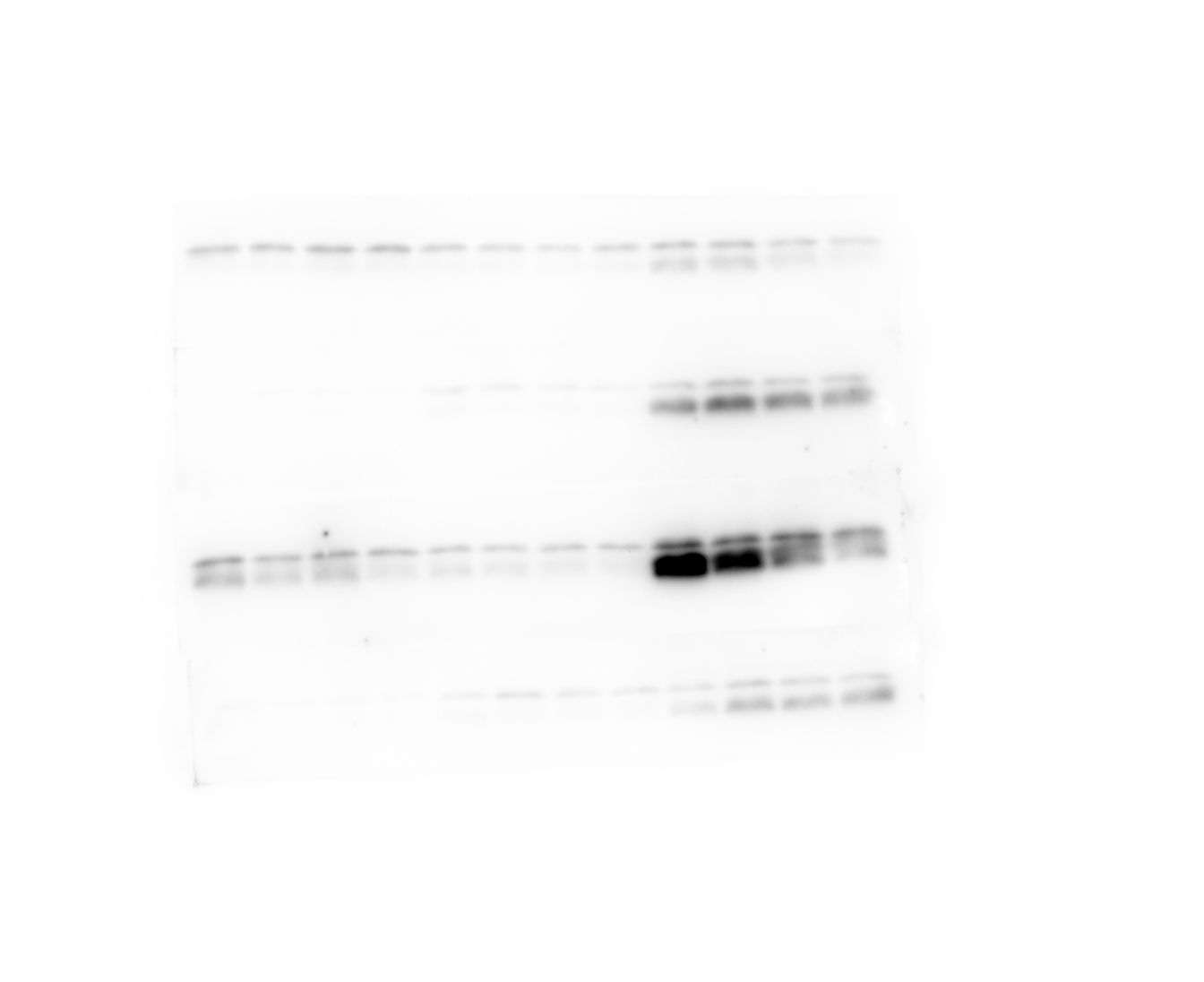

Supplement: S1 Dataset — (ZIP) [file ppat.1012800.s012.zip › Figs 1-9 minimal data set/fig 5/fig 5A/LC3B,the third one.Tif]

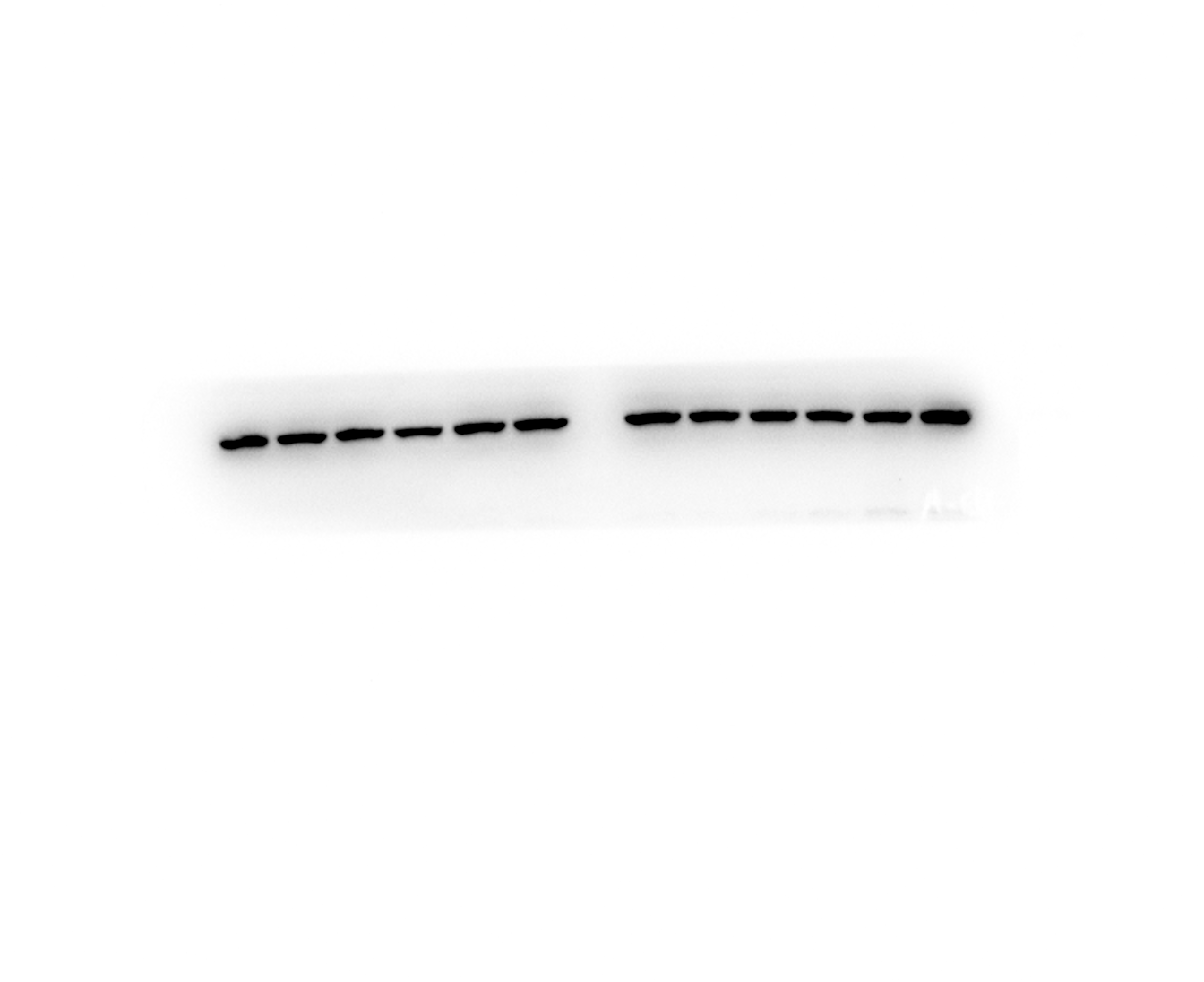

Supplement: S1 Dataset — (ZIP) [file ppat.1012800.s012.zip › Figs 1-9 minimal data set/fig 5/fig 5B/ACTB.Tif]

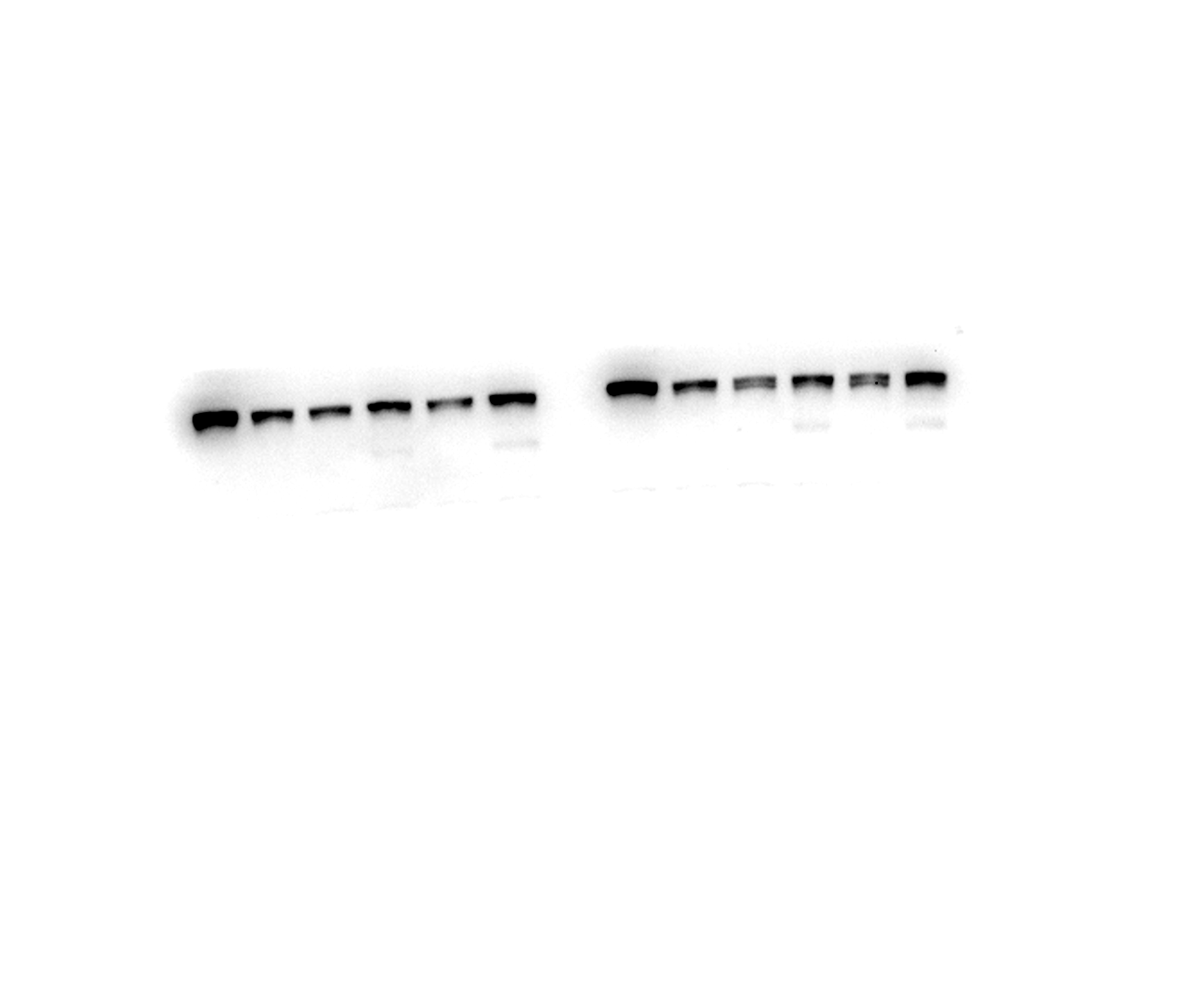

Supplement: S1 Dataset — (ZIP) [file ppat.1012800.s012.zip › Figs 1-9 minimal data set/fig 5/fig 5B/EGFR.Tif]

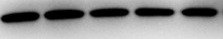

Supplement: S1 Dataset — (ZIP) [file ppat.1012800.s012.zip › Figs 1-9 minimal data set/fig 6/fig 6D/ACTB.tif]

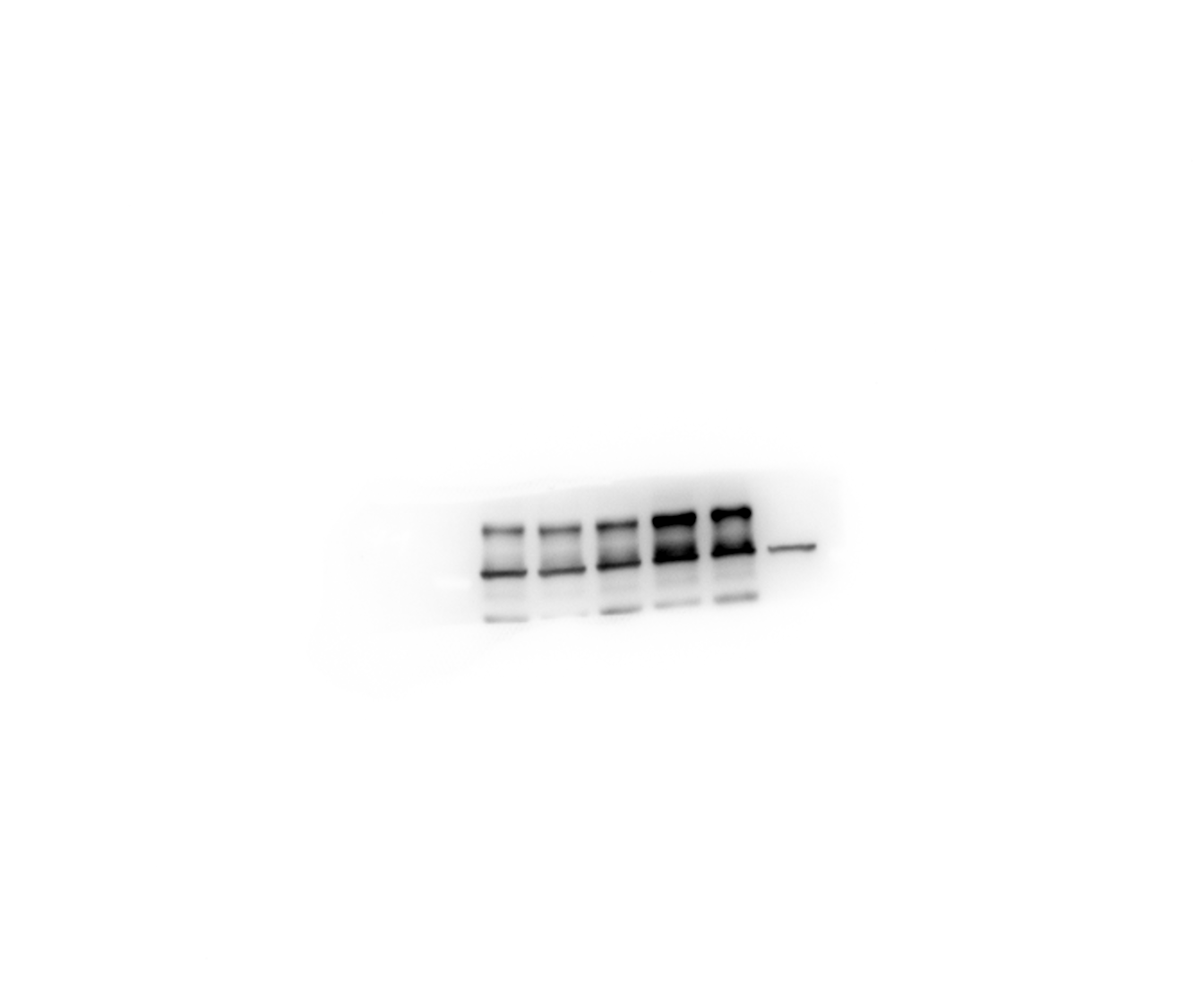

Supplement: S1 Dataset — (ZIP) [file ppat.1012800.s012.zip › Figs 1-9 minimal data set/fig 6/fig 6D/TFEB-GFP.Tif]

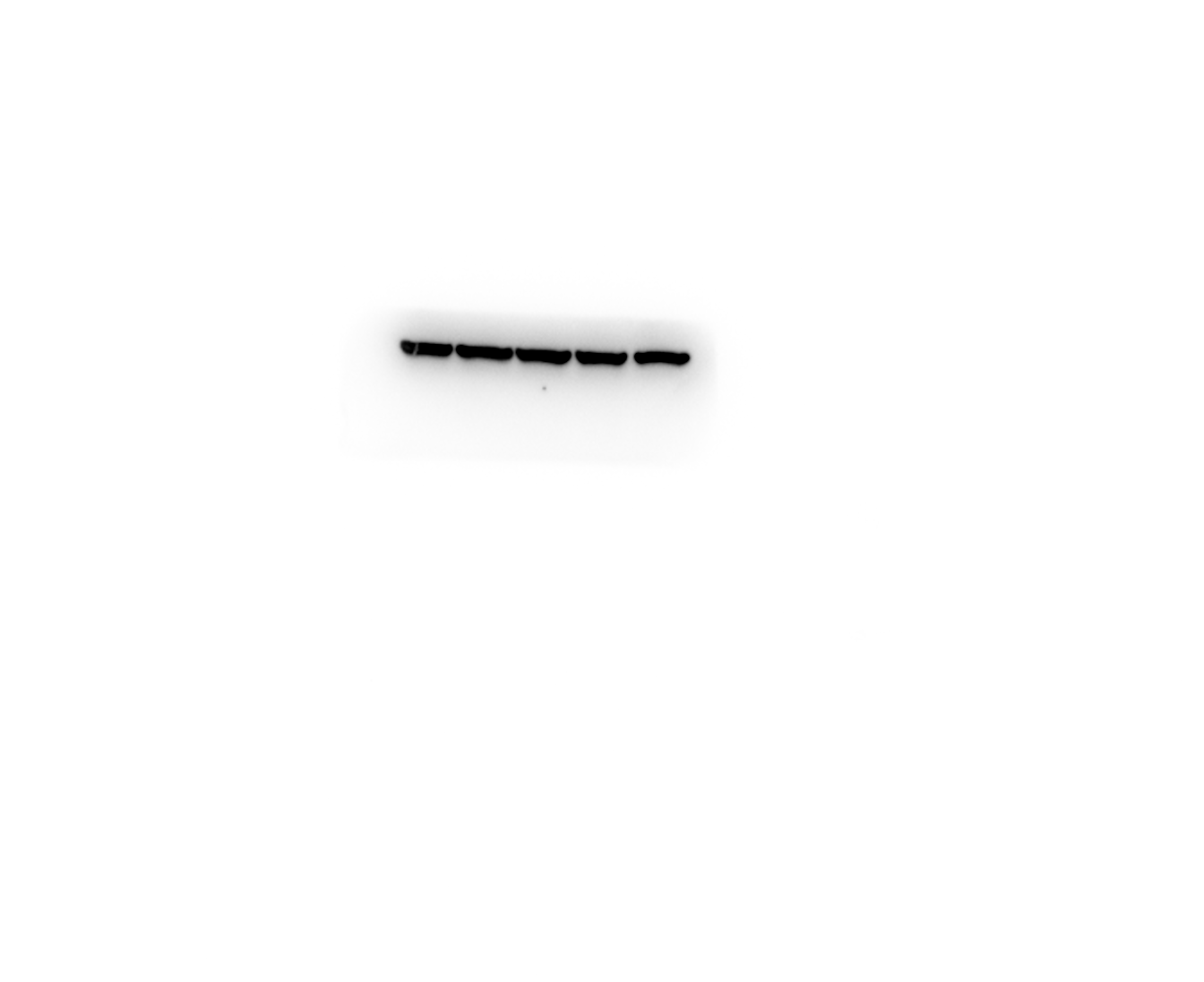

Supplement: S1 Dataset — (ZIP) [file ppat.1012800.s012.zip › Figs 1-9 minimal data set/fig 7/Fig 7C/ACTB.Tif]

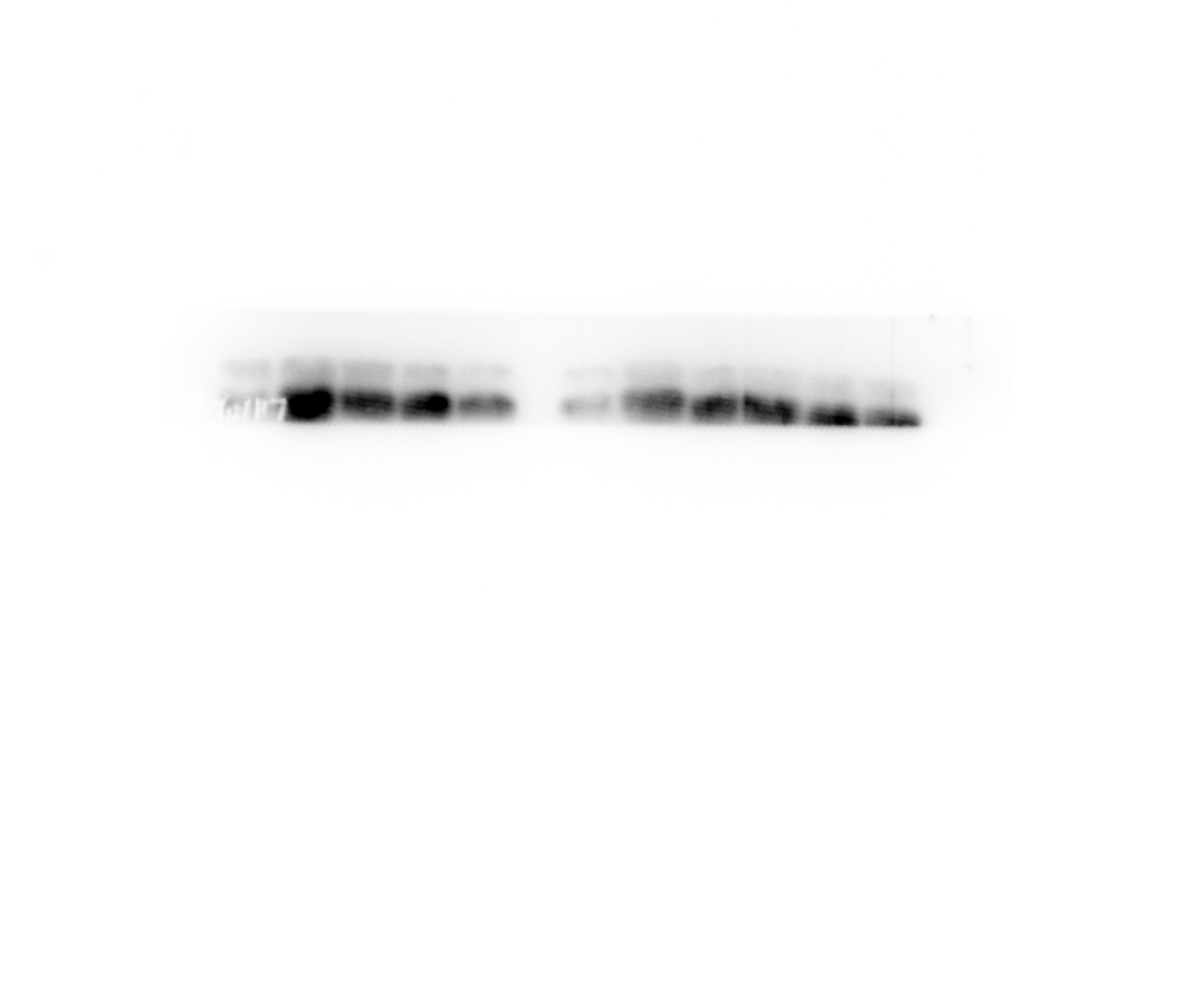

Supplement: S1 Dataset — (ZIP) [file ppat.1012800.s012.zip › Figs 1-9 minimal data set/fig 7/Fig 7C/LC3B.Tif]

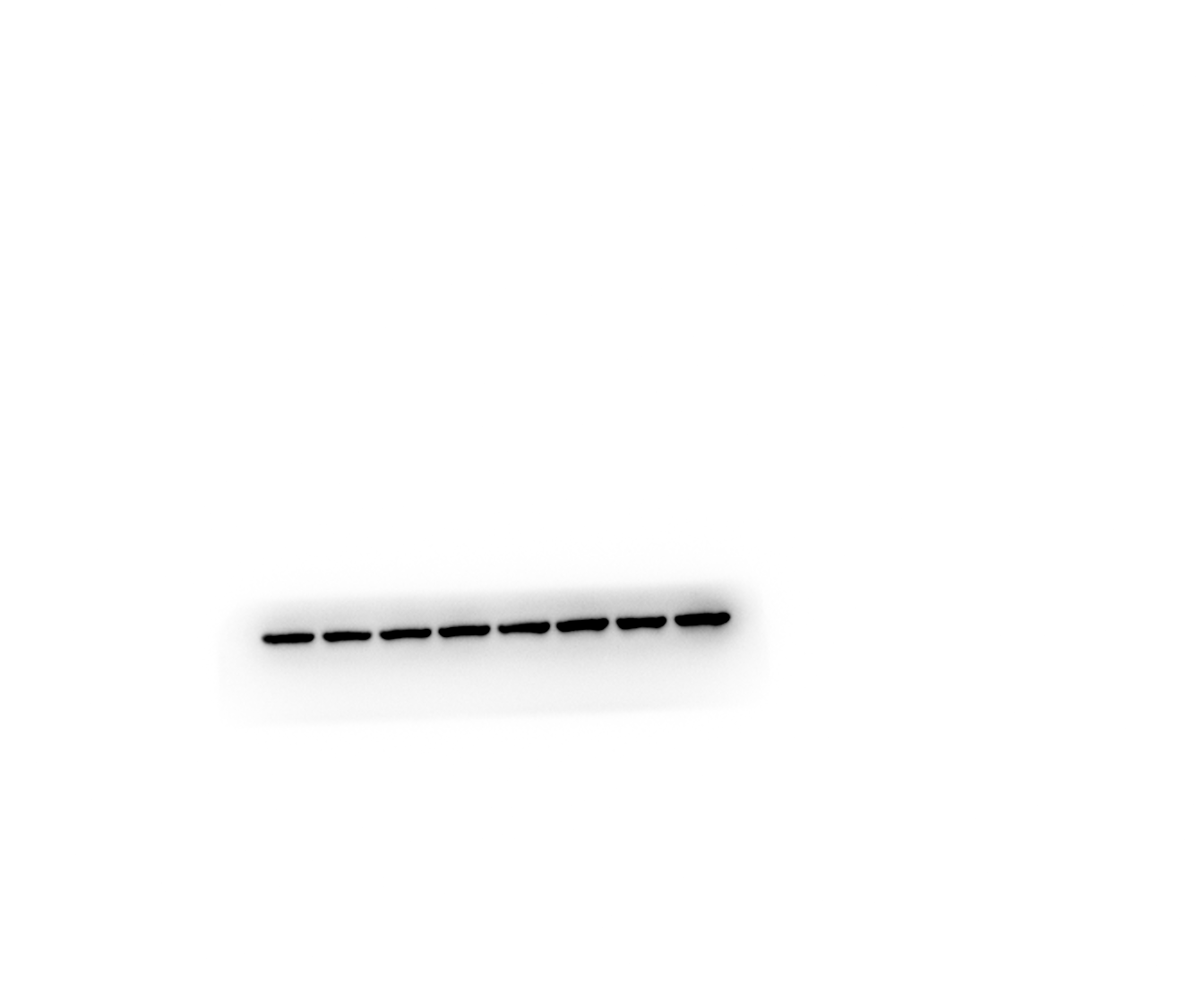

Supplement: S1 Dataset — (ZIP) [file ppat.1012800.s012.zip › Figs 1-9 minimal data set/fig 7/fig 7B/ACTB for left panel.Tif]

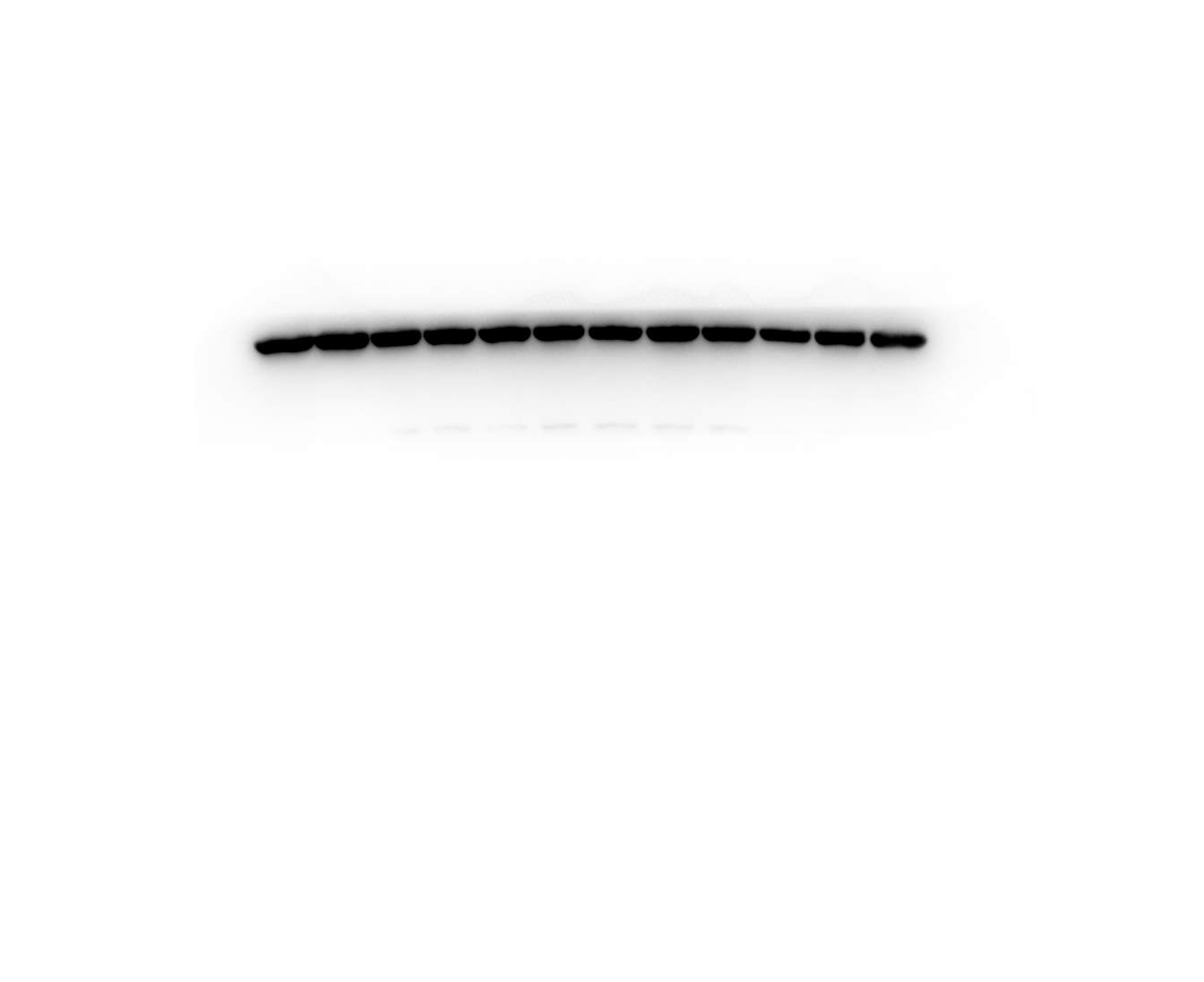

Supplement: S1 Dataset — (ZIP) [file ppat.1012800.s012.zip › Figs 1-9 minimal data set/fig 7/fig 7B/ACTB for right panel.Tif]

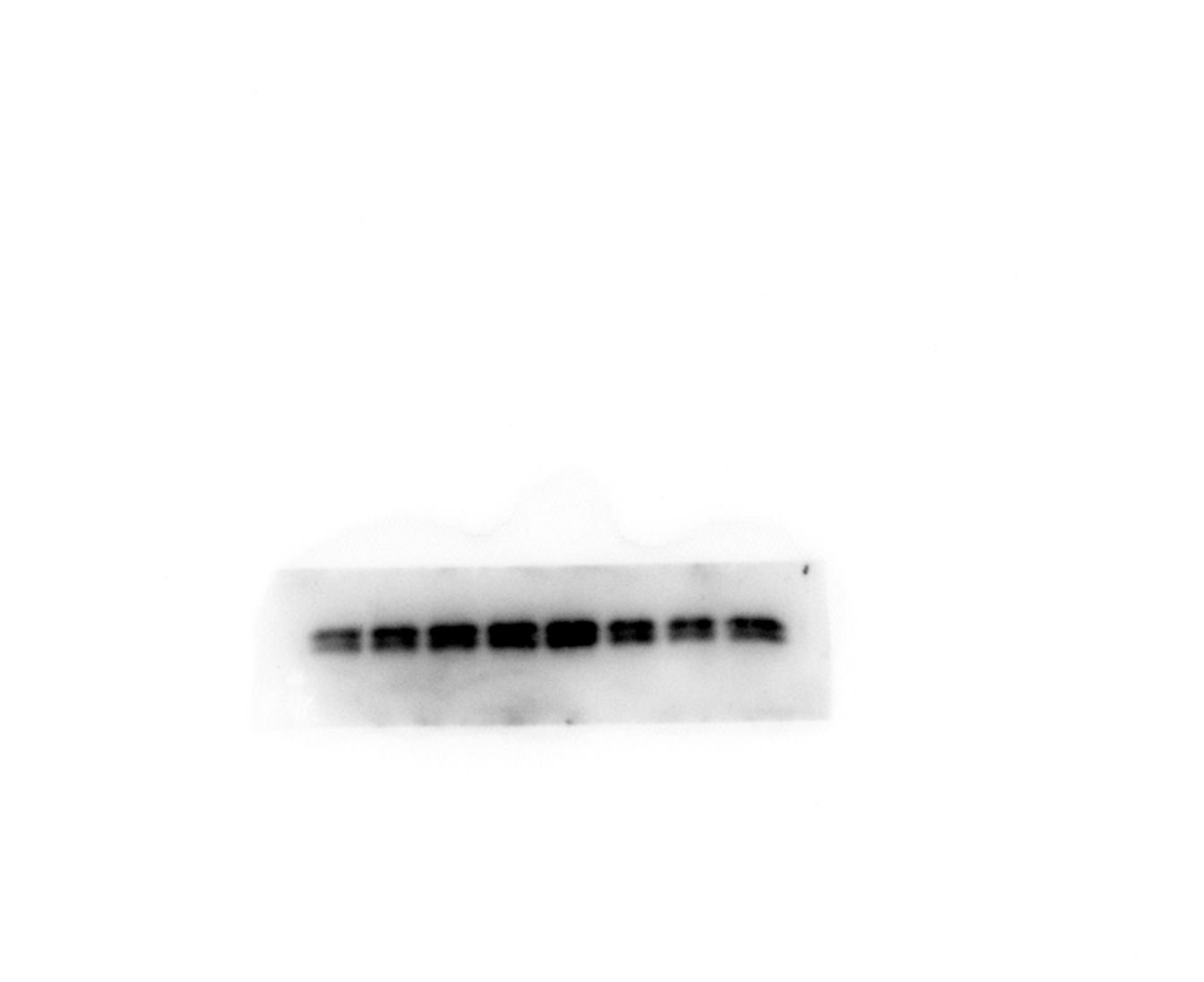

Supplement: S1 Dataset — (ZIP) [file ppat.1012800.s012.zip › Figs 1-9 minimal data set/fig 7/fig 7B/P-4EBP1.Tif]

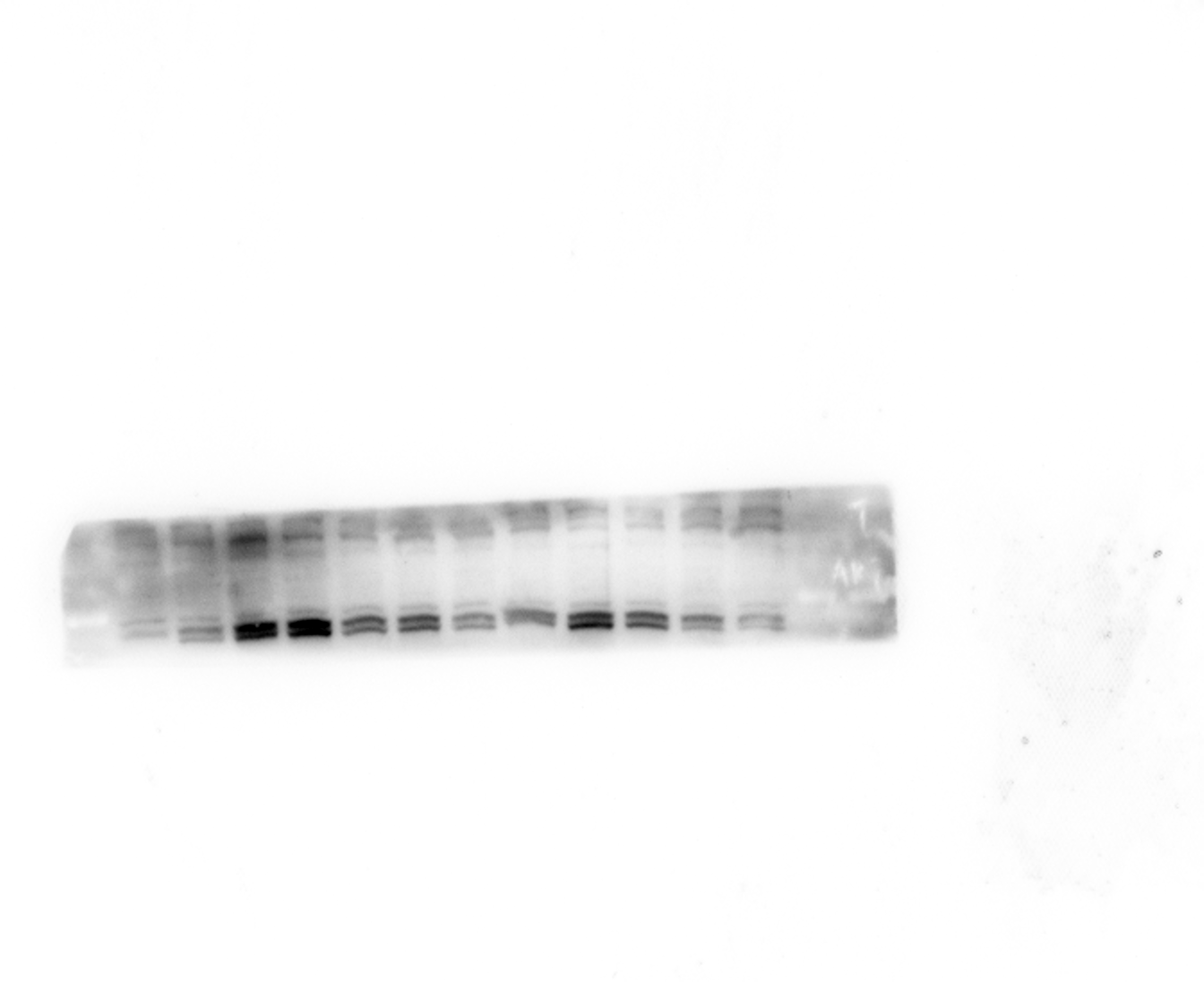

Supplement: S1 Dataset — (ZIP) [file ppat.1012800.s012.zip › Figs 1-9 minimal data set/fig 7/fig 7B/P-AKT.Tif]

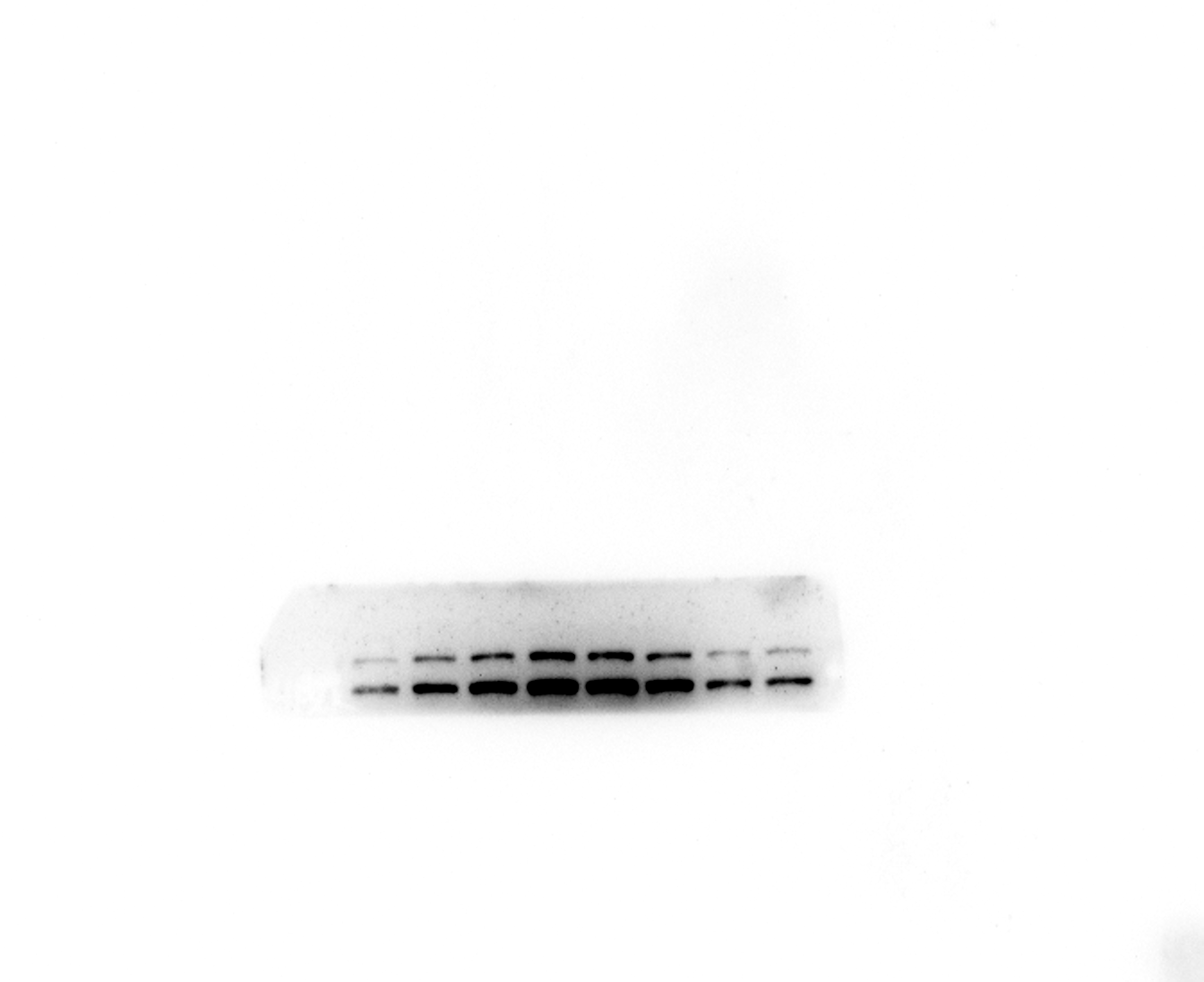

Supplement: S1 Dataset — (ZIP) [file ppat.1012800.s012.zip › Figs 1-9 minimal data set/fig 7/fig 7B/P-S6K.Tif]

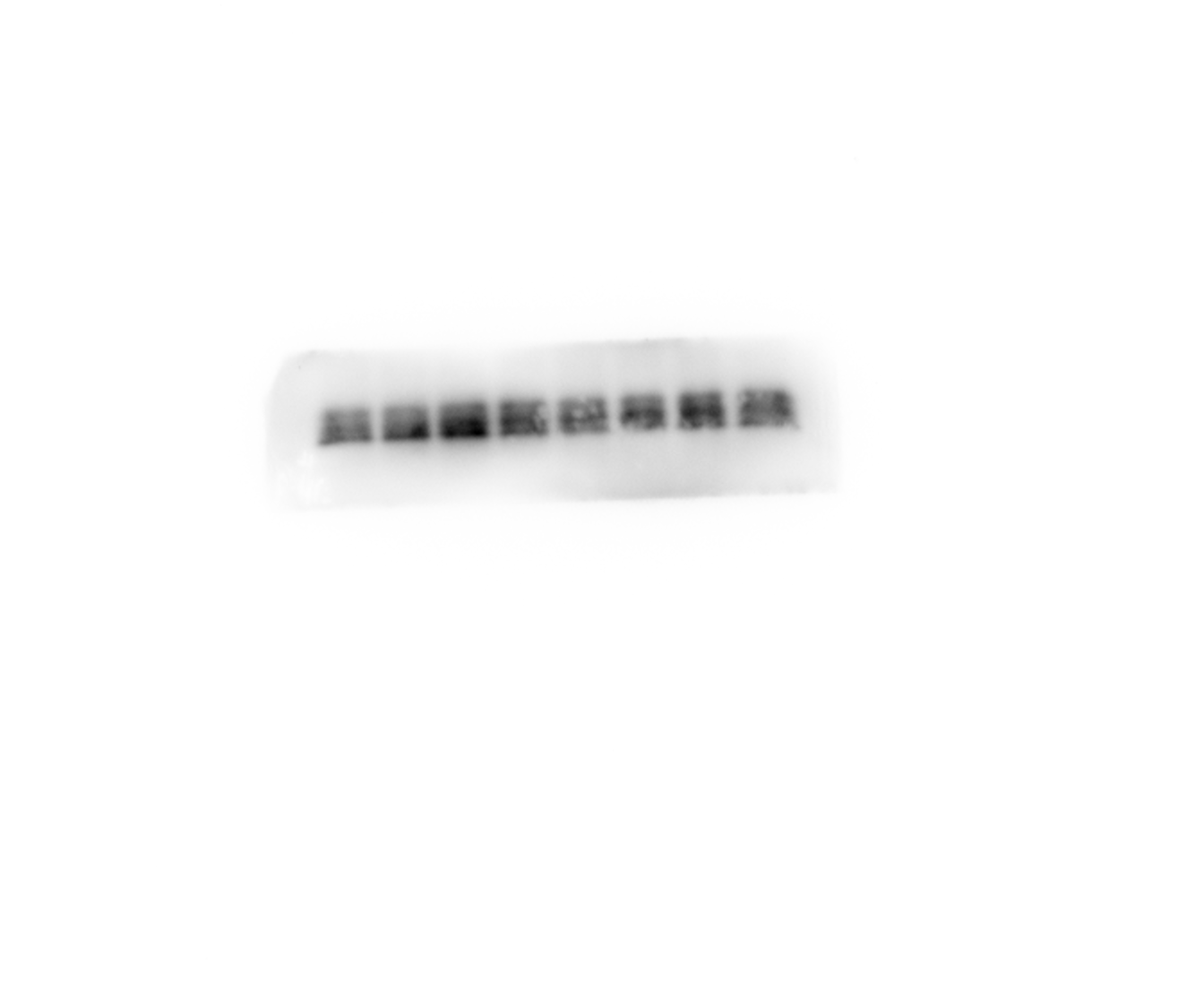

Supplement: S1 Dataset — (ZIP) [file ppat.1012800.s012.zip › Figs 1-9 minimal data set/fig 7/fig 7B/total 4EBP1.Tif]

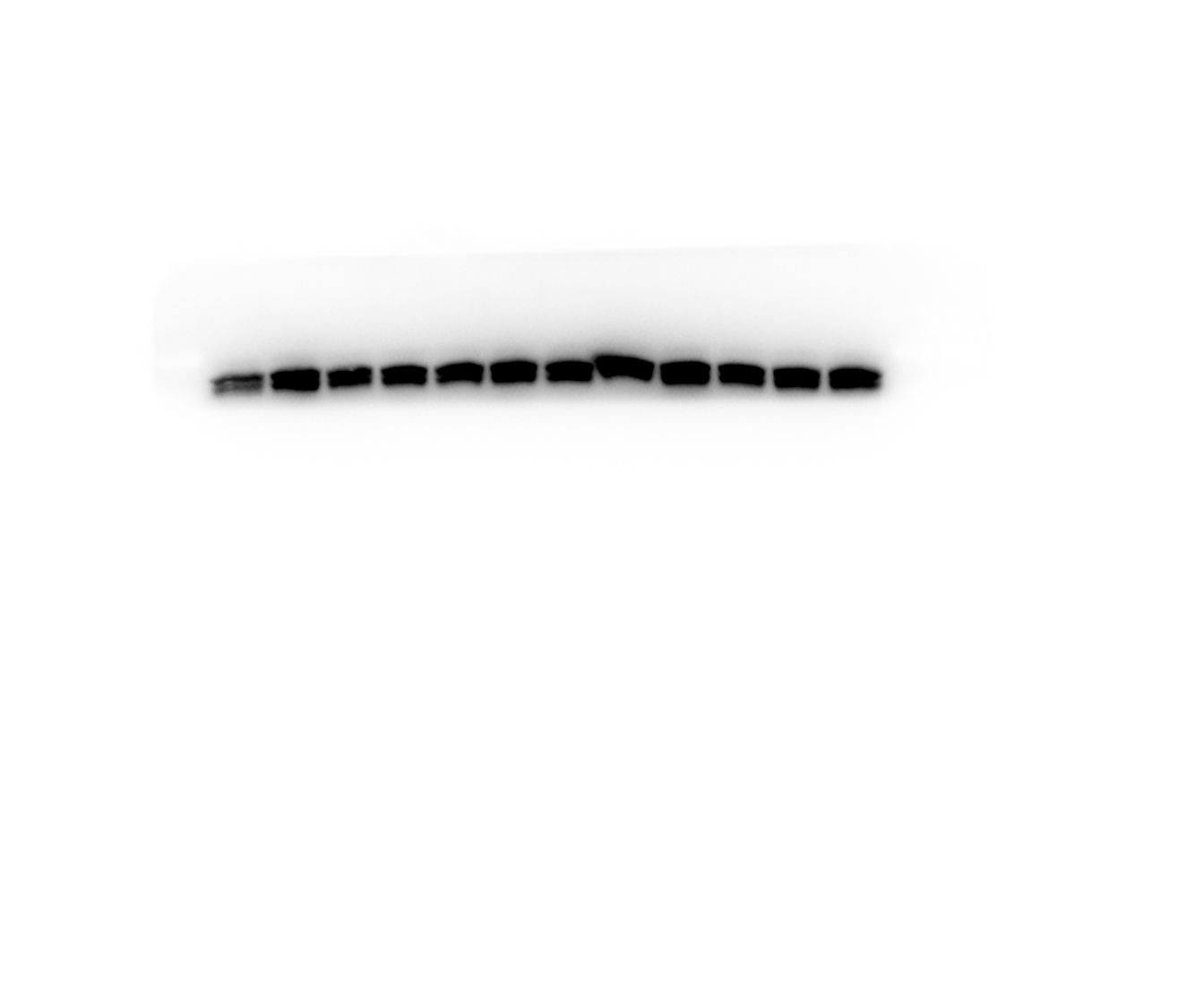

Supplement: S1 Dataset — (ZIP) [file ppat.1012800.s012.zip › Figs 1-9 minimal data set/fig 7/fig 7B/total AKT.Tif]

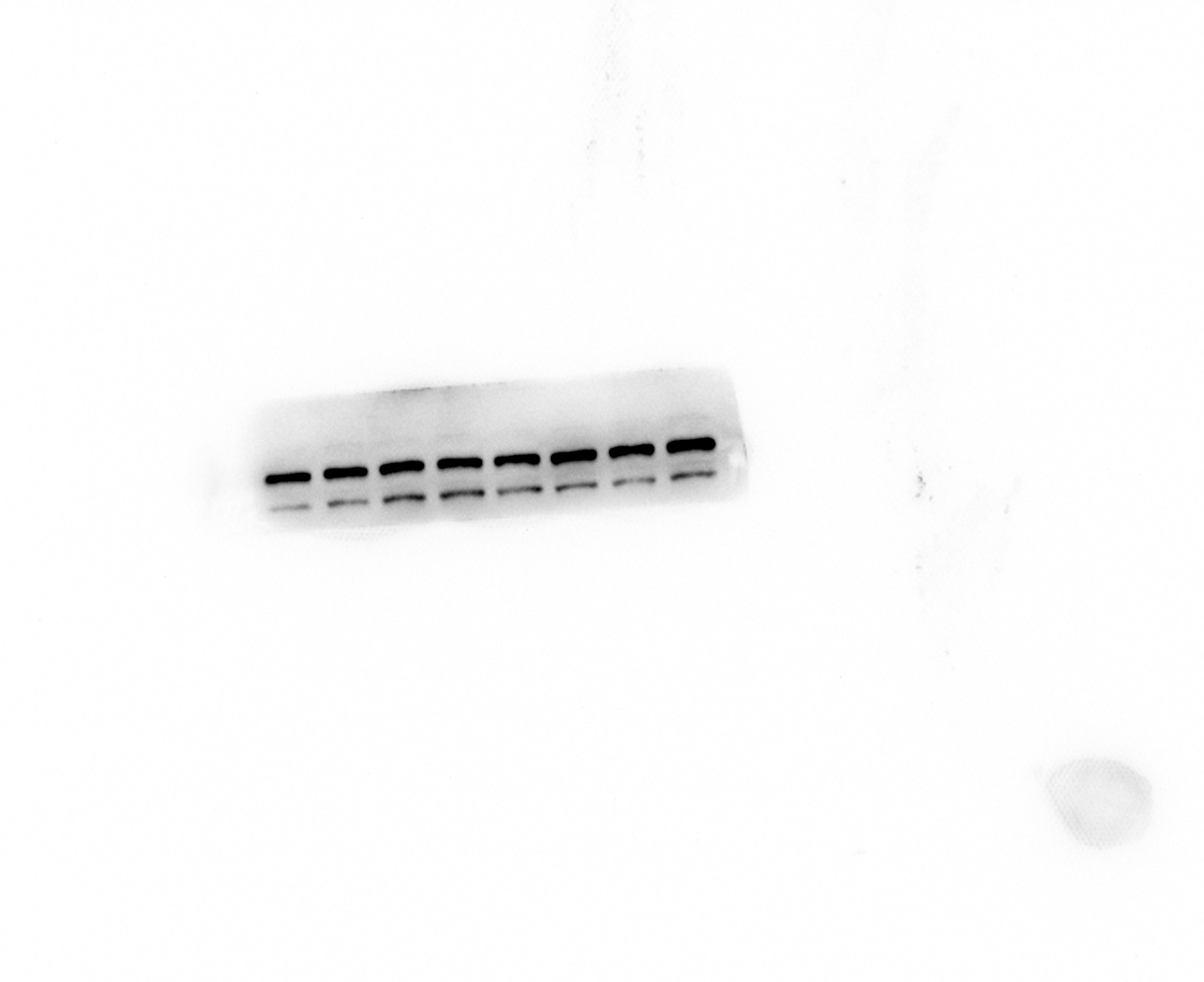

Supplement: S1 Dataset — (ZIP) [file ppat.1012800.s012.zip › Figs 1-9 minimal data set/fig 7/fig 7B/total S6K.Tif]

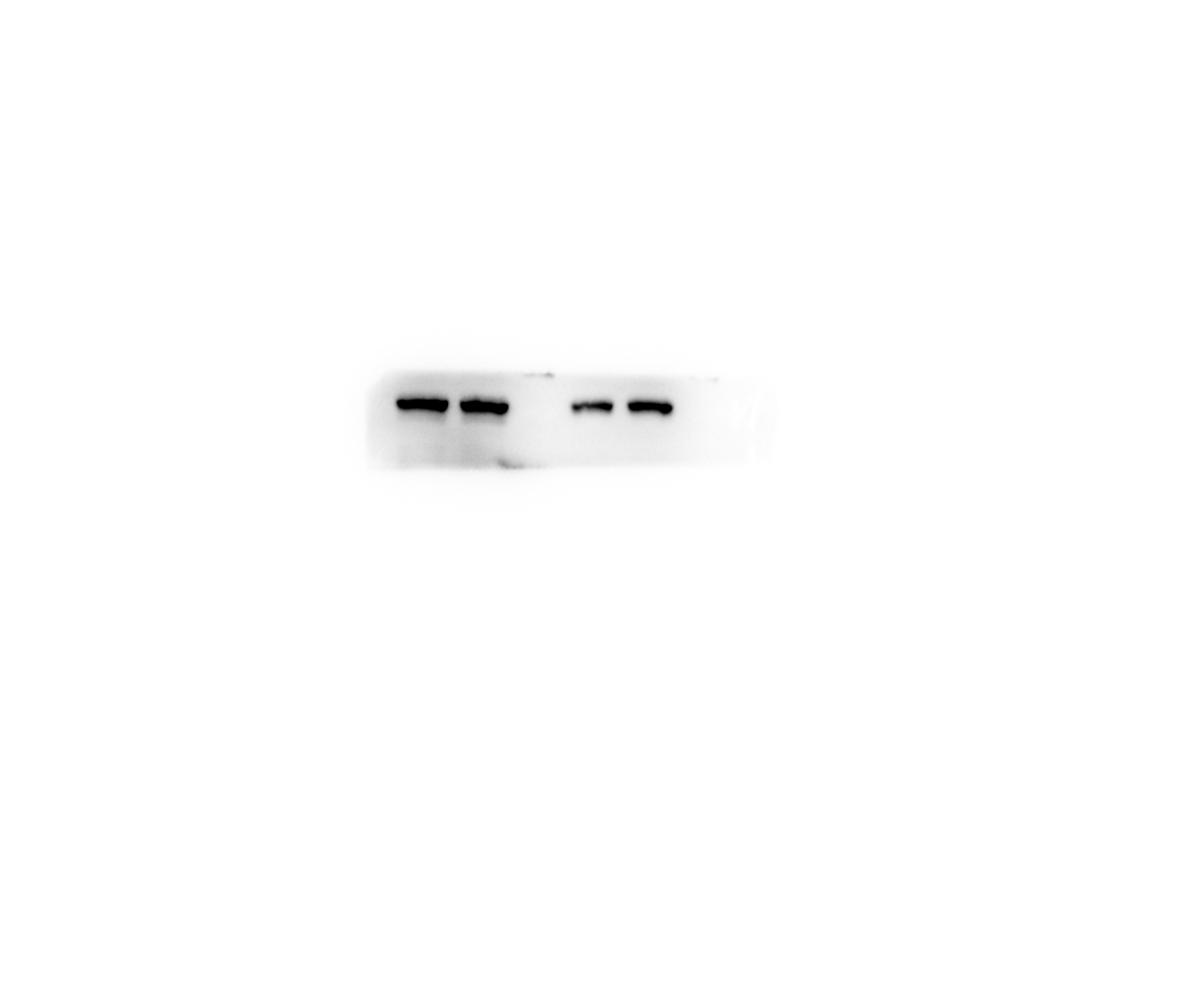

Supplement: S1 Dataset — (ZIP) [file ppat.1012800.s012.zip › Figs 1-9 minimal data set/fig 7/fig 7G/ATP6V1B2.Tif]

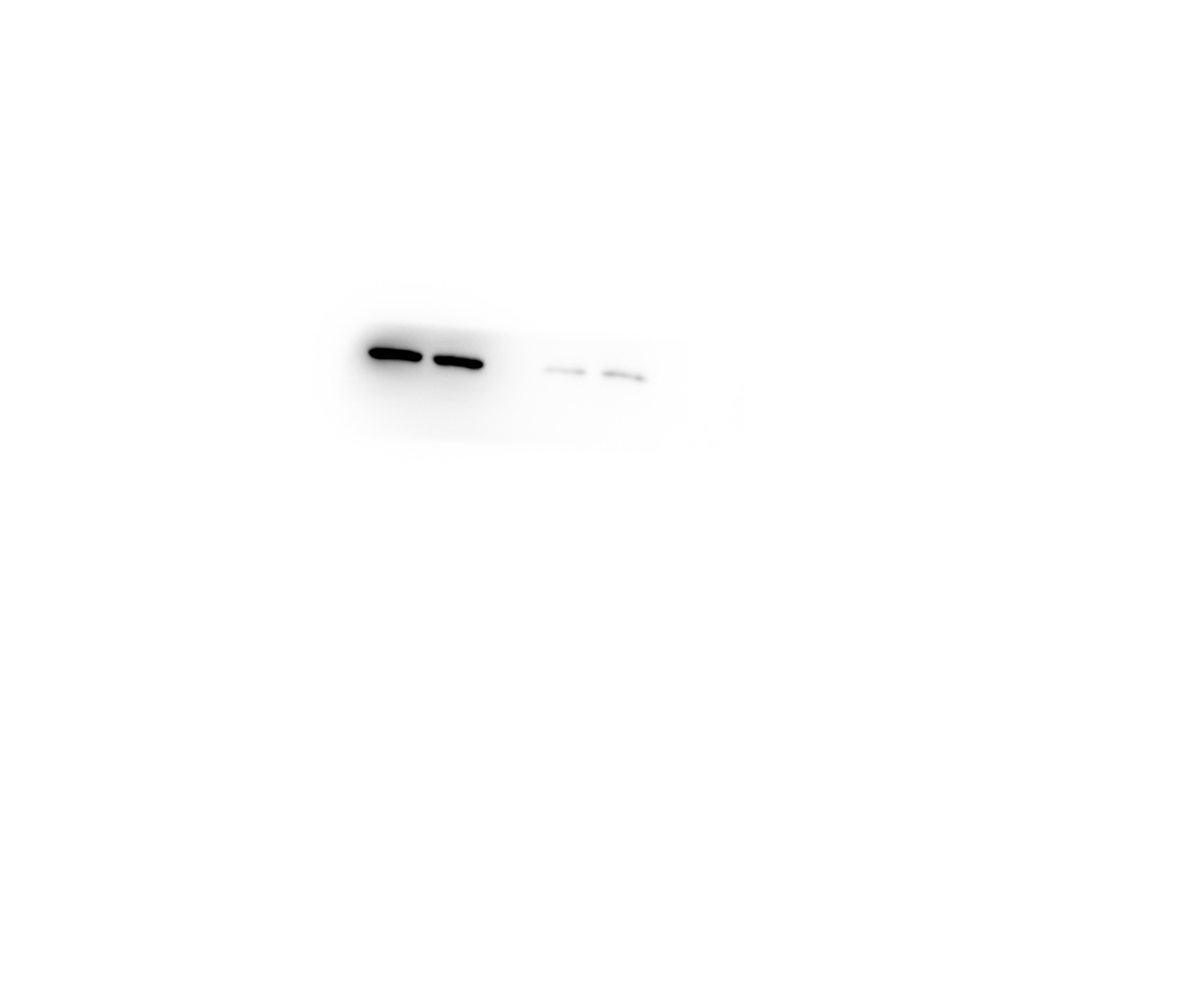

Supplement: S1 Dataset — (ZIP) [file ppat.1012800.s012.zip › Figs 1-9 minimal data set/fig 7/fig 7G/GAPDH.Tif]

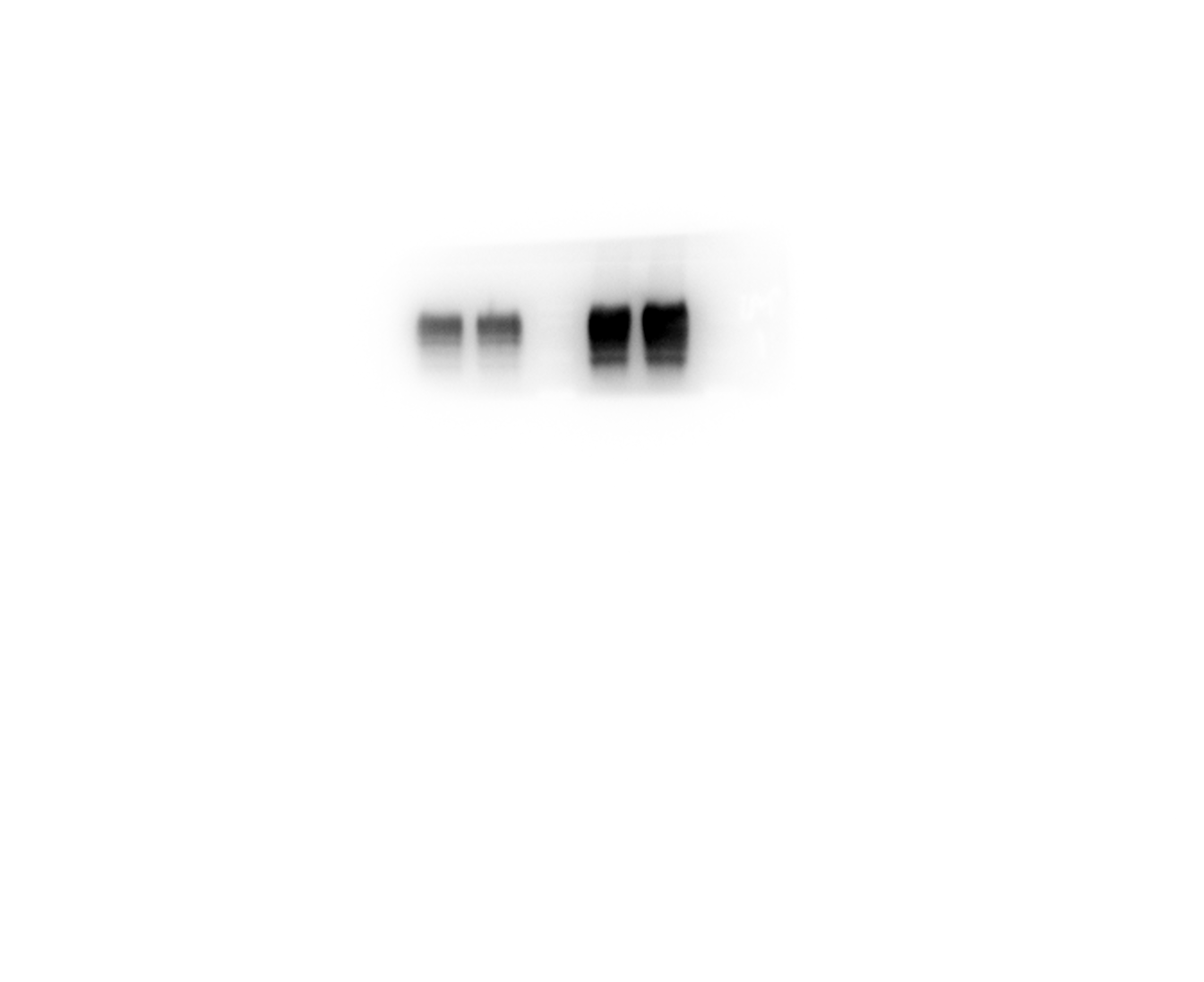

Supplement: S1 Dataset — (ZIP) [file ppat.1012800.s012.zip › Figs 1-9 minimal data set/fig 7/fig 7G/LAMP1.Tif]

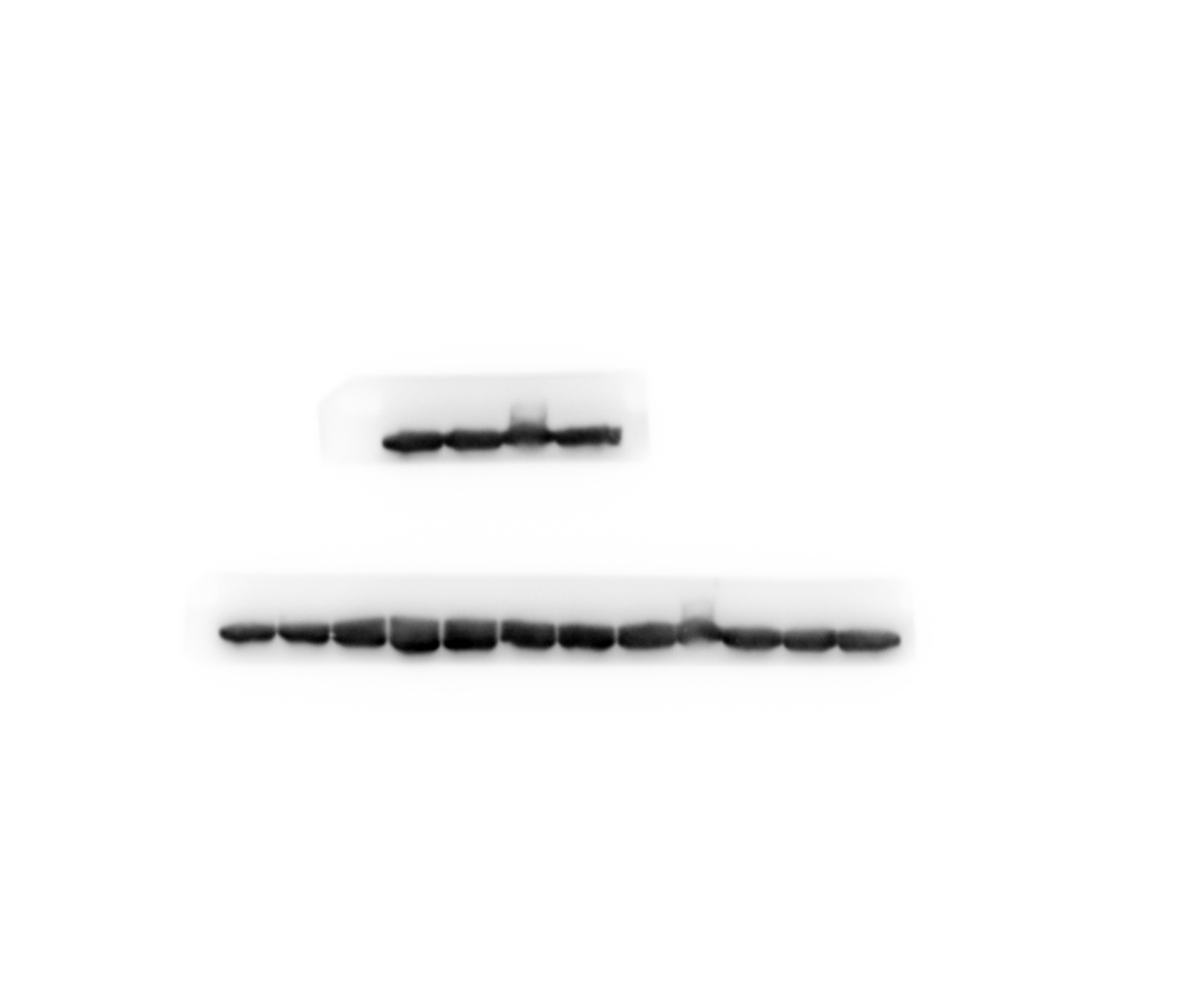

Supplement: S2 Dataset — (ZIP) [file ppat.1012800.s013.zip › 1-9 SFigs minimal data set/S1 fig/S1A fig/M-TUBULIN-3.Tif]

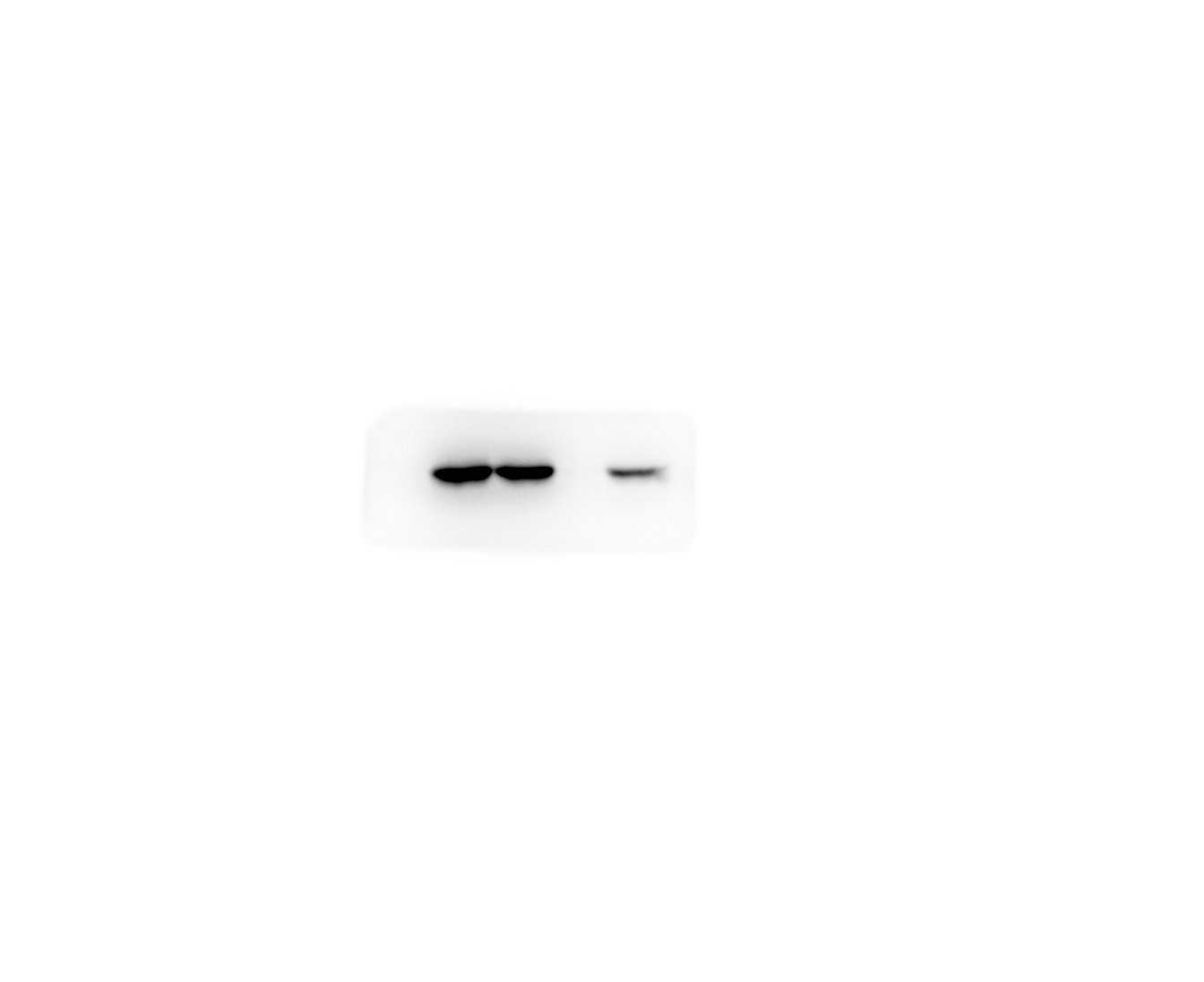

Supplement: S2 Dataset — (ZIP) [file ppat.1012800.s013.zip › 1-9 SFigs minimal data set/S1 fig/S1A fig/STING.Tif]

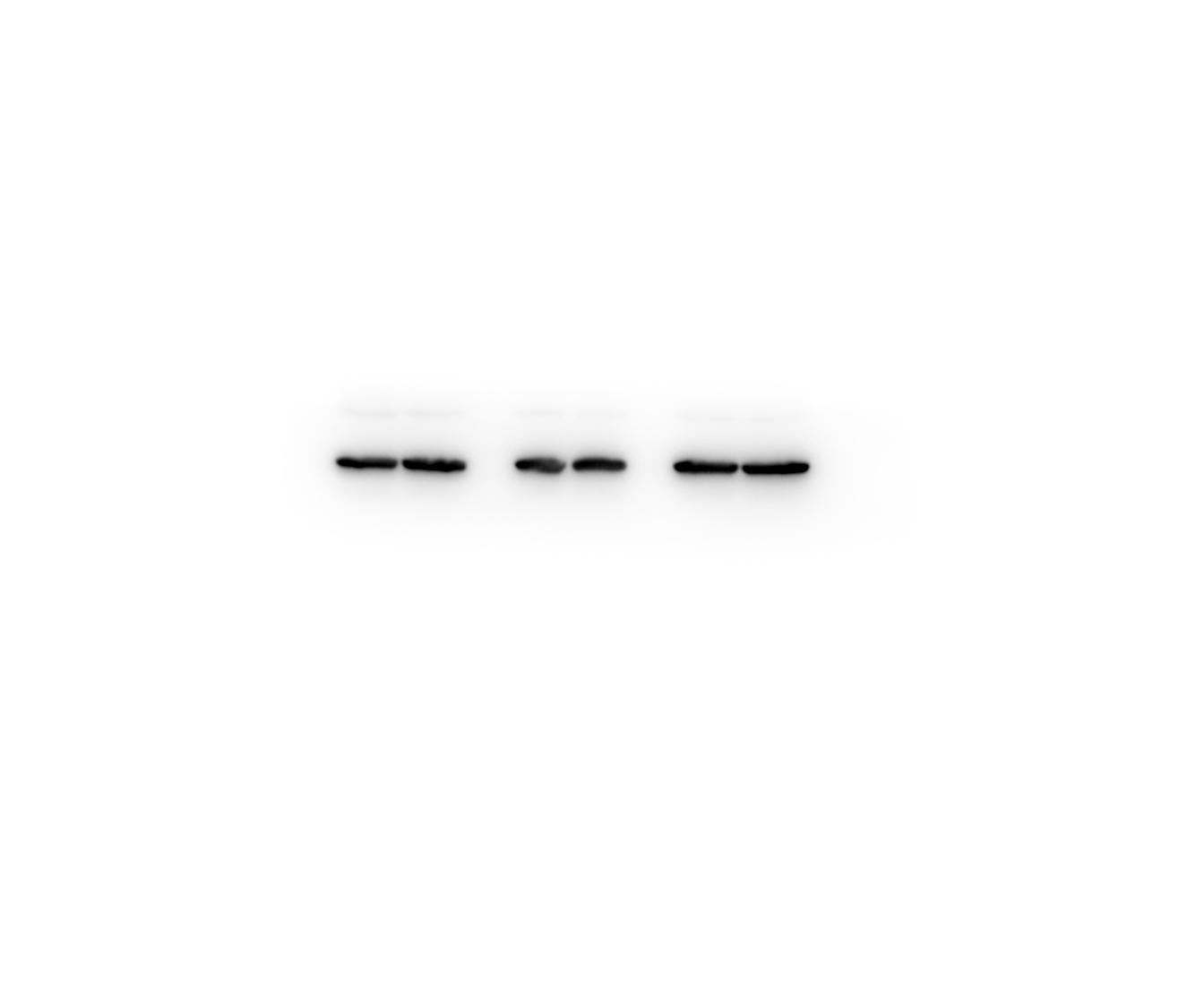

Supplement: S2 Dataset — (ZIP) [file ppat.1012800.s013.zip › 1-9 SFigs minimal data set/S2 fig/S2B fig/ACTIN.Tif]

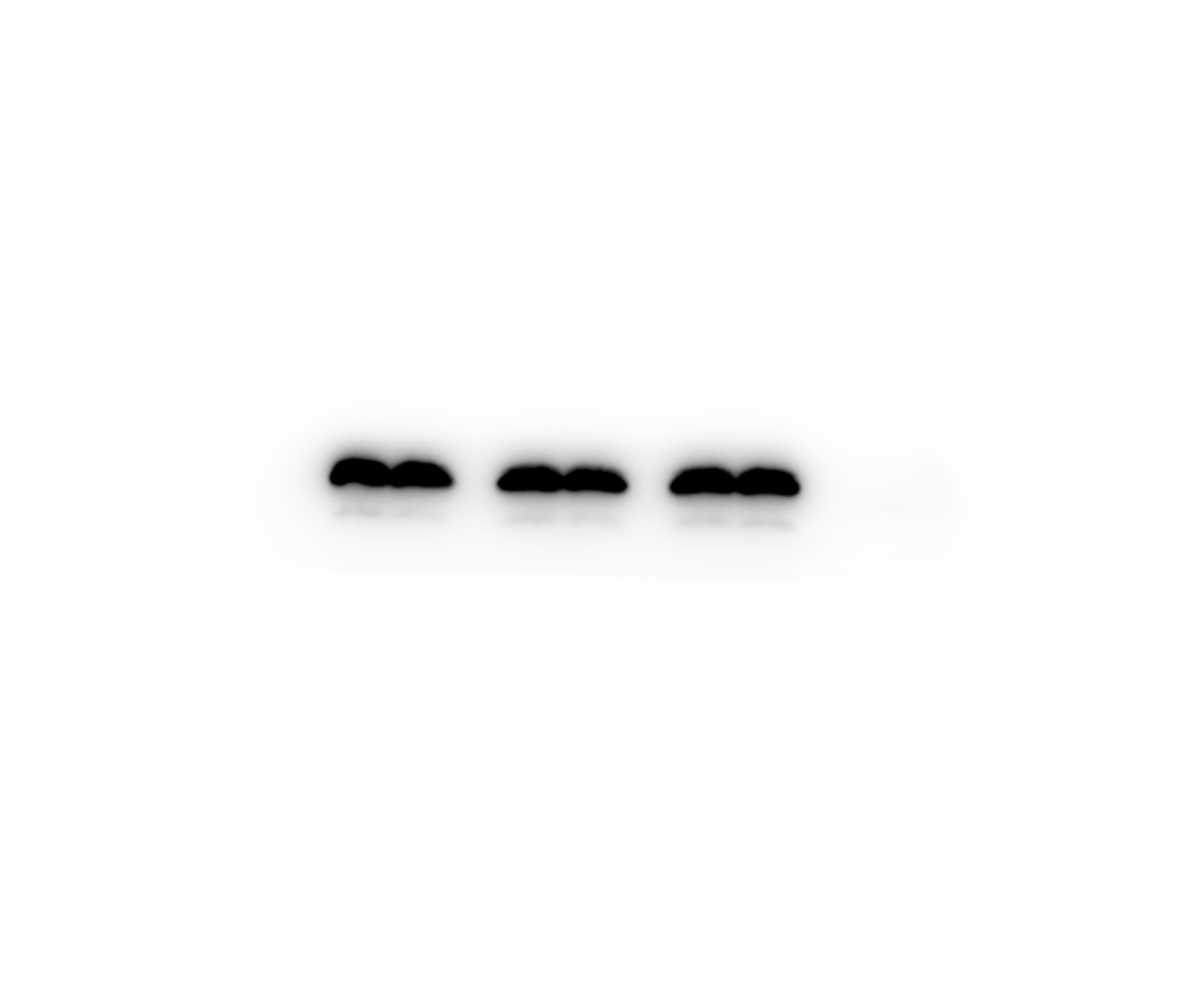

Supplement: S2 Dataset — (ZIP) [file ppat.1012800.s013.zip › 1-9 SFigs minimal data set/S2 fig/S2B fig/HBC.Tif]

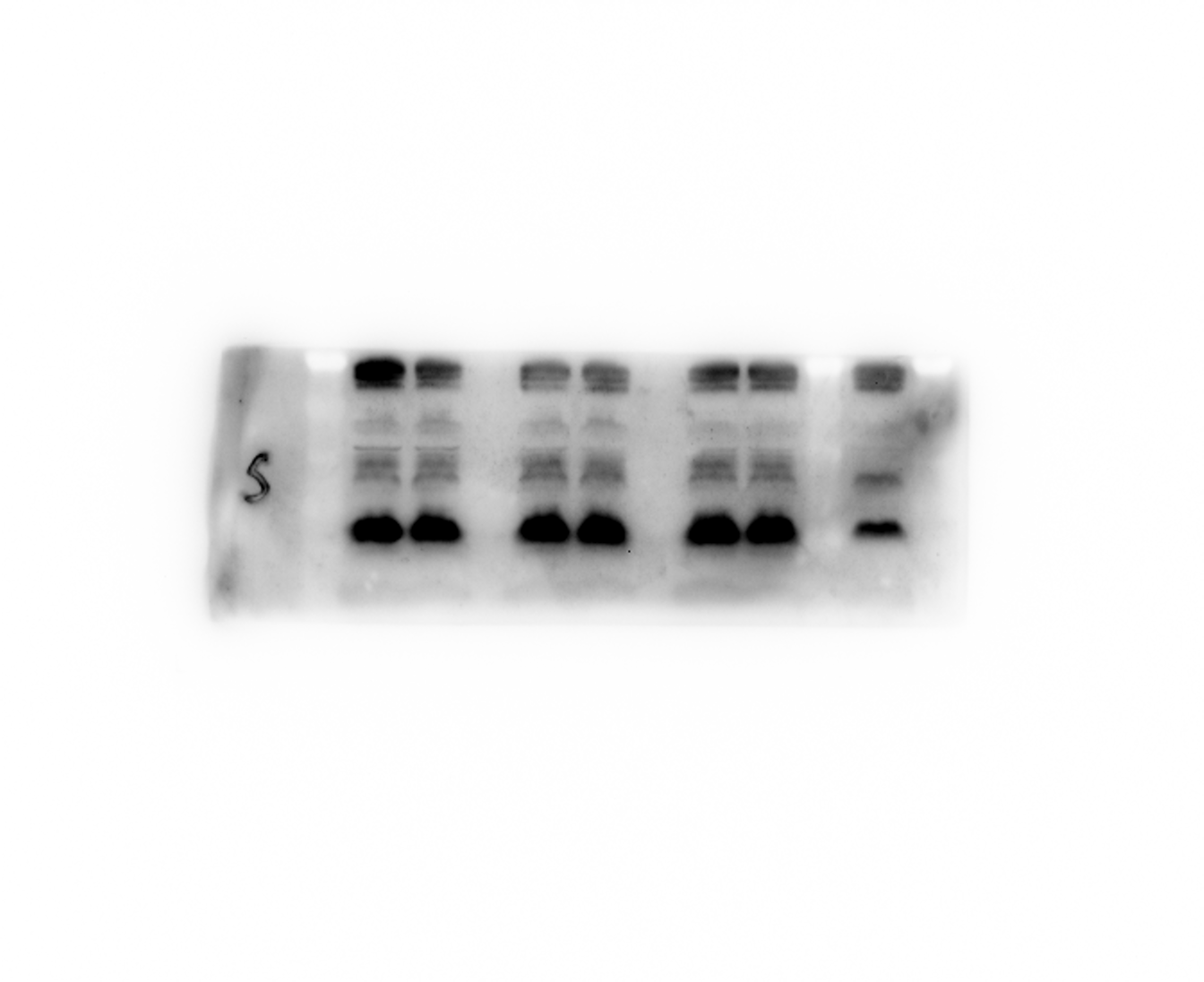

Supplement: S2 Dataset — (ZIP) [file ppat.1012800.s013.zip › 1-9 SFigs minimal data set/S2 fig/S2B fig/HBS.Tif]

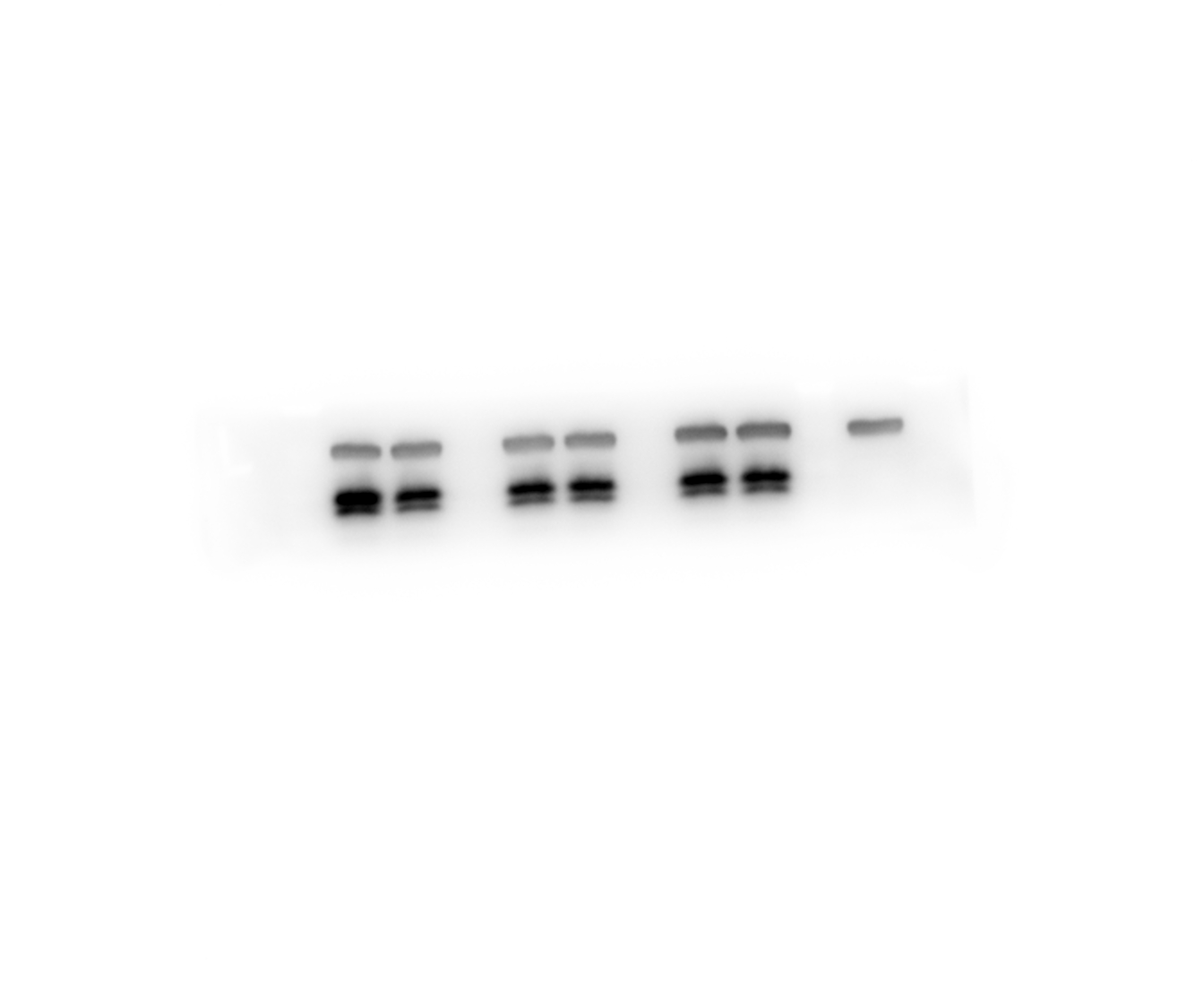

Supplement: S2 Dataset — (ZIP) [file ppat.1012800.s013.zip › 1-9 SFigs minimal data set/S2 fig/S2B fig/LHBS.Tif]

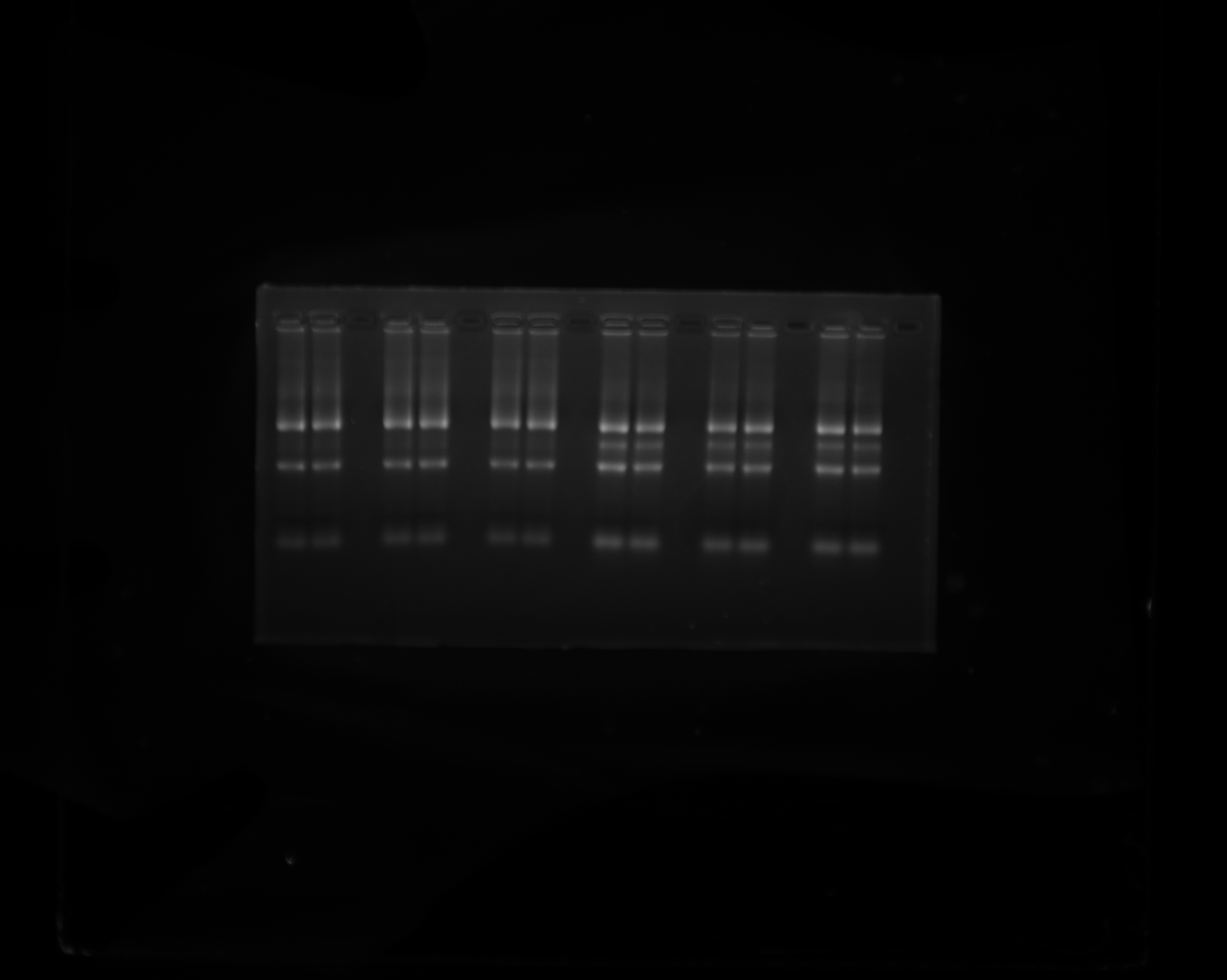

Supplement: S2 Dataset — (ZIP) [file ppat.1012800.s013.zip › 1-9 SFigs minimal data set/S2 fig/S2B fig/Northern blot, 18S 28S RNA.tif]

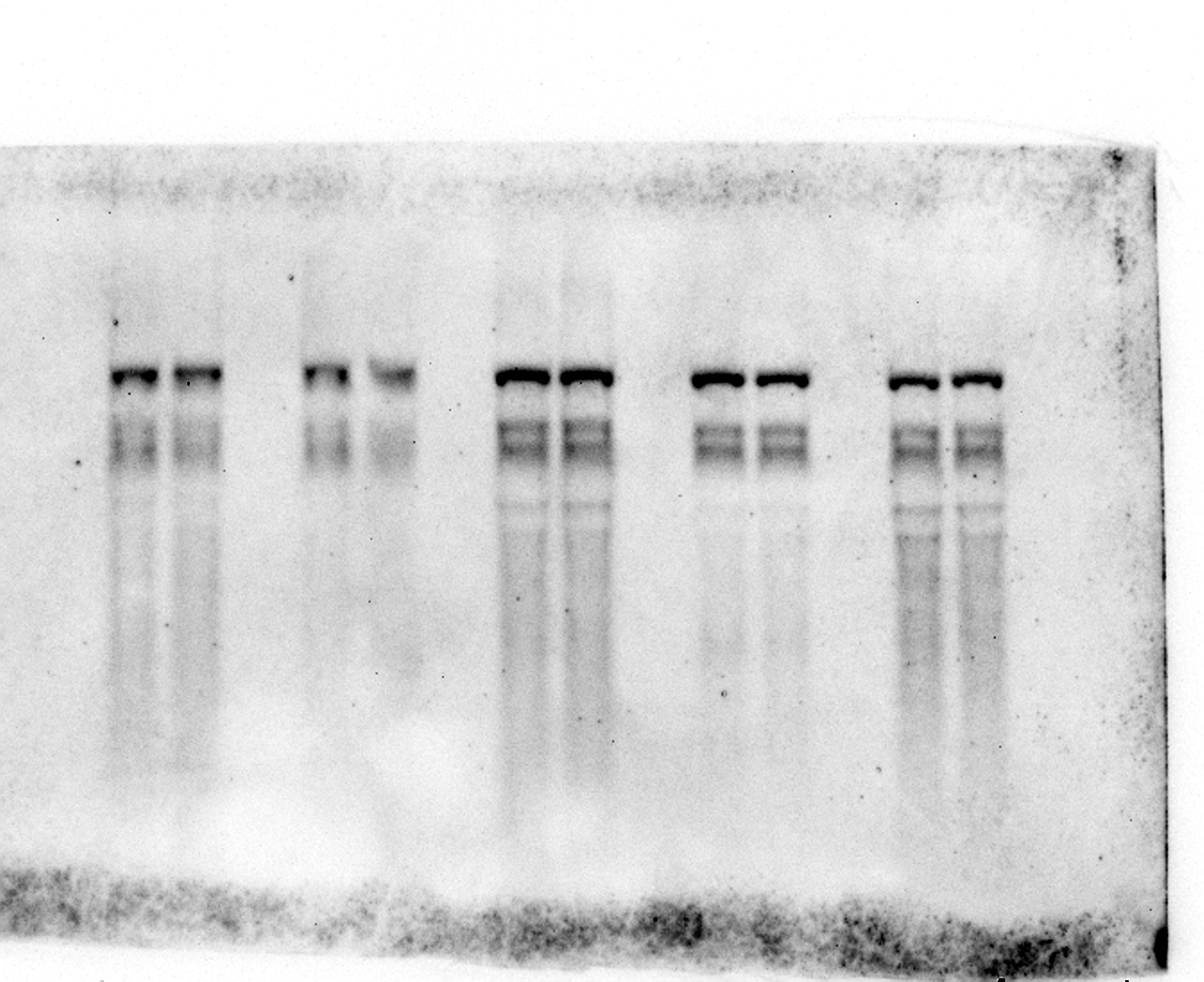

Supplement: S2 Dataset — (ZIP) [file ppat.1012800.s013.zip › 1-9 SFigs minimal data set/S2 fig/S2B fig/Northern blot.Tif]

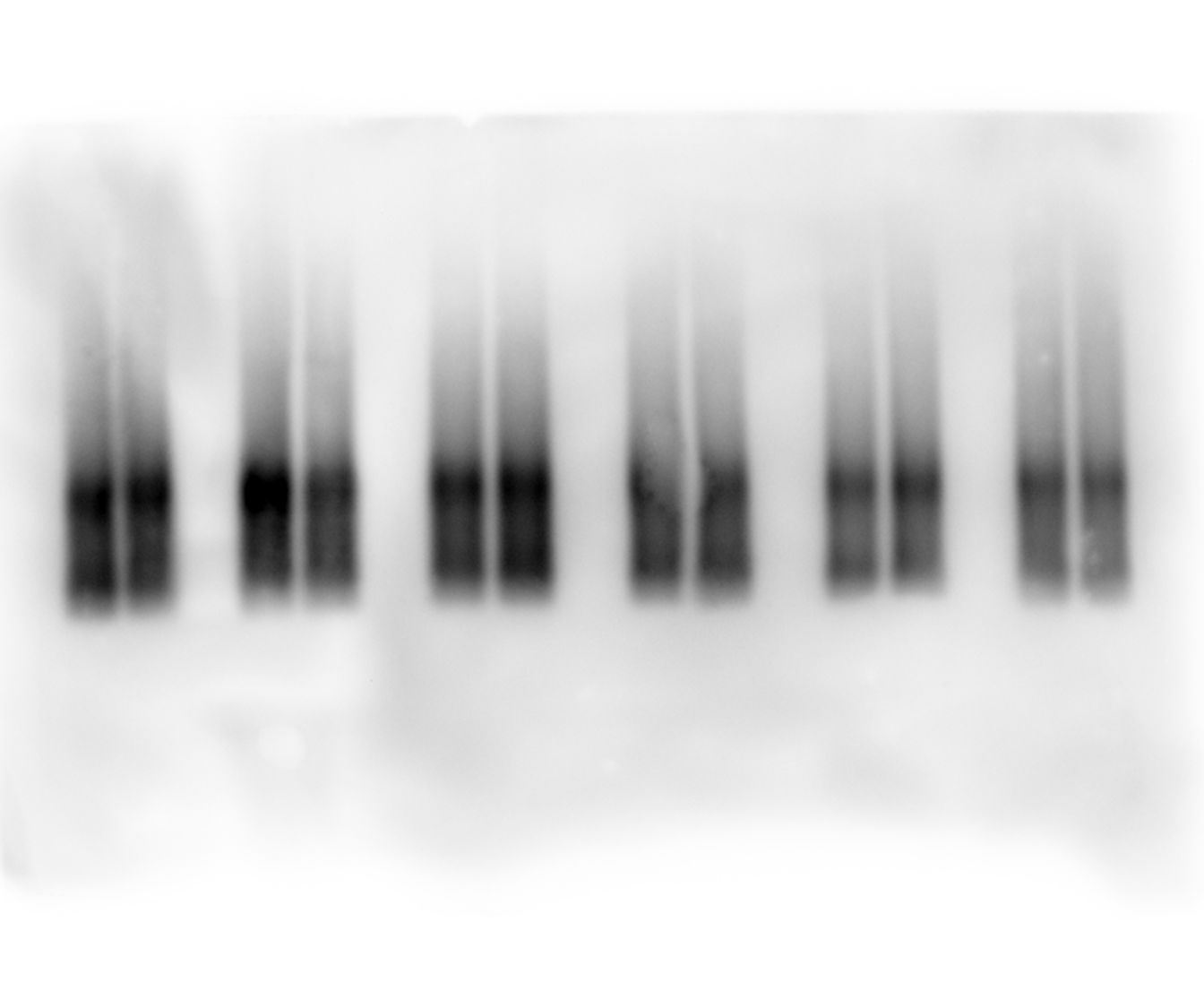

Supplement: S2 Dataset — (ZIP) [file ppat.1012800.s013.zip › 1-9 SFigs minimal data set/S2 fig/S2B fig/Particle gel.Tif]

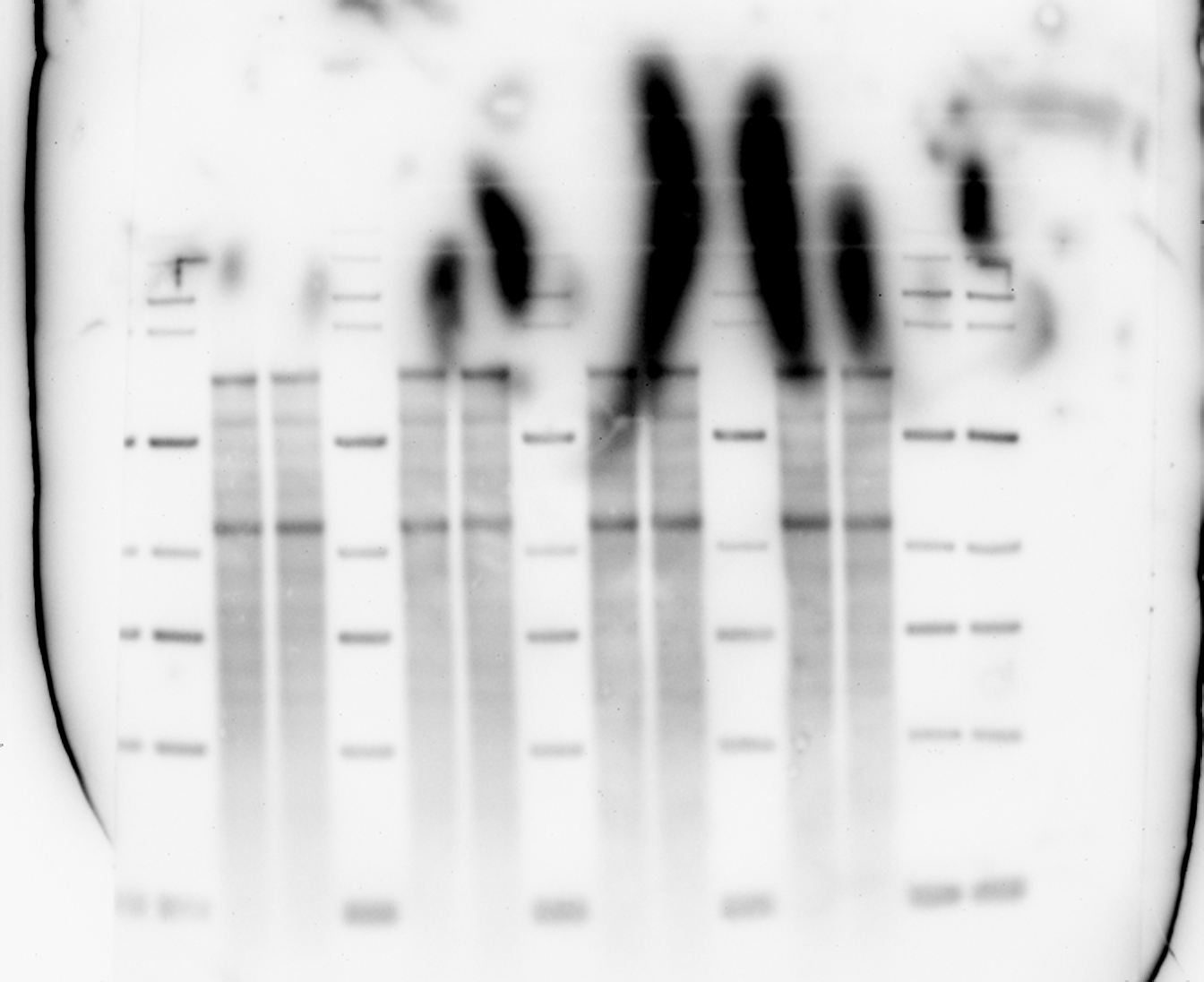

Supplement: S2 Dataset — (ZIP) [file ppat.1012800.s013.zip › 1-9 SFigs minimal data set/S2 fig/S2B fig/Southern blot.Tif]

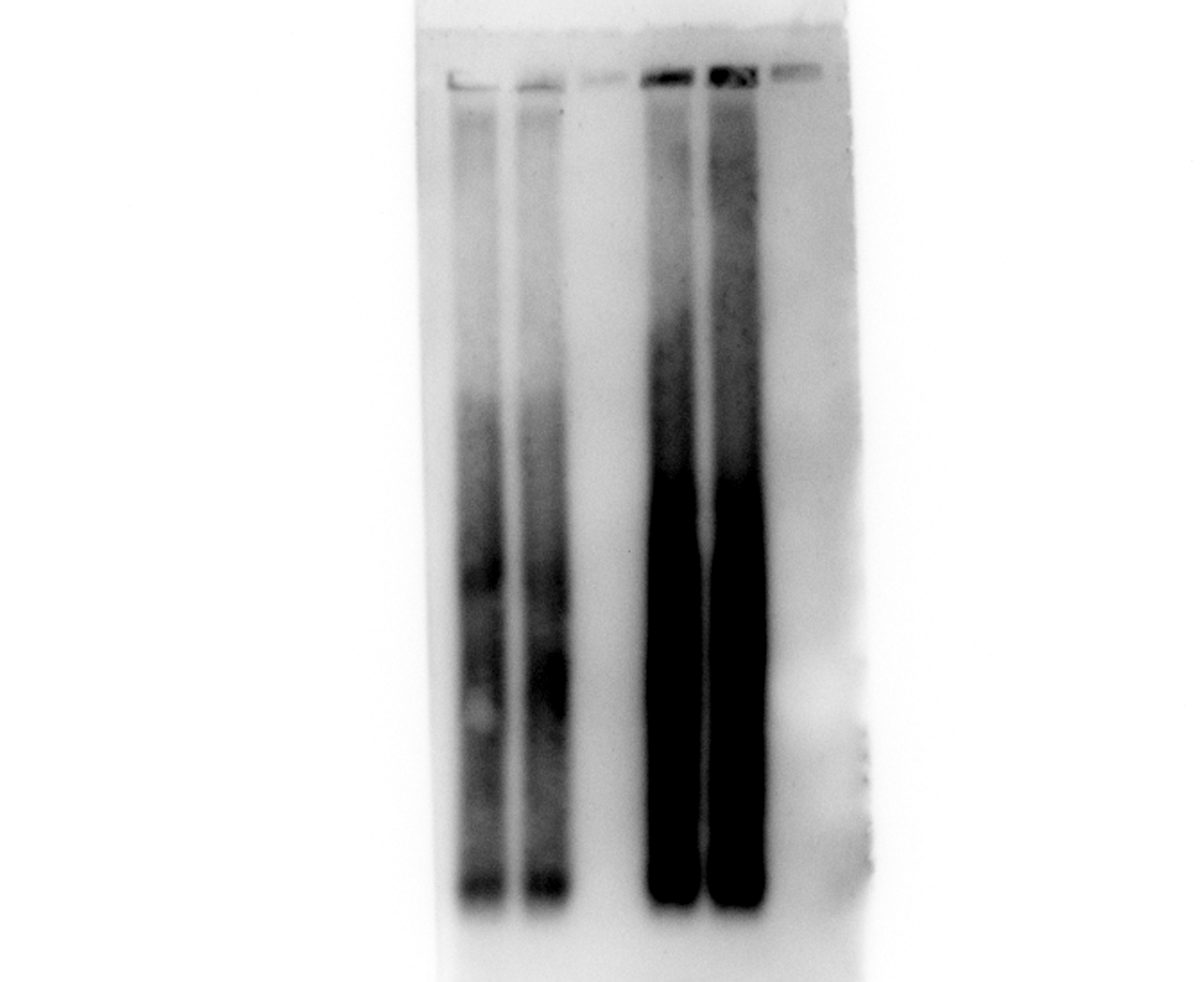

Supplement: S2 Dataset — (ZIP) [file ppat.1012800.s013.zip › 1-9 SFigs minimal data set/S2 fig/S2E fig/HBc, left.Tif]

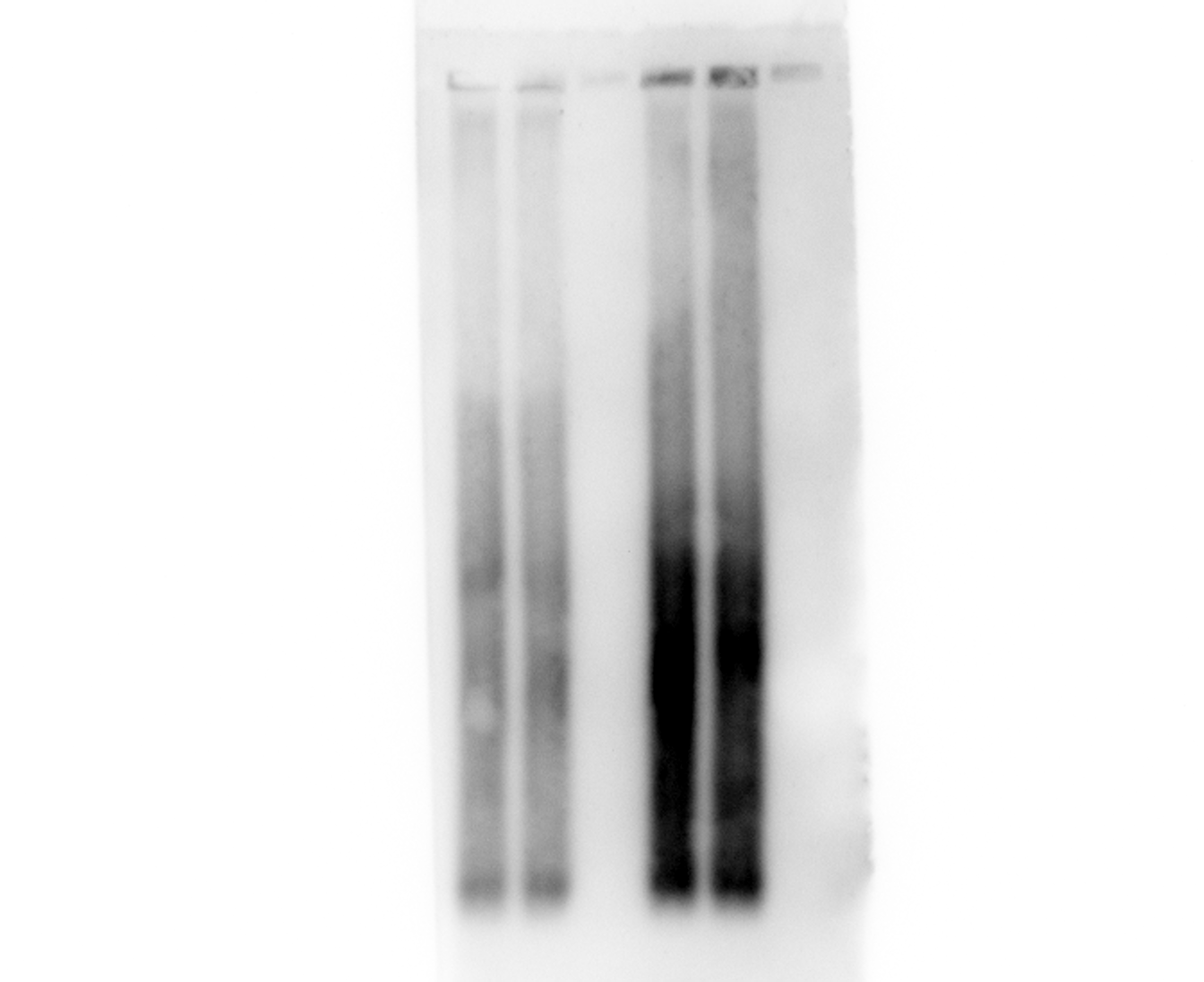

Supplement: S2 Dataset — (ZIP) [file ppat.1012800.s013.zip › 1-9 SFigs minimal data set/S2 fig/S2E fig/HBc, right.Tif]

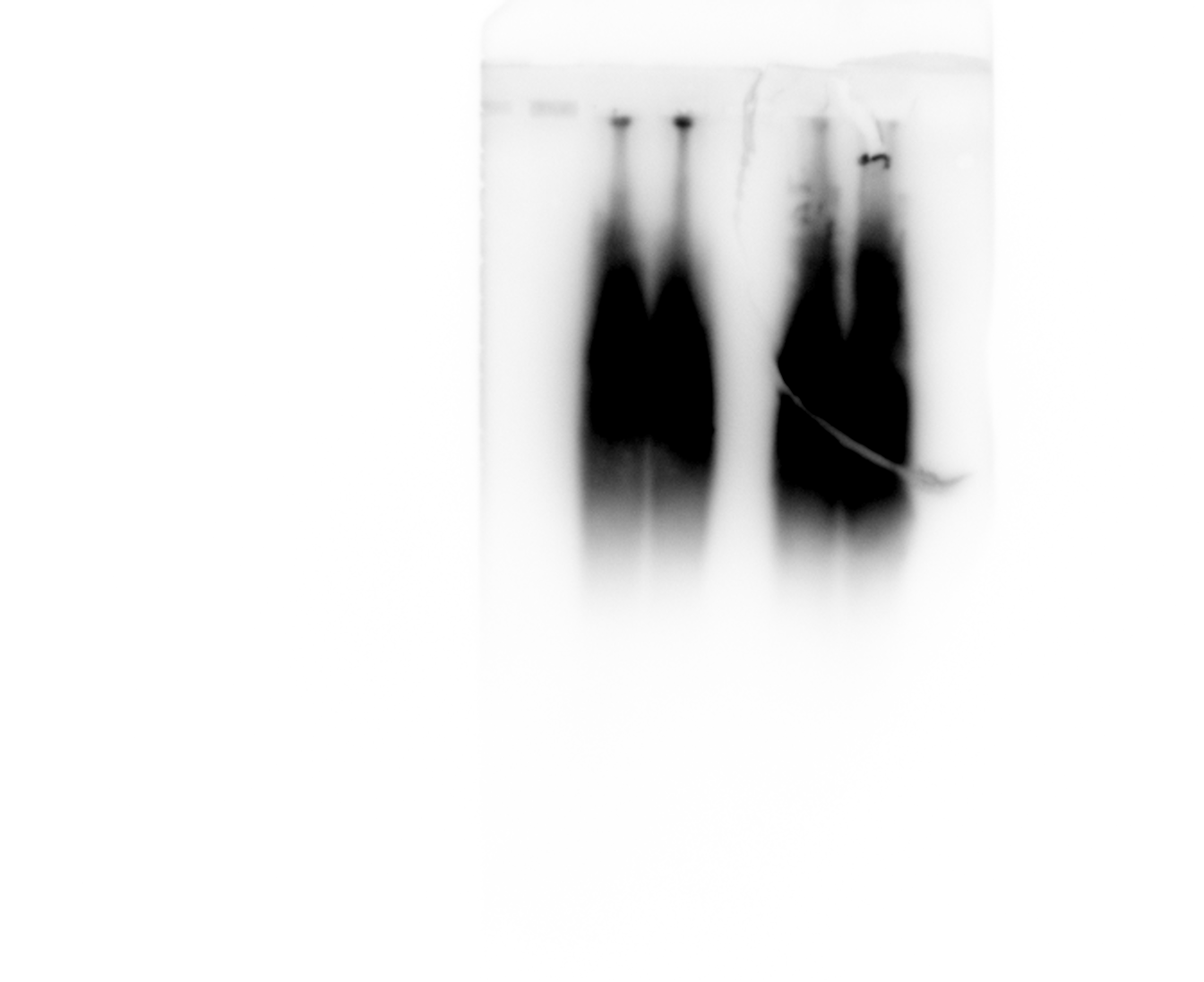

Supplement: S2 Dataset — (ZIP) [file ppat.1012800.s013.zip › 1-9 SFigs minimal data set/S2 fig/S2E fig/HBs, left.Tif]

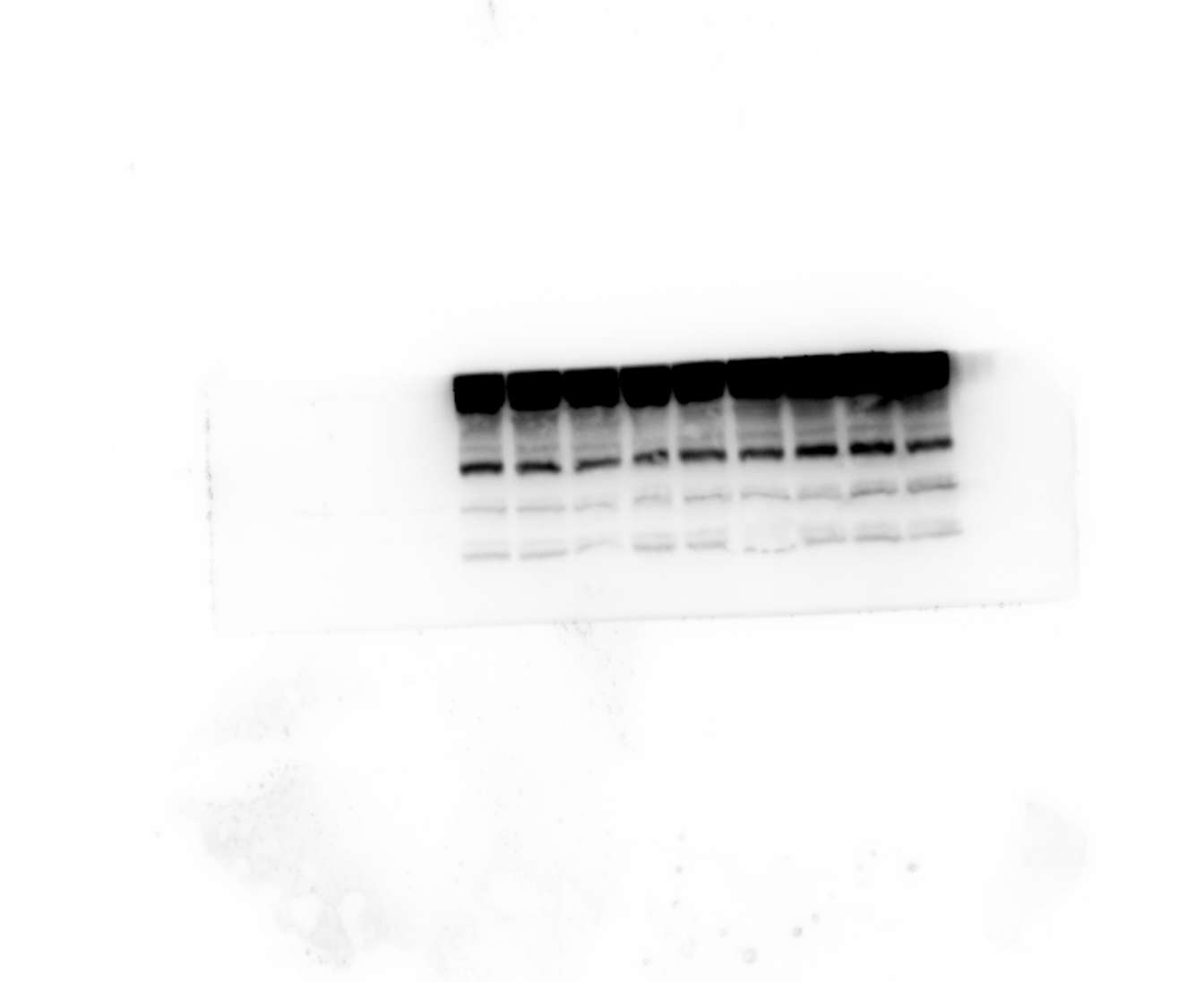

Supplement: S2 Dataset — (ZIP) [file ppat.1012800.s013.zip › 1-9 SFigs minimal data set/S3 fig/S3A fig NTCP.Tif]

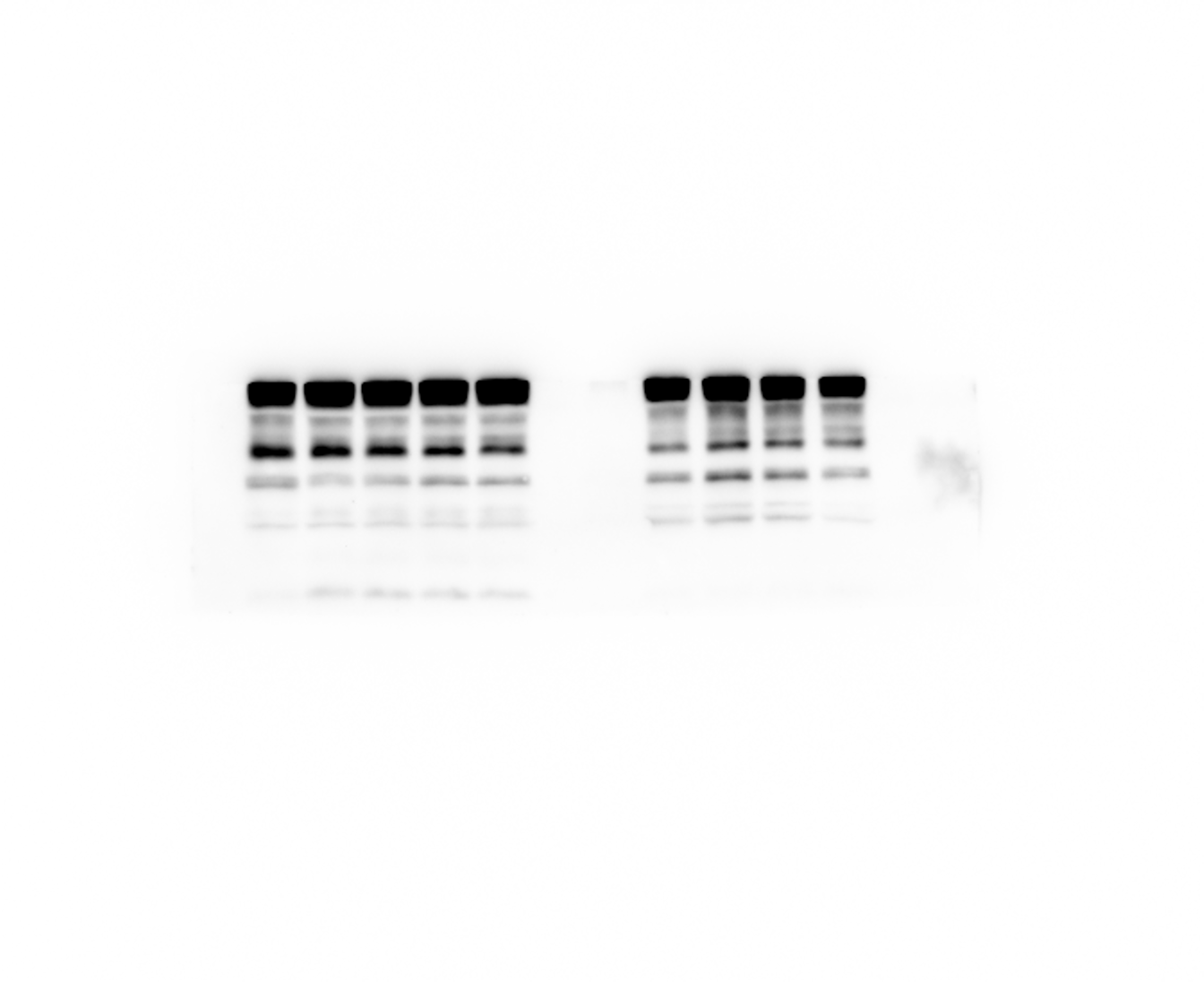

Supplement: S2 Dataset — (ZIP) [file ppat.1012800.s013.zip › 1-9 SFigs minimal data set/S3 fig/S3B fig NTCP.Tif]

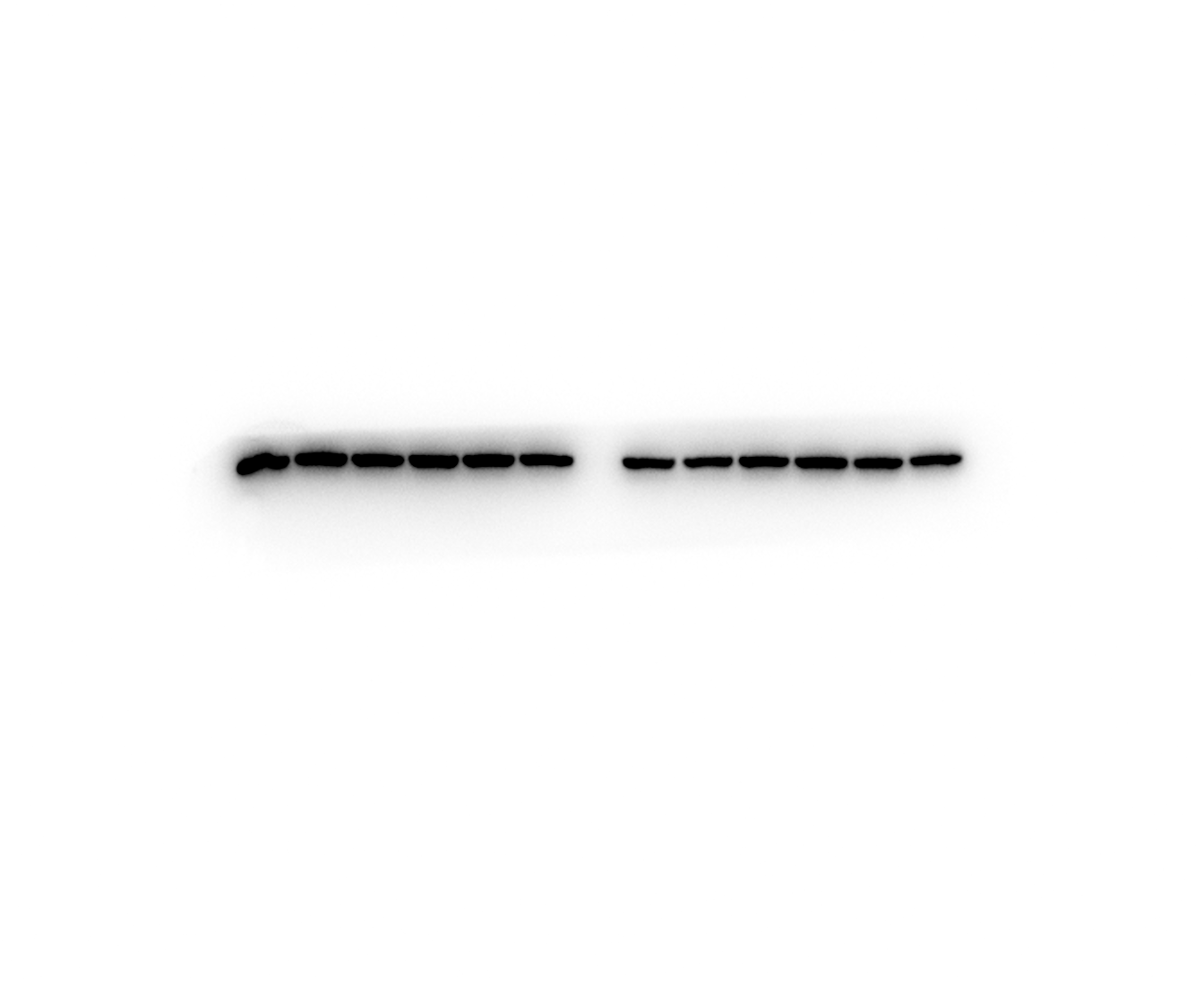

Supplement: S2 Dataset — (ZIP) [file ppat.1012800.s013.zip › 1-9 SFigs minimal data set/S4 fig/S4A fig/ACTB.Tif]

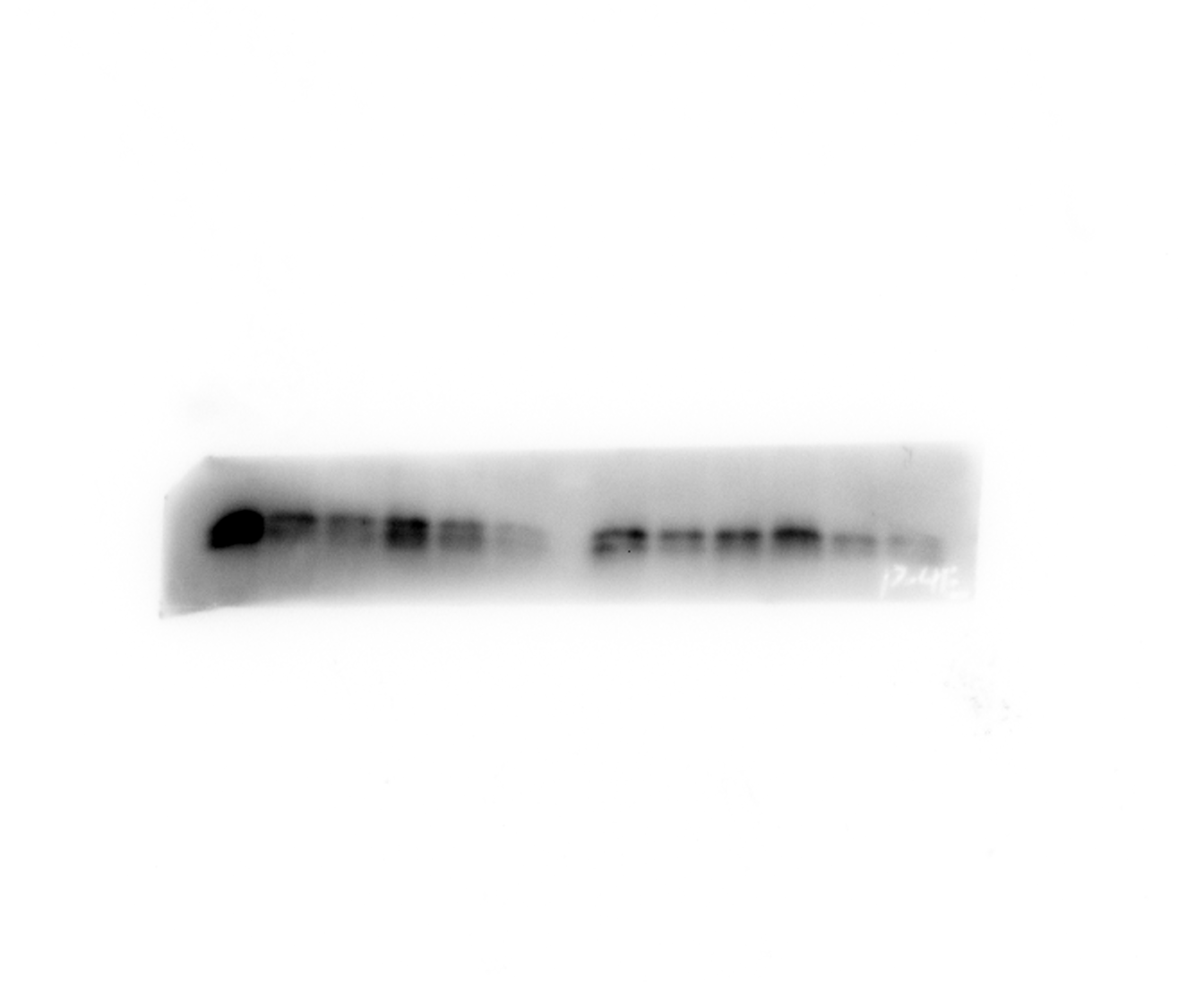

Supplement: S2 Dataset — (ZIP) [file ppat.1012800.s013.zip › 1-9 SFigs minimal data set/S4 fig/S4A fig/P-4E-BP1.Tif]

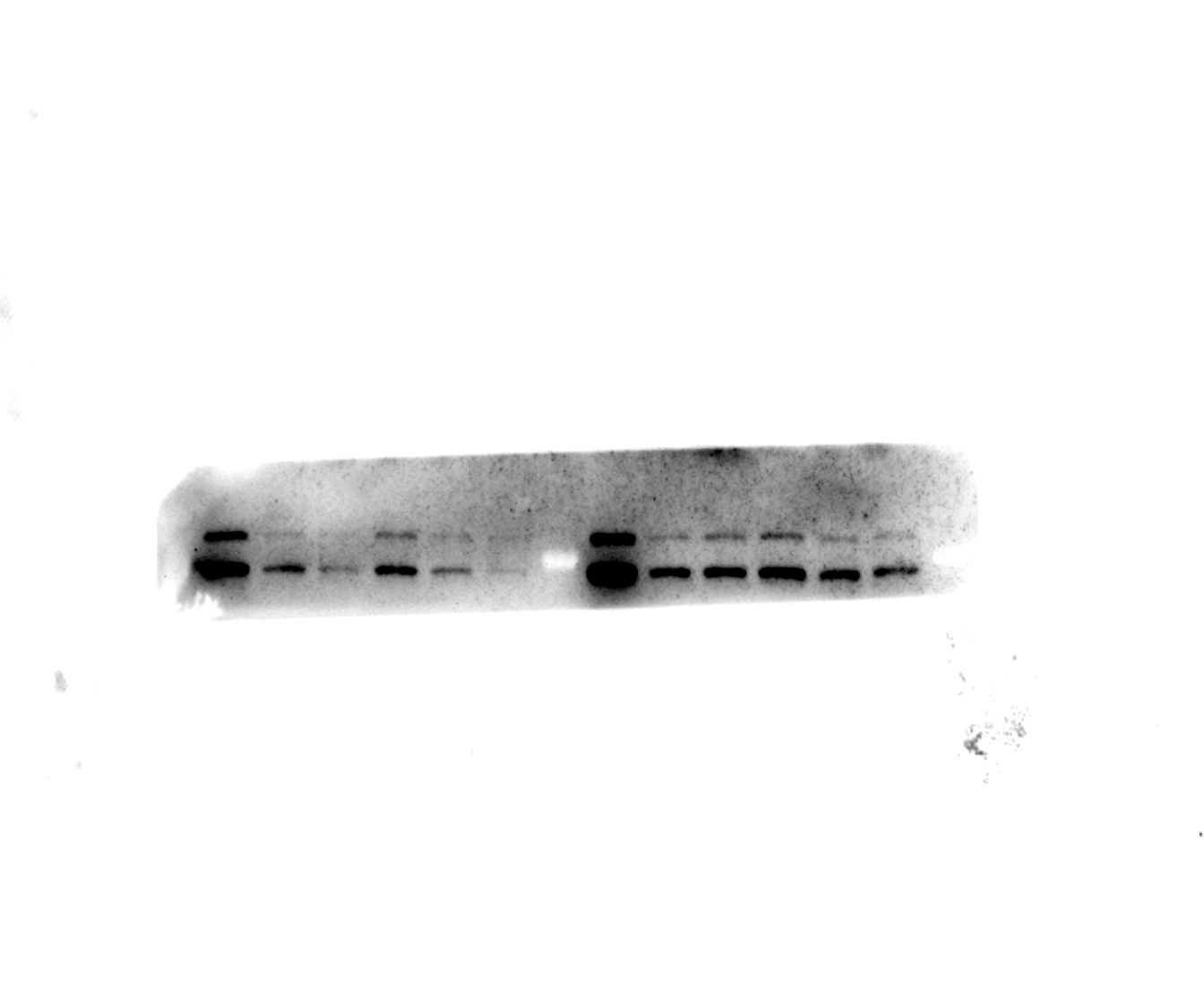

Supplement: S2 Dataset — (ZIP) [file ppat.1012800.s013.zip › 1-9 SFigs minimal data set/S4 fig/S4A fig/P-S6K.Tif]

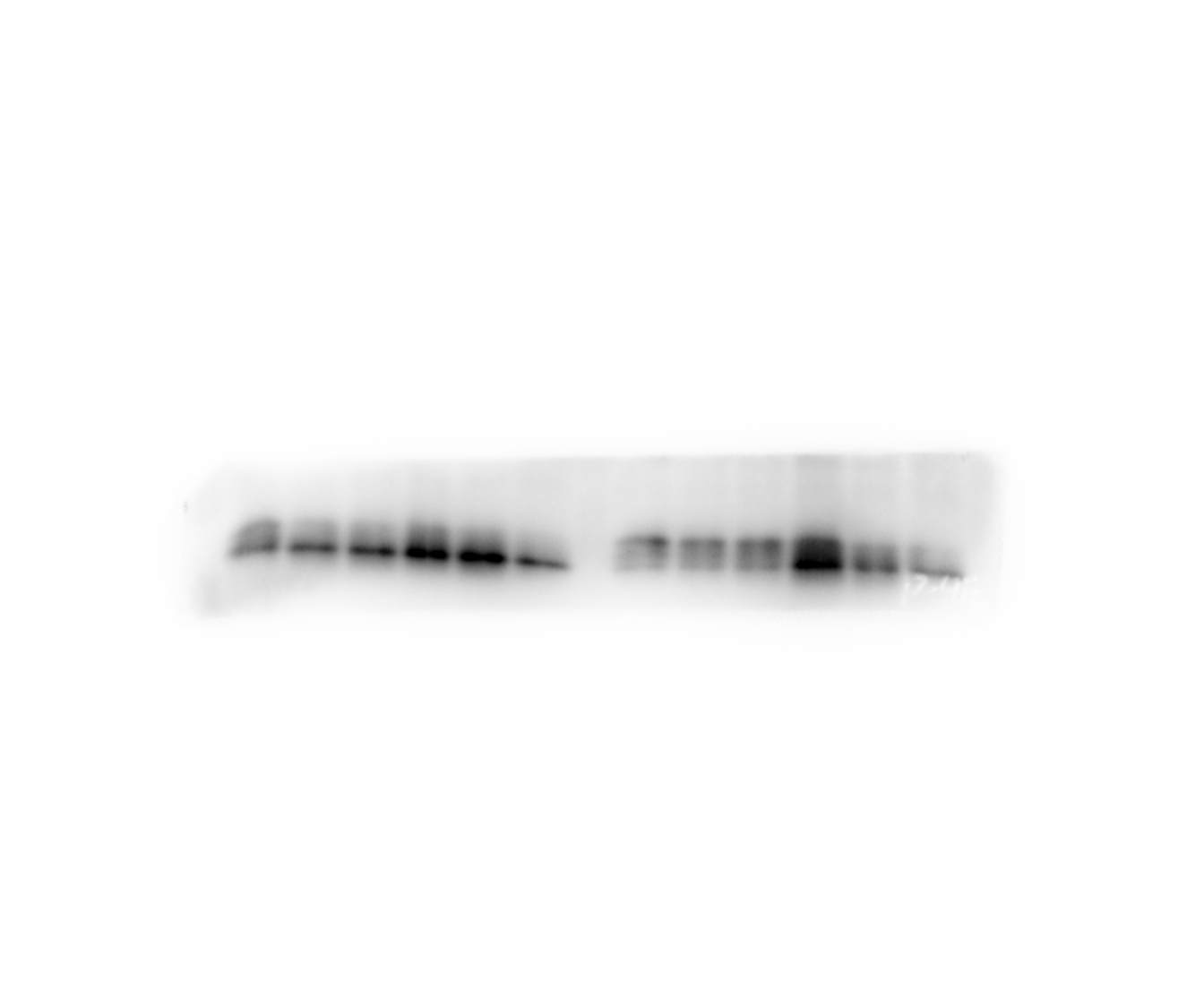

Supplement: S2 Dataset — (ZIP) [file ppat.1012800.s013.zip › 1-9 SFigs minimal data set/S4 fig/S4A fig/total 4E-BP1.Tif]

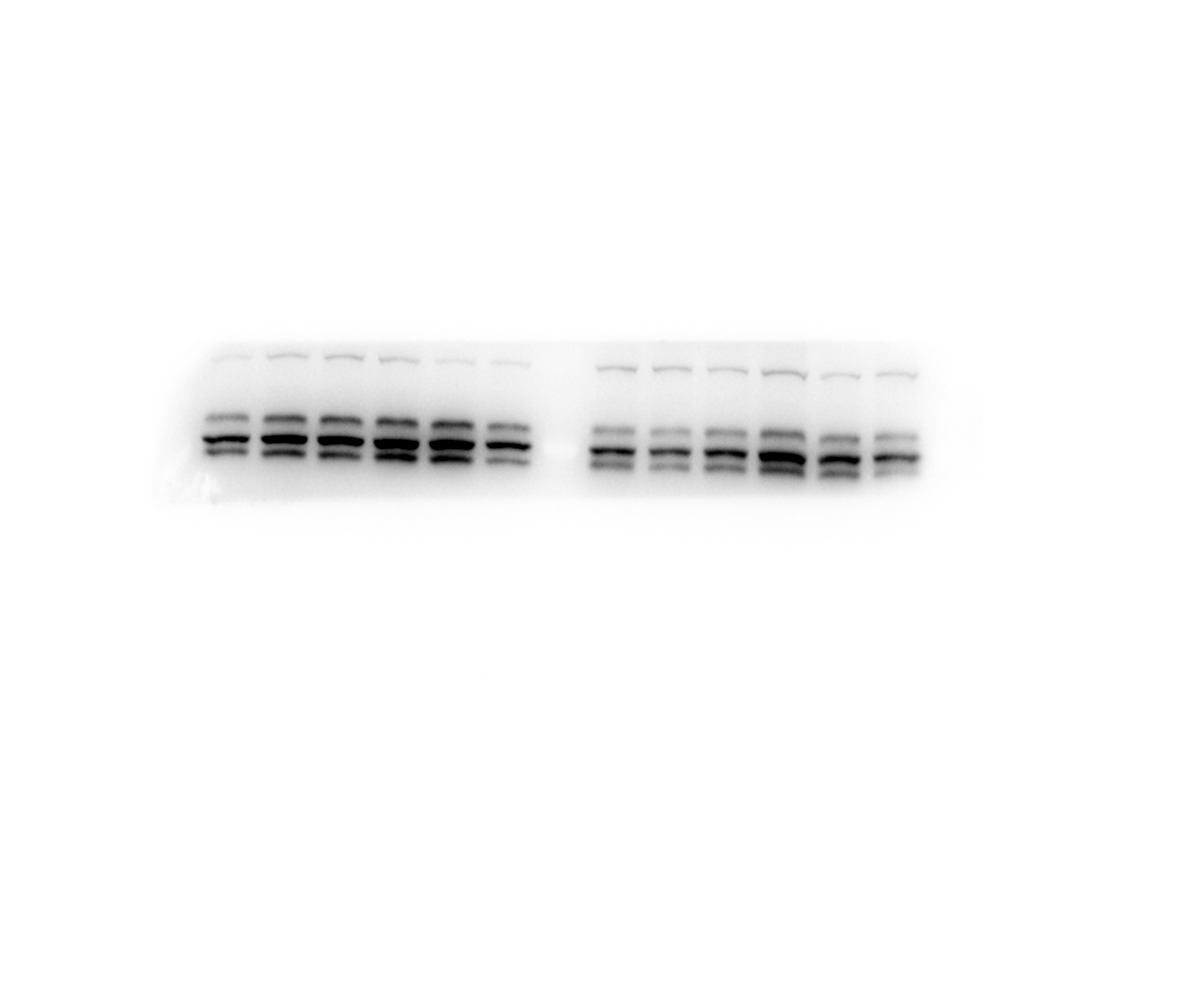

Supplement: S2 Dataset — (ZIP) [file ppat.1012800.s013.zip › 1-9 SFigs minimal data set/S4 fig/S4A fig/total S6K.Tif]

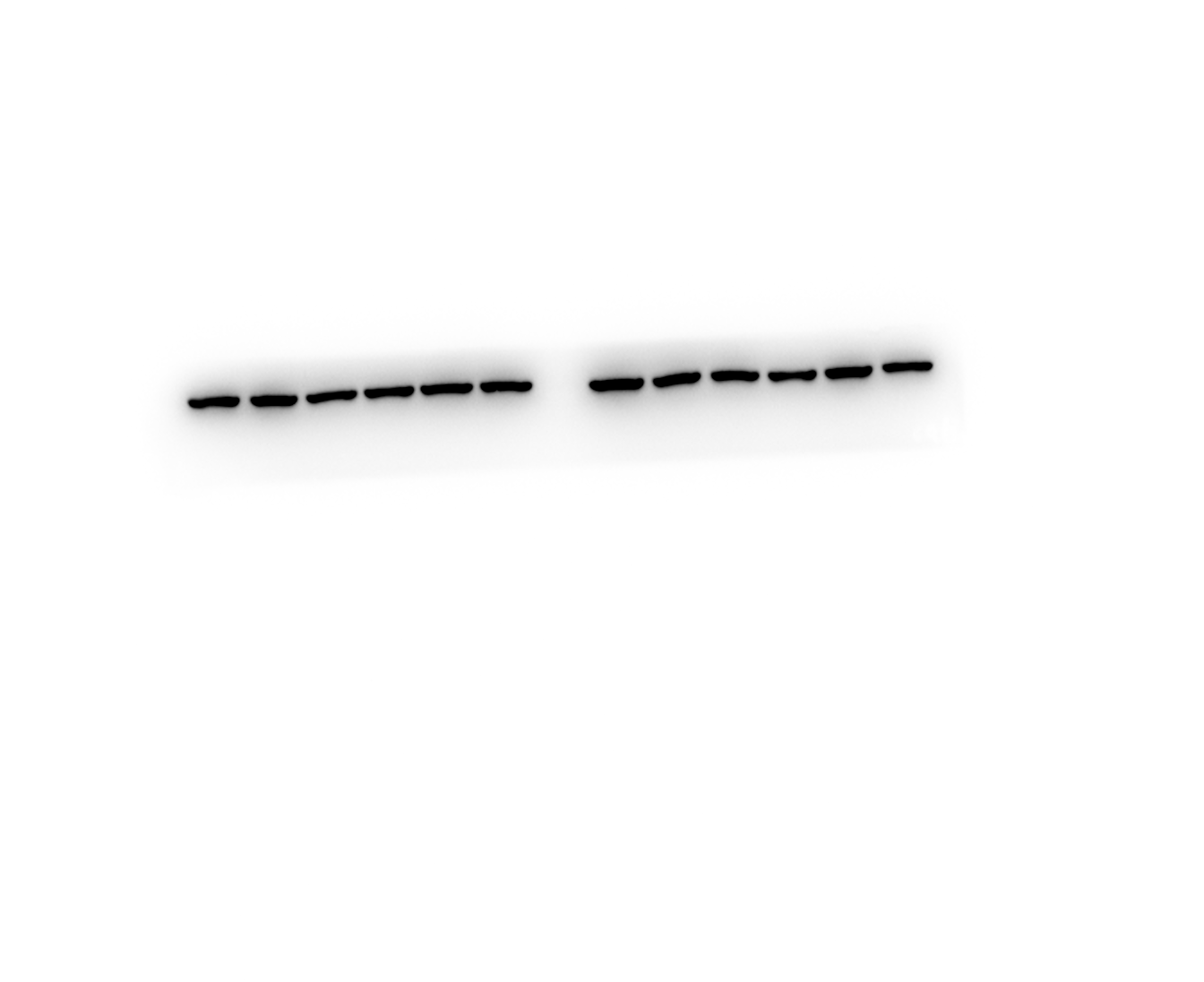

Supplement: S2 Dataset — (ZIP) [file ppat.1012800.s013.zip › 1-9 SFigs minimal data set/S4 fig/S4B fig/ACTB.Tif]

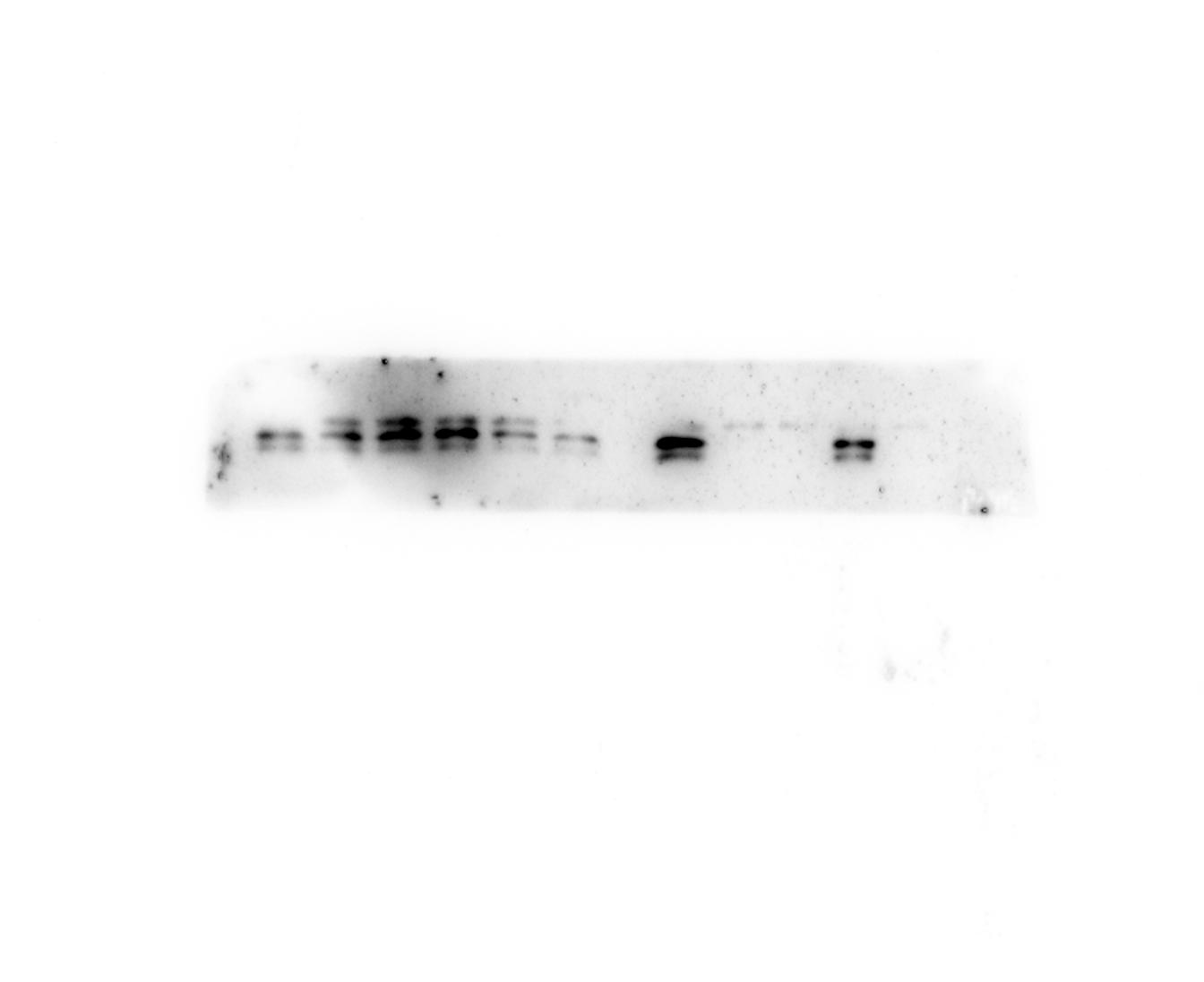

Supplement: S2 Dataset — (ZIP) [file ppat.1012800.s013.zip › 1-9 SFigs minimal data set/S4 fig/S4B fig/P-4E-BP1.Tif]

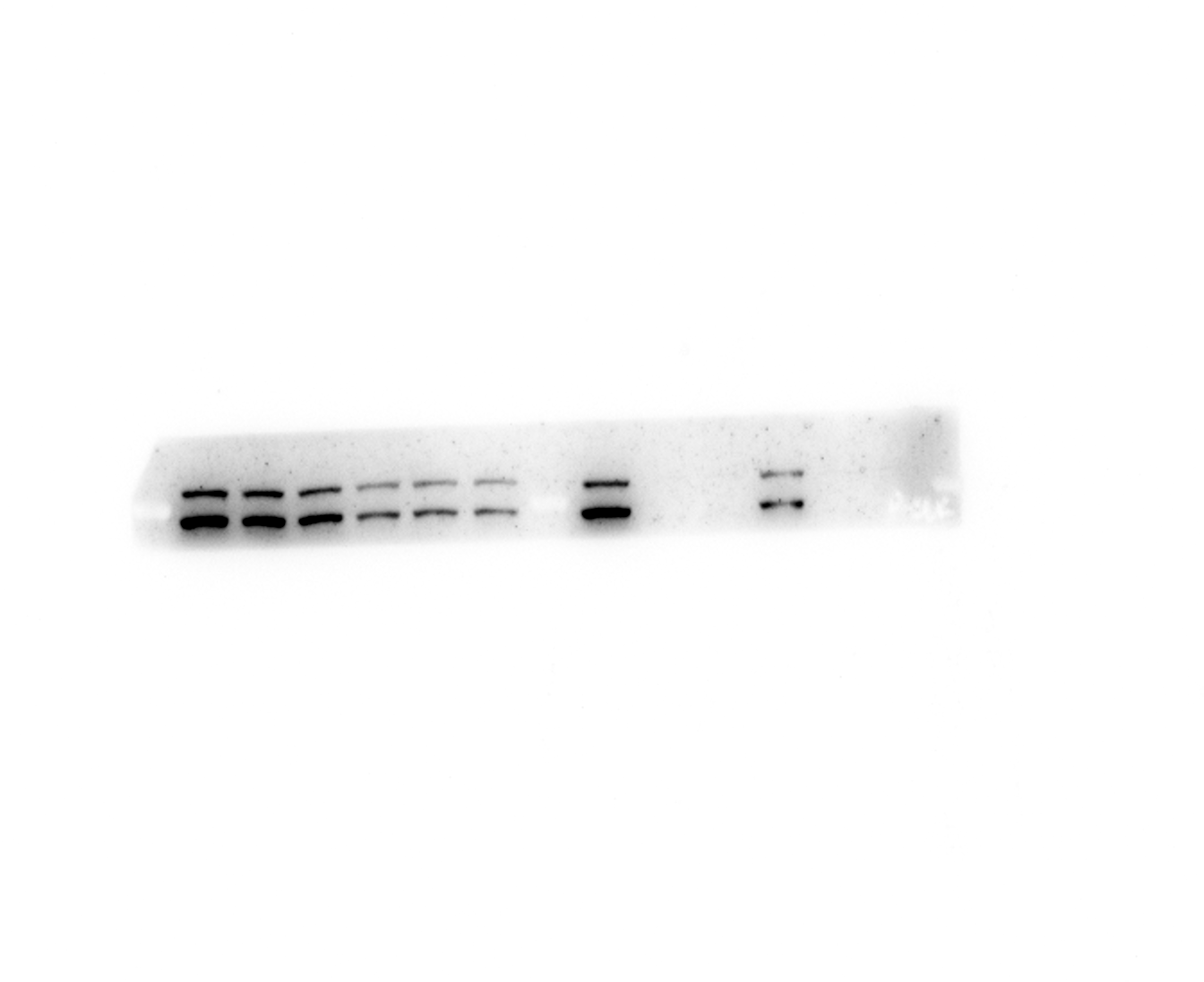

Supplement: S2 Dataset — (ZIP) [file ppat.1012800.s013.zip › 1-9 SFigs minimal data set/S4 fig/S4B fig/P-S6K.Tif]

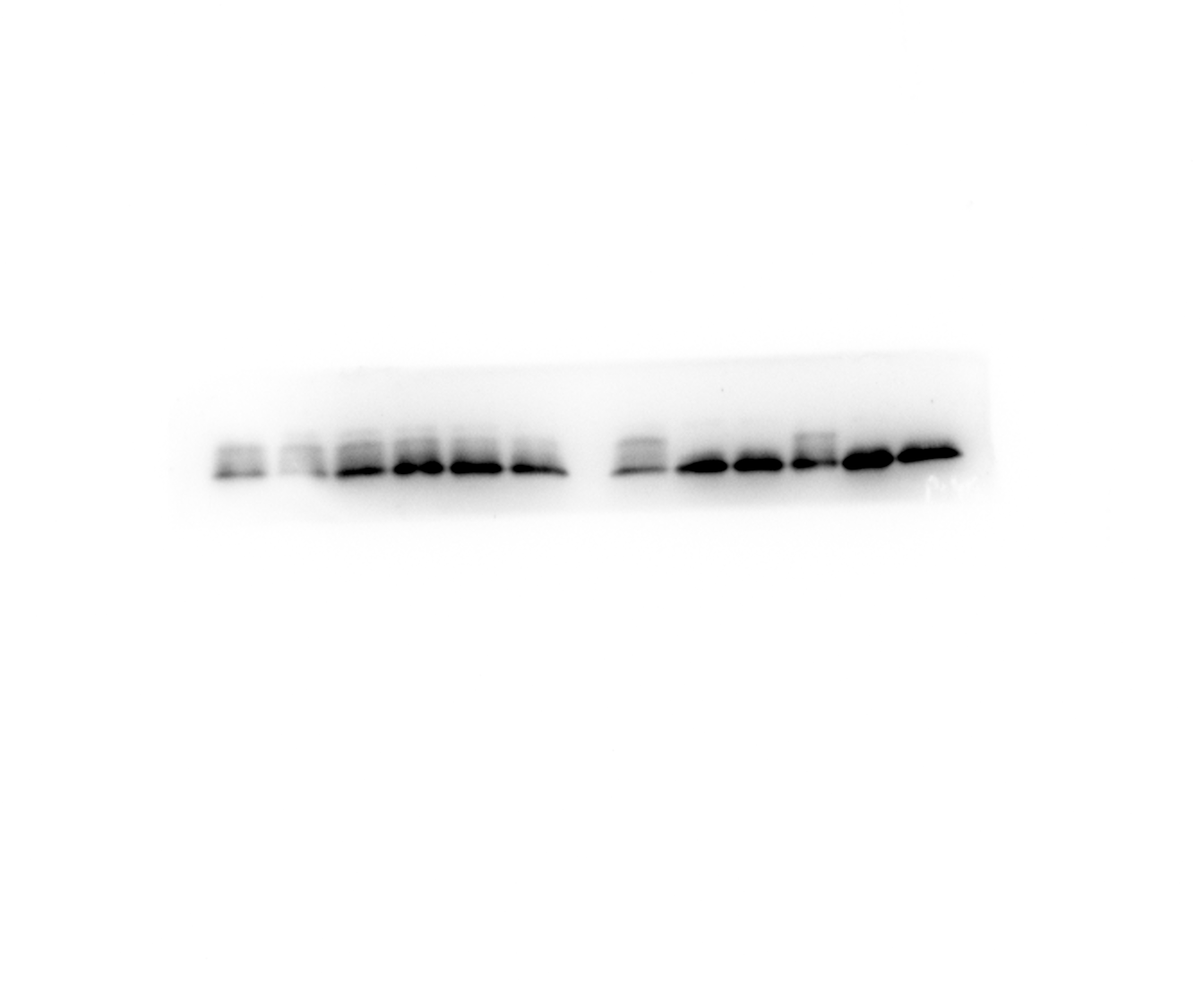

Supplement: S2 Dataset — (ZIP) [file ppat.1012800.s013.zip › 1-9 SFigs minimal data set/S4 fig/S4B fig/Total 4E-BP1.Tif]

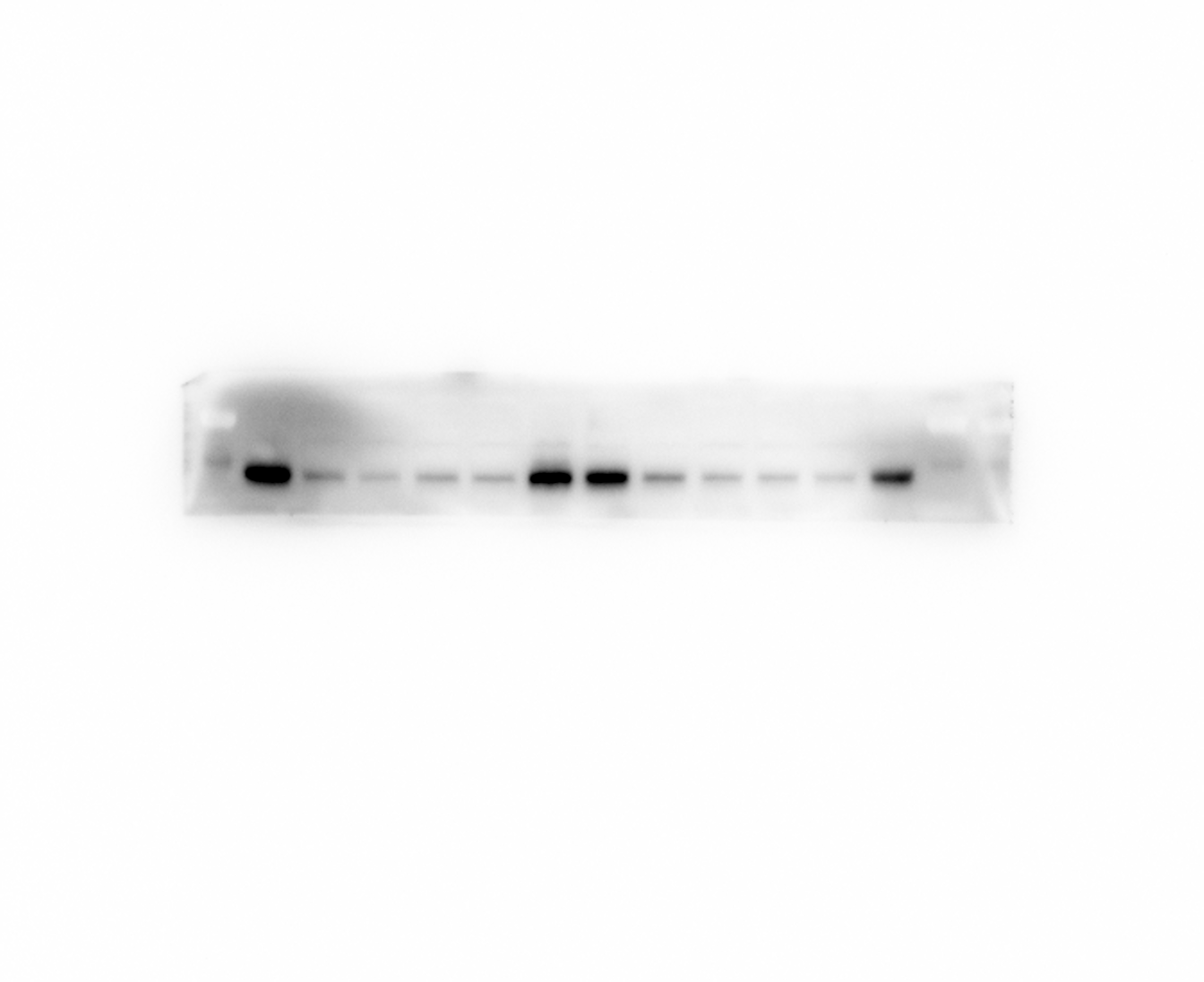

Supplement: S2 Dataset — (ZIP) [file ppat.1012800.s013.zip › 1-9 SFigs minimal data set/S4 fig/S4C fig/ATG5.Tif]

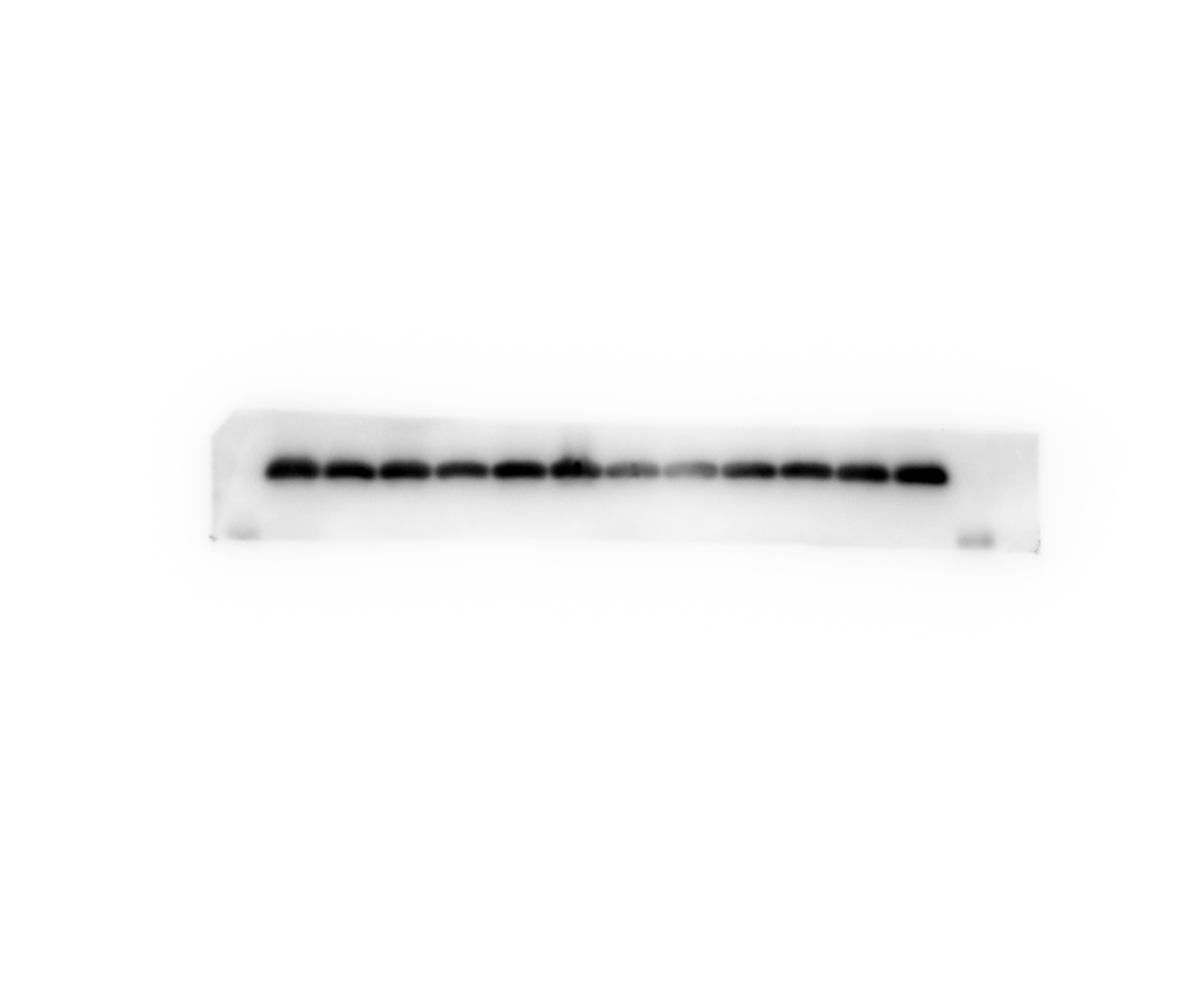

Supplement: S2 Dataset — (ZIP) [file ppat.1012800.s013.zip › 1-9 SFigs minimal data set/S4 fig/S4C fig/Histone H3.Tif]

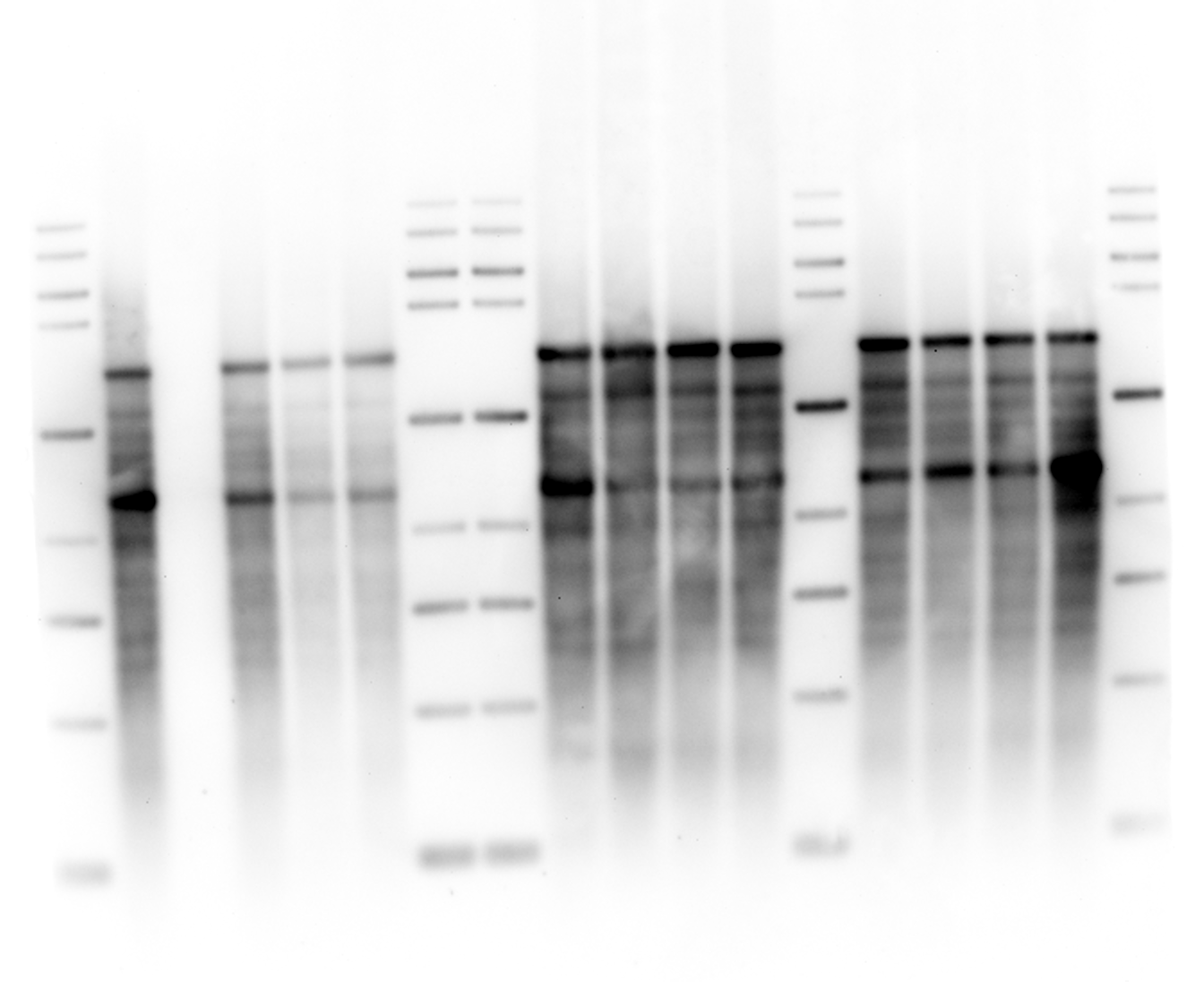

Supplement: S2 Dataset — (ZIP) [file ppat.1012800.s013.zip › 1-9 SFigs minimal data set/S4 fig/S4F.Tif]

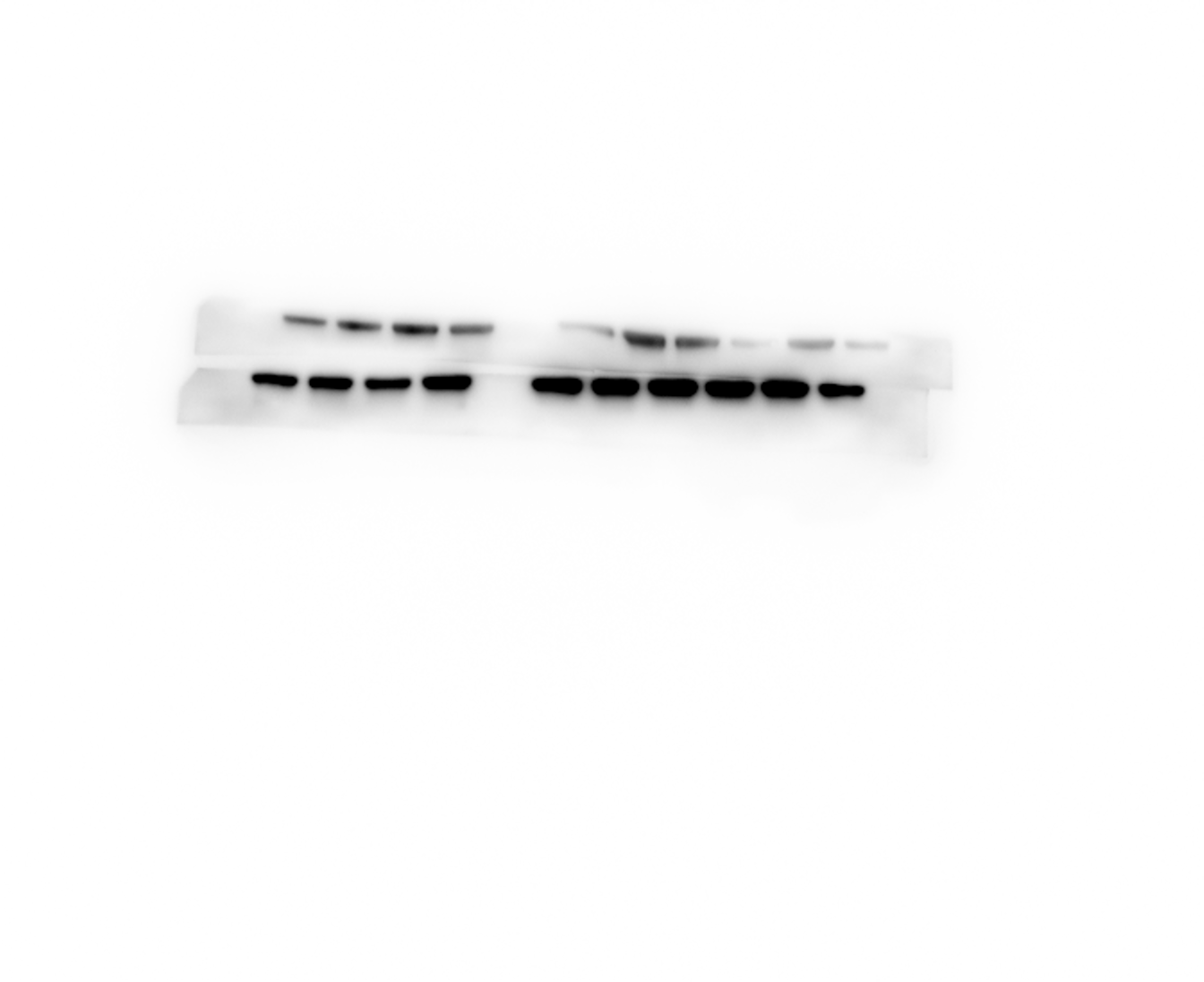

Supplement: S2 Dataset — (ZIP) [file ppat.1012800.s013.zip › 1-9 SFigs minimal data set/S5 fig/S5A fig/ACTB, lower.Tif]

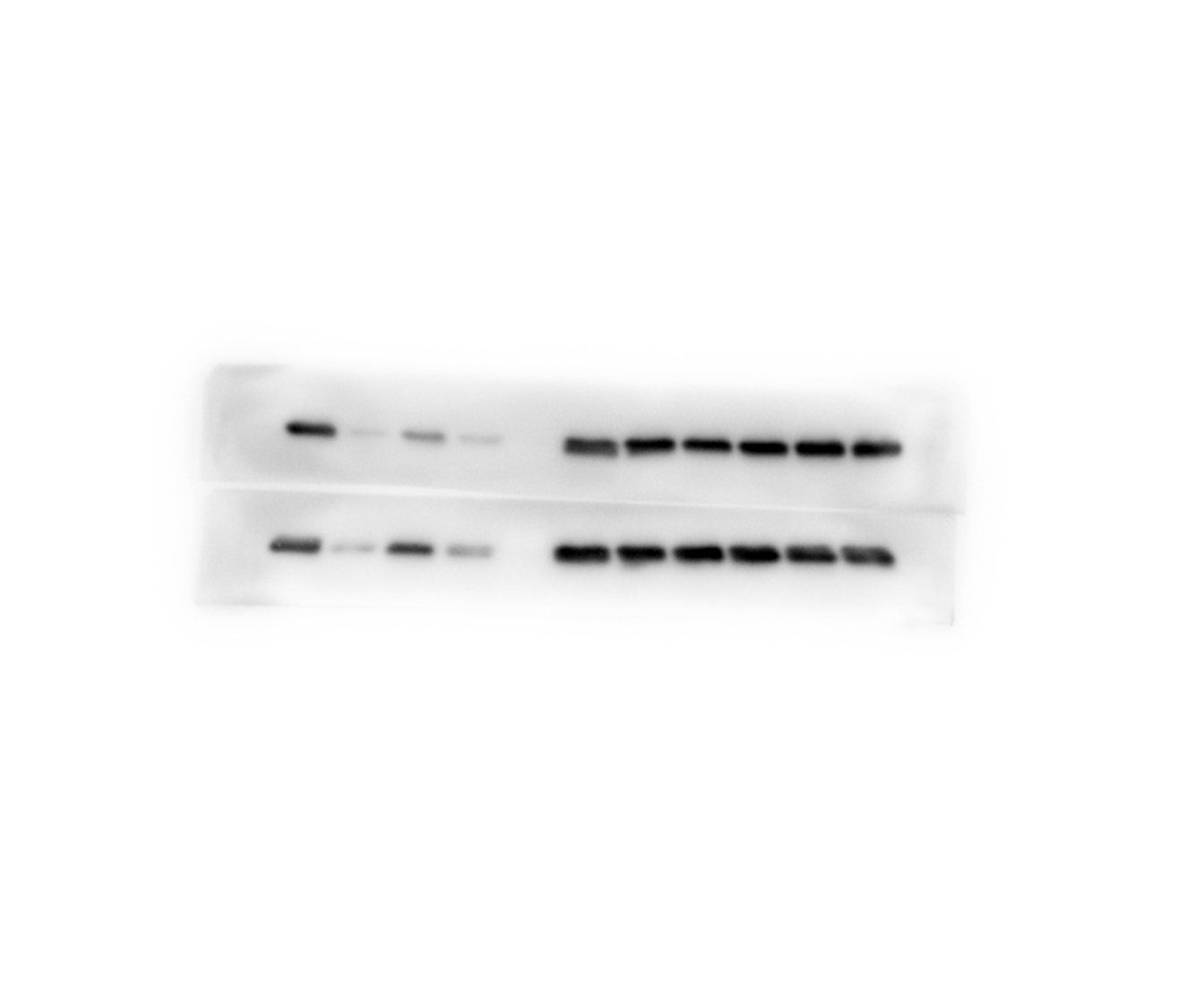

Supplement: S2 Dataset — (ZIP) [file ppat.1012800.s013.zip › 1-9 SFigs minimal data set/S5 fig/S5A fig/RAB7, upper.Tif]

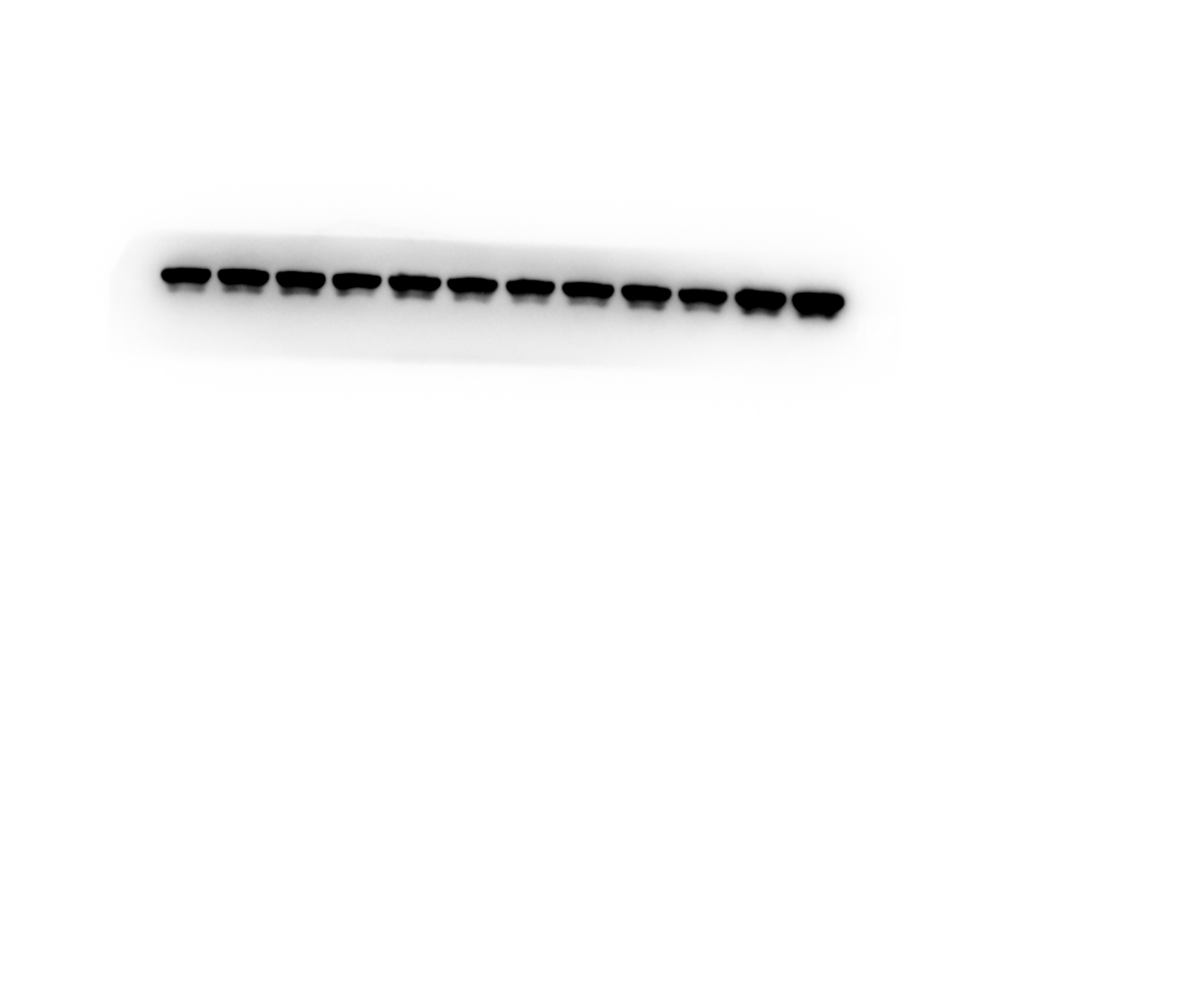

Supplement: S2 Dataset — (ZIP) [file ppat.1012800.s013.zip › 1-9 SFigs minimal data set/S5 fig/S5B fig/ACTB.Tif]

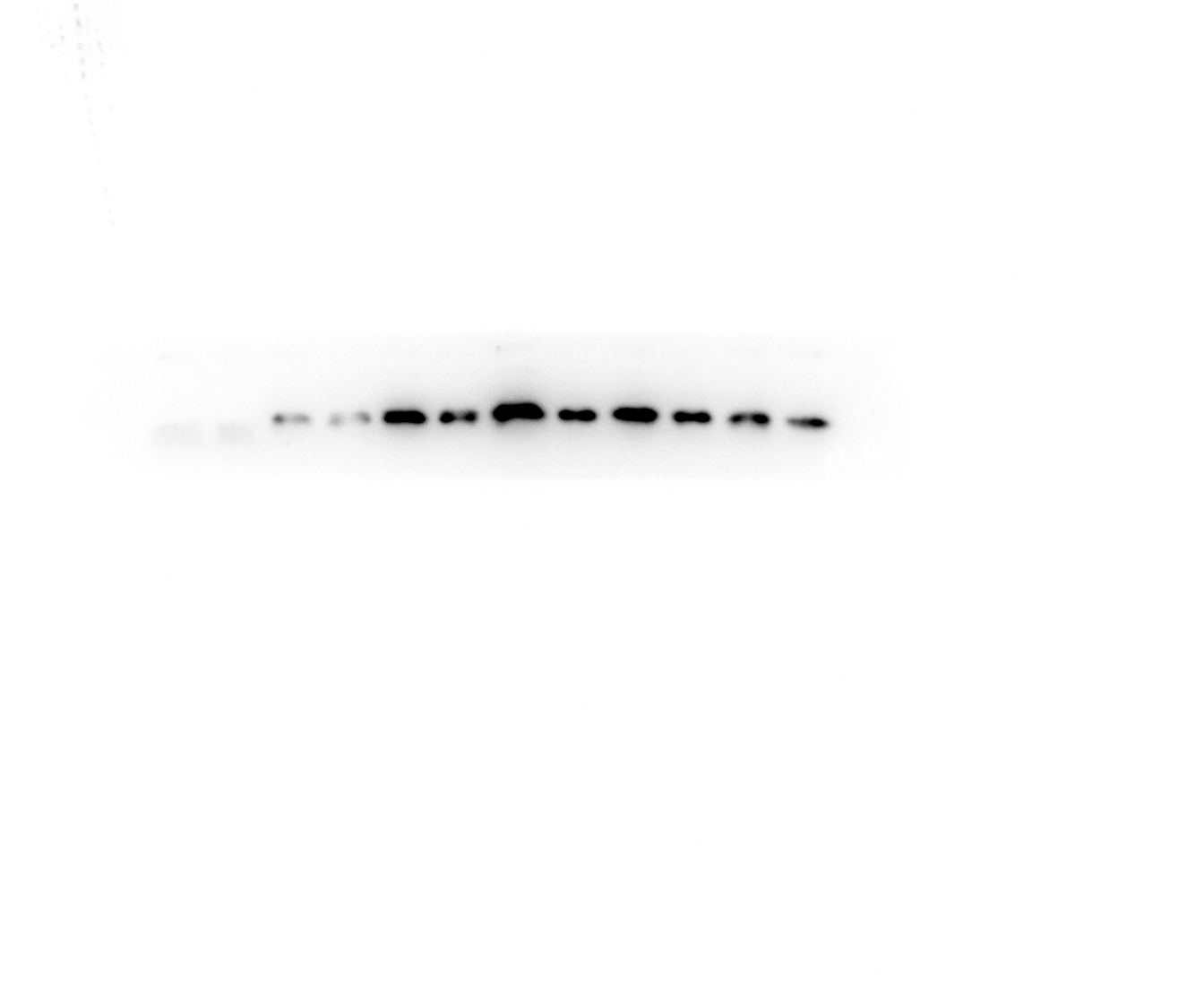

Supplement: S2 Dataset — (ZIP) [file ppat.1012800.s013.zip › 1-9 SFigs minimal data set/S5 fig/S5B fig/P-4E-BP1.Tif]

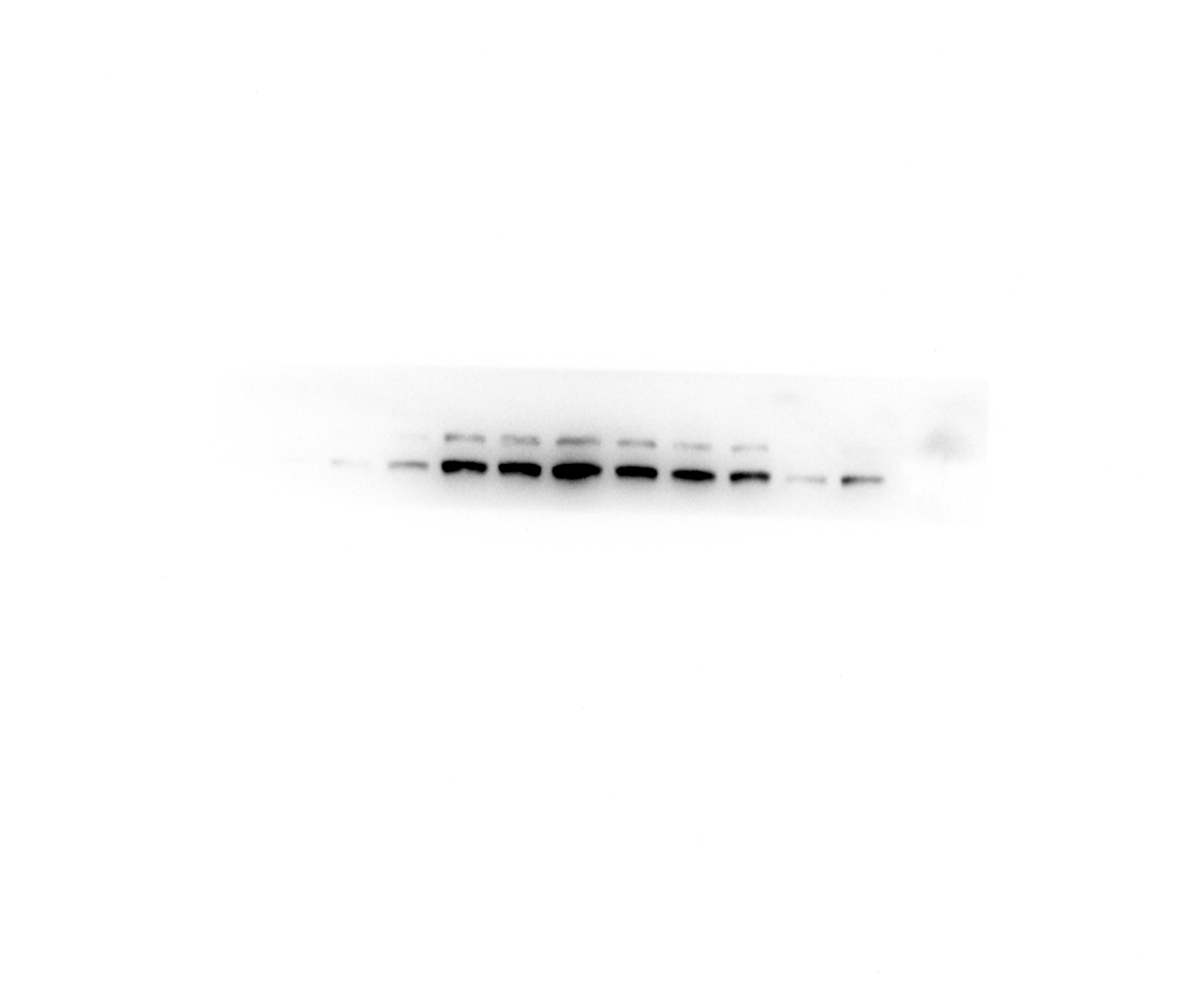

Supplement: S2 Dataset — (ZIP) [file ppat.1012800.s013.zip › 1-9 SFigs minimal data set/S5 fig/S5B fig/P-S6K.Tif]

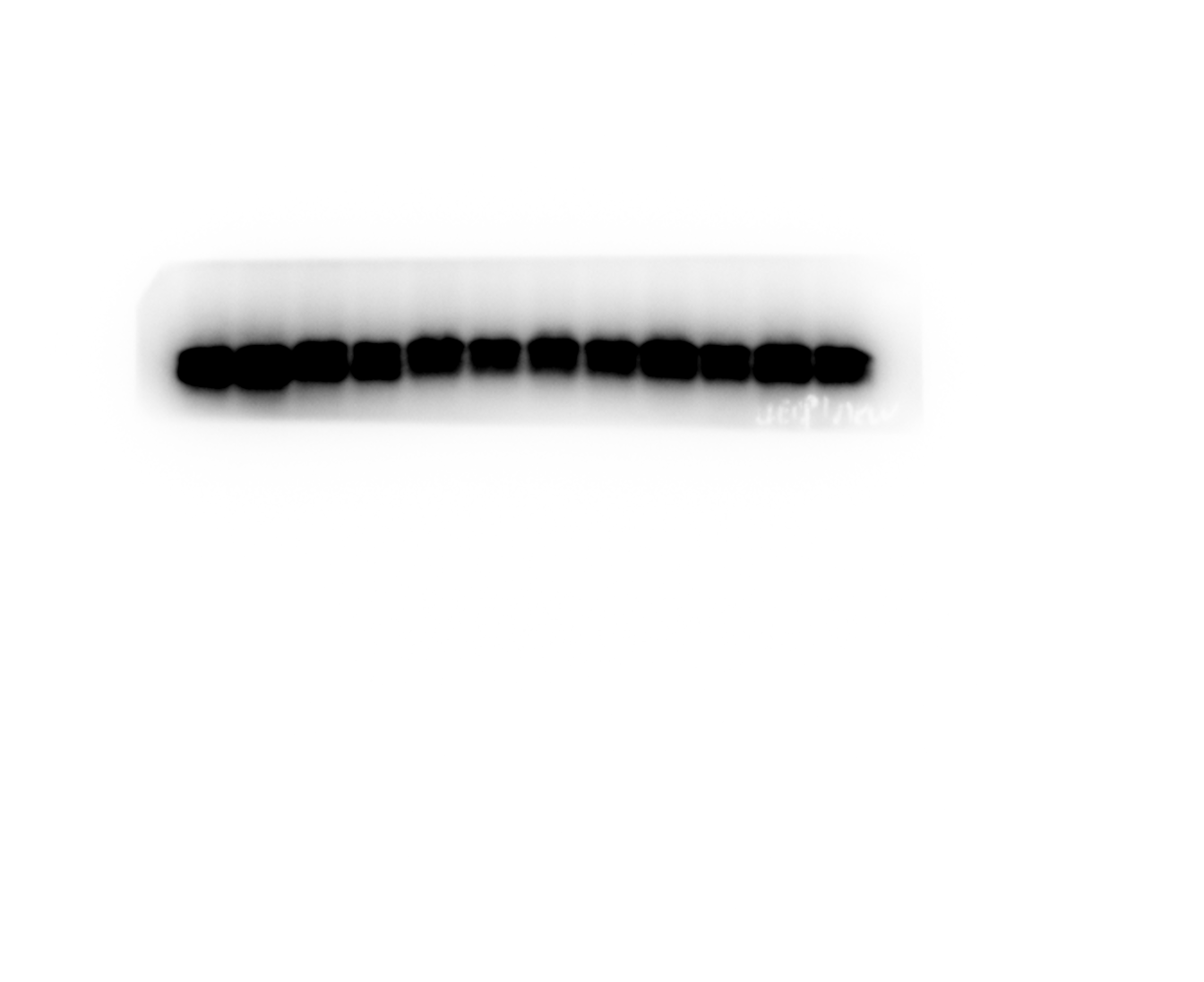

Supplement: S2 Dataset — (ZIP) [file ppat.1012800.s013.zip › 1-9 SFigs minimal data set/S5 fig/S5B fig/total 4E-BP1.Tif]

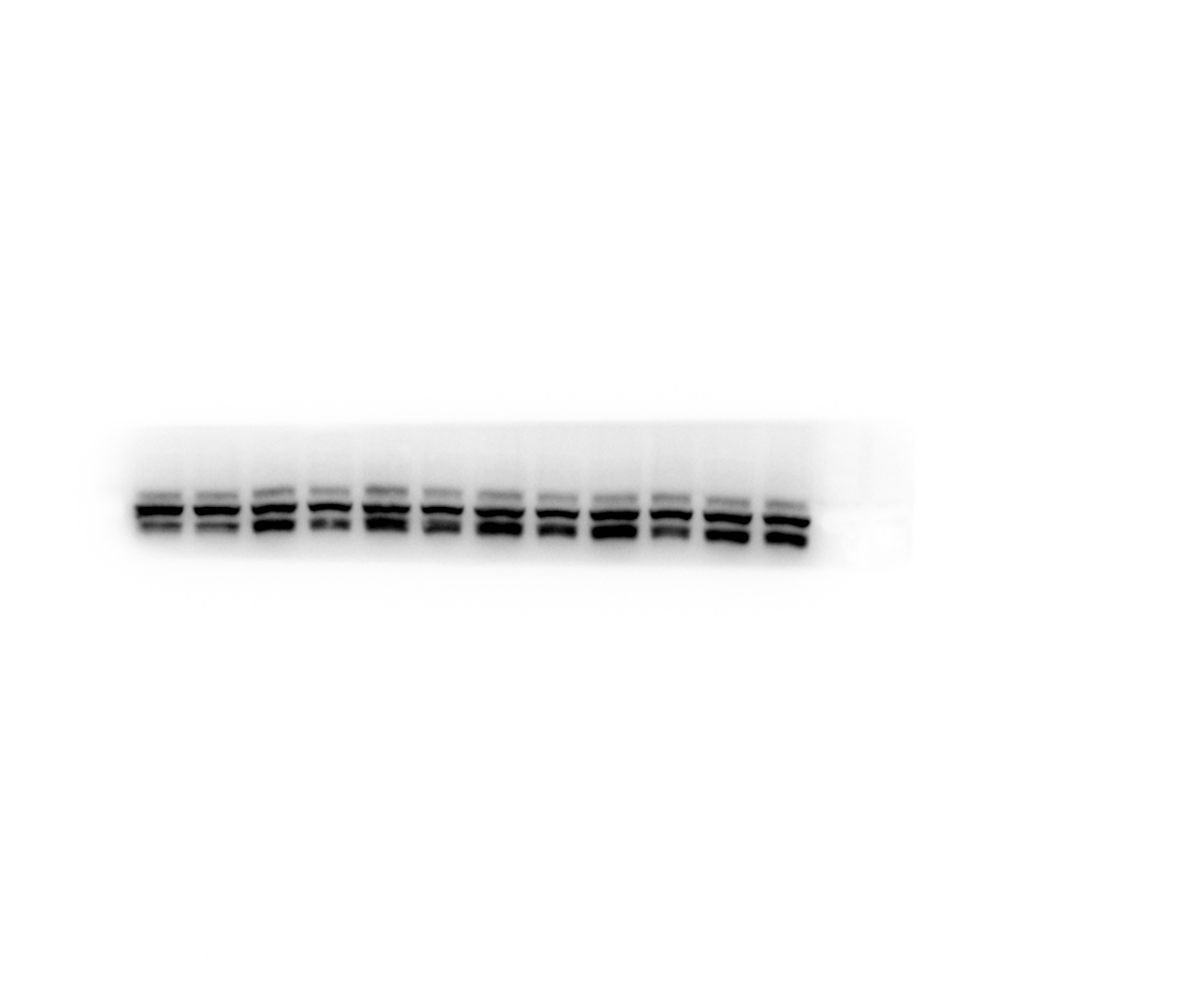

Supplement: S2 Dataset — (ZIP) [file ppat.1012800.s013.zip › 1-9 SFigs minimal data set/S5 fig/S5B fig/total S6K.Tif]

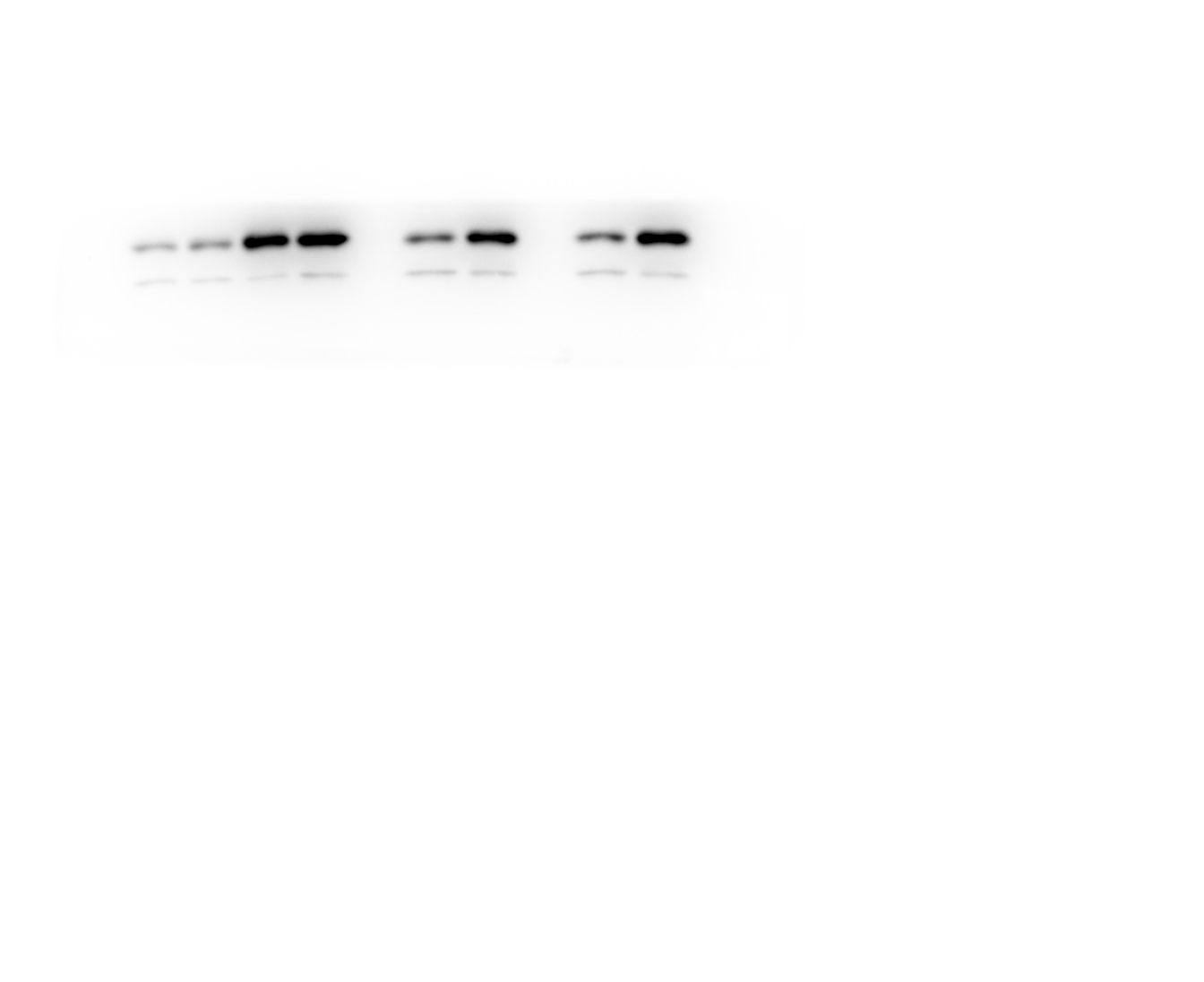

Supplement: S2 Dataset — (ZIP) [file ppat.1012800.s013.zip › 1-9 SFigs minimal data set/S5 fig/S5D fig/STX7.Tif]

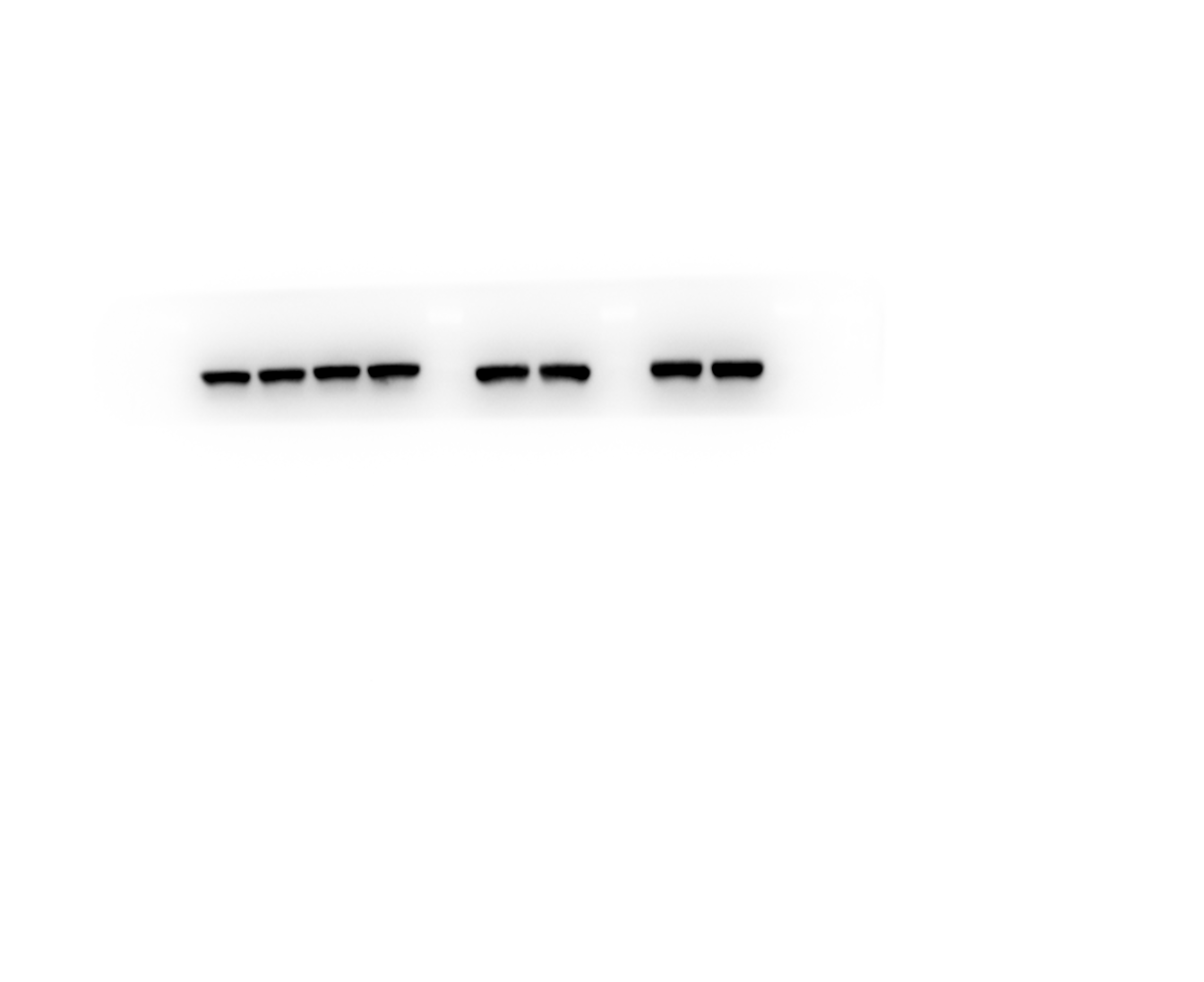

Supplement: S2 Dataset — (ZIP) [file ppat.1012800.s013.zip › 1-9 SFigs minimal data set/S5 fig/S5D fig/Tubulin.Tif]

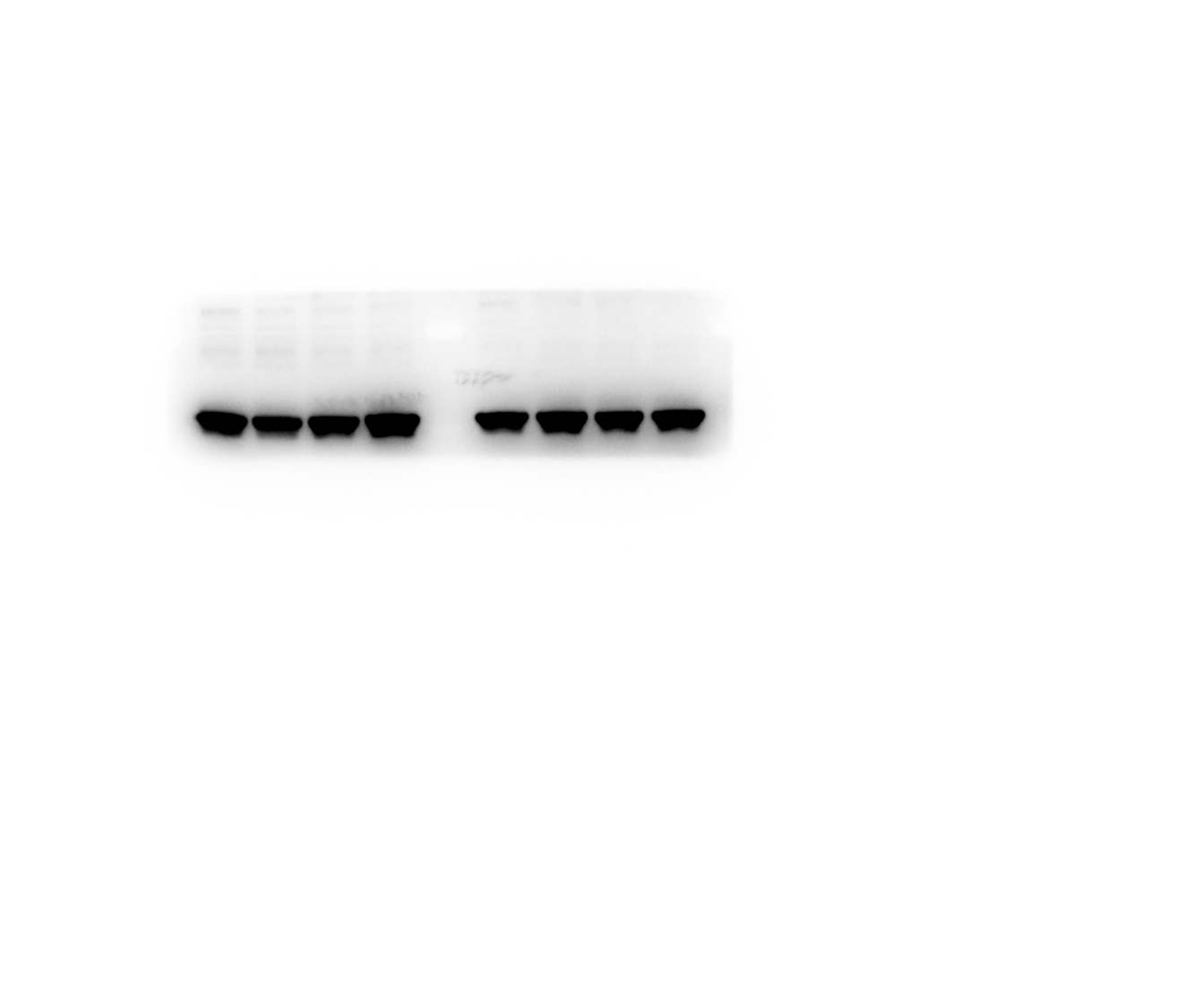

Supplement: S2 Dataset — (ZIP) [file ppat.1012800.s013.zip › 1-9 SFigs minimal data set/S6 fig/S6B fig/ACTB for CTSD.Tif]
