# Supplementary material for: Manganese is a potent inducer of lysosomal activity that inhibits de novo HBV infection
Source: PLoS Pathog. 2025 Jan 2;21(1):e1012800. doi: 10.1371/journal.ppat.1012800 (PMC11694974; doi:10.1371/journal.ppat.1012800)
Supplement: S1 Table — (DOCX) [file ppat.1012800.s010.docx]

**S1 Table.** siRNA sequences

| **Name** | **Company** | **Sequence (5’-3’)** |
| --- | --- | --- |
| siHrs-1 | RiboBio | GAGGTAAACGTCCGTAACA |
| siHrs-2 | RiboBio | GAAAGTGTTCTTCCAAGTA |
| siTSG101-1 | RiboBio | GAATCTGTATGCAGAAGAA |
| siTSG101-2 | RiboBio | GGTTACCCGTTTAGATCAA |
| siTSG101-3 | RiboBio | CCACAACAAGTTCTCAGTA |
| siVAMP7-1 | RiboBio | GCCTAGACAAAGTGATGGA |
| siVAMP7-2 | RiboBio | TGAAGAACCTCAAGCTCACTATTAT |
| siVAMP7-3 | RiboBio | GATTCTGGCTAAGATACCTTCTGAA |
| siRab7-1 | RiboBio | CAACGAATTTCCTGAACCT |
| siRab7-2 | RiboBio | GAGCTGACTTTCTGACCAA |
| siRab7-3 | RiboBio | CCAGACGATTGCACGGAAT |
| siTFEB-1 | RiboBio | CCAGACGATTGCACGGAAT |
| siTFEB-2 | RiboBio | CCAGACGATTGCACGGAAT |
| siTFEB-3 | RiboBio | CCAGACGATTGCACGGAAT |
| siSTX7-1 | Sangon Biotech | GAGTTTGTTGCTCGAGTAATT |
| siSTX7-2 | Sangon Biotech | GCAAATCAGCAGCTGTCAATT |
