# Supplementary material for: Manganese is a potent inducer of lysosomal activity that inhibits de novo HBV infection
Source: PLoS Pathog. 2025 Jan 2;21(1):e1012800. doi: 10.1371/journal.ppat.1012800 (PMC11694974; doi:10.1371/journal.ppat.1012800)
Supplement: S2 Table — (DOCX) [file ppat.1012800.s011.docx]

**S2 Table.** Primer sequences for PCR and RT-qPCR

| **Oligonucleotide** | **Sequence (5’-3’)** |
| --- | --- |
| Mx1 F | ACAGGACCATCGGAATCTTG |
| Mx1 R | CCCTTCTTCAGGTGGAACAC |
| ISG15 F | GGTGCTGCCTGCCGAAGCC |
| ISG15 R | GTGATCTGCGCCTTCAGCTCT |
| OAS1 F | CTACCAGGCTGTCCAGAAGG |
| OAS1 R | TCCAGCTGGCTGAAATACCA |
| HBV DNA F | ATCCTGCTGCTATGCCTCATCTT |
| HBV DNA R | ACAGTGGGGGAAAGCCCTACGAA |
| HBV RNA F | ACCGACCTTGAGGCATACTT |
| HBV RNA R | GCCTACAGCCTCCTAGTACA |
| hGAPDH F | TCAAGATCATCAGCAATGCCT |
| hGAPDH R | GATGATGTTCTGGAGAGCCC |
| HBV1.1-ΔHBc-F1 | GCAGTACATCAAGTGTATCATATGCCAAGTACGCCCCCTA |
| HBV1.1-ΔHBc-R1 | ATAAGGGTCGATGTCCACGCCCCAAAGCCACCCAA |
| HBV1.1-ΔHBc-F2 | TTGGGTGGCTTTGGGGCGTGGACATCGACCCTTAT |
| HBV1.1-ΔHBc-R2 | TGGTGGAAGGTTGTGGAATTCCACTGCATGGCCTGAGGAT |
