# Supplementary figures and images for: Manganese is a potent inducer of lysosomal activity that inhibits de novo HBV infection (part 2 of 2)
Source: PLoS Pathog. 2025 Jan 2;21(1):e1012800. doi: 10.1371/journal.ppat.1012800 (PMC11694974; doi:10.1371/journal.ppat.1012800)

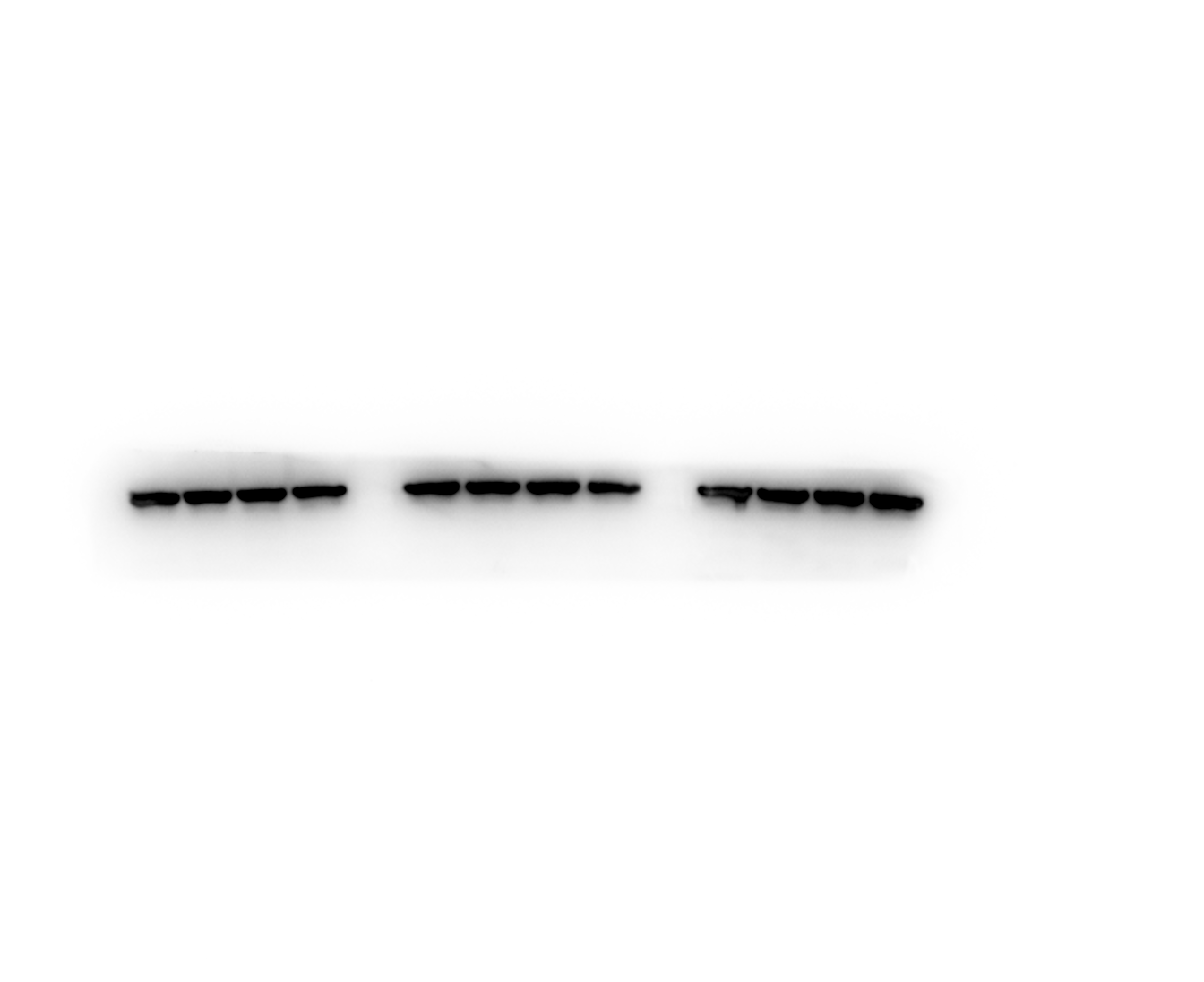

Supplement: S2 Dataset — (ZIP) [file ppat.1012800.s013.zip › 1-9 SFigs minimal data set/S6 fig/S6B fig/ACTB for LAMP1.Tif]

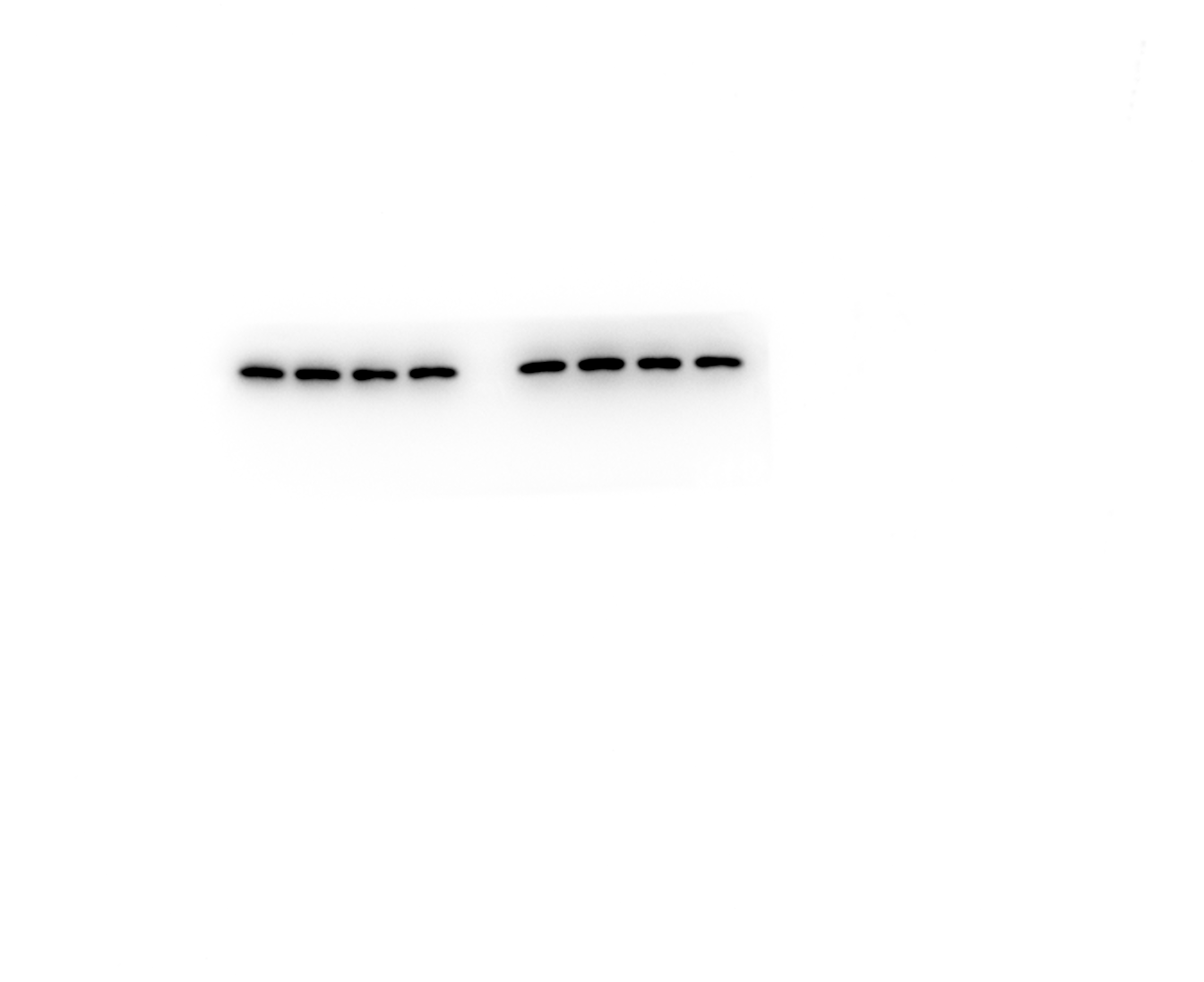

Supplement: S2 Dataset — (ZIP) [file ppat.1012800.s013.zip › 1-9 SFigs minimal data set/S6 fig/S6B fig/CTSD.Tif]

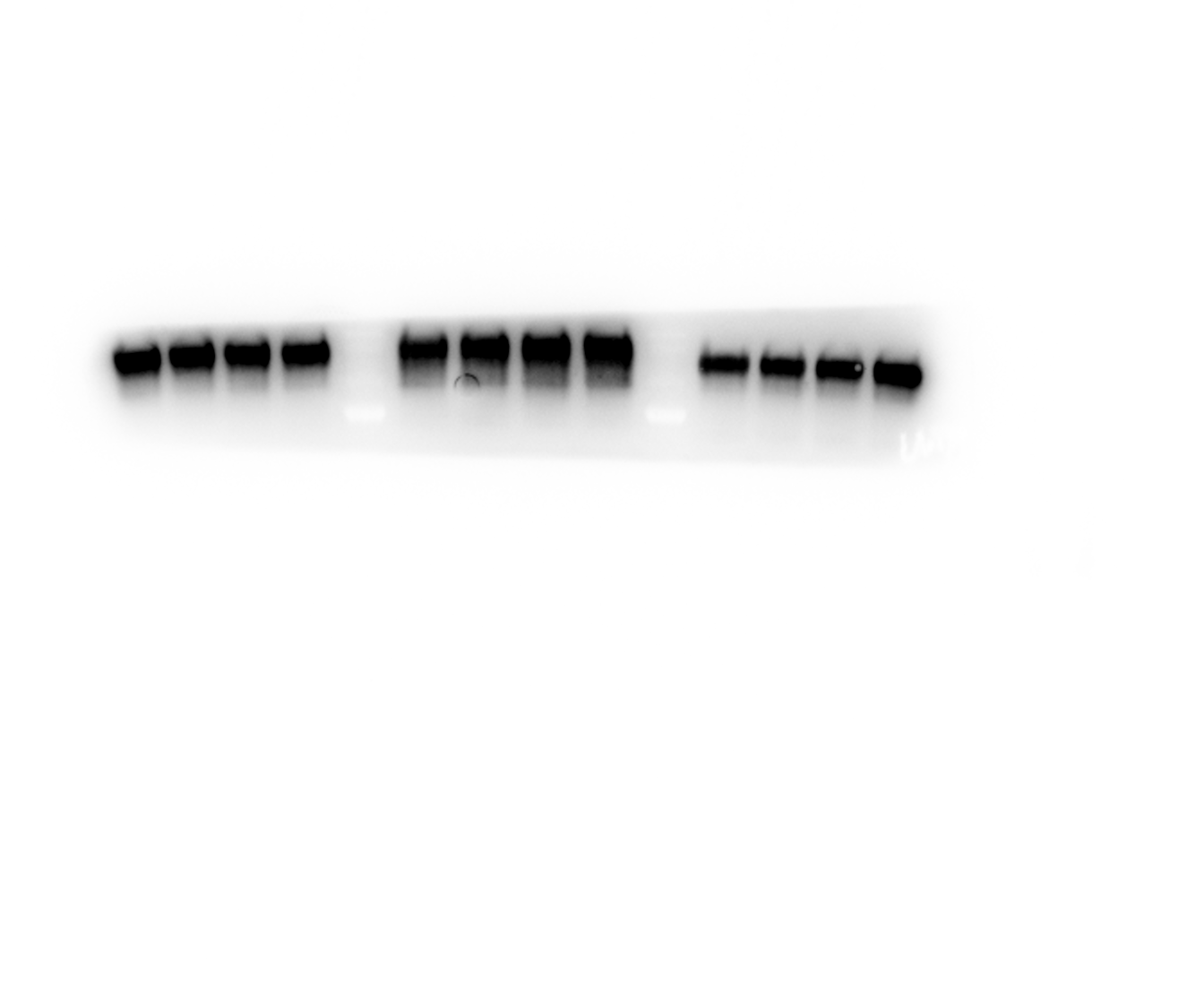

Supplement: S2 Dataset — (ZIP) [file ppat.1012800.s013.zip › 1-9 SFigs minimal data set/S6 fig/S6B fig/LAMP1.Tif]

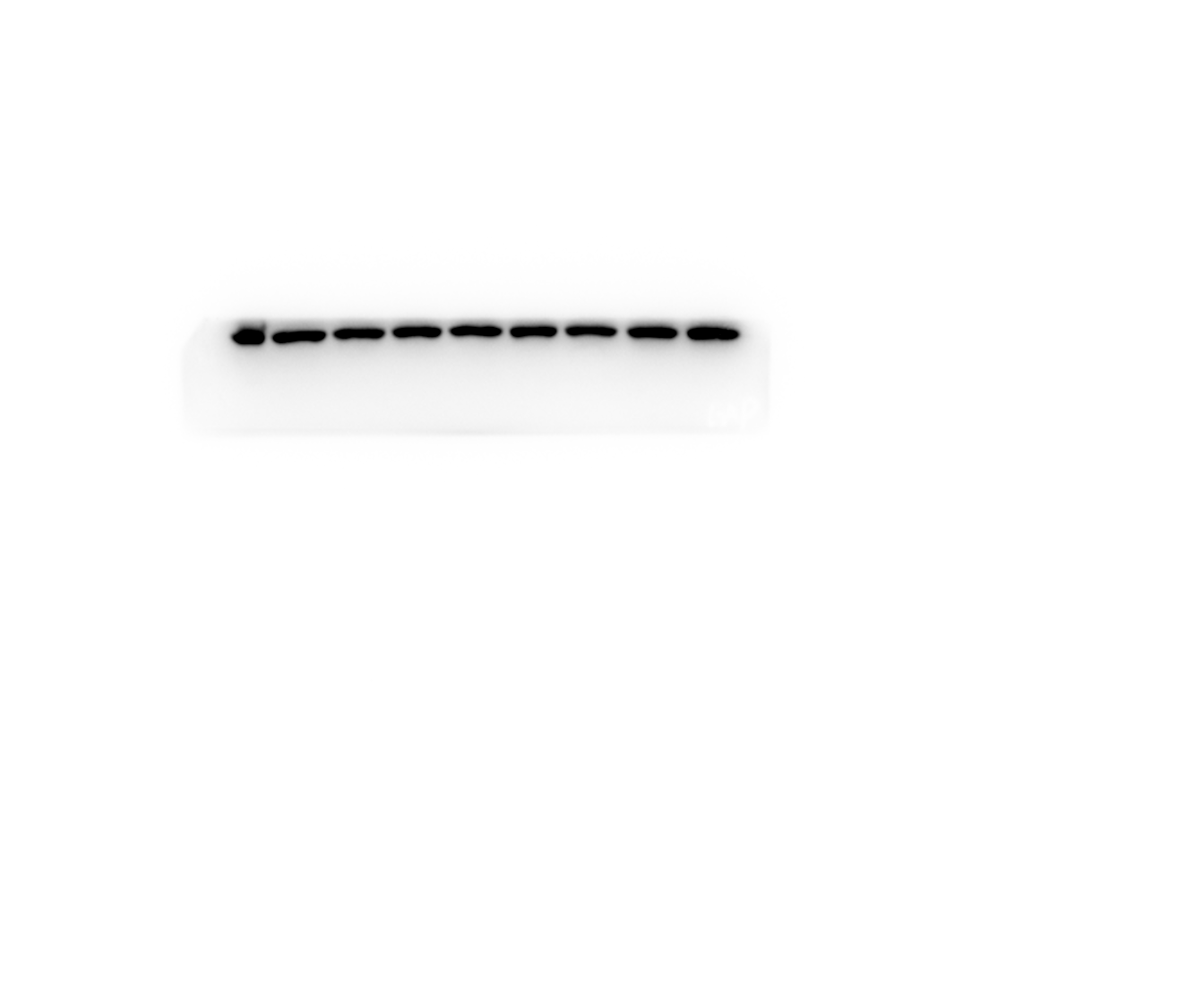

Supplement: S2 Dataset — (ZIP) [file ppat.1012800.s013.zip › 1-9 SFigs minimal data set/S6 fig/S6D fig/GAPDH.Tif]

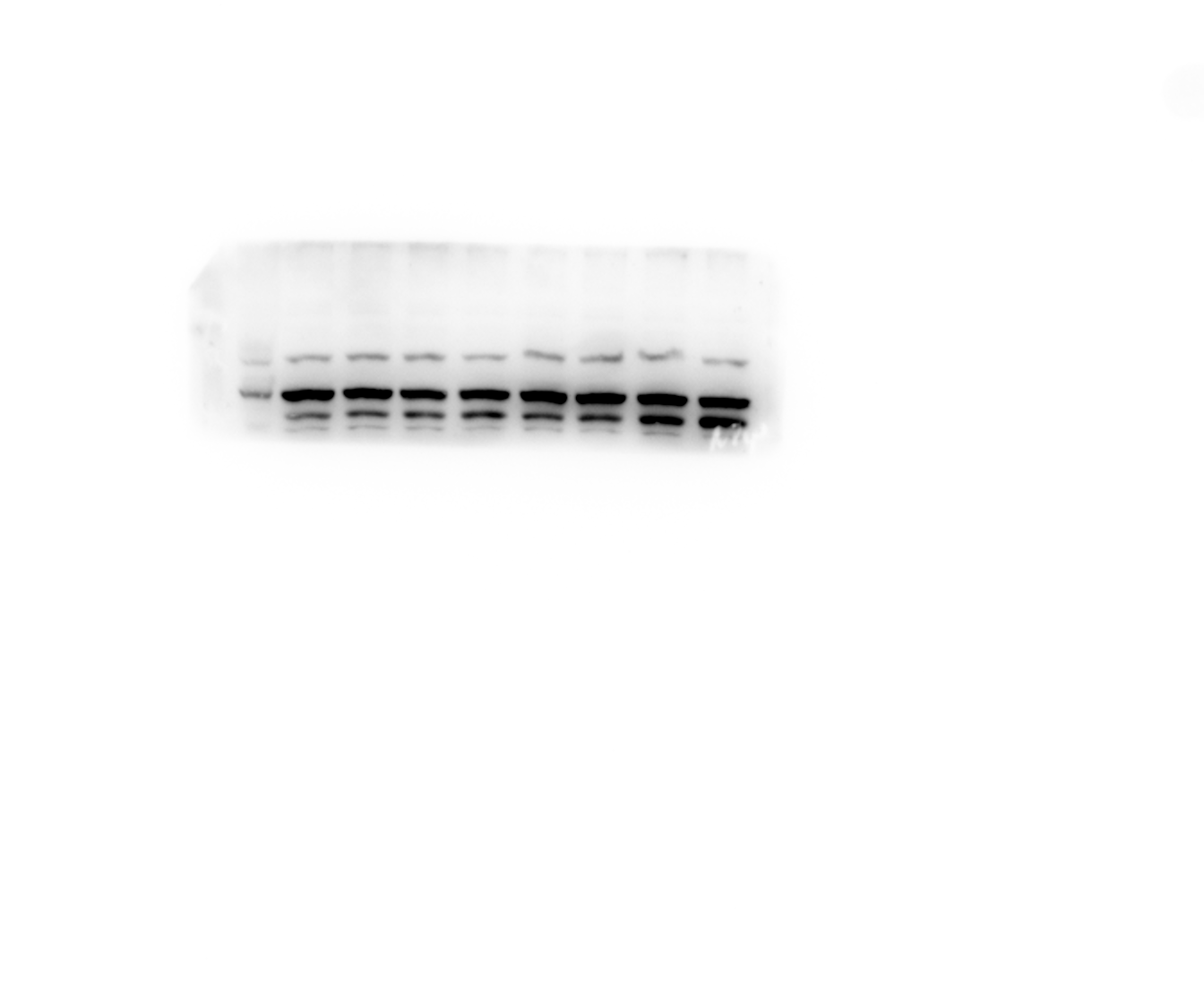

Supplement: S2 Dataset — (ZIP) [file ppat.1012800.s013.zip › 1-9 SFigs minimal data set/S6 fig/S6D fig/NTCP.Tif]

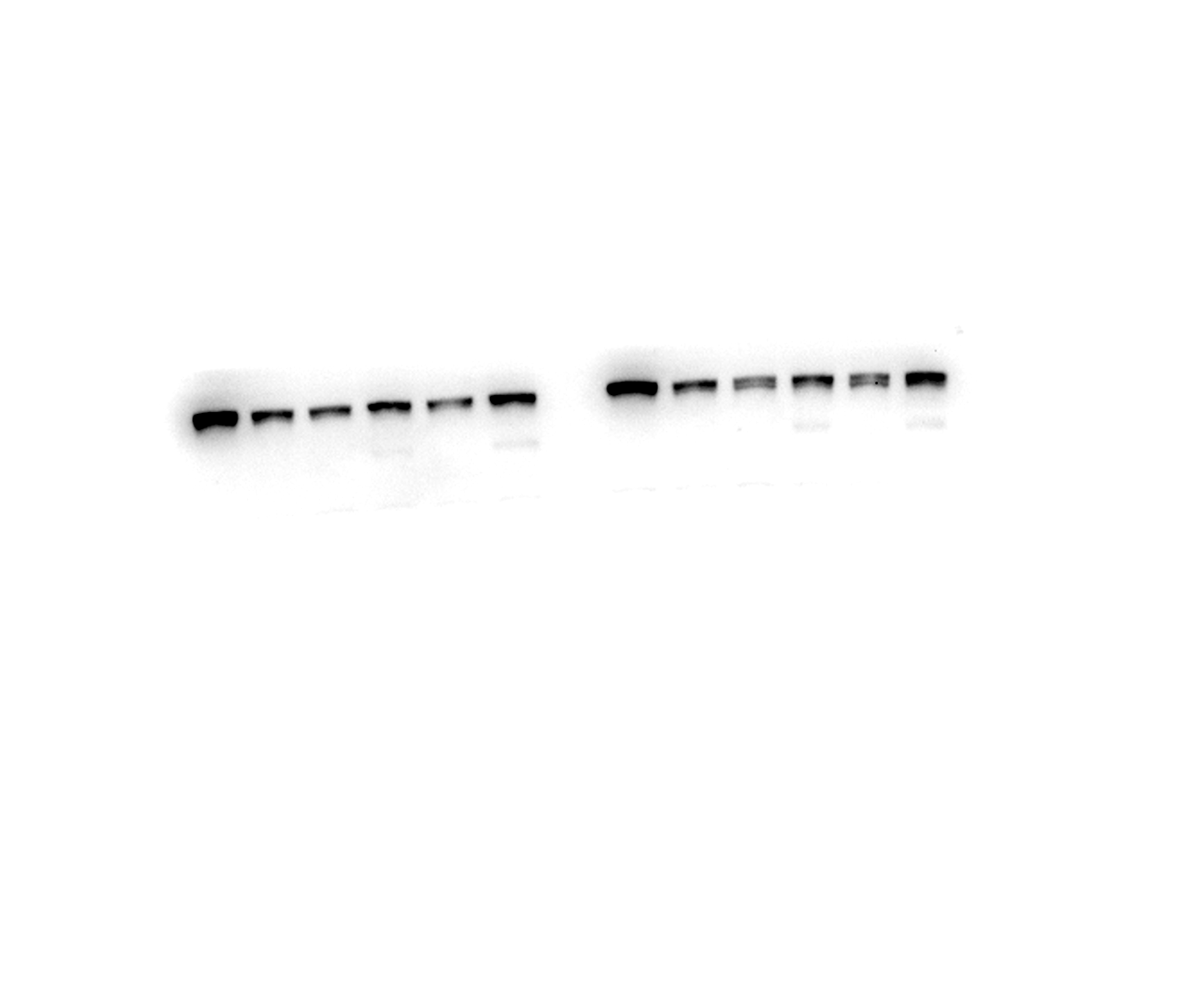

Supplement: S2 Dataset — (ZIP) [file ppat.1012800.s013.zip › 1-9 SFigs minimal data set/S7 fig/S7A fig/EGFR.Tif]

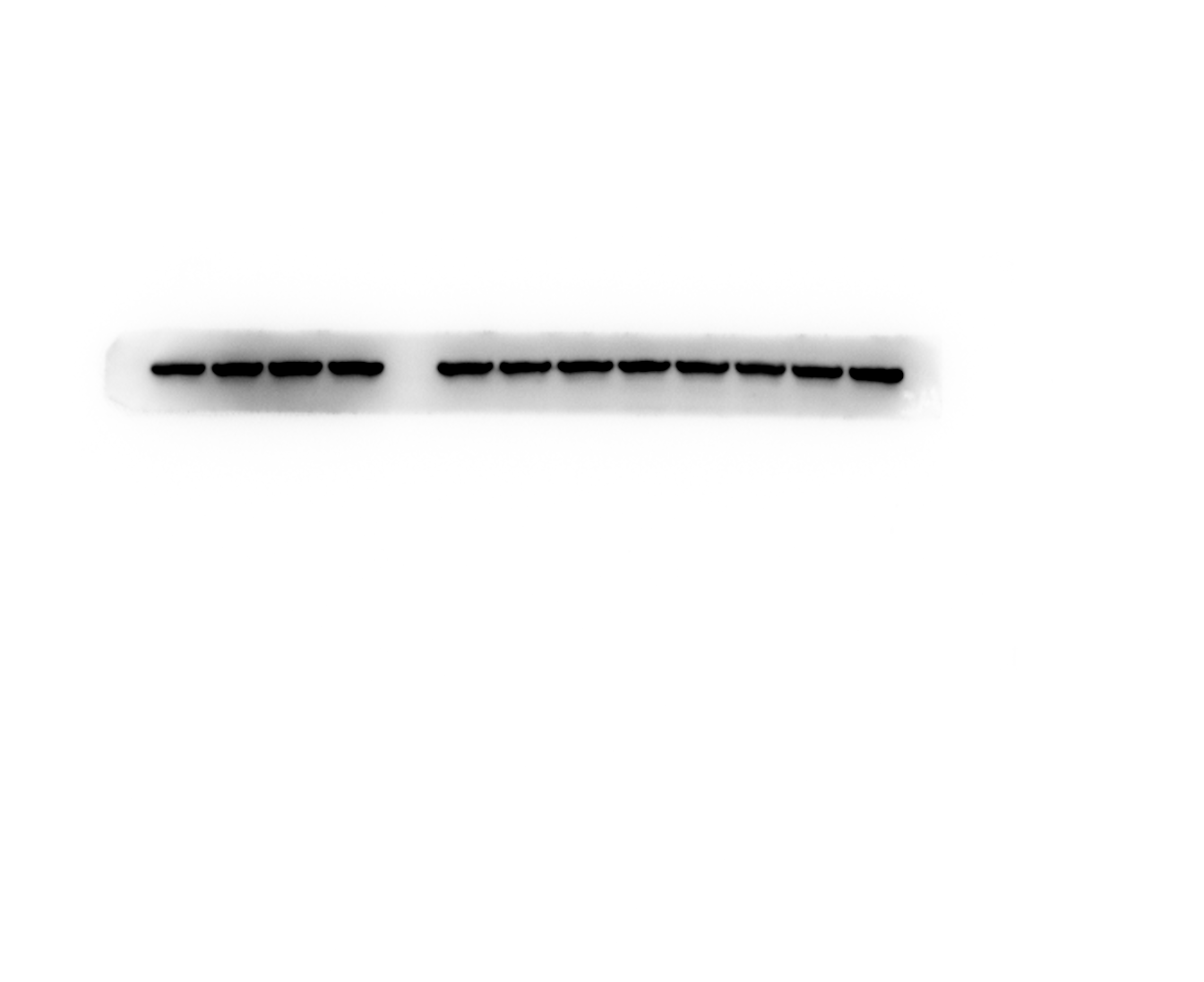

Supplement: S2 Dataset — (ZIP) [file ppat.1012800.s013.zip › 1-9 SFigs minimal data set/S7 fig/S7C fig/ACTB for CTSD.Tif]

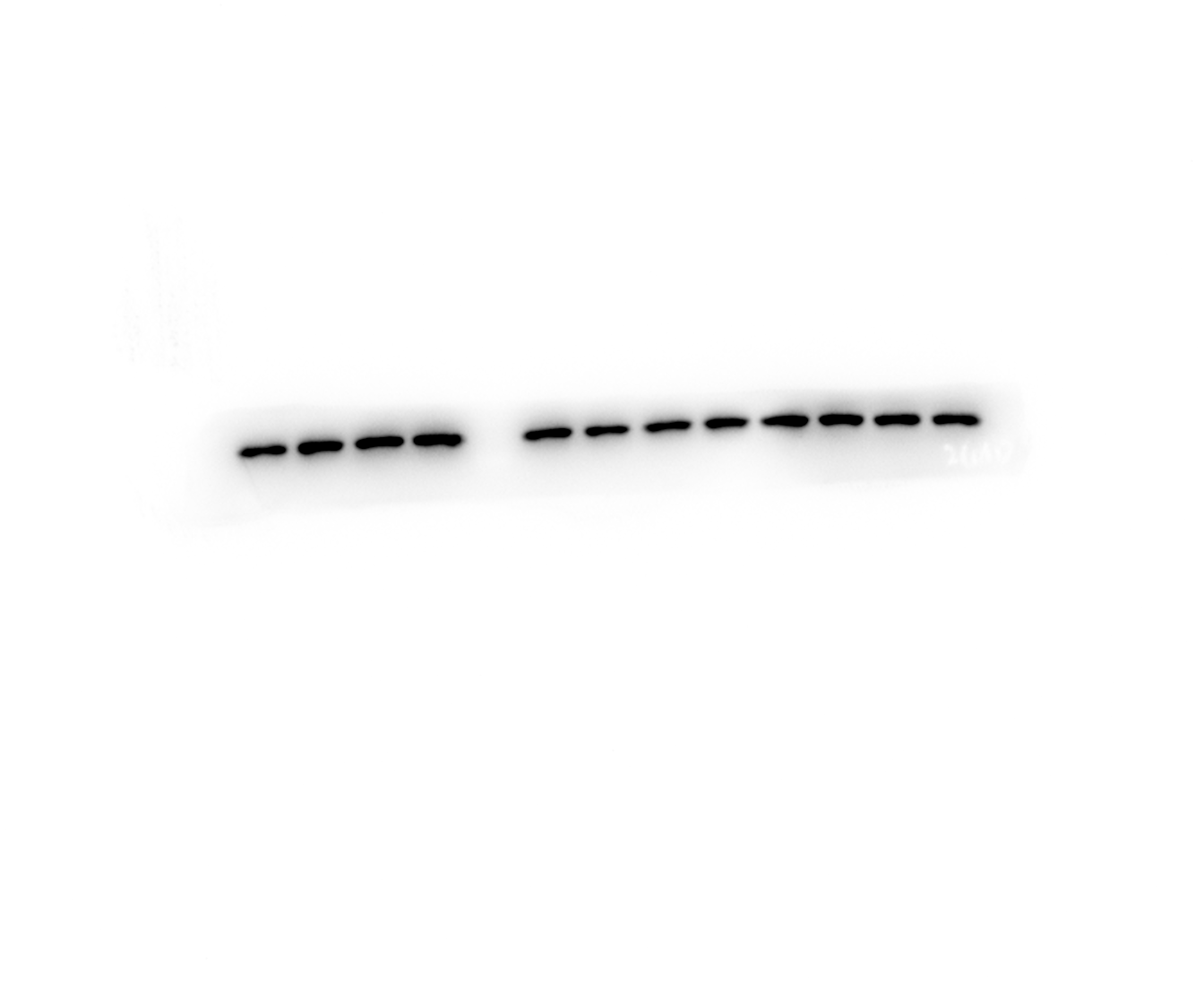

Supplement: S2 Dataset — (ZIP) [file ppat.1012800.s013.zip › 1-9 SFigs minimal data set/S7 fig/S7C fig/CTSD.Tif]

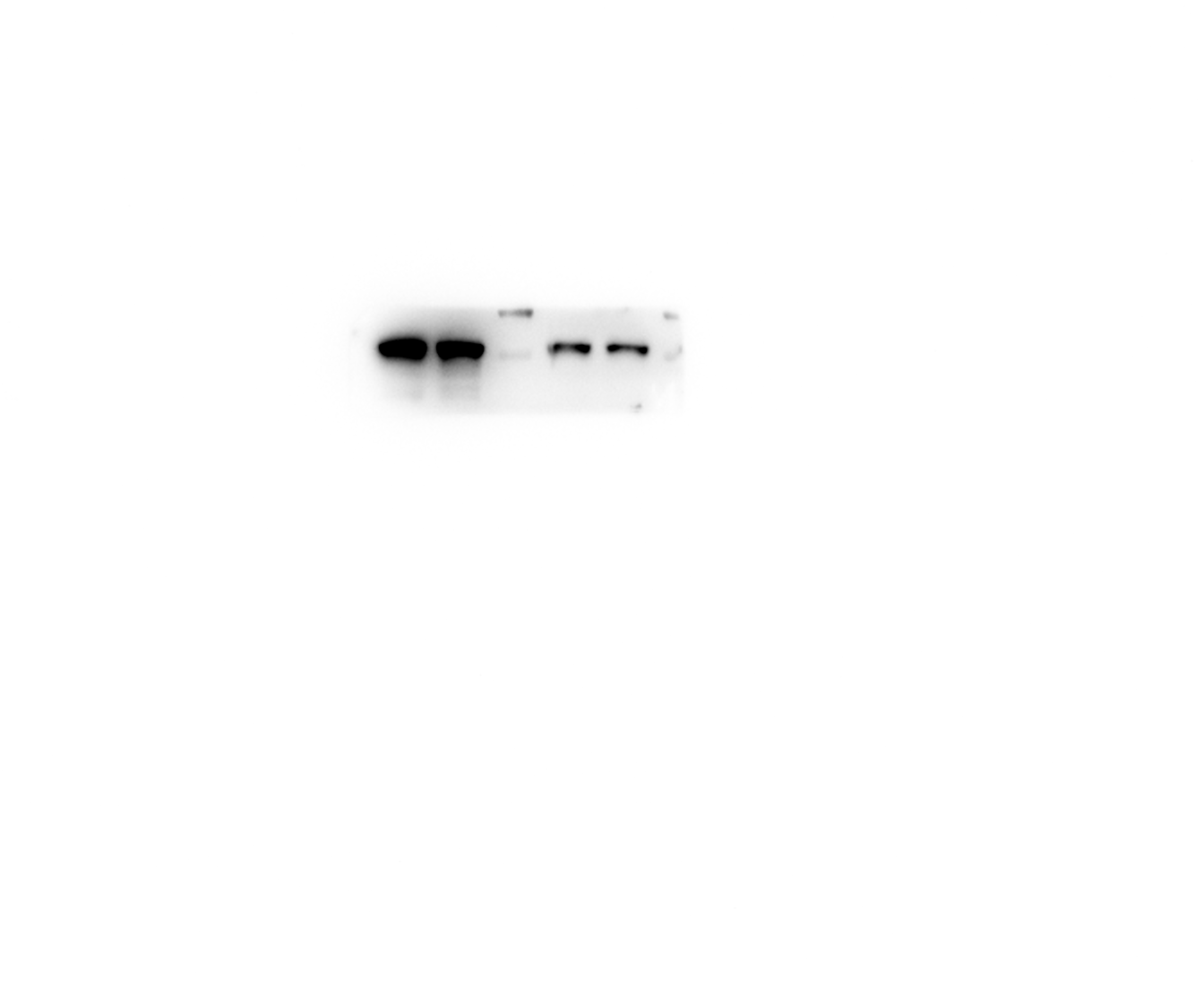

Supplement: S2 Dataset — (ZIP) [file ppat.1012800.s013.zip › 1-9 SFigs minimal data set/S7 fig/S7D fig/ATP6V1B2.Tif]

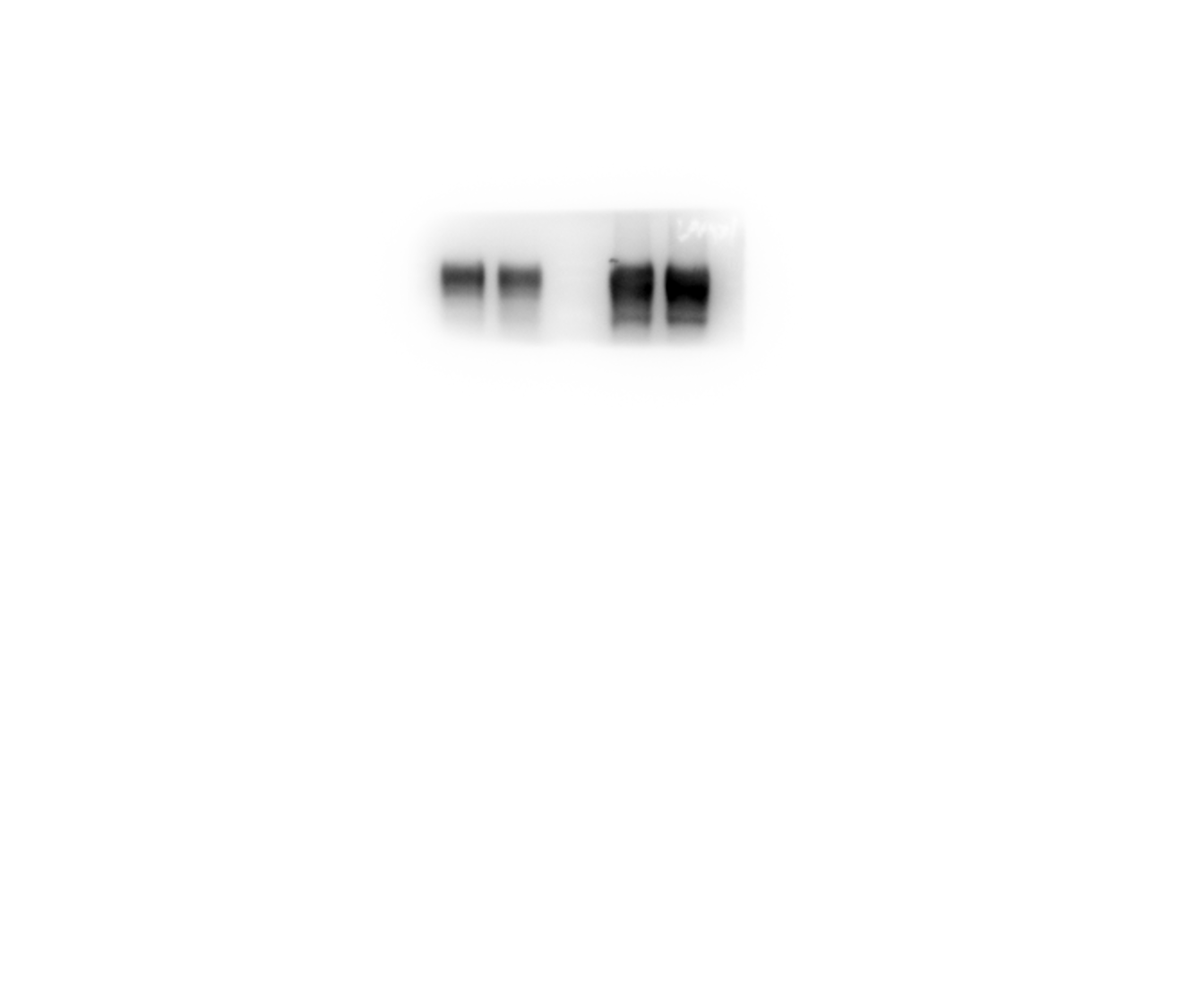

Supplement: S2 Dataset — (ZIP) [file ppat.1012800.s013.zip › 1-9 SFigs minimal data set/S7 fig/S7D fig/GAPDH.Tif]

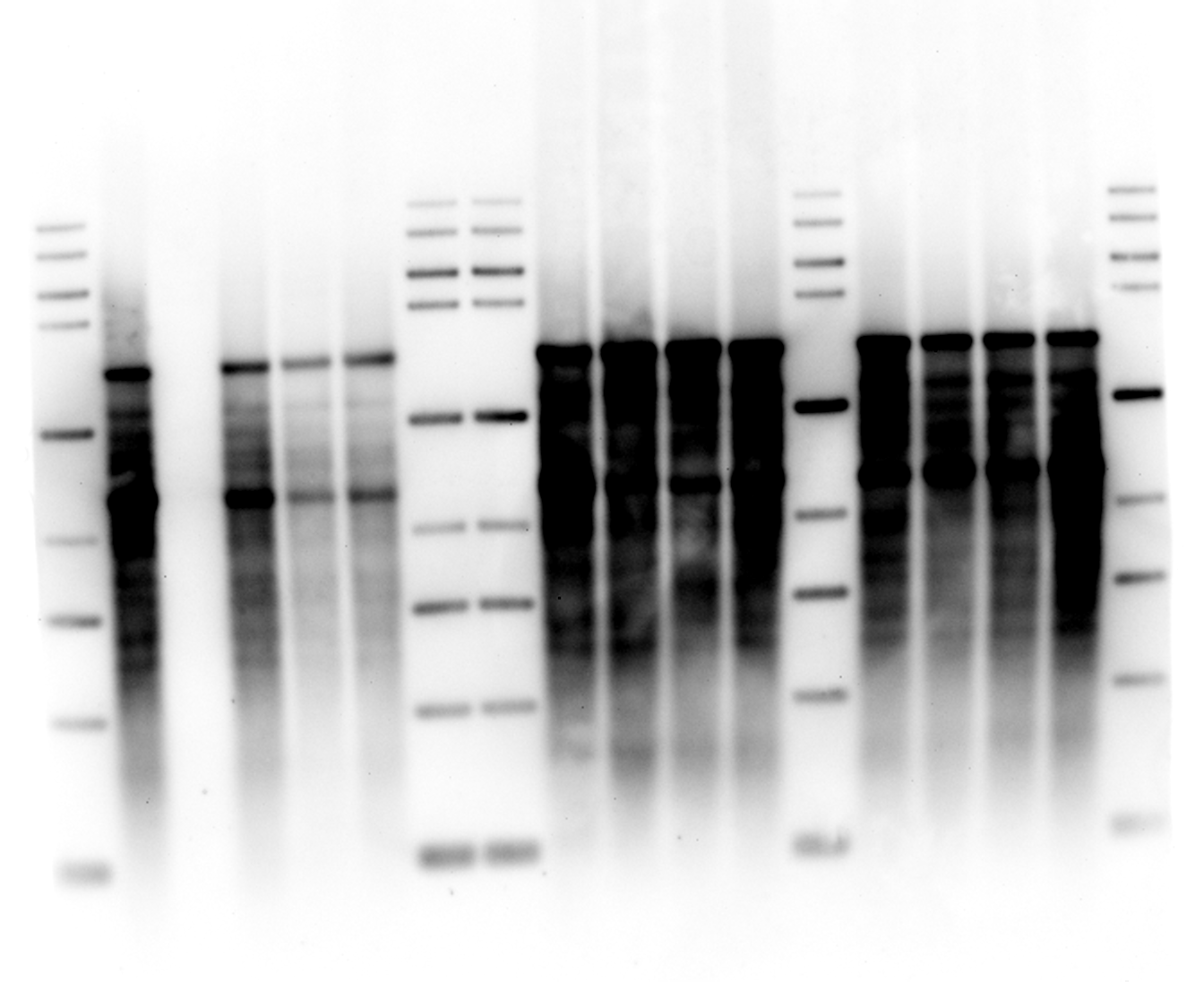

Supplement: S2 Dataset — (ZIP) [file ppat.1012800.s013.zip › 1-9 SFigs minimal data set/S8 fig/S8A fig.Tif]

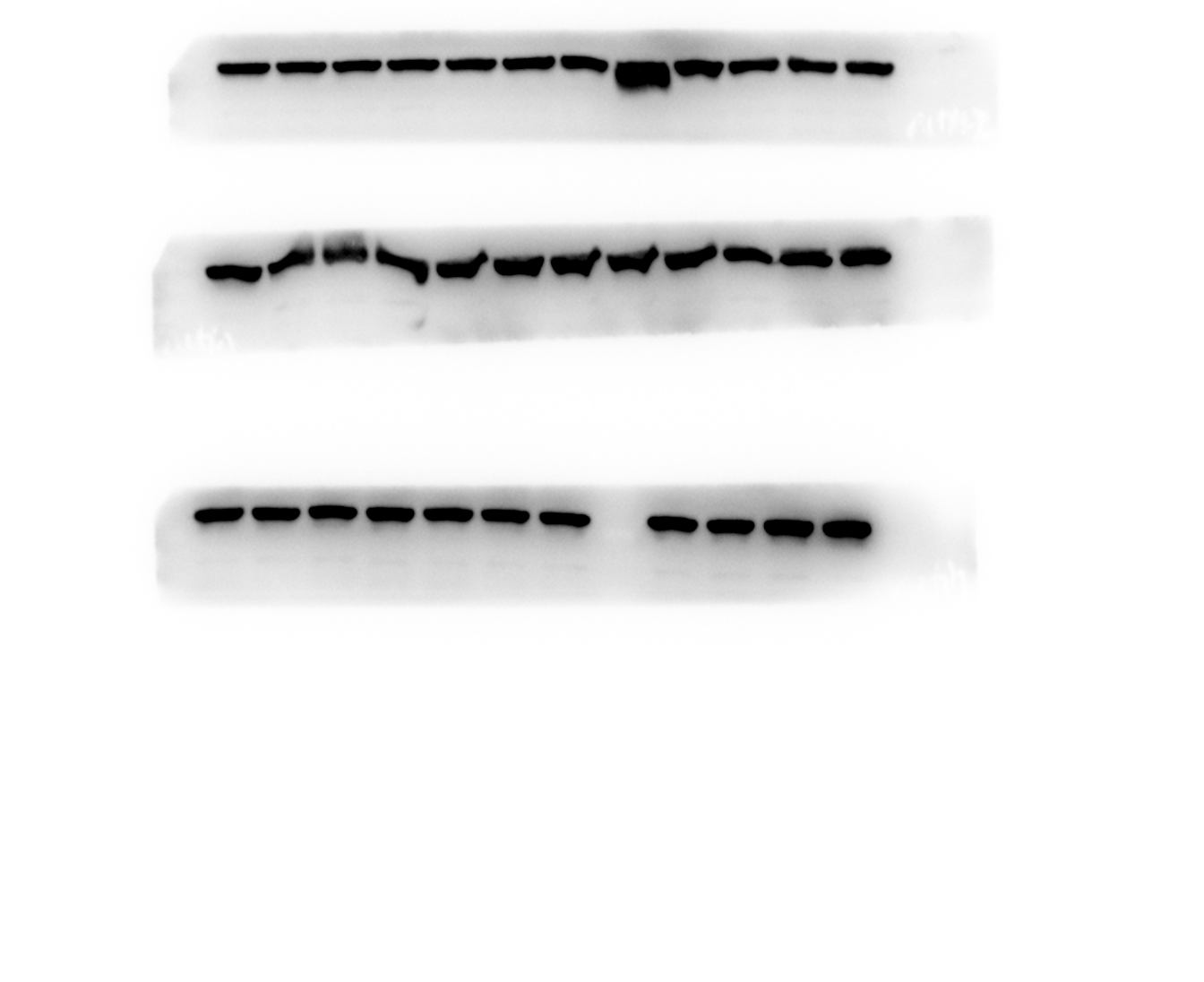

Supplement: S2 Dataset — (ZIP) [file ppat.1012800.s013.zip › 1-9 SFigs minimal data set/S9 fig/S9B fig/ACTB.Tif]

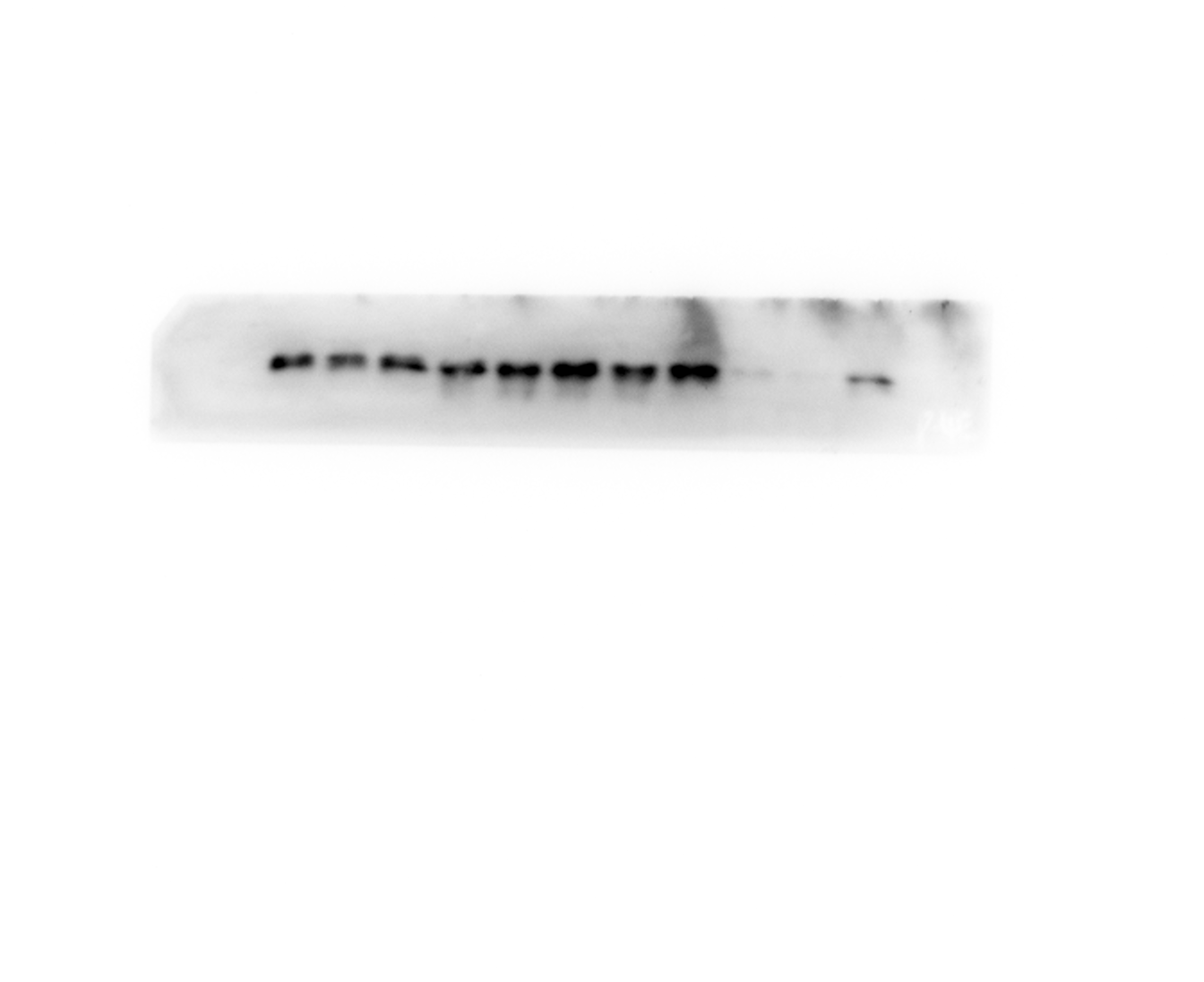

Supplement: S2 Dataset — (ZIP) [file ppat.1012800.s013.zip › 1-9 SFigs minimal data set/S9 fig/S9B fig/P-4EB-P1.Tif]

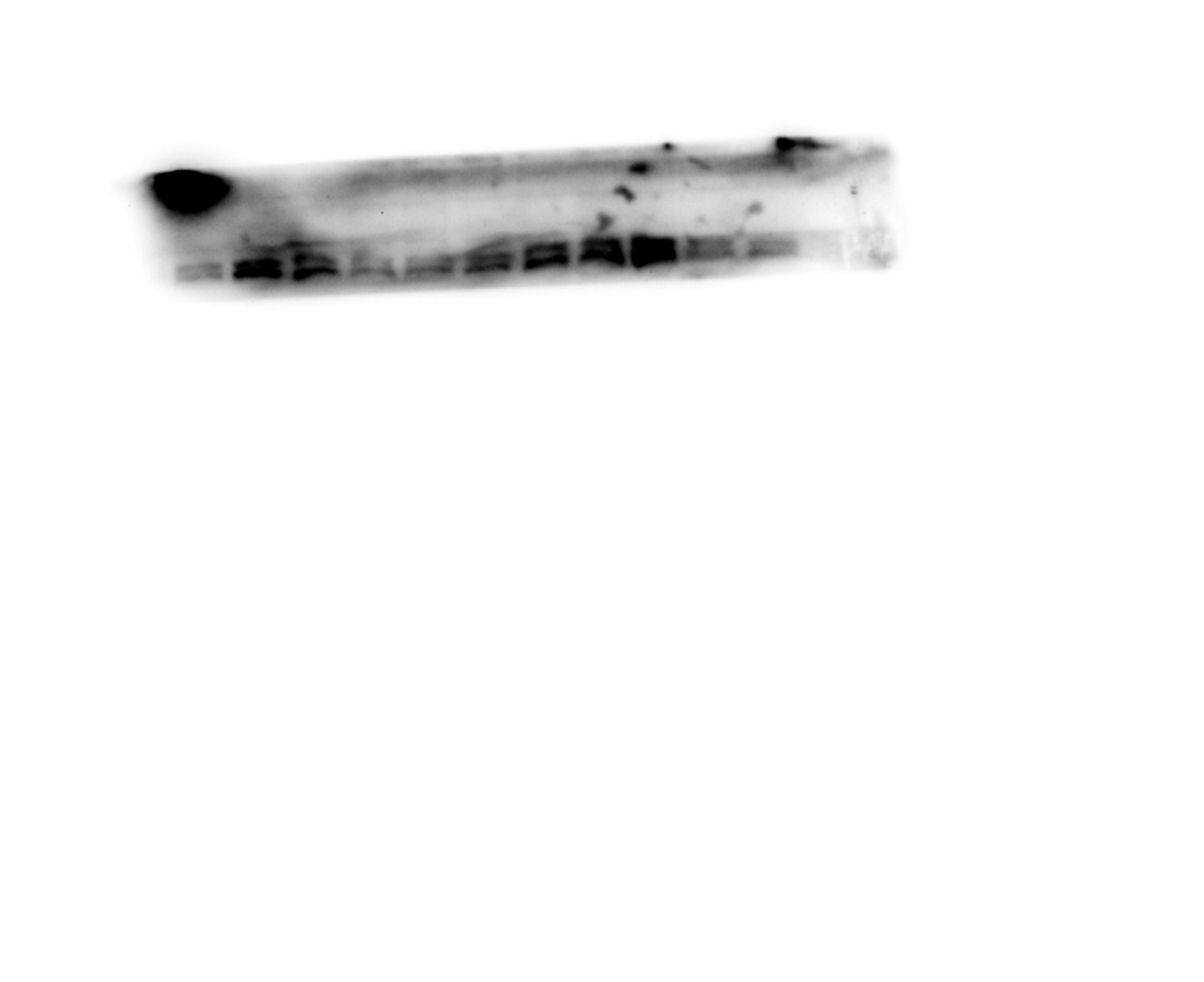

Supplement: S2 Dataset — (ZIP) [file ppat.1012800.s013.zip › 1-9 SFigs minimal data set/S9 fig/S9B fig/P-AKT.Tif]

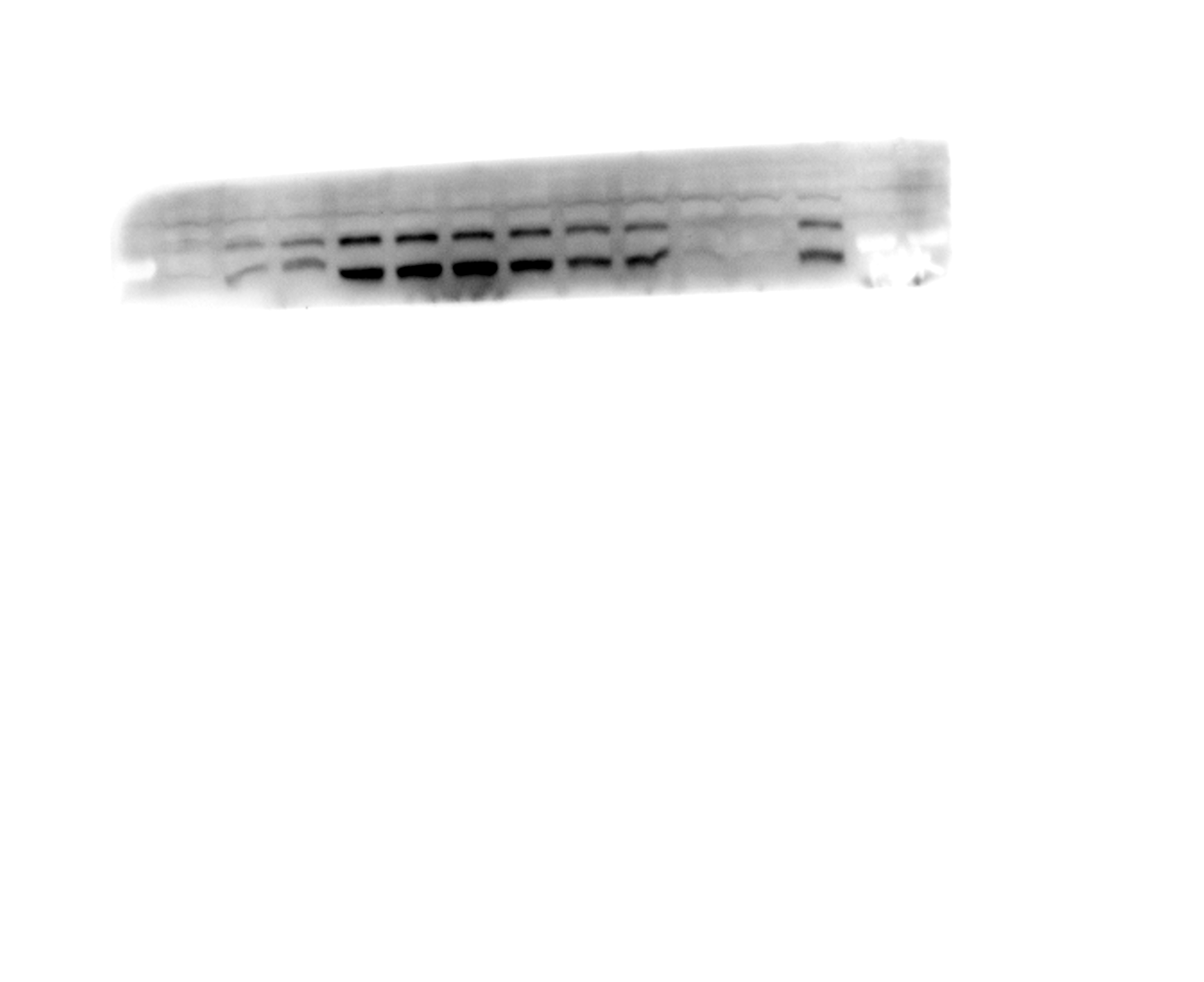

Supplement: S2 Dataset — (ZIP) [file ppat.1012800.s013.zip › 1-9 SFigs minimal data set/S9 fig/S9B fig/P-S6K.Tif]

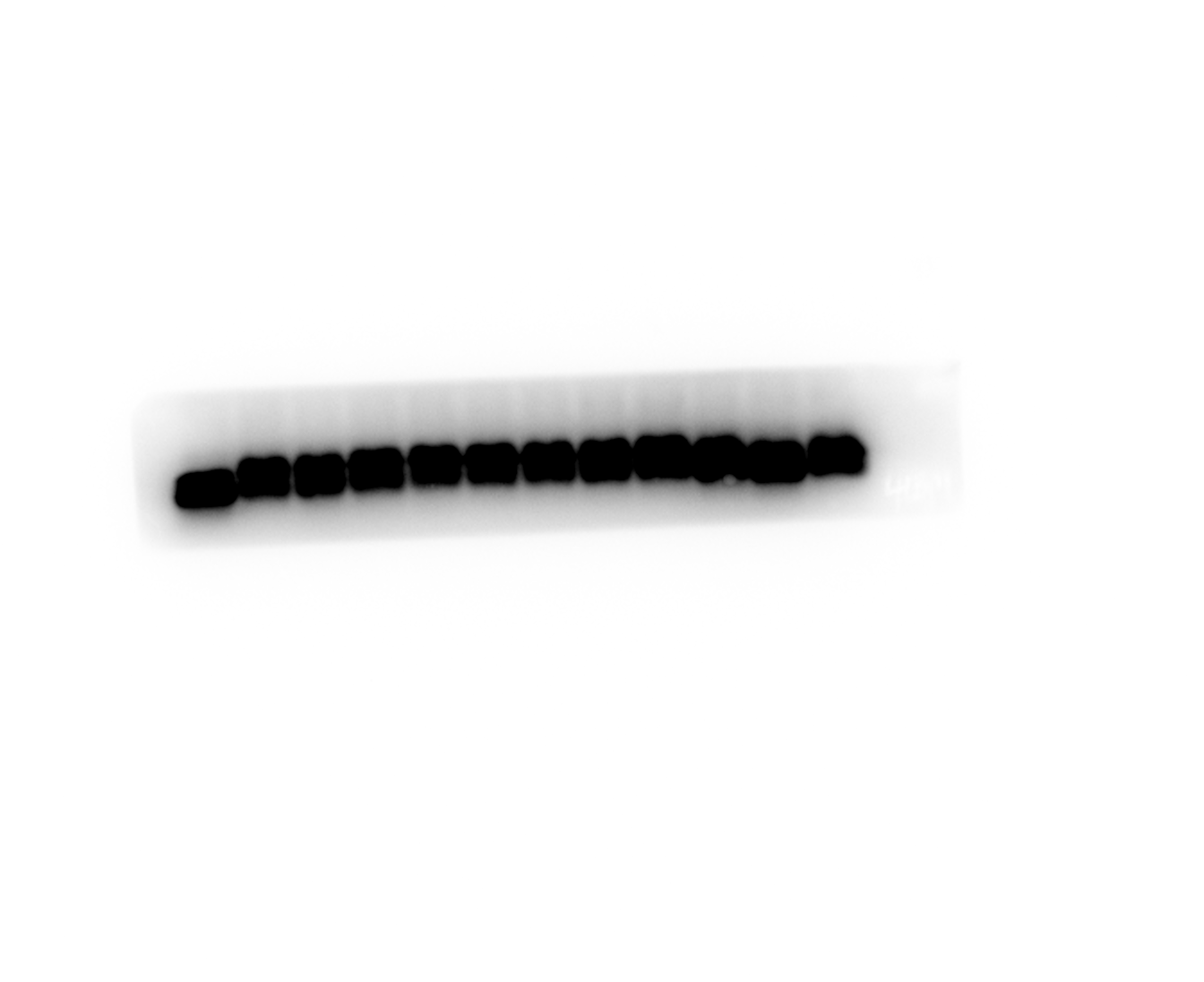

Supplement: S2 Dataset — (ZIP) [file ppat.1012800.s013.zip › 1-9 SFigs minimal data set/S9 fig/S9B fig/total 4E-BP1.Tif]

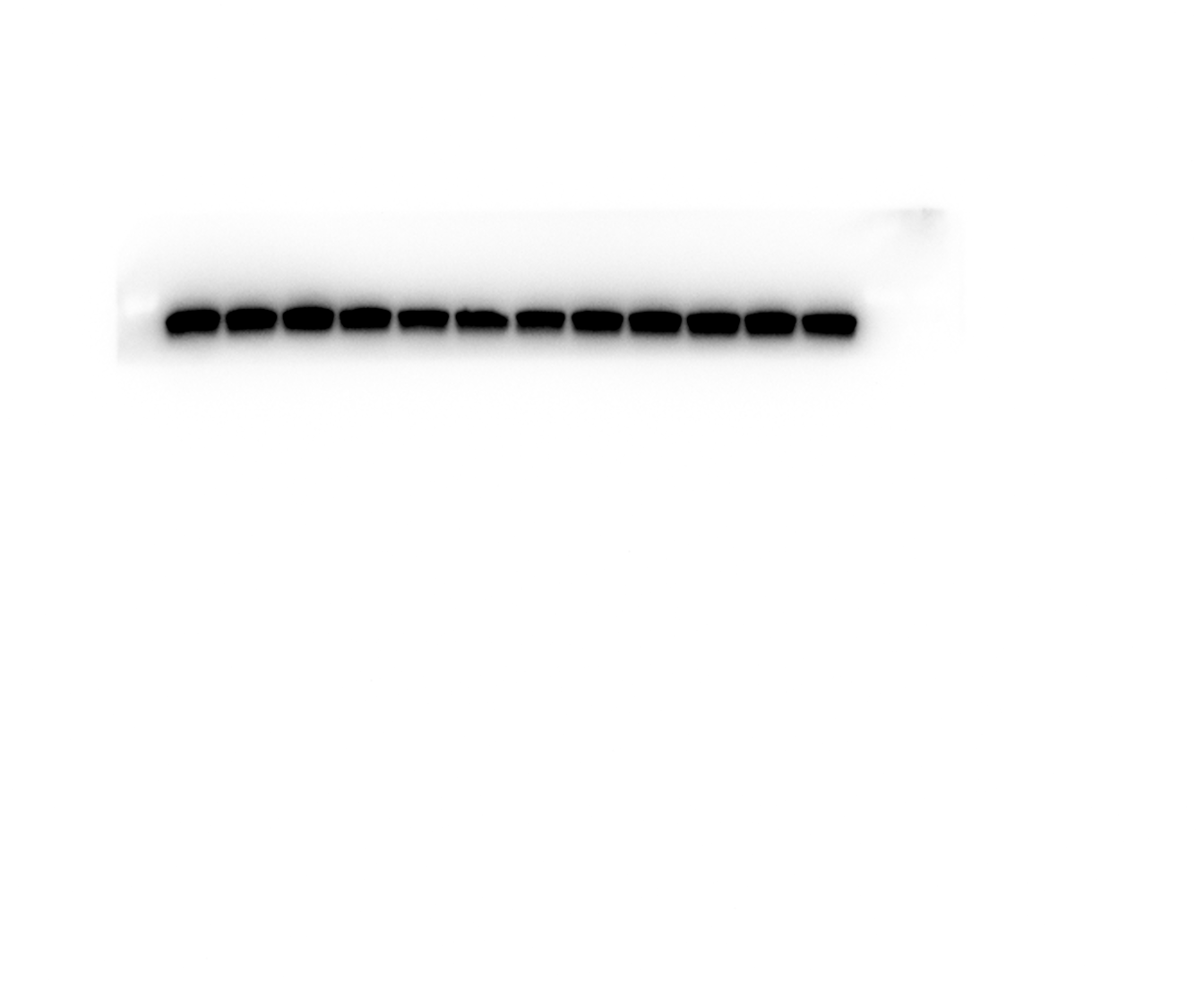

Supplement: S2 Dataset — (ZIP) [file ppat.1012800.s013.zip › 1-9 SFigs minimal data set/S9 fig/S9B fig/total AKT.Tif]

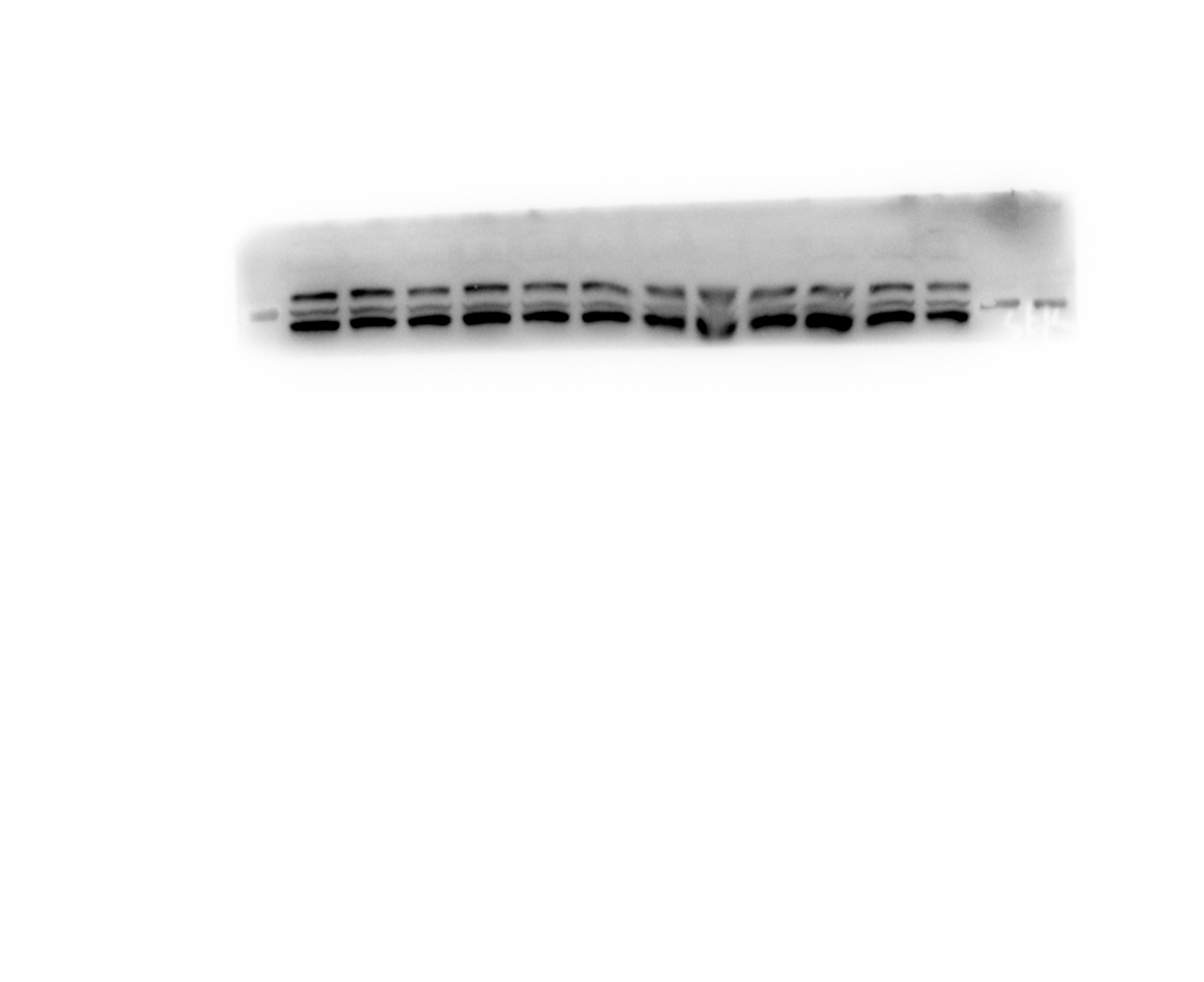

Supplement: S2 Dataset — (ZIP) [file ppat.1012800.s013.zip › 1-9 SFigs minimal data set/S9 fig/S9B fig/total S6K.Tif]
