# Supplementary material for: Manganese is a potent inducer of lysosomal activity that inhibits de novo HBV infection
Source: PLoS Pathog. 2025 Jan 2;21(1):e1012800. doi: 10.1371/journal.ppat.1012800 (PMC11694974; doi:10.1371/journal.ppat.1012800)
Supplement: Attachment — Submitted filename: Response to Reviewers.pdf [file ppat.1012800.s014.pdf]

Shanghai, November 4 2024

Re: PLOS Pathogens: Decision on your submission PPATHOGENS-D-24-01711 - [EMID:605f52921c5d4a0a]

Dear Editors:

We appreciate the valuable comments from all the three referees on our manuscript of PPATHOGENS-D-24-01711 entitled “Manganese is a potent inducer of lysosomal activity that inhibits de novo HBV infection”.

The reviews were very helpful and we have incorporated them into the revised manuscript. To address the reviewers' concerns, we included some new data in Figures **2E** and Supplementary Figures **S2E**, **S4C**, **S4D**, **S4E**, **S5D**, **S5E**, **S5G**, **S6C**, and **S6D**. We have also made revisions on the figure organization of Figures **1E**, **8F**, and **9**. The entire text has been carefully revised on the basis of the reviewers' comments.

We hope that, with these revisions, our manuscript will fulfill the requirements for acceptance. Please find enclosed our point-by-point response to the reviewers. We would be happy to receive further suggestions for improving the paper.

Thank you for your consideration. We look forward to hearing from you.

Sincerely,

Dr. Qiang Deng
Fudan University,
Fosun Bldg,
131 Dong-An Rd,
Shanghai 200032
Phone: (86) 21 5423 7542
qdeng@fudan.edu.cn

Point-to-Point Response to Reviewer Comments

Part I – Summary

Reviewer #1:

This is an interesting work on the late step of HBV entry process (endosomal escape/membrane fusion), which remains poorly understood following the discovery of NTCP as the functional HBV receptor more than a decade ago. First, the authors tested effect of manganese (Mn++) on HBV infectivity. Although Mn++ did not induce interferon response, it could still inhibit HBV infectivity in HepG2-NTCP cells. It was most effective if added before and/or during virus inoculation, and had little effect if added post-inoculation. Thus it affected the step of viral entry. Subsequent RNA-Seq followed by pathway analysis led them to test and validate mTORC1 as an Mn++ target and mediator of its inhibition of HBV infection. Thus reducing mTORC1 via serum deprivation or specific inhibitors (Torin1 and PP242) rather increased HBV infectivity in HepG2-NTCP cell line. Additional host factors in the early/late endosome and endosome/lysosome were examined. Chemicals that prevent acidification, including chloroquine and NH4Cl, improved HBV infectivity. Further studies confirmed that lysosomal activity is detrimental to HBV infectivity. Besides manganese, the authors also studied tomatidine, another activator of mTORC1. It moderately reduced HBV infectivity in both HepG2-NTCP cells and proliferating human hepatocytes (proliHH). In addition, the promoting effect of chloroquine and NH4Cl on HBV infectivity was also validated in proliHH. Overall, the studies were highly professional with complete data set, with the effect of mTORC1 demonstrated by both its stimulators (Mn++ and tomatidine) and inhibitors (FBS-free medium, Torin 1 and PP242). The work also shed light on why removing 10% FBS in the culture medium during HBV inoculation improves HBV infectivity.

Reviewer #2:

Lin Yu and colleagues report in this manuscript that MnCl2 inhibits HBV infection of human hepatoma cells expressing NTCP via promotion of endocytosed HBV virions to be sorted into lysosomes for degradation. Mechanistically, the authors provide strong experimental evidence supporting the notion that MnCl2 treatment efficiently increases endocytic trafficking and endolysosomal acidification. The work significantly advances our understanding of the post-endocytosis events and regulation of HBV entry into hepatocytes.

Overall, the study is well conceived and executed. The conclusions are supported by the data presented. Further language editing should improve the manuscript.

My comments and suggestions are mostly minor and mainly for more clear/logical presentation and thorough discussion.

Reviewer #3:

In this manuscript, Yu and colleagues serendipitously discovered that manganese (Mn^{2+}) inhibits HBV infection in a cGAS/STING-independent but lysosomal activity-dependent manner. Using Mn^{2+} and its functional mimetics tomatidine as chemical

probes, the authors further uncovered an ESCRT-mediated HBV post-entry sorting mechanism involving late endosomal escape. Along with this, activation of mTORC1 and subsequent endosome-lysosome fusion is detrimental to HBV infection, while inhibiting lysosome formation facilitates the process. Overall, this study provides novel and interesting insights into the cellular regulation of HBV post-entry trafficking and suggests that this mechanism could be exploited to develop new antiviral strategies for treating HBV infection. The presented results are largely compelling and supportive of the conclusions. The manuscript is also well-written together with well-organized figures. The following comments are provided for the authors to further strengthen the manuscript.

We would like to thank the three reviewers for their recognition and encouragement of our work. We have revised the manuscript according to the reviewers' comments, and below are our point-by-point responses to their feedback.

Part II – Major Issues: Key Experiments Required for Acceptance

Reviewer #1:

Ref. 10 (Macovei A et al., J Virol 2013; 87: 6415-6427) also characterized the early steps of HBV entry process, but they used differentiated HepaRG cells rather than HepG2/NTCP cells. The current study validated finding from that report that RAB7 is essential for HBV infection (Fig. S5A). On the other hand, the previous work found treatment with 50mM NH4Cl did not affect HBV infectivity (they did not study chloroquine), whereas in this study, NH4Cl at 10mM or 5mM promoted HBV infection of HepG2-NTCP cells (Fig. 4E) or proliHH (Fig. 8E). Chloroquine had similar promotion of HBV infection. One possible explanation for the discordant findings is the different cell types used. Alternatively, the time point of treatment was different. In Ref. 10, NH4Cl was added 24hrs post-inoculation and the duration of treatment was 24hrs. In the present study, cells were pretreated with NH4Cl for 10hrs, followed by HBV inoculation for 12hrs (with or without NH4Cl? Not clearly indicated). What if the authors use the same schedule as the previous work (with NH4Cl added 24hrs post-inoculation rather than prior to inoculation)? If no promotion is observed, then the discordance could be attributed to different schedules.

We thank Reviewer #1 for his careful reading of our manuscript and the valuable suggestions. In the study by Macovei et al., the authors removed the inoculum 24 h after HBV infection of differentiated HepaRG cells and then waited another 24 h before NH_4Cl treatment. This protocol is comparable to the post-infection treatment described in **Fig. 1B** of our manuscript. In response to the reviewer's comment, we conducted an additional experiment and found that 24 h after the removal of the HBV inoculum, treatment with NH_4Cl (for another 24 h) did not increase the efficiency of viral infection in HepG2-NTCP cells. This result is now included as **Fig. S5G** in the revised manuscript, and we have added a discussion of this finding:

This result seems to contrast with the previous finding from HBV-infected differentiated HepaRG cells, most likely due to the different treatment protocols used for NH_4Cl administration (**lines 303 to 305**).

Reviewer #2:

It will be interesting to experimentally demonstrate that MnCl2 and tomatidine treatment increase the degradation of internalized HBV protein and/or DNA in infected HepG2-NTCP cells.

We appreciate the reviewer's insightful comments and suggestions. In the present study, we utilized EGF to stimulate EGFR internalization and subsequent lysosomal degradation, demonstrating that both Mn^{2+} and tomatidine can enhance this process (**Fig. 5B** and **S7A**). Additionally, we showed that HBV induces the NTCP-EGFP-mCherry fusion protein to enter late endosomes (LE) via the ESCRT pathway, while the administration of mTORC1 inhibitors significantly accelerates the endocytic trafficking of this protein (**Fig. 3C** and **3G**). However, we did not directly demonstrate that Mn^{2+} or tomatidine promote the degradation of HBV/NTCP.

Quantification of the internalized virus is technically challenging, particularly given the low efficiency of HBV infection in HepG2-NTCP cells (as well as the non-specific absorption of virions on the cell surface). In response to Reviewer #2's suggestion and also considering Reviewer #3's comments, we conducted the following additional experiment: NTCP-EGFP-expressing Huh7 cells were mock treated, or treated with $MnCl_2$ or tomatidine for 10 h. After inoculating with HBV for 1 h, NTCP-EGFP, HBsAg, and the endolysosomal marker LAMP1 exhibited partial colocalization, primarily in the perinuclear regions. As anticipated, this colocalization was significantly diminished in cells treated with $MnCl_2$ or Tomatidine. Concurrently, there was a notable reduction in intracellular puncta positive for HBsAg. $MnCl_2$ treatment did not markedly reduce total NTCP protein levels during HBV infection in HepG2-NTCP cells, likely due to the constitutive overexpression of NTCP, with only a small fraction undergoing HBV-triggered endocytosis and lysosomal trafficking. These results have been included in **Fig. S6C and S6D** of the revised manuscript.

Reviewer #3:

1. There is somewhat a disconnection between Figs.1-2 and Figs.3-4. In Fig.3, the authors demonstrated that mTOR suppression led to an increase in HBV-induced MVB formation. How was this connected to Mn2+ treatment? Why not directly treat with Mn2+ and then use an mTOR inhibitor as a control group? In Fig.4, VAMP7 was used to represent endosome-lysosome fusion. How is VAMP7 related to Mn2+ or mTOR? After siVAMP7 treatment, does Mn2+ still have an effect? Is mTOR activated? Some interesting information comes from the use of other inhibitors. Were these inhibitors applied continuously or only in the pre-infection stage? Furthermore, clarification is needed for the STX17-SNAP47-VAMP7/VAMP8 complex and its involvement in mTOR signaling.

We appreciate the valuable comments and suggestions from reviewer #3.

1). Under normal cell culture conditions (nutrient-rich), mTORC1 remains in an activated state. Our experiments in **Fig. 2** demonstrate that treatment of cells with Mn^{2+} results in even greater activation of mTORC1. It was unclear whether such mTORC1 hyperactivation is related to the inhibition of *de novo* HBV infection by Mn^{2+}. Therefore, we initially employed a **loss-of-function** strategy to determine whether inhibiting mTORC1 would subsequently promote HBV infection (**Fig. 3**). In contrast, the final **Figs 7, 8,** and **S9** utilized tomatidine (Td) and α-tomatine (α-

Tt), functional mimetics of Mn^{2+}, to explore whether mTORC1 hyperactivation could inhibit HBV infection (**gain-of-function**).

2). In response to Reviewer #3's suggestion, we transfected Huh7 cells with a plasmid expressing NTCP-EGFP-mCherry and inoculated them with HBV following 2 h of Mn^{2+} treatment (corresponding to peak mTORC1 activation, though lysosomal acidification was not yet increased). The results showed that Mn^{2+} treatment did not significantly reduce the formation of red-only puncta. Therefore, while mTORC1 inhibitors significantly promoted the endocytic trafficking of HBV/NTCP, the hyperactivation of mTORC1 induced by Mn^{2+} did not lead to the opposite process (new **Fig. S4D, S4E,** and **lines 250 to 254** in the revised manuscript).

This finding aligns with the results from the α-tomatine study (**Fig. S9**), suggesting that the level of endo/lysosomal acidification, rather than mTORC1 hyperactivation, is the primary factor influencing the efficiency of HBV infection.

3). The mTORC1 inhibitor Torin1 (**Fig. 2E**) and PP242 (data not shown), which act on the catalytic center of enzyme activity, almost fully suppressed mTORC1 activation even in the presence of $MnCl_2$ treatment (10-100 μM) (please see the new **Fig. 2E**). Therefore, we did not co-treat cells with Mn^{2+} and mTORC1 inhibitors in subsequent studies.

4). The knockdown of VAMP7 had limited effect on Mn^{2+}-induced hyperactivation of mTORC1 (new **Fig. S5B**); in HepG2-NTCP cells pre-transfected with siVAMP7, Mn^{2+} still significantly inhibited *de novo* HBV infection (new **Fig. S5C**).

5). VAMP7 and syntaxin 7 (STX7) are complementary SNAREs involved in late endosome-lysosome fusion, as reported in the literature (Pryor PR, et al. Embo Rep. 2004). Consistent with the results from the VAMP7 knockdown experiments, knocking down STX7 also inhibits the maturation of late endosomes, but does not have a significant impact on de novo infection of HBV. This result has been included as supplementary data in the revised manuscript (new **Fig. S5D and S5E**).

6). Previous studies have shown that the STX17-SNAP47-VAMP7/VAMP8 complex mediates autophagosome-lysosome fusion; however, there is no literature supporting its role in endosome-lysosome fusion. In response to the reviewer's comment, we conducted experiments and observed that STX17 knockdown slightly inhibited HBV infection. Interestingly, STX17 knockdown also modestly reduced mTORC1 activity under nutrient-rich conditions (as shown in the figure below). It should be noted that STX17 knockdown cells showed slower growth and appeared less healthy.

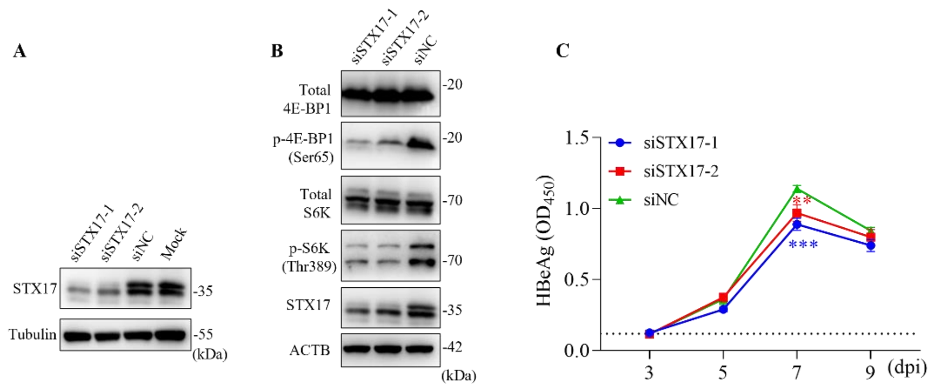

*Unpublished figure provided specifically for Reviewers: (**A**) The knockdown efficiency of individual siRNAs targeting STX17 was assessed by western blotting. (**B**) HepG2-NTCP cells were transfected with the indicated siRNAs for 48 h, followed by western blot analysis using the specified antibodies. (**C**) HepG2-NTCP cells with STX17 knockdown, as described in (B), were inoculated with HBV for 12 h. Secreted HBeAg in the supernatant was measured by ELISA at the indicated time points.*

7). In the experiment of **Fig. 4C**, HepG2-NTCP cells were treated with Apilimod or YM201636 at the indicated doses for 10 h, followed by HBV infection for an additional 12 h in the presence of the inhibitors. Cells were then maintained in fresh media (without the inhibitors) for subsequent analysis. In response to the issue raised by Reviewer #3, we have revised the corresponding figure legends (**lines 1001 to 1003**).

2. While this study suggests a role of MVB/ESCRT in HBV post entry movement and cytosolic release, it is well-known that the MVB/ESCRT pathway is responsible for HBV virion secretion. Hence, the authors may want to check whether Mn2+ or tomatidine possesses any effect on HBV virion egress in their experimental system.

In response to the reviewer's suggestion, we have included a new experiment in the revised manuscript that examines HBV virion egress in HBV-transfected Huh7 cells following treatment with Mn^{2+} or tomatidine. As shown in the new **S2E Fig**, treatment with Mn^{2+} or tomatidine does not significantly affect the levels of virions, naked capsids, or viral DNA in the culture supernatant. Therefore, while mTORC1 inhibitors promote ESCRT-mediated MVB biogenesis, the Mn^{2+}/tomatidine-induced hyperactivation of mTORC1 does not impact HBV virion egress, despite the latter being closely related to the ESCRT mechanism (**lines 186 to 191**).

3. It would be ideal to graphically show the colocalization of HBV/NTCP complex and lysosomal marker(s) under Mn2+ treatment or mTORC1 activation.

This comment is similar to the major point raised by Reviewer #2. In response to both reviewers' comments, we conducted the following additional experiment:

NTCP-EGFP-expressing Huh7 cells were mock treated, or treated with $MnCl_2$ or tomatidine for 10 h. After inoculating with HBV for 1 h, NTCP-EGFP, HBsAg, and the endolysosomal marker LAMP1 exhibited partial colocalization, primarily in the perinuclear regions. As anticipated, this colocalization was significantly diminished in cells treated with $MnCl_2$ or tomatidine. Concurrently, there was a notable reduction in intracellular puncta positive for HBsAg, presumably due to enhanced lysosomal activity promoting the degradation of NTCP/HBsAg. These results have been included in **Figs. S6C and S6D** of the revised manuscript.

Part III – Minor Issues: Editorial and Data Presentation Modifications

Reviewer #1:

1. The Discussion section may add something on limitations of work performed in

vitro, mostly based on HepG2-NTCP cells. It will be interesting in the future to repeat the experiments of Mn++ and tomatidine treatment, VAMP7 knockdown, chloroquine and NH4Cl treatment, and bafilomycin A1 treatment in HepaRG cells or primary human hepatocytes. Moreover, all current in vitro HBV infection systems require artificial addition of 4% PEG, and a high genome copy number/cell. Thus one concern is whether the antiviral (therapeutic) effect of Mn++ and tomatidine observed in HepG2-NTCP cells can be observed in vivo (such as immunodeficient mice with humanized liver) under more authentic conditions of HBV infection. Under such conditions viral particles probably do not proceed to the endolysosome fraction.

The reviewers' comments are very helpful to us in improving our manuscript. Please find the following discussion in the revised manuscript:

Our study primarily relied on an *in vitro* model of *de novo* HBV infection using HepG2-NTCP cells. For certain functional analyses, we also utilized ProliHH organoids, which are derived from dedifferentiated primary human hepatocytes (PHHs) that regain mature hepatic properties after three-dimensional (3D) culture. This model offers advantages over hepatoma cell lines, as it more accurately simulates the natural HBV infection environment. However, challenges remain with the ProliHH organoid model, particularly regarding the complexity of molecular biology manipulations, such as DNA/siRNA transfection and confocal microscopy. Future investigations will still require the use of 2D-cultured PHHs to corroborate findings from our current experiments.

Additionally, during our study, we attempted to use HBV-infected chimeric mice with humanized livers to investigate the *in vivo* antiviral effects of tomatidine. Unfortunately, pharmacokinetic analyses revealed that tomatidine was rapidly cleared from the circulation after intraperitoneal or intravenous administration (data not shown), complicating the attainment of effective concentrations *in vivo*. Future research may focus on exploring tomatidine derivatives or other potential lysosomal activators for their anti-HBV efficacy in humanized mouse models (**lines 533 to 539**).

2. Fig. 9 summarizes the major findings from this work and is supposed to be the highlight. But the current diagram is difficult to follow even with the figure legend. Some writings are extremely small, with some words even inverted. The word “Hepatocyte” at the bottom is not needed. What is “EV” on top right?

We have revised the diagram in accordance with the comments and clarified that 'EV' refers to endocytic vesicles in the figure legends.

3. For supplementary figures, they should be called “Fig. S5” rather than “S5 Fig”.

We thank the reviewer for her/his careful examination of our manuscript. In fact, the format of the 'S5 Fig' is dictated by the guidelines of the journal *PLoS Pathogens*.

Reviewer #2:

1. Line 41, because virus entry into host cells is generally considered to include all the molecular and cellular processes from receptor binding at cell surface to the delivery of viral capsids into the cytoplasm. Accordingly, endocytic trafficking of internalized HBV virion particles and endosomal/lysosomal escaping should still be considered as the part of viral entry process, but not "the molecular events of the viral post-entry steps". More accurately, it should be post-endocytosis steps.

We appreciate the reviewer's thoughtful comment on our manuscript and have made corresponding revisions.

2. Line 47, put the sentence in a logic order, it should be "hyperactivation of mTORC1 and endo/lysosomal acidity".

We have made the corresponding correction in the revised manuscript based on the reviewer's comment.

3. Line 130, a reference should be cited to support the claim that "Unlike many other enveloped viruses that employ low pH to facilitate membrane fusion, raising endolysosomal pH or inhibiting endosome-lysosome fusion promoted HBV infection." If this statement is based on the findings reported in this manuscript, the paragraph should be rephrased to indicate this fact.

Indeed, many viruses, such as influenza virus, severe acute respiratory syndrome coronavirus (SARS-CoV), and vesicular stomatitis virus (VSV), fuse with host membrane structures due to pH-induced conformational changes in their envelope proteins. In response to the reviewer's comment, we have added a relevant reference in the revised manuscript: Harrison, S.C. 'Viral Membrane Fusion.' Nat Struct Mol Biol, 2008.

4. Logically, the data presented in Fig. 1 and Figs. S1 to S3 suggest MnCl2 treatment inhibits one or multiple steps of HBV infection post NTCP binding, but before viral RNA transcription. Therefore, the sentence in line 175 to 176 should be modified.

We appreciate the insightful comment by the reviewer. Accordingly, we have revised the sentence to: Thus, the anti-HBV effects of Mn^{2+} may occur at one or several stages of HBV infection following NTCP binding, yet prior to viral RNA transcription.

5. The data presented in Fig. 2C and D clearly indicate that MnCl2 treatment activates AKT-mTORC1 pathway in nutrient-rich condition. It will be very helpful to briefly interpret in the text why amino acid starvation completely abolishes the effects of MnCl2 activation of mTORC1 and downstream signaling pathway. Although not necessary, it will be very interesting to demonstrate or at least speculate/discuss how MnCl2 activates AKT.

We have incorporated the interpretations into the revised manuscript as recommended by the reviewer:

1. Activation of mTORC1 involves spatio-temporally coordinated events. The mTORC1 complex must localize to the endo/lysosomal membrane via an amino acid-dependent process, where its kinase activity is differentially stimulated by growth factor signaling (**lines 212 to 215**). Therefore, amino acid deprivation can fully abolish the effects of Mn^{2+} on mTORC1 activation and its downstream signaling pathways.
2. The KEGG analysis (**Fig. 2A**) shows significant activation of the MAPK pathway in Mn^{2+}-treated cells. This leads to the speculation that Mn^{2+}-induced cellular stress and MAPK activation may hyperactivate endo/lysosomal mTORC1 through intricate crosstalk between the Ras/MAPK and PI3K/AKT signaling pathways (**lines 223 to 227**).

6. It is very interesting that increased lysosomal acidification, but not activation of mTORC1, is more directly associated with the inhibition of HBV infection (Fig. S9). However, mTORC1 inhibitor efficiently promoted HBV infection (Fig. 3B). Why?

We appreciate the reviewer's thoughtful reading of our manuscript.

Mn^{2+}/tomatidine-induced lysosomal hyperfunction is characterized by two features: hyperactivation of mTORC1 and a significant increase in lysosomal acidity. In contrast, α-tomatine induces mTORC1 hyperactivation without significantly increasing lysosomal acidity. Since Mn^{2+}/tomatidine inhibits HBV infection while α-tomatine does not, we hypothesize that the increased lysosomal acidification induced by Mn^{2+}/tomatidine, rather than mTORC1 activation, is more directly associated with the inhibition of HBV infection.

The effectiveness of mTORC1 inhibitors in promoting HBV infection does not contradict the perspective outlined above. As shown in **Fig. 3**, mTORC1 inhibition accelerated the HBV-stimulated endocytic transport of NTCP. However, in this context, many invading virions would be rapidly degraded in endolysosomes. Interestingly, additional findings presented in **Fig. 5** indicate that prolonged mTORC1 inactivation led to lysosomal depletion, which proved to be advantageous for HBV infection. This result in turn supports our hypothesis that Mn^{2+}/tomatidine-induced lysosomal acidification plays a critical role in preventing HBV infection.In response to the reviewer’s comment, we have added this discussion to the revised manuscript (**lines 473 to 479**).

Reviewer #3:

1. Fig.1D-E, the figure labeling is unclear. Are the results represented in two replicates? Why was 100 μM not included? Was the treatment a one-time thing or repeated?

In response to the reviewer comments, we have modified the corresponding figure legends. The Southern blot and immunoblot analyses in **Fig. 1D-E** were performed with two replicates at each concentration of $MnCl_2$, representing three independent experiments (**lines 931 to 933**). In separate experiments, we treated cells with 0, 40, and 100 μM $MnCl_2$, yielding similar results (data not shown). For consistency of figure organization, we have removed the data for 100 μM $MnCl_2$ from the ELISA

analysis in **Fig. 1E** of the revised manuscript.

2. The activation of PI3K/AKT-mTOR has been shown to inhibit HBV replication by several previous studies, while this study did not observe direct antiviral effect on HBV replication upon Mn2+-induced AKT-mTOR activation (Fig. 2). Such discrepancy needs an explanation or discussion. The bottom panel of Fig. 2C shows a biphasic phosphorylation of AKT, any explanation?

We appreciate the insightful comment by the reviewer. It was previously reported that constitutively active AKT1 significantly inhibited HBV RNA transcription and, consequently, reduced HBV DNA replication in HepG2 cells (Guo Haitao, et al. *J Virol*, 2007). In our study, however, Mn^{2+}-induced AKT–mTORC1 activation did not have a similar antiviral effect (**Fig. 1D** and **Fig. S2B**), presumably due to its lower potency and shorter duration compared to the sustained activation achieved by transient transfection with the AKT mutant (**lines 480 to 485** in the revised manuscript).

For the second question, we do not have a definitive explanation. The experiment in **Fig. 2C** was repeated four times, and two of those showed a similar biphasic phosphorylation of AKT. Interestingly, a similar biphasic phosphorylation of AKT was also observed in tomatidine-treated cells (**Fig. 7B**), presumably due to a dynamic cellular feedback mechanism following Mn^{2+}/tomatidine treatment.

3. Fig.S3, did the authors see a reduction in total NTCP protein in HBV-infected HepG2-NTCP cells upon Mn2+ treatment?

In response to the reviewer's question, we conducted an additional experiment. HepG2-NTCP cells were inoculated with HBV and treated with 40 µM Mn^{2+}, and NTCP expression was examined at various time points. The results showed no significant change in total NTCP levels, likely due to the constitutive overexpression of NTCP, with only a small proportion undergoing HBV-induced endocytosis and lysosomal trafficking. This result has been included in the revised manuscript as **Fig. S6E**.

In addition, please refer to our response to the major point 3 by Reviewer #3 for further details.

4. Fig.S4, what is the concentration of Mn in FBS? Does it matter?

In human, normal Mn^{2+} levels in serum range from approximately 0.4-1.0 µg/L (7.3-18.2 nM). The levels of Mn^{2+} in fetal bovine serum (FBS) are believed to be in a similar range. At concentrations below 1 µM, Mn^{2+} activation of mTORC1 and stimulation of lysosomal acidification were not prominent (data not shown).

5. Fig.8F, right panel, please include an uninfected control. Do the authors have additional data to support a productive HBV infection in the ProliHH organoids such as intracellular HBV RNA and DNA?

In the previous manuscript version, we omitted data on uninfected ProliHH organoids due to space constraints. In the revised manuscript, we have restructured **Fig. 8** to include this important control.

The ProliHH-derived 3D organoids were susceptible to HBV infection, as indicated by the presence of intracellular HBc and secreted HBeAg over time following viral inoculation. (**Fig. 8E and 8F**). We also found that treatment with CQ or NH_4Cl significantly increased *HBV DNA* levels in the culture supernatant of infected ProliHH organoids, as measured by quantitative PCR (**Fig. 8E**).

The challenge in detecting intracellular HBV DNA lies in the strong adsorption of viral particles on or within 3D spheres, which appear to be resistant to entecavir treatment and difficult to wash away (as shown in the figure below). For this reason, we did not include these data in the manuscript.

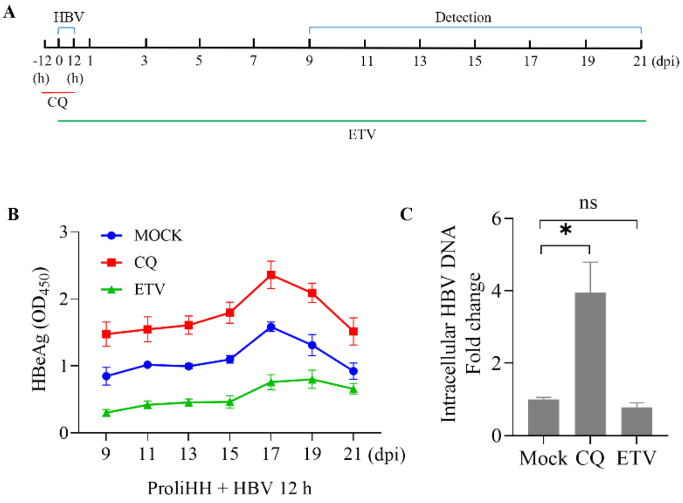

*Unpublished figure provided specifically for Reviewers: ProliHHs treated with CQ (10 μM) or entecavir (ETV, 5 μM) were inoculated with HBV as shown in (**A**). Secreted HBeAg in the supernatant was measured by ELISA at the indicated time points (**B**). Intracellular HBV DNA was quantified by qPCR at 21 dpi (**C**).*

6. While Mn2+ did not inhibit HBV DNA replication in HepAD38 cells (Fig. S2B), tomatidine treatment resulted in an obvious reduction of HBV DNA in the same cells (Fig. S8A). The authors may discuss the possible reason for such discrepancy. Is it related to the different degrees of mTORC1 activation between these two chemicals?

$MnCl_2$ is a metal ion compound, while tomatidine is a natural steroidal alkaloid. Although both compounds exhibit a common characteristic in promoting lysosomal hyperfunction, they are fundamentally distinct entities. Even in terms of enhancing lysosomal activity, tomatidine markedly facilitates the assembly of v-ATPase complexes—an attribute that Mn^{2+} lacks. The differences in their physicochemical properties may correlate with different degrees of mTORC1 activation, or may contribute to their divergent effects on HBV replication independent of the lysosomal hyperfunction (**lines 507 to 512** in the revised manuscript).

7. Line 106, "primary" should be "primarily".

We have made the corresponding correction in the revised manuscript.
